# The Impact of Cand1 in Prostate Cancer

**DOI:** 10.3390/cancers12020428

**Published:** 2020-02-12

**Authors:** Andrea Eigentler, Piotr Tymoszuk, Johanna Zwick, Arndt A. Schmitz, Andreas Pircher, Florian Kocher, Andreas Schlicker, Ralf Lesche, Georg Schäfer, Igor Theurl, Helmut Klocker, Isabel Heidegger

**Affiliations:** 1 Department of Urology, Medical University of Innsbruck, 6020 Innsbruck, Austria; andrea.eigentler@i-med.ac.at (A.E.); johanna.zwick@rolmail.net (J.Z.); helmut.klocker@i-med.ac.at (H.K.); 2 Laboratory for Immunotherapy, Department of Internal Medicine II, Medical University of Innsbruck, 6020 Innsbruck, Austria; piotr.tymoszuk@i-med.ac.at (P.T.); igor.theurl@i-med.ac.at (I.T.); 3 Bayer AG, Research & Development, Pharmaceuticals, 13353 Berlin, Germanyandreas.schlicker@bayer.com (A.S.); ralf.lesche@bayer.com (R.L.); 4 Department of Internal Medicine V, Medical University of Innsbruck, 6020 Innsbruck, Austria; andreas.pircher@i-med.ac.at (A.P.); florian.kocher@i-med.ac.at (F.K.); 5 Department of Pathology, Medical University of Innsbruck, 6020 Innsbruck, Austria; georg.schaefer@i-med.ac.at

**Keywords:** Prostate cancer, Cand1, cancer aggressiveness, survival, tumor recurrence, mutation, enzalutamide resistance

## Abstract

Evidence has accumulated asserting the importance of cullin-RING (really interesting new gene) ubiquitin ligases (CRLs) and their regulator Cullin-associated neural-precursor-cell-expressed developmentally down-regulated 8 (NEDD8) dissociated protein 1 (Cand1) in various cancer entities. However, the role of Cand1 in prostate cancer (PCa) has not been intensively investigated so far. Thus, in the present study, we aimed to assess the relevance of Cand1 in the clinical and preclinical setting. Immunohistochemical analyses of radical prostatectomy specimens of PCa patients showed that Cand1 protein levels are elevated in PCa compared to benign areas. In addition, high Cand1 levels were associated with higher Gleason Scores, as well as higher tumor recurrence and decreased overall survival. In line with clinical findings, *in vitro* experiments in different PCa cell lines revealed that knockdown of Cand1 reduced cell viability and proliferation and increased apoptosis, therefore underlining its role in tumor progression. We also found that the cyclin-dependent kinase inhibitor p21 is significantly upregulated upon downregulation of Cand1. Using bioinformatic tools, we detected genes encoding for proteins linked to mRNA turnover, protein polyubiquitination, and proteasomal degradation to be significantly upregulated in Cand1^high^ tumors. Next generation sequencing of PCa cell lines resistant to the anti-androgen enzalutamide revealed that *Cand1* is mutated in enzalutamide-resistant cells, however, with little functional and clinically relevant impact in the process of resistance development. To summarize the present study, we found that high Cand1 levels correlate with PCa aggressiveness.

## 1. Introduction

Prostate cancer (PCa) remains one of the leading causes of death among men in Western countries [1]. Organ-confined PCa can often be cured predominantly either by radical prostatectomy or by primary radiation therapy, however about 15% of patients are diagnosed in a primary metastatic stage [2]. In addition, it has been demonstrated that up to 50% of patients relapse after primary treatment over time claiming for an androgen deprivation therapy [3,4]. However, after a time of about 2–3 years, patients experience resistance to androgen deprivation therapy (metastatic castration-resistant prostate cancer, mCRPC). In the past five years, several new therapeutic options targeting the androgen receptor (AR)-signaling axis have been developed for this patient population, including the AR-inhibiting agent enzalutamide [5,6]. Response to these drugs, however, is only temporary, as the majority of treated patients develop drug resistance. Currently, the biological mechanisms of drug resistance are still under investigation.

The cullin-RING ligases (CRLs) comprise the largest class of E3 ubiquitin ligases. As part of the ubiquitin-proteasome system, CRLs mediate the ubiquitination of protein substrates destined for proteasomal degradation and are thus responsible for posttranslational regulation of numerous targets [7,8]. CRLs are multi-protein complexes with a common catalytic core consisting of a member of the cullin-family (CUL1-3, 4A, 4B, 5, 7 in human) and a RING (really interesting new gene) protein, also known as RBX1 (RING box protein-1) or ROC1 (regulator of cullins-1) [9]. Adaptor proteins, which recruit a variety of different receptors, link specific substrates to the catalytic core, while the RING subunit provides a docking site to the ubiquitin conjugating enzyme E2. Besides the regulation of substrate recruitment by the variety of receptor subunits and the required post-translational modifications of the targets, the activity of CRLs is further modulated by the NEDD8/Cand1 cycle [10]. In a process named neddylation, the NEDD8 protein is covalently bound to the CUL subunit of the enzymatic core by the NEDD8-conjugating enzyme UBC12, resulting in the activation of the CRL. By the intrinsic deneddylating activity of the constitutive photomorphogenesis 9 signalosome (CSN), NEDD8 is removed, leading to the dissociation of adapter protein and substrate-receptor. Deneddylated cullins are subsequently sequestered by the cullin-associated NEDD8-dissociated protein 1 (Cand1) and held in an inactive state. NEDD8 conjugation again causes the dissociation of Cand1, and thus activation of the ligase [8]. It has been previously shown that suppression of Cand1 results in increased CRL activity *in vitro* [11,12,13]. Generally, aberrant regulation of the ubiquitin system is associated with the development and progression of several cancer entities, including urological malignancies [14,15]. Interestingly, depending on the CRL activity and the bound substrate-receptor, RING-ligases can have both oncogenic and tumor-suppressive properties [16,17,18].

In the present study, we therefore aimed to elucidate the role of Cand1 in PCa patients’ samples as well as in PCa cell lines. In this context, we not only examined therapy-naive PCa cells, but also included cell lines resistant to the AR-inhibiting agent enzalutamide, as we speculated an involvement of Cand1 in enzalutamide resistance mechanisms.

## 2. Materials and Methods 

### 2.1. Tissue Microarray and Immunohistochemistry

The use of archived tissue samples for this study was approved by the Ethics Committee of the Medical University Innsbruck (UN3174, AM 3174), informed consent of all patients included in the study is available.

To evaluate differences in Cand1 expression between malignant and benign prostate tissue, we constructed a tissue microarray (TMA) of PCa patients who underwent a radical prostatectomy due to biopsy confirmed localized PCa. In addition, punches of paraffin embedded metastatic PCa cell lines (PC3, DU145, PC3-DR and DU145-DR) were included as control. For each selected case, three cancer tissue cores and three benign cores were punched. The TMA was assembled using a manual tissue arrayer (Beecher Instruments, Sun Prairie, WI, USA). Hematoxilin/Eosin (HE) and p63/alpha-methylacyl-CoA racemase (AMACR) immunohistochemistry (IHC) double staining to control the histological diagnosis and Cand1 IHC were performed on a Discovery-XT staining device (Ventana, Tucson, AZ, USA) using the following antibodies: Cand1 (Cell Signaling Technology, 2316 ZA Leiden, The Netherlands), anti-p63 (Sigma Aldrich, Vienna, Austria), anti-AMACR (Dako, Vienna, Austria). Microscope images were taken with a Zeiss Imager Z2 microscope (Zeiss, Vienna, Austria) equipped with a Pixelink PLB622-CU camera (Canimpex Enterprises Ltd, Halifax, NS, Canada). IHC expression analysis was performed by the uro-pathologist G.S. multiplying the percentage of positive cells with the staining intensity (no staining: 0, weak light: 1, medium: 2, strong: 3). 

### 2.2. Cell Lines and Cell Culture

The human PCa cell lines LNCaP, DU145 and PC3 were purchased from American Type Culture Collection (ATCC; Manassas, VA, USA), whereas LAPC-4 cell line was a generous gift from Dr. A. Cato (University of Karlsruhe, Karlsruhe, Germany). The androgen independent cell subline LNCaP abl was established by Culig et al. by cultivating androgen sensitive LNCaP cells in steroid free medium for 87 passages [19]. The enzalutamide-resistant cell lines (EnzaR) of LAPC-4 and LNCaP abl were also generated by our group as described before [20]. The DUCaP cell line as well as the benign prostatic hyperplasia epithelial cell line BPH-1 were a generous gift from Dr. J. Schalken (Radboud University Nijmegen, 6525 XZ Nijmegen, Netherlands), whereas NAF PF179T (hTERT immortalized normal prostate tissue associated fibroblasts), CAF PF179T (hTERT immortalized prostate cancer associated fibroblasts) and EP156T (hTERT immortalized prostate epithelial cells) were established in collaboration with Dr. Varda Rotter (Weizmann Institute, Rehovot, Israel) [21]. The RWPE-1 cell line established at the Michigan State University was provided by Dr. William Watson (University College Dublin, Ireland) [22]. The identity of the used cancer cell lines was confirmed by forensic DNA fingerprinting methods using the AmpFlSTR^®^ SGM Plus^®^ PCR amplification kit (Applied Biosystems, Brunn am Gebirge, Austria).

LNCaP, DU145, PC3, DUCaP and BPH-1 were grown in Roswell Park Memorial Institute (RPMI) 1640 medium without L-Glutamine (Lonza, Basel, Switzerland) containing 10% fetal bovine serum (FBS; Biowest, Nuaillé, France), 1% penicillin/streptomycin (Lonza, Basel, Switzerland) and 1% GlutaMAX (Gibco, Vienna, Austria). LAPC-4 cells were cultivated in the same medium but additionally supplemented with 1 nM dihydrotestosterone (DHT) (Sigma Aldrich, Vienna, Austria). NAF and CAF were cultivated in Minimal Essential Medium (MEM, Gibco, Vienna, Austria) with Earle´s Salts without L-glutamine supplemented with 10% FBS, 1% penicillin/streptomycin, 1% GlutaMAX, 1 mM sodium pyruvate (100 mM, Lonza, Basel, Switzerland) and 1% NEAA (Lonza, Basel, Switzerland). The epithelial cell line RWPE-1 was grown in Keratinocyte Serum Free Medium (K-SFM, Gibco, Vienna, Austria) without FBS but supplemented with 0.05 mg/mL Bovine Pituitary Extract (BPE) and 5 ng/mL epidermal growth factor (EGF). LNCaP abl were grown in RPMI 1640 without L-Glutamine containing 10% charcoal stripped fetal bovine serum (CS-FBS; PAN Biotech, Aidenbach, Germany), 1% penicillin/streptomycin, 1% GlutaMAX, 1 mM sodium pyruvate, 10 mM HEPES and 2.5 g/L D (+) glucose. EnzaR cell lines were treated with the corresponding medium plus 8 µM enzalutamide (MedChemExpress, Stockholm, Sweden) for LAPC-4 EnzaR and 13 µM enzalutamide for LNCaP abl EnzaR cells. The corresponding amount of ethanol was used for vehicle treated control cells.

### 2.3. Cand1 Overexpression

Depending on the corresponding PCa cell line, 1.2 × 10^5^ to 2.0 × 10^5^ cells were seeded in 6-well plates to 50%–60% confluence 24 hours before transfection. For overexpression of Cand1, ViaFect^TM^ transfection reagent (Promega, Mannheim, Germany) was used according to the manufacture´s instruction as well as pCMV6-Entry-Cand1 (SC125317, Origene, Hiddenhausen, Germany) overexpression plasmid and pCMV6-Entry (PS100001, Origene, Hiddenhausen, Germany) control plasmid. Then, 72 hours after transfection, cells were used for immunofluorescence staining.

### 2.4. Cand1 Downregulation

For downregulation of Cand1 in PCa cells, a pool of four targeting siRNAs (On-Targetplus Human Cand1siRNA smart pool, L-015562-01, Dharmacon, Vienna, Austria) was used: GACUUUAGGUUUAUGGCUA (J-015562-09), CGUGCAACAUGUACAACUA (J-015562-10), CAACAAGAACCUACAUACA (J-015562-11), CAUAACAAGCCAUCAUUAA (J-015562-12). A pool of 4 non-targeting siRNAs (ON-Targetplus Non-targeting Control Pool, D-001810-10-20, Dharmacon, Vienna, Austria) served as negative control (siCtrl). Both, siCand1 and siCtrl were resuspended in 1× siRNA Buffer (B-002000-UB-100, Dharmacon, Vienna, Austria). Transfection of targeting or control siRNAs (50 nM) was performed using Lipofectamine2000 transfection reagent (Invitrogen, Lofer, Austria) according to the manufacturer´s instruction. Target gene downregulation was confirmed by qRT-PCR and Western blot analysis. When performing functional experiments (p21 expression levels and SKP Elisa), the cell cycle was synchronized with a thymidine block using 2 mM thymidine (Sigma Aldrich, Vienna, Austria) for 18 hours. 

### 2.5. Western Blot Analysis

Total protein was extracted from cells using RIPA lysis buffer (1% Triton X-100 (Serva, Heidelberg, Germany), 0.5% sodium deoxicholate (Sigma Aldrich, Vienna, Austria), 150 mM sodium chloride (Lactan, Graz, Austria), 50 mM Tris (Sanova, Vienna, Austria)) without sodium dodecyl sulphate (SDS) containing 5 mM sodium fluoride (NaF, Sigma Aldrich, Vienna, Austria), 1 mM phenylmethylsulfonyl fluoride (PMSF, Sigma Aldrich, Vienna, Austria), 1% Triton X-100, 1% Phosphatase Inhibitor Cocktail 2 (Sigma Aldrich, Vienna, Austria) and 0.5% Protease Inhibitor Cocktail Set III (Calbiochem, Vienna, Austria). After addition of lysis buffer to the cell pellets, the samples were shaken at 4 °C for 1 hour. Cell debris were then removed by centrifugation (maximum speed, 4 °C,10 minutes), samples were diluted in lysis buffer and 4× NuPAGE LDS Sample Buffer (Invitrogen, Lofer, Austria) and 50 µg protein per lane were loaded on a 4%–12% Bis-Tris protein (Invitrogen, Lofer, Austria) sodium dodecyl sulfate polyacrylamide gel. Electrophoresis (SDS-PAGE) was performed in a XCell SureLock Mini-Cell Electrophoresis System at 150 V using 1× NuPAGE MOPS running buffer (Invitrogen, Lofer, Austria).

Western blot was performed as previously described by our group [20,23]. Nitrocellulose membranes were incubated with the primary antibodies overnight at 4 °C. The following primary antibodies were used: Cand1 (1:500, Rabbit monoclonal Antibody (RmAb), Cell Signaling Technology, 2316 ZA Leiden, The Netherlands), PARP p85 Fragment (1:500, Rabbit polyclonal Antibody (RpAb), Promega, Mannheim, Germany), p21 (1:500, Mouse monoclonal Antibody (MmAb), Cell Signaling, 2316 ZA Leiden, The Netherlands) and GAPDH (1:50000, Mouse monoclonal Antibody (MmAb), Millipore, Vienna, Austria). Afterwards the membranes were incubated with infrared fluorescent dye labeled secondary antibodies (LiCor Biosciences, Bad Homburg, Germany) for 1 hour at room temperature and scanned using the Odyssey infrared imaging system (LiCor Biosciences, Bad Homburg, Germany). Densitometric analysis was performed using Odyssey application software (LiCor Biosciences, Bad Homburg, Germany).

### 2.6. Bradford Assay

The protein concentration was determined using the Bradford assay. For this, a standard curve was generated using bovine serum albumin (BSA, Sigma Aldrich, Vienna, Austria) with concentrations ranging from 0 to 40 µg/mL. The samples were diluted in phosphate buffered saline (PBS) and 1 mL of 1× Bradford reagent was added to each cuvette. The absorbance was measured at 595 nm using the Spectrophotometer U 2000 (Hitachi, Metrohm Inula GmbH, Vienna, Austria) and the protein concentrations were calculated based on the standard curve.

### 2.7. Caspase-Glo 3/7 Assay

Depending on the cell line, 7.5 × 10^4^ to 3.0 × 10^5^ cells were seeded in 6 well plates, transfected with 50 nM siCand1 or siCtrl and harvested after 96 hours of incubation. Cell lysis and protein extraction were performed as described above for SDS-PAGE samples. The measurement was carried out in black 96-well plates using lysis buffer as blank. 20 µL of PBS and 5 µL of well vortexed sample were incubated with 25 µL of Caspase-Glo 3/7 Substrate (Promega, Mannheim, Germany) on a horizontal shaker for 5 minutes and other 30 minutes at room temperature in the dark. Luminescence was measured using the Chameleon V Multitechnology Plate Reader (HVD Life Sciences, Vienna, Austria) and the results (relative light units, RLU) were normalized to the inserted amount of protein determined via Bradford assay.

### 2.8. Cell Viability Assay (WST-1)

Cell viability upon downregulation of Cand1 was assessed using the Cell Proliferation Reagent WST-1 (Roche Diagnostics GmbH, Vienna, Austria). Depending on the cell line, 3000–5000 cells were grown in five replicates in 96-well plates and transfected the next day as indicated. After 96 hours, WST-1 reagent was added 1:10 to the cells in each well and further incubated at 37 °C. The absorbance was measured after 2 hours at 420–480 nm using the Chameleon V Multitechnology Plate Reader (HVD Life Sciences, Vienna, Austria).

### 2.9. Proliferation Assay (^3^H-Thymidine Incorporation Assay)

Cell proliferation upon downregulation of Cand1 was determined by the ^3^H-thymidine incorporation assay. Thereby, 24 hours before transfection with 50 nM siRNA, depending on the cell line, 3000–5000 cells were seeded in five replicates onto 96-well plates. Then, 72 hours after transfection, 1 µCi of ^3^H-thymidine (Hanke Laboratory Products, Vienna, Austria) was added to each well and the cells were further incubated at 37 °C for 24 hours. After freezing the plate at −20 °C, cell DNA was harvested on 96-well filter plates (PerkinElmer Life Sciences Austria, Vienna, Austria) and 50 µL of scintillation fluid (PerkinElmer Life Sciences Austria, Vienna, Austria) were added. Incorporated radioactivity was quantified using the Chameleon V Multitechnology Plate Reader. 

### 2.10. SKP1 and SKP2 ELISA 

Cell pellets were re-suspended in ice-cold PBS and cells lysis was performed by ultra-sonification. Cell lysates were then centrifuged at 1500× g, 10 minutes at 4 °C and supernatants were used in the ELISA for SKP1 and SKP2, which were performed according to the manual of the manufactory instructions (Abbexa, Cambridge, UK; SKP1: Catalog No.: abx153099, SKP2: Catalog No.: abx153100). The SKP1 and SKP2 concentrations of the samples were interpolated from each standard curve and the obtained results were normalized to the amount of protein determined via Bradford Assay.

### 2.11. Immunofluorescence

Depending on the cell line, 1.2 × 10^5^ to 2.0 × 10^5^ cells were grown on sterile glass coverslips in 6-well plates for 24 hours and transfected with 50 nM siCtrl, 50 nM siCand1 or 4 µg pCMV6-Entry-Cand1 as described above. After 72 hours of incubation, cells were washed with PBS and fixed in 4% paraformaldehyde (PFA) for 10 minutes at room temperature. After a second washing procedure, cells were permeabilized with 0.2% Triton X-100 in PBS with 1% BSA for 5 minutes on ice. Blocking was carried out with 1% BSA in PBS for 30 minutes at room temperature, exchanging the solution three times. The cells on the coverslips were incubated for 1 hour at 37 °C in a wet chamber with 20 µL of primary antibody diluted in blocking solution. The following antibodies were used: Cand1 (1:50, RmAb, Cell Signaling Technology, 2316 ZA Leiden, The Netherlands), Rabbit IgG Isotype Control (1:250, RmAb, Cell Signaling Technology, 2316 ZA Leiden, The Netherlands) and Cytokeratin-8/KRTB (1:100, Chicken polyclonal Antibody (CpAb), Sigma Aldrich, Vienna, Austria) for isotype control. After incubation, cells were rinsed in PBS four times, dried on a paper towel and probed for 1 hour at 37 °C in a wet chamber in the dark with 100 µL of respective secondary antibody (goat anti rabbit IgG (Alexa Fluor 555), goat anti-chicken IgG (Alexa Fluor 488), both Invitrogen, Lofer, Austria) diluted 1:500 in blocking solution. Again, cells were then rinsed four times in PBS and twice in aqua dest., dried on a paper towel and embedded using VECTASHIELD Antifade Mounting Medium containing DAPI (Linaris (VectorLabs), Szabo Scandic, Vienna, Austria), which stains DNA blue. Finally, cells were visualized by fluorescent microscopy using the Axio Imager Z2 microscope (Zeiss, Vienna, Austria) and the TissueFaxs Cell Analysis System (TissueGnostics, Vienna, Austria).

### 2.12. Next Generation Sequencing of Cell Lines

Briefly, 200 ng of genomic DNA per sample was sheared using a Covaris M220 instrument (Covaris, Brighton, United Kingdom) resulting in an average fragment size of 183 base pairs as determined by Agilent Bioanalyzer 2100 (Agilent Technologies Austria, Vienna, Austria), analysis. Whole exome sequencing libraries were constructed using Agilent SureSelectXT Human All ExonV6+COSMIC kit (#5190-9307, Agilent Technoligies, Vienna, Austria) and sequenced paired-end (2×125 base pairs) on a HiSeq 2500 (Illumina, San Diego, U.S.A.) instrument with an average yield of 2 × 98.7 million reads per sample. Mutation calling was performed using the GATK best practices pipeline. Specifically, raw sequencing reads were aligned to the human genome (hg19) using Burrows-Wheeler Aligner and then processed using Picard tools (version 2.1.1) and GATK (version 3.6); mutations were called with the HaplotypeCaller.

### 2.13. Statistical Analyses

Statistical analyses were performed using GraphPad Prism 5.0 and R Platform for Statistical Computing. For cell culture experiments, data are shown as mean with standard error of mean (SEM) from at least three independent experiments. For patient studies, data are presented as boxplots (box denoting for median with interquartile range (IQR) and whiskers depicting median ± 150% IQR) with points coding for single patient samples layered on them. Statistical significance for differences between two independent groups were analyzed with two tailed T test or Mann–Whitney U test for normally and non-normally distributed data, respectively. Statistical significance for more than two independent groups was assessed, dependent on number of factors, with one- or two-way ANOVA. Significances are encoded as follows: *p < 0.05; **p < 0.01; ***p < 0.001.

### 2.14. Survival and Differential Gene Expression

For survival and differential gene expression, published studies available from Gene Expression Omnibus (GEO) were re-analyzed: GSE16560 (*n* = 280), GSE70768 (*n* = 110), GSE70769 (*n* = 91), GSE40272 (*n* = 87) and TCGA (*n* = 419) [24,25,26,27]. For characteristics of the study cohorts, see Supplementary Table S1. In brief, clinical information such as survival or Gleason score as well as normalized whole-genome and *Cand1* gene expression data provided by the study authors were extracted. In each study, patients were stratified according to tumor tissue Cand1 expression into Cand1^lo^ and Cand1^hi^ expressers. The stratification threshold was iteratively calculated with an in-house-written algorithm maximizing differences in survival tested with Mantel–Haenszel test. For analysis of survival differences between Cand1^lo^ and Cand1^hi^ individuals, Kaplan-Meier plots were generated and statistical significance for survival differences assessed with Mantel–Haenszel and Wilcoxon test and univariate Cox proportional hazard modeling (R packages survival and survminer). Identification of differentially expressed genes was performed with two-tailed T tests, p value correction was done with the Benjamini–Hochberg method (False Discovery Rate, p_FDR_). Genes with pFDR < 0.05 and fold-regulation between Cand1^lo^ and Cand1^hi^ samples exceeding 1.25 were deemed significantly regulated. Genes significantly up- and downregulated in at least two out of four analyzed studies were further investigated for Gene Ontology (GO) term enrichment (biological process and molecular function GOs) with DAVID [28,29].

## 3. Results

### 3.1. Cand1 Expression in Patient Tissue Samples

To assess the impact of Cand1 in patients, we generated a TMA of 95 patients undergoing radical prostatectomy at our department for localized PCa. For all patients the mean age was 64.9 years (SD: 6.02). In the published GSE 70769 study no age-related differences in Cand1 expression were observed (Supplementary Figure 1A). 

**Figure 1:**
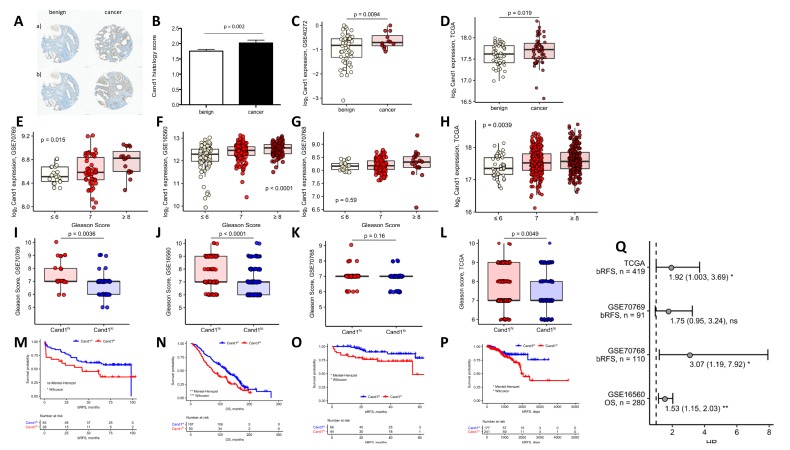
(**A**) Representative benign and malignant tissue cores of patients with low (**a**) and high (**b**) Cand1 expression (scale bar = 100 μm); (**B**) Cand1 expression in 95 radical prostatectomy specimens analyzed by immunohistochemistry in benign vs. malignant prostate tissue. Significance assessed by two-tailed T test; (**C**) log2 Cand1 expression in benign and malignant prostate tissue in the GSE40272 (benign: *n* = 52, malignant: *n* = 12). Significance assessed by unpaired two-tailed T test; (**D**) log2 Cand1 expression in benign and malignant prostate tissue in TCGA cohort (matched benign-malignant tissue samples from *n* = 52 individuals). Significance assessed by paired two-tailed T test; (**E–H**) Comparison of Gleason scores in Cand1 high (Cand1^hi^) and Cand1 low (Cand1^lo^) individuals in the GSE70769 (Cand1^lo^: *n* = 64; Cand1^hi^: *n* = 28) (**E**), GSE16560 (Cand1^lo^: *n* = 187; Cand1^hi^: *n* = 93) (**F**), GSE70768 (Cand1^lo^: *n* = 67; Cand1^hi^: *n* = 44) and (**G**) TCGA studies (Cand1^lo^: *n* = 180, Cand1^hi^: *n* = 258) (**H**). Significance assessed by two-tailed T test; (**I–L**) Differences in log2 Cand1 expression in samples stratified according to the sum Gleason score in the GSE70769 (GS ≤ 6: *n* = 20, GS = 7: *n* = 56, GS ≥ 8, *n* = 15) (**I**), GSE16560 (GS ≤ 6: *n* = 83, GS = 7: *n* = 117, GS ≥ 8, *n* = 81) (**J**), GSE70768 (GS ≤ 6: *n* = 17, GS = 7: *n* = 87, GS ≥ 8: *n* = 18) (**K**), and TCGA studies (GS ≤ 6: *n* = 45, GS = 7: *n* = 246, GS ≥ 8: *n* = 204) (**L**). Significance assessed by one-way ANOVA; (**M–P**) Patients from the investigated transcription studies (GSE70769, Cand1^lo^: *n* = 64; Cand1^hi^: *n* = 28 (**M**), GSE16560, Cand1^lo^: *n* = 187; Cand1^hi^: *n* = 93 (**N**); GSE70768, Cand1^lo^: *n* = 67; Cand1^hi^: *n* = 44 (**O**) and TCGA Cand1^lo^: *n* = 177; Cand1^hi^: *n* = 241 (**P**) were stratified according to Cand1 expression in prostate cancer tissue. The optimal log_2_ Cand1 expression threshold was found with an algorithm iteratively maximizing the difference in survival assessed by Mantel-Haenszel test (GSE16560: 12.56, GSE70768: 8.26, GSE70769: 8.70, GSE40272: −0.76, TCGA: 17.45). Kaplan-Meyer plots are presented together with the risk tables in four different data sets: GSE70769, bRFS: biochemical relapse-free survival (**M**), GSE16560, OS: overall survival (**N**), GSE70768, bRFS: biochemical relapse-free survival (**O**) TCGA, bRFS: biochemical relapse-free survival (**P**). Statistical significance was assessed with Mantel-Haenszel and Wilcoxon tests; (**Q**) Results of univariate Cox proportional hazard modeling of survival (OS: overall survival, bRFS: biochemical relapse-free survival) as a function of log2 Cand1 expression. Points denote for estimated hazard ratio (HR), whiskers indicate 95% confidence intervals (CI). Model estimates in form: HR (2.5% CI, 97.5% CI) and *p* values are presented.

In total, we analyzed 251 benign and 212 cancer areas from the local cohort of 95 patients on Cand1 expression and studied different expression patterns of Cand1 in tumor versus benign samples. We could show that Cand1 is expressed in the nucleus as well as in the cytoplasm of benign and prostate carcinoma cells. Remarkably, Cand1 was significantly higher expressed in cancer cores compared to benign cores (*p* < 0.002) (Figure 1A,B). This different expression pattern was further confirmed in independent published gene expression studies providing information on expression levels in benign and cancer tissue samples GSE40272 (*p* < 0.009, Figure 1C) and TCGA (*p* < 0.019, Figure 1D).

Besides this, we aimed to explore the correlation between PCa aggressiveness measured by Gleason score (GS) and Cand1 expression in the GSE16560, GSE70768, GSE70769 and TCGA studies. Thereby we found that in three (GSE16560, GSE70769, TCGA) out of four published datasets with GS data available, higher GS was associated with higher Cand1 expression (Figure 1E–H). Stratifying the GS in 6 (low grade), 7 (intermediate), and ≥ 8 (high grade), a significant correlation between high Cand1 expression and higher GS was observed in three (GSE16560, GSE70769, TGCA) out of four datasets (Figure 1I–L). Further correlations between Cand1 expression and PSA levels (Supplementary Figure 1B) or histo-pathological features (Supplementary Figure 1C–D) were not observed with different published datasets.

### 3.2. Cand1 Gene Expression-Stratified Survival Analysis of PCa Patients

In the next step, we assessed the impact of *Cand1* gene expression on survival in PCa patients. Out of four published studies analyzed, three displayed an association between relapse-free survival (GSE70768, GSE70769, TGCA) and overall survival (OS) (GSE16560) after primary tumor resection and high *Cand1* mRNA expression (Figure 1 M–Q). 

### 3.3. Cand1 Expression in PCa Cell Lines

In order to define Cand1 protein expression levels in human prostate cells, a cell line screen of various PCa (LNCaP, LNCaP abl, DUCaP, LAPC-4, CAF, PC3, DU145) and non-cancerous cell lines (EP156T, RWPE-1, BPH-1, NAF) was performed using Western blot. Cand1 was detected in all benign and malignant, epithelial and stromal as well as AR positive and negative cell lines (Supplementary Figure 2A).

### 3.4. Cand1 Role in Treatment-Naïve PCa Cells

#### 3.4.1. siRNA Mediated Downregulation of Cand1 in LNCaP and PC3

**Figure 2:**
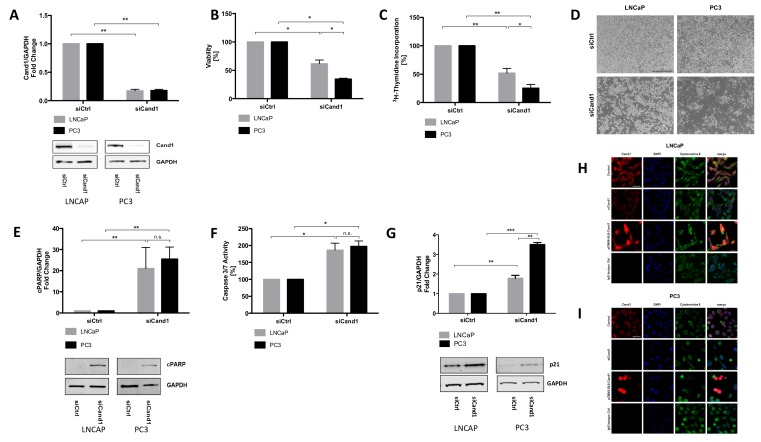
(**A**) Statistical analyses and representative Western blot images of Cand1 levels of LNCaP and PC3 cells (fold change of Cand1 expression normalized to GAPDH as loading control) transfected with 50 nM siCtrl or siCand1 and incubated for 96 h. Statistical analysis of cell viability (**B**) and proliferation (**C**) after Cand1 downregulation compared to siCtrl. Data represent mean + SEM from 4 (**A**) or 7 (**B, C**) independent experiments; *p < 0.05, **p < 0.01. Significance assessed by non-parametric Mann-Whitney U test; (**D**) Representative microscopic pictures (magnification 40×, scale bar 500 µm) of reduced cell number upon Cand1 downregulation in LNCaP and PC3 cells; (**E**) Statistical analyses and representative Western blot images of cPARP levels in LNCaP and PC3 transfected with 50 nM siCtrl or siCand1 and incubated for 96 h. The graph displays fold change of cPARP expression normalized to GAPDH; (**F**) Statistical analysis of caspase 3 and 7 activities in LNCaP and PC3 upon downregulation of Cand1. Statistical analysis of caspase 3/7 activity is shown. The graph displays the percentage of caspase 3 and 7 activities (RFU) normalized to the amount of protein (µg). Caspase-Glo 3/7 assay was carried out 96 h after transfection of LNCaP and PC3 with 50 nM siCtrl or siCand1. Data represent mean + SEM from 5 independent experiments; n.s.: not significant, *p < 0.05. (**G**) Statistical analysis of p21 protein levels after Cand1 downregulation compared to siCtrl. Data represent mean + SEM from 7 (**E, F**) or 4 (**G**) independent experiments; *p < 0.05, **p < 0.01, ***p < 0.001. Significance assessed by non-parametric Mann-Whitney U test; (**H–I**) LNCaP (**H**) and PC3 (**I**) cells were transfected with 50 nM siCtrl (Control), 50 nM siCand1 or 4 µg of pCMV6-Entry-Cand1 overexpression vector and incubated for 72 h. Cand1 was detected using Cand1 RmAb and visualized using goat anti-rabbit secondary antibody (Alexa Fluor 555). Primary antibody anti-KRTB CpAb against Cytokeratin-8 reacted with goat anti-chicken secondary antibody (Alexa Fluor 488). Rabbit mAb IgG served as isotype control. Cell nuclei/DNA were counterstained using DAPI. *n* = 3. Magnification 400×, scale bar: 50 µm.

To examine the biological role of Cand1 in PCa cell lines, we modulated its expression in AR positive LNCaP cells as well as in AR negative PC3 cells. Downregulation of Cand1 was successfully performed using a pool of four specifically targeting siRNAs. A significant decrease of Cand1 on protein level was detected by Western blot analysis after 96 hours of incubation using 50 nM siCand1 (Figure 2A).

#### 3.4.2. Cell Viability and Proliferation Assays 

Furthermore, we aimed to evaluate the impact of Cand1 downregulation on cell viability and proliferation using WST assay and ^3^H-thymidine incorporation assay. Cand1 knockdown significantly reduced the viability of LNCaP cells compared to the control cells (Figure 2B). Moreover, a decrease in proliferation by 50% was observed (Figure 2C). In PC3 cells, transfection with siCand1 also significantly reduced cell viability and proliferation by 66% and 75%, respectively (Figure 2B–C). Interestingly, AR negative PC3 cells were affected to a greater extent by the downregulation of Cand1 compared to AR positive LNCaP cells. In addition to cell proliferation and cell viability assay, a diminished cellular growth upon downregulation of Cand1 was also microscopically observed. After 96 hours following transfection with siCand1, the cell density in the wells was reduced and the number of dead cells increased compared to the control (Figure 2D).

#### 3.4.3. Assessment of Apoptotic Processes 

Next, we aimed to assess whether Cand1 downregulation triggers the programmed cell death of human PCa cells. Determination of cleaved poly (ADP-ribose) polymerase (cPARP) by Western blot as well as measurement of caspase 3/7 activity were performed. The protein level of cPARP upon Cand1 downregulation was quantified using Western blot analysis. In LNCaP cells, a significant, 21-fold increase in cPARP level was detected after transfection with siCand1 (Figure 2E). Also in PC3 cells, cPARP levels significantly increased 25.5-fold after transfection (Figure 2E). There were no differences in cPARP level measurable between AR positive LNCaP and AR negative PC3. In addition to cPARP levels, activity of caspase 3 and 7 was measured using a luminescent assay. Apoptosis significantly increased in LNCaP and PC3 cells after downregulation of Cand1 (Figure 2F). Both, AR positive LNCaP and AR negative PC3 showed a comparable increase of the apoptotic marker. As CRLs target cell cycle regulatory proteins we also found that the cyclin-dependent kinase inhibitor p21 is significantly upregulated upon downregulation of Cand1 on both LNCaP and PC3 cells (Figure 2G).

#### 3.4.4. SKP1/ 2 Alterations upon Cand1 Downregulation

Generally, S-phase kinase-associated protein 1 (SKP1) is an essential component of the SCF ubiquitin ligase complex mediating the ubiquitination of proteins involved in cell cycle progression, signal transduction and transcription. In the SCF complex, SKP1 serves as an adapter that links the F-box protein to CUL1, while SKP2 regulates Myc ubiquitination and stability [11,30]. However, in the present study we were not able to find a consistent correlation pattern between Cand1 and SKP1 and SKP2 mRNA levels in the re-analyzed studies (Supplementary Figure 3A-B). Along this line, knockdown of Cand1 did not affect SKP1 and 2 protein levels in any of the studied prostate cell lines (Supplementary Figure 3C-D).

#### 3.4.5. Cellular Localization of Cand1

To examine and visualize the cellular localization of Cand1, immunofluorescence staining was performed. Thereby, we found Cand1 in both, nucleus and cytoplasm (Figure 2H-I). In both, LNCaP (Figure 2H) and PC3 cells (Figure 2I), Cand1 signal was significantly reduced after transfection with siCand1. Using the overexpression plasmid pCMV6-Entry-Cand1, Cand1 specific staining was increased in a substantial fraction of transfected cells (Figure 2H–I).

### 3.5. Regulatory and Functional Impact of Cand 1 Expression

**Figure 3:**
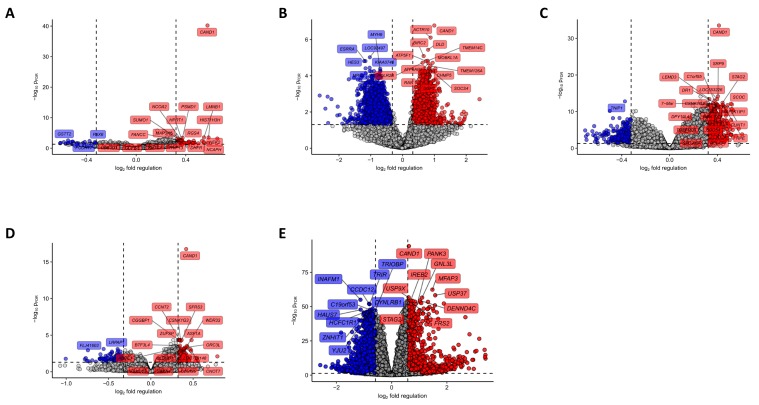
Identification of differentially regulated genes in tumor samples stratified by Cand1 expression. The optimal thresholds for the Cand1 stratification were calculated in Figure 1. Statistical significance for each probe was determined with two-tailed T test. P values were corrected with the Benjamini-Hochberg method (False Discovery Rate, p_FDR_). Significant regulation was defined as p_FDR_ < 0.05 and mean fold-regulation between Cand1^hi^ and Cand1^lo^ samples > 1.25 (p and fold-regulation thresholds plotted as horizontal and vertical lines, respectively). -log10 p_FDR_ and log2 fold regulation for each probe was plotted, probes significantly upregulated in Cand1^hi^ samples are highlighted in red, probes significantly downregulated in Cand1^hi^ samples are highlighted in blue. Dashed horizontal line denotes for p_FDR_ = 0.05, dashed vertical lines denote for 1.25- fold up- and downregulation considered as significance thresholds. Top 20 most significantly regulated probes were labeled with the respective gene symbols. (**A**) GSE16560 (Cand1^lo^: n = 187; Cand1^hi^: n = 93), (**B**) GSE70768 (Cand1^lo^: *n* = 106; Cand1^hi^: *n* = 92), (**C**) GSE70769 (Cand1^lo^: *n* = 65; Cand1^hi^: *n* = 28), (**D**) GSE40272 (Cand1^lo^: *n* = 44; Cand1^hi^: *n* = 43), (**E**) TCGA (Cand1^lo^: *n* = 203; Cand1^hi^: *n* = 296). A list of significantly regulated genes in each study can be found in Supplementary Tables S2 and S3.

To corroborate our findings from *in vitro* experiments, we had a closer look on genes differentially expressed in Cand1^hi^ and Cand1^lo^ cancers. Depending on the study, we were able to detect between 53 and 1854 significantly upregulated (GSE16560: 53 genes, GSE70768: 353, GSE70769: 72, GSE40272: 1854, TCGA: 296 genes) and between 29 and 1880 significantly downregulated genes (GSE16560: 29 genes, GSE70768: 269, GSE70769: 114, GSE40272: 1880, TCGA 200) in Cand1^hi^ tumors (Figure 3 and Supplementary Tables S2 and S3).

**Figure 4:**
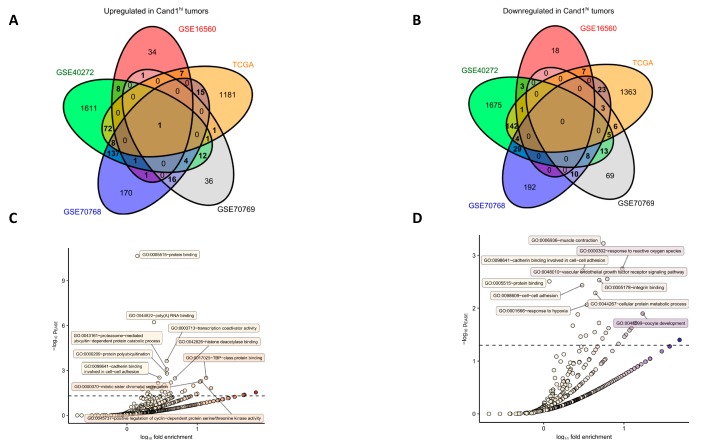
Genes differentially upregulated and downregulated in the GSE16560, GSE70768, GSE70769, GSE40272 and TCGA studies were identified as presented in Figure 3. Genes found to be significantly upregulated or downregulated in at least two of the analyzed data sets were further investigated for GO term enrichment with DAVID. (**A–B**) Venn diagram schematically depicting numbers of significantly upregulated (**A**, *n* = 284) and downregulated (**B**, *n* = 253) genes shared between the analyzed studies. Genes whose counts are presented in bold were subjected to GO term enrichment analysis. Lists of common upregulated and downregulated genes can be found in Supplementary Table S4; (**C–D**) Results of GO term enrichment analysis for common upregulated (**C**) and downregulated (**D**) genes with biological process and molecular function GOs. For each GO term, -log10 *p* value for significant enrichment obtained with Fisher exact test (p_EASE_) was plotted against log10 fold enrichment over whole genome occurrence. Dashed horizontal line denotes for p_EASE_ = 0.05 considered as a significance threshold. Top 10 most significantly enriched GO terms are labeled with identifiers and descriptions. Detailed results of the GO term enrichment analysis can be found in Supplementary Tables S5 and S6.

We further investigated those genes identified in at least two studies (189 common upregulated genes and 75 common downregulated genes; Figure 4A–B and Supplementary Table S4) for their function by GO enrichment analysis. Importantly, several prominent gene groups were found to be significantly enriched in Cand1^hi^ tumors: genes encoding for proteins linked to mRNA turnover, protein polyubiquitination, and proteasomal degradation as well as the positive regulation of cell cycle (Figure 4C, Supplementary Table S5). Notably, this observation fits well to the anti-proliferative effects of the Cand1 knockdown observed in cultured PCa cells and expected distortion of protein ubiquitination in Cand1-deficient cells. Among genes downregulated in the Cand1^hi^ cancers, those linked to cell adhesion and cytoskeleton re-arrangement built a prominently representative group (Figure 4D, Supplementary Table S6).

### 3.6. Cand1 Expression in Enzalutamide-Resistant PCa Cells

Enzalutamide is a second-generation AR inhibitor used for treatment of non-metastatic and metastatic CRPC [31,32,33]. However, up to 40% of patients are primary resistant to treatment or acquire resistance upon a short period of time. We therefore generated enzalutamide-resistant (EnzaR) cell lines to elucidate potential resistance mechanisms to the drug [20].

#### 3.6.1. DNA Sequencing of Parental and EnzaR Cell Lines

We performed DNA sequencing of three EnzaR cell lines (DuCaP EnzaR, LNCaP abl EnzaR and LAPC-4 EnzaR) and compared them to parental cells (DuCaP, LNCaP abl, LAPC-4) to assess potential differences. 

Interestingly, we were able to identify mutations in Cand1 in all three cell lines, which were not present in the parental cell lines (Table 1). The identified mutations were all single nucleotide missense mutations, which have not been reported, neither in the catalogue of somatic mutations in cancer (COSMIC) (http://cancer.sanger.ac.uk, https://academic.oup.com/nar/article/45/D1/D777/2605743), nor in any data set available in cBioPortal (https://www.ncbi.nlm.nih.gov/pubmed/22588877). The level of the mutant alleles was around 15% only in all three cases. The mutation results in an amino acid substitution at position 549 of Cand1 (V549A) in DuCaP EnzaR cells, position 21 (D21N) in LNCaP abl EnzaR cells and 198 (A198D) in LAPC4 EnzaR cells. The Cand1 protein structure was inspected and amino acid exchanges analyzed using COOT 0.8.7.1 [34]. Placing mutations using the mutation function in COOT suggested a moderate impact on the 3D structure only for the exchange of the alanine (small apolar aa) to aspartic acid (charged medium size amino acid) at position 198.

#### 3.6.2. Cand1 Expression in Enzalutamide Resistant PCa Cell Lines

Analyzing Cand1 protein expression levels in enzalutamide resistant vs. parental prostate cells (LNCaP abl, DUCaP, LAPC-4), no statistically significant differences in Cand1 expression among resistant and parental cell lines were observed (Supplementary Figure 2B).

#### 3.6.3. Cand1 Downregulation in LAPC-4 and LNCaP abl EnzaR Cells

**Figure 5:**
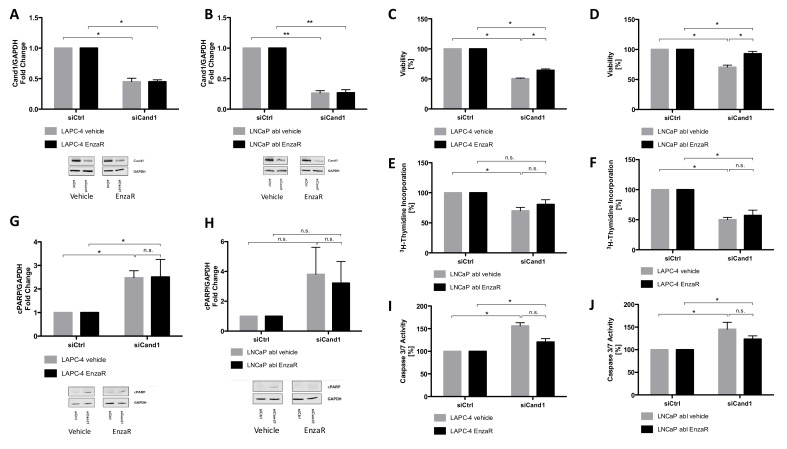
(**A–B**) Statistical analyses and representative Western blot images of LAPC-4 EnzaR (**A**) and LNCaP abl EnzaR (**B**) cells (fold change of Cand1 expression normalized to GAPDH as loading control) transfected with 50 nM siCtrl or siCand1 and incubated for 96 h; (**C–F**) Statistical analysis of cell viability and proliferation after Cand1 downregulation in LAPC-4 EnzaR (**C, E**) and LNCaP abl EnzaR (**D, F**) cells compared to siCtrl. Data represent mean + SEM from 4 independent experiments; *p < 0.05, **p < 0.01. Significance assessed by non-parametric Mann-Whitney U test; (**G–H**) Statistical analyses and representative Western blot images of cPARP levels in LAPC-4 EnzaR (**G**) and LNCaP abl EnzaR (**H**) cells transfected with 50 nM siCtrl or siCand1 and incubated for 96 h. The graphs display fold change of cPARP expression normalized to GAPDH. Data represent mean + SEM from 7 independent experiments; *p < 0.05, **p < 0.01. Significance assessed by non-parametric Mann-Whitney U test; (**H–I**) Statistical analysis of caspase 3 and 7 activities in LAPC-4 EnzaR and LNCaP abl EnzaR cells upon downregulation of Cand1. Statistical analysis of caspase 3/7 activity is shown. The graph displays the percentage of caspase 3 and 7 activities (RFU) normalized to the amount of protein (µg). Caspase-Glo 3/7 assay was carried out 96 h after transfection of LAPC-4 EnzaR (**H**) and LNCaP abl EnzaR (**I**) cells with 50 nM siCtrl or siCand1. Data represent mean + SEM from 5 independent experiments; n.s.: not significant, *p < 0.05. Significance assessed by non-parametric Mann-Whitney U test.

In line with treatment-naïve cell lines, successful knockdown of Cand1 in LAPC-4 EnzaR and LNCaP abl EnzaR cells was confirmed using Western blot analysis. Transfection with 50 nM siCand1 resulted in a significant decrease of Cand1 protein expression (Figure 5A-B).

#### 3.6.4. Cell Viability and Proliferation Assays

Cell viability and proliferation of EnzaR cells upon downregulation of Cand1 was assessed using WST assay and ^3^H-thymidine incorporation assay, respectively. Viability of both cell lines was significantly reduced after transfection with siCand1 compared to siCtrl. EnzaR cells of LAPC-4 (Figure 5C) and of LNCaP abl (Figure 5D) were significantly less affected in their viability than the respective control cells (Figure 5C-D). Proliferation of LAPC-4 EnzaR and their vehicle-treated control cells was reduced after knockdown of Cand1 (Figure 5E), with no significant difference between resistant cells and controls. In contrast, proliferation of LNCaP abl EnzaR cells was not significantly inhibited, while the respective control cells displayed reduced ^3^H-thymidine incorporation (Figure 5F).

To summarize, Cand1 downregulation has a negative impact on the viability of both EnzaR sublines, although to a different extent, while it only significantly inhibits the proliferation of LAPC-4 EnzaR cells.

#### 3.6.5. Assessment of Apoptotic Processes 

As in treatment-naïve cells, the protein level of cPARP upon Cand1 downregulation was assessed using Western blot analysis. Vehicle-treated LAPC-4 cells showed a 2.5-fold increase of cleaved PARP after knockdown of Cand1. Also in LAPC-4 EnzaR cells, the cPARP level rose 2.5-fold. Hence, Cand1 downregulation similarly affects apoptosis of EnzaR and treatment-naive LAPC-4 cells (Figure 5G). The cPARP level in vehicle-treated LNCaP abl cells did not significantly increase after the knockdown of Cand1. In both, vehicle-treated LNCaP abl and LNCaP abl EnzaR cells, we did not observe any statistical increase in apoptosis upon Cand1 downregulation (Figure 5H), nonetheless, a trend towards an upregulated apoptosis was observed, with EnzaR cells being slightly less affected compared to control cells. Analyses of caspase activity of vehicle-treated LAPC-4 cells showed a significant increase of 56% after knockdown of Cand1. In LAPC-4 EnzaR cells, a 20% higher activity of caspase 3 and 7 was measured compared to siCtrl (Figure 5H). The difference in apoptosis between vehicle-treated and EnzaR cells was not significant, however, EnzaR cells tended to be less affected by Cand1 knockdown. In addition, downregulation of Cand1 significantly induced apoptosis in vehicle-treated LNCaP abl cells (+45%) (Figure 5I). EnzaR cells of LNCaP abl showed a 24% increased activity of caspase 3 and 7 after transfection. Hence, Cand1 knockdown triggers apoptosis in both vehicle-treated and EnzaR LNCaP abl cells, with EnzaR cells tending to be affected less than the control cells.

## 4. Discussion

The deregulation of CRL components such as of substrate-receptors or Cand1 have been described in various cancer entities, however there is conflicting evidence concerning the impact of Cand1 in prostatic malignancies. For example, Rulina et al. previously showed that the knockdown of Cand1 induces apoptosis in PCa cell lines, while Murata et al. provided evidence that Cand1 downregulation enhances cell growth in LNCaP PCa cells [35,36]. Therefore, the present study elucidated Cand1 in PCa using not only a preclinical model, but also patient samples to better clarify its clinical impact.

Using a TMA of patients with localized PCa, we found that Cand1 is significantly upregulated in PCa tissue compared to benign controls. These findings are in line with a previous small study [37] as well as two publicly available datasets (GSE40272 and TGCA). In addition, we found that high Cand1 expression was associated with more aggressive PCa in three out of four GEO datasets. The next step was to investigate the potential impact of Cand1 in tumor recurrence and OS. From our patients, who donated samples for IHC analyses, only a small number of patients relapsed yet and fewer are deceased. Therefore, we relied on publicly available datasets to investigate this aspect. Thereby, data revealed that high expression of Cand1 was associated with poor prognosis (GSE 70769, GSE 70768, TCGA) and OS rates (GSE 16560). To summarize our clinical findings: for the first time we could show that in patient samples an upregulated expression of Cand1 yields a more aggressive PCa phenotype.

To understand the biological mechanisms of Cand1 expression in PCa, we modulated its expression in metastatic PCa cell lines and analyzed the resulting functional effects. Briefly, we observed that downregulation of Cand1 caused a significant decrease in cell viability and proliferation in both AR positive LNCaP and AR negative PC3 cells. Further functional experiments and investigations of crucial targets both *in vitro* but also *in vivo* of Cand1 in PCa would be highly interesting to further strengthen the findings of this study.

Our findings contrast the data of Murata et al., showing that Cand1 knockdown using miR-148a promotes proliferation of LNCaP cells [36]. However, according to their published results, Cand1 knockdown in LNCaP cells, which was the only cell line used in this study, was quite moderate, compromising their conclusions. Generally, miRNA´s as well as siRNA´s can result in unspecific effects as the results can be biased by off-target effects. In the present study, this aspect was taken into account using an siRNA pool consisting of four targeting siRNA´s (On-Targetplus Human Cand1 siRNA smart pool, Dharmacon) to reduce off-target effects while still providing guaranteed gene silencing of gene targets. Moreover, one has to consider that the growth promoting effects of miR-148a overexpression described by Murata and colleagues may not only involve Cand1, but conceivably, other signaling pathways like PTEN-inhibited PI-3K/AKT (data not shown), also known to have pleotropic effects in cancer [38,39].

Another argument speaking in favor of the model presented in our work is that low Cand1 expression in primary prostate carcinoma tissue could be linked to better patient survival in four out of five investigated study cohorts. Besides the findings of Rulina that knockdown of Cand1 induces apoptosis in PCa, our findings of reduced cell proliferation due to Cand1 knockdown are also in line with a recent study in liver cancer where Che et al. showed that Cand1 was overexpressed in cancerous issues compared to the corresponding adjacent benign ones and that high expression of Cand1 was associated with poor OS [35,40].

Besides impaired cell viability and proliferation, we were able to show that downregulation of Cand1 resulted in significantly increased apoptosis, reflected by increased caspase 3/7 activity and elevated levels of cPARP in LNCaP and PC3 cells. Consistent with this finding, Rulina et al. demonstrated that Cand1 knockdown induces apoptosis in PCa cell lines [35]. Chua and colleagues hypothesized that Cand1 differentially regulates CRLs depending on their cellular localization, as they found Cand1 in human embryonic kidney cells (HEK293) to be predominantly present in the cytoplasm [41].

In the present study, we provide evidence using immunofluorescence staining and IHC analyses that Cand1 is localized in both, nucleoplasm and cytosol of LNCaP and PC3 cells and PCa tissue areas, respectively. Moreover, our findings are supported by validated data from the Human Protein Atlas 

As CRLs target a variety of cell cycle regulatory proteins including p21, p27 and cyclin E, we measured p21protein levels in PCa cell lines upon Cand1 downregulation and found as expected a significant upregulation of p21. However, in contrast to Dubiel et al., who showed that Cand1 knockdown increases the level of the FBP SKP2 in cervical cancer cells, we did not find any alterations of SKP1 or SKP2 upon Cand1 downregulation [42]. Using bioinformatics tools, we detected genes significantly upregulated in Cand1^hi^ tumors like genes coding for proteins linked to mRNA turnover, protein polyubiquitination, and proteasomal degradation as well as positive regulation of the cell cycle, fitting to anti-proliferative effects of the Cand1 knockdown in cell culture experiments. Cand1 is a central modulator/regulator in protein ubiquitinylation and degradation and unlikely to directly regulate gene transcription. We therefore expect that the different gene expressions associated with Cand1^Hi^/Cand1^Lo^ is an indirect effect.

In this study, we used AR positive (LNCaP) and AR negative (PC3) cell lines to address the issue if the effect of Cand1 is in regard of the androgen receptor. The results obtained in our study clearly demonstrate that Cand1 acts independently of the AR as we observed that in AR negative PC3 cells cell proliferation and cell viability were affected to a greater extent by the downregulation of Cand1 compared to AR positive LNCaP cells. Furthermore, all pathway analyses conducted in our study did not identify the AR-pathway to be involved in Cand1 signalling and therefore we conclude that Cand1 does not influence the AR.

**Figure 6:**
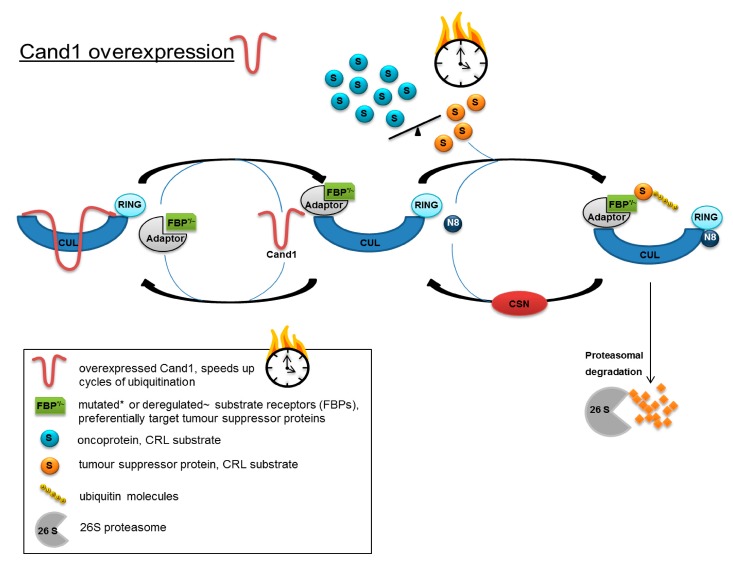
Model for accelerated protein degradation caused by increased Cand1 expression in PCa cells. Increased expression of Cand1 could accelerate the turnover of CRLs and advancing ubiquitination and degradation of tumor suppressor proteins, which are preferentially targeted by deregulated/mutated substrate receptors.

When integrating the results obtained in the present study into the model of CRL control proposed by Schmidt et al. [43], Cand1 may adopt the role of a time-limiting factor in the two regulatory cycles of Cand1 and CSN: intracellular availability of Cand1 combined with a deregulated balance or mutation-dependent change in binding affinity of the different substrate-receptors (FBPs in SCF) might influence the fate of a prostatic cell. In fact, overexpression and deletions of FBPs as well as point mutations have been detected in cancer (reviewed in [30]). High Cand1 expression could accelerate the turnover of CRLs, thus advancing ubiquitination and degradation of tumor suppressor proteins, which are preferentially targeted by the deregulated/mutated FBPs (Figure 6). By lowering the level of Cand1, proteasomal degradation would be slowed down, resulting in an accumulation of tumor suppressor proteins and the induction of apoptosis.

Enzalutamide is an AR inhibitor approved for patients with non-metastatic hormone and metastatic castration-resistant PCa. In addition, it recently has been FDA approved in patients with hormone-naïve metastatic PCa due to a positive phase III study where enzalutamide plus ADT has shown to increase OS and progression free survival (PFS) compared to ADT plus standard androgen ablation [44]. Generally, enzalutamide binds competitively to the AR, inhibits nuclear translocation, and impairs binding of the receptor to the DNA [45]. Response to this drug, however, is only temporary, as the majority of treated patients develop drug resistance after 9–15 months. Thus, the identification of resistance mechanisms to the drug is of important need.

Interestingly, DNA sequencing performed on our recently generated EnzaR PCa cells revealed Cand1 to be commonly mutated compared to corresponding parental cell lines. The identified mutations were all single nucleotide missense mutations not present in the parental cell lines associated with an amino acid substitution, however only with a small impact on the 3D structure and a low level of the mutant alleles of around 15%. To assess a potential functional involvement of Cand1 in the development of drug resistance, we tested the effect of Cand1 knock-down in the EnzaR sublines of LAPC-4 and LNCaP abl. Thereby, we could show that Cand1 knockdown negatively affected cell viability and proliferation and induced apoptosis in both vehicle-treated control cells as well as EnzaR cells. Hence, we conclude that the mutations found in Cand1 of EnzaR cells are not likely to play a major role in conferring resistance to the drug.

## 5. Conclusions

To summarize, we found that Cand1 expression is associated with prostate tumor aggressiveness and recurrence, and that targeting Cand1 reduces the viability and proliferative capacity of prostate tumor cells. Although mutant alleles were detected in EnzaR PCa cell lines, a significant impact on the process of resistance development could not be confirmed in the present study.

## Figures and Tables

**Table 1:** Details of mutations identified in *Cand1* in enzalutamide resistant cell lines.

Cell Line	Gene Symbol	Ensembl Gene ID	Ensembl Transcript ID	Genomic Position	Reference Allele	Alternative Allele	CDS Position	Mutant Allele Frequency	Protein Change	Mutation Classification	Exon
DuCaP EnzaR	*Cand1*	ENSG00000111530	ENST00000545606	12:67699094	T	C	1646	14,97%	V549A	missense_variant	44119
LNCaP abl EnzaR	*Cand1*	ENSG00000111530	ENST00000545606	12:67663558	G	A	61	14,57%	D21N	missense_variant	43845
LAPC-4 EnzaR	*Cand1*	ENSG00000111530	ENST00000545606	12:67691288	C	A	593	16,22%	A198D	missense_variant	43966

## Supplementary Materials

The following are available online at https://www.mdpi.com/2072-6694/12/2/428/s1, Figure S1: (A) Comparison of age at diagnosis between Cand1 high (Cand1hi) and Cand1 low (Cand1lo) individuals in the GSE16560 (Cand1lo: n = 187; Cand1hi: n = 93), GSE70768 (Cand1lo: n = 67; Cand1hi: n = 44), GSE40272 (Cand1lo: n = 44; Cand1hi: n = 42) and TCGA cohort (Cand1lo: n = 180; Cand1hi: n = 258). Significance assessed by two-tailed T test; (B) Comparison of PSA levels between Cand1hi and Cand1lo patients in the GSE70768 (Cand1lo: n = 67; Cand1hi: n = 44), GSE70769 (Cand1lo: n = 64; Cand1hi: n = 28) and TCGA studies (Cand1lo: n = 180; Cand1hi: n = 258). Significance assessed by two-tailed T test; (C) Comparison of probability of developing extracapsular expansion (ECS) between Cand1hi and Cand1lo individuals in the GSE70768 (Cand1lo: n = 67; Cand1hi: n = 44) and GSE70769 (Cand1lo: n = 64; Cand1hi: n = 28) studies. Significance assessed by Chi square test; (D) Comparison of probability of developing positive surgery margins between Cand1hi and Cand1lo individuals in the GSE70768 (Cand1lo: n = 67; Cand1hi: n = 44) and GSE70769 (Cand1lo: n = 64; Cand1hi: n = 28) studies. Significance assessed by Chi square test, Figure S2: (A) Cand1 expression in human prostate cell lines. Bar chart and representative Western blot images of Cand1 protein expression are shown. The graph displays expression of Cand1 normalized to GAPDH as loading control. Data represent mean + SEM from 3 independent experiments. Significance assessed by non-parametric Mann-Whitney U test; (B) Cand1 expression in parental and enzalutamide resistant (EnzaR) prostate cancer cells. Bar chart and representative Western blot images of Cand1 protein expression are shown. The graph displays expression of Cand1 normalized to GAPDH as loading control. Data represent mean + SEM from 3 independent experiments. Significance assessed by non-parametric Mann-Whitney U test, Figure S3: Correlation of tumor tissue Cand1 and SKP1 (A) and SKP2 (B) mRNA levels. For each re-analyzed study, log2 Cand1 expression was modeled as a function of log2 Skp1 (A) or log2 Skp2 (B) expression using Pearson’s linear regression. For GEO microarray studies signals from multiple probes mapped to Skp1/2 transcripts were analyzed separately. Points denote estimated regression line slopes; whiskers indicate 95% confidence intervals (CI). For each study and probe, points were labeled with estimated regression slopes with 95% confidence interval (CI) and p-values (slope ≠ 0, two-tailed T test). Protein levels measured by ELISA of SKP1 (C) and SKP2 (D) upon Cand1 downregulation in PC3 and LNCaP cell lines, Figure S4–9: Whole blots for Western blotting experiments, Table S1-S6: Raw data of bioinformatic analyses.
