# Supplementary material for: The Impact of Cand1 in Prostate Cancer
Source: Cancers (Basel). 2020 Feb 12;12(2):428. doi: 10.3390/cancers12020428 (PMC7072594; doi:10.3390/cancers12020428)
Supplement: Supplementary file 1 [file cancers-12-00428-s001.zip › Supplementary material revision/Supplementary_Table_S5.pdf]

GO term

GO:0018342~protein prenylation
GO:0008318~protein prenyltransferase activity
GO:0061684~chaperone-mediated autophagy
GO:0090168~Golgi reassembly
GO:0006616~SRP-dependent cotranslational protein targeting to membrane, translocation
GO:0007253~cytoplasmic sequestering of NF-kappaB
GO:0008312~7S RNA binding
GO:0051683~establishment of Golgi localization
GO:0043312~neutrophil degranulation
GO:1903071~positive regulation of ER-associated ubiquitin-dependent protein catabolic process
GO:1901409~positive regulation of phosphorylation of RNA polymerase II C-terminal domain
GO:0045047~protein targeting to ER
GO:0045899~positive regulation of RNA polymerase II transcriptional preinitiation complex assembly
GO:0033673~negative regulation of kinase activity
GO:1904903~ESCRT III complex disassembly
GO:0048280~vesicle fusion with Golgi apparatus
GO:0001893~maternal placenta development
GO:0017025~TBP-class protein binding
GO:0090161~Golgi ribbon formation
GO:0045737~positive regulation of cyclin-dependent protein serine/threonine kinase activity
GO:0008334~histone mRNA metabolic process
GO:0060394~negative regulation of pathway-restricted SMAD protein phosphorylation
GO:0045717~negative regulation of fatty acid biosynthetic process
GO:0003724~RNA helicase activity
GO:0043024~ribosomal small subunit binding
GO:0006312~mitotic recombination
GO:1901673~regulation of mitotic spindle assembly
GO:0016538~cyclin-dependent protein serine/threonine kinase regulator activity
GO:0006839~mitochondrial transport
GO:0006301~postreplication repair
GO:0010633~negative regulation of epithelial cell migration
GO:0032781~positive regulation of ATPase activity
GO:0046328~regulation of JNK cascade
GO:0031396~regulation of protein ubiquitination
GO:0001671~ATPase activator activity
GO:0070979~protein K11-linked ubiquitination
GO:0000920~cell separation after cytokinesis
GO:0010971~positive regulation of G2/M transition of mitotic cell cycle
GO:0061631~ubiquitin conjugating enzyme activity
GO:0010824~regulation of centrosome duplication
GO:0006099~tricarboxylic acid cycle
GO:1990841~promoter-specific chromatin binding
GO:0005086~ARF guanyl-nucleotide exchange factor activity
GO:0001205~transcriptional activator activity, RNA polymerase II distal enhancer sequence-specific binding
GO:0097352~autophagosome maturation
GO:0051020~GTPase binding
GO:0003785~actin monomer binding
GO:0006506~GPI anchor biosynthetic process
GO:0045930~negative regulation of mitotic cell cycle
GO:0006997~nucleus organization
GO:0050999~regulation of nitric-oxide synthase activity

GO:0072332~intrinsic apoptotic signaling pathway by p53 class mediator
GO:0006891~intra-Golgi vesicle-mediated transport
GO:0043001~Golgi to plasma membrane protein transport
GO:0000387~spliceosomal snRNP assembly
GO:0022400~regulation of rhodopsin mediated signaling pathway
GO:0043021~ribonucleoprotein complex binding
GO:0005080~protein kinase C binding
GO:0000149~SNARE binding
GO:0019058~viral life cycle
GO:0036258~multivesicular body assembly
GO:0070936~protein K48-linked ubiquitination
GO:0002479~antigen processing and presentation of exogenous peptide antigen via MHC class I, TAP-dependent
GO:0006890~retrograde vesicle-mediated transport, Golgi to ER
GO:0038061~NIK/NF-kappaB signaling
GO:0006521~regulation of cellular amino acid metabolic process
GO:0048487~beta-tubulin binding
GO:0040008~regulation of growth
GO:0051436~negative regulation of ubiquitin-protein ligase activity involved in mitotic cell cycle
GO:0051287~NAD binding
GO:0007080~mitotic metaphase plate congression
GO:0005484~SNAP receptor activity
GO:0051437~positive regulation of ubiquitin-protein ligase activity involved in regulation of mitotic cell cycle transition
GO:0038128~ERBB2 signaling pathway
GO:0006695~cholesterol biosynthetic process
GO:0052689~carboxylic ester hydrolase activity
GO:0050681~androgen receptor binding
GO:0008219~cell death
GO:0008333~endosome to lysosome transport
GO:0000079~regulation of cyclin-dependent protein serine/threonine kinase activity
GO:0051087~chaperone binding
GO:0003743~translation initiation factor activity
GO:0031145~anaphase-promoting complex-dependent catabolic process
GO:0008654~phospholipid biosynthetic process
GO:0018279~protein N-linked glycosylation via asparagine
GO:0043161~proteasome-mediated ubiquitin-dependent protein catabolic process
GO:0031072~heat shock protein binding
GO:0006813~potassium ion transport
GO:0030163~protein catabolic process
GO:0044255~cellular lipid metabolic process
GO:0000045~autophagosome assembly
GO:0030971~receptor tyrosine kinase binding
GO:0006986~response to unfolded protein
GO:0004004~ATP-dependent RNA helicase activity
GO:0032436~positive regulation of proteasomal ubiquitin-dependent protein catabolic process
GO:0006368~transcription elongation from RNA polymerase II promoter
GO:0034613~cellular protein localization
GO:0016197~endosomal transport
GO:0030968~endoplasmic reticulum unfolded protein response
GO:0006413~translational initiation
GO:0000209~protein polyubiquitination
GO:0043154~negative regulation of cysteine-type endopeptidase activity involved in apoptotic process
GO:0042795~snRNA transcription from RNA polymerase II promoter
GO:0006614~SRP-dependent cotranslational protein targeting to membrane

GO:0010467~gene expression
GO:0003729~mRNA binding
GO:0042826~histone deacetylase binding
GO:0051865~protein autoubiquitination
GO:0042542~response to hydrogen peroxide
GO:0043488~regulation of mRNA stability
GO:0019003~GDP binding
GO:0006464~cellular protein modification process
GO:0045727~positive regulation of translation
GO:0006888~ER to Golgi vesicle-mediated transport
GO:0046718~viral entry into host cell
GO:0098609~cell-cell adhesion
GO:0050821~protein stabilization
GO:0016049~cell growth
GO:0006749~glutathione metabolic process
GO:0048306~calcium-dependent protein binding
GO:0098641~cadherin binding involved in cell-cell adhesion
GO:0016569~covalent chromatin modification
GO:0016887~ATPase activity
GO:0051289~protein homotetramerization
GO:0030433~ER-associated ubiquitin-dependent protein catabolic process
GO:0016192~vesicle-mediated transport
GO:0006950~response to stress
GO:0019903~protein phosphatase binding
GO:0060071~Wnt signaling pathway, planar cell polarity pathway
GO:0006977~DNA damage response, signal transduction by p53 class mediator resulting in cell cycle arrest
GO:0050660~flavin adenine dinucleotide binding
GO:0016740~transferase activity
GO:0047485~protein N-terminus binding
GO:0032981~mitochondrial respiratory chain complex I assembly
GO:0072659~protein localization to plasma membrane
GO:0030512~negative regulation of transforming growth factor beta receptor signaling pathway
GO:0034599~cellular response to oxidative stress
GO:0031398~positive regulation of protein ubiquitination
GO:0006396~RNA processing
GO:0015031~protein transport
GO:0006006~glucose metabolic process
GO:0001618~virus receptor activity
GO:0008203~cholesterol metabolic process
GO:0003713~transcription coactivator activity
GO:0035690~cellular response to drug
GO:0006810~transport
GO:0033138~positive regulation of peptidyl-serine phosphorylation
GO:0098869~cellular oxidant detoxification
GO:0002223~stimulatory C-type lectin receptor signaling pathway
GO:0051082~unfolded protein binding
GO:0051117~ATPase binding
GO:0048010~vascular endothelial growth factor receptor signaling pathway
GO:0019900~kinase binding
GO:0044822~poly(A) RNA binding
GO:0000398~mRNA splicing, via spliceosome
GO:0007030~Golgi organization
GO:0019905~syntaxin binding

GO:0043086~negative regulation of catalytic activity
GO:1900034~regulation of cellular response to heat
GO:0006367~transcription initiation from RNA polymerase II promoter
GO:0016236~macroautophagy
GO:0060021~palate development
GO:0045454~cell redox homeostasis
GO:0016925~protein sumoylation
GO:0003690~double-stranded DNA binding
GO:0033209~tumor necrosis factor-mediated signaling pathway
GO:0000287~magnesium ion binding
GO:0090263~positive regulation of canonical Wnt signaling pathway
GO:0035264~multicellular organism growth
GO:0008543~fibroblast growth factor receptor signaling pathway
GO:0051726~regulation of cell cycle
GO:0043066~negative regulation of apoptotic process
GO:0006919~activation of cysteine-type endopeptidase activity involved in apoptotic process
GO:0030307~positive regulation of cell growth
GO:0030036~actin cytoskeleton organization
GO:0007049~cell cycle
GO:0009055~electron carrier activity
GO:0008022~protein C-terminus binding
GO:0006914~autophagy
GO:0006457~protein folding
GO:0003697~single-stranded DNA binding
GO:0061630~ubiquitin protein ligase activity
GO:0006511~ubiquitin-dependent protein catabolic process
GO:0004842~ubiquitin-protein transferase activity
GO:0010629~negative regulation of gene expression
GO:0006897~endocytosis
GO:0030336~negative regulation of cell migration
GO:0071456~cellular response to hypoxia
GO:0000166~nucleotide binding
GO:0042802~identical protein binding
GO:0003714~transcription corepressor activity
GO:0050852~T cell receptor signaling pathway
GO:0019904~protein domain specific binding
GO:0016567~protein ubiquitination
GO:0007062~sister chromatid cohesion
GO:0002576~platelet degranulation
GO:0044212~transcription regulatory region DNA binding
GO:0001649~osteoblast differentiation
GO:0006629~lipid metabolic process
GO:0010628~positive regulation of gene expression
GO:0001764~neuron migration
GO:0004872~receptor activity
GO:0003723~RNA binding
GO:0090090~negative regulation of canonical Wnt signaling pathway
GO:0006979~response to oxidative stress
GO:0008134~transcription factor binding
GO:0006357~regulation of transcription from RNA polymerase II promoter
GO:0031625~ubiquitin protein ligase binding
GO:0008152~metabolic process
GO:0019083~viral transcription

GO:0003924~GTPase activity
GO:0016477~cell migration
GO:0005975~carbohydrate metabolic process
GO:0006281~DNA repair
GO:0042393~histone binding
GO:0038095~Fc-epsilon receptor signaling pathway
GO:0000184~nuclear-transcribed mRNA catabolic process, nonsense-mediated decay
GO:0003824~catalytic activity
GO:0050900~leukocyte migration
GO:0030335~positive regulation of cell migration
GO:0016787~hydrolase activity
GO:0006412~translation
GO:0006366~transcription from RNA polymerase II promoter
GO:0000165~MAPK cascade
GO:0019221~cytokine-mediated signaling pathway
GO:0045944~positive regulation of transcription from RNA polymerase II promoter
GO:0007050~cell cycle arrest
GO:0042803~protein homodimerization activity
GO:0003735~structural constituent of ribosome
GO:0000122~negative regulation of transcription from RNA polymerase II promoter
GO:0008289~lipid binding
GO:0005515~protein binding
GO:0005525~GTP binding
GO:0016032~viral process
GO:0003682~chromatin binding
GO:0042787~protein ubiquitination involved in ubiquitin-dependent protein catabolic process
GO:0008284~positive regulation of cell proliferation
GO:0016301~kinase activity
GO:0006886~intracellular protein transport
GO:0019899~enzyme binding
GO:0007264~small GTPase mediated signal transduction
GO:0007067~mitotic nuclear division
GO:0008380~RNA splicing
GO:0045892~negative regulation of transcription, DNA-templated
GO:0005524~ATP binding
GO:0005125~cytokine activity
GO:0051301~cell division
GO:0006397~mRNA processing
GO:0046982~protein heterodimerization activity
GO:0019901~protein kinase binding
GO:0004674~protein serine/threonine kinase activity
GO:0007507~heart development
GO:0005516~calmodulin binding
GO:0016055~Wnt signaling pathway
GO:0006915~apoptotic process
GO:0007420~brain development
GO:0016491~oxidoreductase activity
GO:0005215~transporter activity
GO:0030198~extracellular matrix organization
GO:0055114~oxidation-reduction process
GO:0006508~proteolysis
GO:0008017~microtubule binding
GO:0042493~response to drug

GO:0034220~ion transmembrane transport
GO:0003676~nucleic acid binding
GO:0006364~rRNA processing
GO:0001525~angiogenesis
GO:0001077~transcriptional activator activity, RNA polymerase II core promoter proximal region sequence-specific bi
GO:0000978~RNA polymerase II core promoter proximal region sequence-specific DNA binding
GO:0007283~spermatogenesis
GO:0045893~positive regulation of transcription, DNA-templated
GO:0016874~ligase activity
GO:0006351~transcription, DNA-templated
GO:0007165~signal transduction
GO:0006468~protein phosphorylation
GO:0003677~DNA binding
GO:0046872~metal ion binding
GO:0005102~receptor binding
GO:0008283~cell proliferation
GO:0008285~negative regulation of cell proliferation
GO:0008270~zinc ion binding
GO:0006355~regulation of transcription, DNA-templated
GO:0007275~multicellular organism development
GO:0043547~positive regulation of GTPase activity
GO:0003700~transcription factor activity, sequence-specific DNA binding

Fold enrichment	p_{EASE}	p_{FDR}
106,278481	0,018612516	0,739026246
73,07792208	0,026946221	0,623389723
70,85232068	0,027789278	0,779376485
53,13924051	0,036880771	0,800531507
35,42616034	0,054811094	0,844362476
35,42616034	0,054811094	0,844362476
31,31910946	0,061755544	0,753990346
30,36528029	0,063651476	0,857128746
30,36528029	0,063651476	0,857128746
26,56962025	0,072409696	0,866728244
26,56962025	0,072409696	0,866728244
23,61744023	0,081086512	0,881634703
23,61744023	0,081086512	0,881634703
23,61744023	0,081086512	0,881634703
21,2556962	0,089682676	0,880155928
21,2556962	0,089682676	0,880155928
21,2556962	0,089682676	0,880155928
20,87940631	8,61E-04	0,078861042
19,32336018	0,098198932	0,89709502
17,71308017	0,001404891	0,260337274
17,71308017	0,10663602	0,910965158
17,71308017	0,10663602	0,910965158
17,71308017	0,10663602	0,910965158
16,86413586	0,111663133	0,831748535
16,86413586	0,111663133	0,831748535
16,35053554	0,11499467	0,922420742
15,18264014	0,123275607	0,918606526
14,61558442	0,127705714	0,830714656
14,17046414	0,131479548	0,919518996
14,17046414	0,131479548	0,919518996
14,17046414	0,131479548	0,919518996
13,71335239	0,002974333	0,346859731
13,28481013	0,139607206	0,920316372
13,28481013	0,139607206	0,920316372
12,17965368	0,151231464	0,869830248
11,80872011	0,026164964	0,780535332
11,80872011	0,155636482	0,935343974
11,80872011	0,155636482	0,935343974
11,33967756	0,028235831	0,559200369
11,18720853	0,163539491	0,941420691
10,99432562	0,02990052	0,764725091
10,96168831	0,166564	0,885958991
8,769350649	0,203702068	0,926157783
8,769350649	0,203702068	0,926157783
8,502278481	0,209437535	0,969085693
8,432067932	0,210929974	0,926163516
8,432067932	0,210929974	0,926163516
8,17526777	0,21684007	0,969656787
8,17526777	0,21684007	0,969656787
8,17526777	0,21684007	0,969656787
8,17526777	0,21684007	0,969656787

7,872480075	0,224173728	0,971850273
7,872480075	0,224173728	0,971850273
7,872480075	0,224173728	0,971850273
7,591320072	0,231439146	0,970665805
7,329550415	0,238636954	0,969544338
7,307792208	0,239195593	0,919712514
7,14892716	0,06518869	0,747691263
7,14892716	0,06518869	0,747691263
6,856676194	0,252832236	0,973332524
6,856676194	0,252832236	0,973332524
6,783732831	0,07145047	0,879987874
6,747840064	0,0211565	0,756176677
6,480395184	0,007313684	0,544916821
6,441120061	0,023892766	0,772831191
6,251675354	0,082289108	0,878167982
6,08982684	0,27971869	0,915371596
6,015763076	0,087886133	0,88835587
5,987520057	0,028858585	0,771892774
5,769309638	0,292744016	0,921133005
5,744782757	0,293859303	0,985074628
5,621378621	0,299168653	0,91623547
5,593604264	0,034328262	0,793493786
5,593604264	0,300476079	0,985006649
5,593604264	0,300476079	0,985006649
5,480844156	0,305535308	0,911531329
5,480844156	0,305535308	0,911531329
5,450178513	0,307031245	0,984942108
5,450178513	0,307031245	0,984942108
5,450178513	0,307031245	0,984942108
5,413179413	0,037287938	0,627688254
5,390994252	0,105723623	0,830589562
5,381188912	0,037849499	0,793292
5,313924051	0,313525372	0,984880757
5,313924051	0,313525372	0,984880757
5,235393153	1,25E-04	0,101849013
5,219851577	0,318096744	0,912256331
5,184316147	0,041548173	0,794651434
5,184316147	0,319959022	0,985615793
5,184316147	0,319959022	0,985615793
5,184316147	0,319959022	0,985615793
5,09845968	0,324292551	0,912593594
5,060880048	0,326332757	0,985543031
5,059240759	0,117450955	0,817604212
4,981803797	0,120483326	0,922825318
4,943185163	0,046752019	0,819444501
4,943185163	0,332647131	0,986213002
4,830840046	0,126694496	0,920086219
4,723488045	0,345099986	0,987418375
4,654532015	0,009326632	0,590701766
4,620803522	0,001743331	0,258749513
4,620803522	0,136149341	0,922904661
4,554792043	0,139335352	0,923835809
4,522488554	0,058070197	0,84009804

4,428270042	0,36334763	0,989515026
4,384675325	0,027045976	0,581591641
4,298701299	0,065558649	0,725510507
4,251139241	0,375231142	0,990331249
4,167783569	0,381089983	0,989683898
4,127319651	0,072193095	0,874307213
4,05988456	0,38886317	0,94682082
4,048704039	0,075522404	0,870190593
4,010508717	0,392644384	0,990918087
3,985443038	0,017288286	0,743410794
3,985443038	0,171995173	0,944518723
3,921715167	0,001030723	0,357512712
3,907297096	0,038859901	0,786852045
3,795660036	0,409575298	0,992258803
3,795660036	0,409575298	0,992258803
3,779892521	0,4107909	0,954383317
3,779892521	0,001328848	0,090696009
3,762070124	0,089497641	0,886299821
3,593996168	0,025679315	0,654543915
3,542616034	0,431422458	0,993899522
3,542616034	0,431422458	0,993899522
3,496002665	0,054641558	0,854630894
3,484540361	0,436757545	0,99376537
3,479901051	0,437104405	0,962636521
3,465602642	0,212587281	0,969058813
3,428338097	0,442042887	0,993633896
3,425527597	0,442225289	0,96213998
3,425527597	0,216228357	0,924283074
3,39021288	0,219556783	0,913251379
3,373920032	0,447278946	0,99350505
3,373920032	0,447278946	0,99350505
3,321202532	0,452466178	0,993378783
3,321202532	0,452466178	0,993378783
3,321202532	0,452466178	0,993378783
3,28696333	0,229776879	0,973027614
3,228713347	0,001237598	0,298245969
3,172491971	0,467739417	0,994218449
3,131910946	0,471993026	0,968135963
3,125837677	0,472735824	0,994376556
3,094024927	0,025610542	0,70965156
3,080535682	0,477685625	0,994527528
3,053979339	0,005507456	0,491821479
3,036528029	0,482589252	0,994409655
3,036528029	0,482589252	0,994409655
3,036528029	0,257448036	0,973969618
2,989551358	0,263106163	0,917489578
2,962618463	0,490955823	0,970137342
2,952180028	0,492259693	0,99469495
2,923116883	0,495589831	0,969655878
2,912760402	2,90E-07	4,14E-05
2,872391379	0,057615621	0,848286015
2,872391379	0,501750521	0,994716892
2,847191769	0,504732486	0,970566255

2,834092827	0,50642962	0,994846319
2,834092827	0,50642962	0,994846319
2,796802132	0,171032954	0,946397956
2,796802132	0,511065054	0,994970445
2,796802132	0,511065054	0,994970445
2,760480026	0,515657228	0,995089557
2,725089257	0,299008792	0,98549866
2,706589707	0,522526791	0,970511211
2,70199528	0,302461666	0,984645513
2,686688312	0,114561422	0,824464205
2,656962025	0,309358587	0,984680428
2,656962025	0,529178187	0,995622074
2,592158073	0,537983117	0,995810914
2,571253573	0,323110365	0,985569038
2,569369871	0,010561873	0,597889479
2,560927253	0,54232403	0,99590006
2,530440024	0,546624415	0,99598592
2,452580331	0,343605735	0,987784275
2,448812927	0,146612151	0,926900147
2,435930736	0,56027543	0,9787654
2,409162266	0,227178807	0,914292265
2,39725897	0,353781627	0,988434617
2,361744023	0,237330724	0,970462668
2,357352325	0,572187279	0,980092855
2,344746163	0,242167874	0,916115099
2,335790791	0,243260175	0,970311326
2,332274109	0,07920471	0,771221495
2,327266008	0,367264305	0,989604483
2,293780166	0,375822831	0,989901617
2,237441706	0,59135889	0,997954791
2,214135021	0,595201296	0,997985862
2,204937304	0,097162255	0,820857512
2,195264682	0,008202258	0,375687273
2,159938584	0,278547815	0,925262635
2,154293534	0,405872839	0,992216415
2,108016983	0,293178106	0,916330639
2,07228236	0,120847005	0,918855896
2,063659825	0,621111342	0,998579425
2,063659825	0,621111342	0,998579425
2,058533016	0,304462857	0,915612812
2,043816943	0,624675697	0,998596557
2,030799	0,434531252	0,993874353
2,028215287	0,230218336	0,971657744
2,024352019	0,628206732	0,998613227
2,020587707	0,314499307	0,914088493
2,003964956	0,061321196	0,778696249
1,956045663	0,451850135	0,993636699
1,932336018	0,645372846	0,998874933
1,929874703	0,257122383	0,923917805
1,927954304	0,120143809	0,92681698
1,909701796	0,263090649	0,923313603
1,897830018	0,467032868	0,994437391
1,897830018	0,652016668	0,998897103

1,873792874	0,356349451	0,931511797
1,853694436	0,479382908	0,99440376
1,832387604	0,490065133	0,994768639
1,808995421	0,37667443	0,989505548
1,796998084	0,672019156	0,990801529
1,791210354	0,49442681	0,994620101
1,786192958	0,674310887	0,999155963
1,749205582	0,510461044	0,970465456
1,742270181	0,683425543	0,999216096
1,732801321	0,521015389	0,995262399
1,721731148	0,519871414	0,971463922
1,68029219	0,422407096	0,993329392
1,657364226	0,205235583	0,968498908
1,622572229	0,444646312	0,993567087
1,622572229	0,709276338	0,999484889
1,516716345	0,129469801	0,920432665
1,507496185	0,735545645	0,999694548
1,501601139	0,217841199	0,918701227
1,481309231	0,600288978	0,983392549
1,476090014	0,232366716	0,969518936
1,451879247	0,748671073	0,996455442
1,434939278	8,66E-09	2,48E-06
1,427303166	0,460773779	0,966523464
1,421785699	0,532073409	0,995617007
1,401750423	0,474669005	0,966937898
1,38926119	0,763972632	0,99983734
1,368392459	0,441810321	0,993931954
1,364525516	0,644994466	0,988753462
1,35099764	0,650460156	0,998921728
1,316719317	0,584141048	0,981373294
1,296079037	0,672762374	0,999174558
1,285626786	0,677082941	0,999157543
1,280463627	0,791350232	0,999911922
1,277897567	0,501423812	0,994931265
1,246479607	0,279644597	0,9207726
1,245646399	0,800280513	0,998479539
1,214611212	0,63892489	0,998797337
1,187469062	0,815570085	0,999954737
1,178676163	0,611417435	0,984378362
1,166137054	0,666045833	0,990736337
1,166137054	0,666045833	0,990736337
1,161513454	0,822444257	0,999961225
1,159967017	0,822804104	0,998965594
1,136668246	0,829063783	0,999966714
1,124640011	0,61535695	0,998505686
1,118720853	0,833866967	0,999969748
1,096168831	0,839878372	0,99915801
1,085315674	0,842802296	0,999137456
1,084474296	0,843074668	0,999976639
1,077146767	0,653273941	0,99886619
1,06278481	0,691601655	0,999297799
1,054008492	0,851259574	0,999231366
1,048800799	0,780232759	0,999887338

1,01217601	0,862634437	0,999988287
1,001575582	0,675499935	0,990580655
0,993256832	0,867763837	0,999989029
0,953170233	0,878619643	0,999992104
0,928956637	0,885122777	0,999676823
0,926339857	0,835382115	0,99914826
0,828144008	0,878405569	0,999992615
0,82546393	0,864199977	0,999988169
0,811976912	0,916103883	0,999886833
0,761073521	0,931481315	0,999999566
0,732323728	0,923620391	0,999999344
0,699200533	0,929485493	0,999999537
0,65482009	0,972553883	0,999997384
0,635767326	0,984991551	0,999999636
0,621058828	0,961152936	0,999992182
0,580756727	0,969074769	0,999999994
0,536760005	0,976822155	0,999999998
0,468848944	0,994730883	0,999999989
0,423983302	0,998837616	1
0,407978814	0,993069737	1
0,376207012	0,995479106	1
0,22813087	0,999877764	1

Gene Symbols
FNTA, PTAR1
FNTA, PTAR1
LAMP2, HSP90AA1
YWHAZ, PDCD10
SRP54, SRP9
PSMD10, G3BP2
SRP54, SRP9
YWHAZ, PDCD10
VAMP7, STXBP3
NFE2L2, UBQLN1
CCNT2, CCNC
SRP54, SPCS1
PSMC6, CAND1
CDKN1B, PRDX3
CHMP5, VPS4B
VAMP7, SEC22C
TMED2, PRDX3
PSMC6, DR1, GTF2A2, CAND1
TMED5, GOLPH3
CCNT2, CDKN1B, PSMD10, CCNC
SSB, SNRPG
STRAP, SNX25
INSIG1, ACADL
DHX15, DDX5
ABCE1, DHX29
RAD51C, TOP2B
CHMP5, VPS4B
CCNT2, CCNC
HSP90AA1, MTX2
UBE2A, HLTF
PFN2, STRAP
GABARAPL2, PFN2, ATP1B3, DNAJB6
RAP2A, COPS5
HSP90AA1, UBQLN1
ATP1B3, DNAJB6
UBE2E3, UBE2D3, UBE2A
CHMP5, VPS4B
RAD51C, VPS4B
UBE2E3, UBE2D3, UBE2A
CHMP5, VPS4B
SDHD, IDH1, MDH1
BMI1, RBL2
FNBP1L, ARF4
SUB1, NFE2L2
VAMP7, UBQLN1
HSP90AA1, FNBP1L
PFN2, LIMA1
PIGY, DPM1
CDKN1B, MAD2L1
CHMP5, VPS4B
HSP90AA1, PTS

HINT1, DDX5
GABARAPL2, COPB1
GOLPH3, VAMP7
STRAP, SNRPG
METAP2, FNTA
SRP54, SECISBP2L
ITGAV, HINT1, TOP2B
GABARAPL2, VAMP7, SEC22C
CHMP5, VPS4B
CHMP5, VPS4B
UBE2E3, UBE2D3, UBE2A
PSMC6, PSMD12, ITGAV, PSMD10
TMED7, TMED2, GOLPH3, COPB1, ARF4
PSMC6, PSMD12, PSMD10, UBA3
PSMC6, PSMD12, PSMD10
GABARAPL2, TBCA
UBE2E3, ARMC10, SOCS4
PSMC6, MAD2L1, PSMD12, PSMD10
IDH1, MDH1
CHMP5, VPS4B
VAMP7, SEC22C
PSMC6, MAD2L1, PSMD12, PSMD10
HSP90AA1, CPNE3
SQLE, INSIG1
FAM135A, LYPLAL1
RAN, DDX5
EIF4G2, CDKN1B
CHMP5, VAMP7
CCNT2, CDKN1B
CDKN1B, TBCA, HSPE1, DNAJB6
EIF4G2, DHX29, COPS5
PSMC6, MAD2L1, PSMD12, PSMD10
AGPAT5, PTDSS1
OSTC, DPM1
TBL1XR1, UBE2D3, UBE2A, PSMC6, MAD2L1, PSMD12, PSMD10, SIAH1, PCNP, NFE2L2
BCOR, DNAJB6
CDKN1B, ATP1B3, KCNN2, VPS4B
PSMC6, SIAH1
TBL1XR1, FAM135A
GABARAPL2, UBQLN1
FNTA, CPNE3
HSP90AA1, HSPE1
DHX29, DHX15, DDX5
SUMO2, PSMD10, SOCS4
ENY2, CCNT2, GTF2E2, GTF2A2
RAP2A, ARMCX3
DPY30, CHMP5, VPS4B
NFE2L2, SERP1
ABCE1, EIF4G2, DHX29, COPS5, RPL34, RPS21
UBE2D3, UBE2A, PSMC6, PSMD12, PSMD10, SIAH1, FBXO3, HLTF
ARL6IP1, PRDX3, DNAJB6
CCNT2, GTF2E2, GTF2A2
SRP54, RPL34, RPS21, SRP9

GOLPH3, HNRNPH1
MRPL13, HSP90AA1, PAIP2, G3BP2, SSB
HSP90AA1, GMNN, TOP2B, BCOR
UBE2D3, UBE2A
PDCD10, PRDX3
PSMC6, YWHAZ, PSMD12, PSMD10
SRP54, RAN
UBE2D3, UBA3, CSNK1G3, SERP1
EIF4G2, SERP1
TMED7, TMED2, COPB1, VAMP7, ARF4, SEC22C
ITGAV, CD46, DYNLT1
EIF4G2, LIMA1, YWHAZ, ZC3H15, DHX29, FNBP1L, CHMP5, RAN, RPL34, IDH1
PFN2, LAMP2, HSP90AA1, PDCD10, ATP1B3
ITGAV, DDX5
IDH1, GLO1
CPNE3, DDX5
EIF4G2, LIMA1, YWHAZ, ZC3H15, DHX29, FNBP1L, CHMP5, RAN, RPL34, IDH1
BMI1, RBL2, HLTF, ASF1A
ABCE1, PSMC6, HSP90AA1, VPS4B, ATP5F1, HLTF
ACADL, HPRT1
PSMC6, UBQLN1
SFT2D1, GOLT1B, COPB1, VAMP7, CPNE3
HSP90AA1, SERP1
HSP90AA1, CDKN1B
PSMC6, PSMD12, PSMD10
CDKN1B, RBL2
SQLE, ACADL
MGST3, SPTLC1, PTDSS1
TBL1XR1, PDCD10, RPS21
NDUFB3, TMEM126B
RAP2A, ATP1B3
STRAP, SNX25
PRDX3, NFE2L2
PSMD10, UBQLN1
DHX29, SSB, HNRNPH1
SFT2D1, ENY2, GABARAPL2, TMED7, TMED5, CHMP5, GOLT1B, ARF4, SNX25, VPS4B, SEC22C, SERP1
PGM2, SERP1
ITGAV, CD46
SQLE, INSIG1
MED31, ENY2, COPS5, SUB1, RAN, GTF2A2, DDX5
RAP2A, NFE2L2
ATP6V1A, TMED7, TMED5, SYPL1, ATP1B3, TMED2, VAMP7, G3BP2, SEC22C, SLC35F5
PFN2, PDCD10
MGST3, PRDX3
PSMC6, PSMD12, PSMD10
HSP90AA1, HSPE1, DNAJB6
GABARAPL2, ATP1B3
HSP90AA1, ITGAV
PRDX3, UBQLN1
YWHAZ, METAP2, STRAP, SUMO2, GTF2E2, MRPL13, FUBP3, DHX15, LSM3, HSPE1, RPS21, SRP54, ZC3H15, [illegible]
DHX15, LSM3, SNRNP27, DDX5, HNRNPH1, SNRPG
TMED2, GOLPH3
VAMP7, STXBP3

CD46, CAND1
HSP90AA1, DNAJB6
MED31, GTF2E2, GTF2A2, CCNC
GABARAPL2, UBQLN1
INSIG1, BCOR
PRDX3, NFE2L2
BMI1, SUMO2, TOP2B
RAD51C, CGGBP1
PSMC6, PSMD12, PSMD10
PGM2, MTHFD2, IDH1, ENOPH1, HPRT1
PSMC6, PSMD12, PSMD10
TBL1XR1, TMED2
TIAL1, HNRNPH1
RBL2, COPS5, UBA3
ARL6IP1, YWHAZ, CDKN1B, PDCD10, MAD2L1, GOLPH3, PSMD10, ARF4, GLO1, PRDX3, HIGD1A
CDKN1B, HSPE1
EIF4G2, PSMD10
PFN2, RAN, DNAJB6
CCNT2, RBL2, GMNN, SIAH1, PCNP
SDHD, ACADL
VPS4B, SIAH1, PRDX3, TOP2B
GABARAPL2, FNBP1L, VPS4B
HSP90AA1, TBCA, HSPE1, DNAJB6
RAD51C, SUB1
MED31, UBE2A, SIAH1, HLTF
UBE2D3, UBE2A, PSMC6, SIAH1
UBE2E3, UBE2D3, UBE2A, KLHL9, SIAH1, FBXO3, BCOR
PDCD10, RBL2, CD46
FNBP1L, VAMP7, CSNK1G3
RAP2A, FAM60A
NFE2L2, UBQLN1
HSP90AA1, HINT1, TIAL1, G3BP2, SSB, HNRNPH1, HPRT1
ARL6IP1, DPY30, YWHAZ, PSMC6, MAD2L1, HSP90AA1, CGGBP1, UBA3, VPS4B, DYNLT1, SIAH1, PRDX3, PTS,
TBL1XR1, DR1, GMNN, BCOR
PSMC6, PSMD12, PSMD10
LAMP2, YWHAZ, KCNN2, NFE2L2
MED31, UBE2D3, KLHL9, PCNP, SOCS4, CAND1, NFE2L2
RAD51C, MAD2L1
LAMP2, FAM3C
TBL1XR1, ZNF217, NFE2L2, BCOR
HSPE1, ASF1A
MGST3, ABHD3, CPNE3
FUBP3, PDCD10, TMED2, CD46, NFE2L2
HSP90AA1, TOP2B
ATP6AP2, CD46, TMEM123, PAQR3
EIF4G2, FUBP3, TIAL1, RPL34, LSM3, SSB, HNRNPH1, YTHDF3, SRP9, SNRPG
PSMC6, PSMD12, PSMD10
IDH1, PRDX3
YWHAZ, DR1, PSMD10, GTF2A2, BCOR
MED31, ENY2, RBL2, SUB1, TIAL1, GLO1, DDX5, HLTF
GABARAPL2, SUMO2, UBE2A, YWHAZ, HLTF
AGPAT5, ATP6AP2, INSIG1
RPL34, RPS21

RAP2A, SRP54, RAN, ARF4
ITGAV, GOLPH3, ARF4
PGM2, GLO1, MDH1
RAD51C, UBE2D3, UBE2A, ASF1A
TBL1XR1, ASF1A
PSMC6, PSMD12, PSMD10
RPL34, RPS21
SPTLC1, ATP6AP2, HINT1
ATP1B3, ITGAV
PDCD10, ITGAV, CPNE3
PSMC6, HINT1, LYPLAL1
MRPL13, COPS5, RPL34, RPS21
CCNT2, GTF2E2, FUBP3, COPS5, SUB1, GTF2A2, NFE2L2, HLTF
PSMC6, PSMD12, PSMD10, PAQR3
ZC3H15, SOCS4
CCNT2, TBL1XR1, COPS5, SUB1, CCNC, DDX5, HLTF, SUMO2, FUBP3, WDR61, GTF2A2, ARF4, ARMCX3, NFE2
EIF4G2, CDKN1B
DPY30, HSP90AA1, PDCD10, MAD2L1, KCNN2, GTF2A2, VPS4B, IDH1, PTS, HPRT1
MRPL13, RPL34, RPS21
BMI1, TBL1XR1, UBE2D3, ZNF217, STRAP, CGGBP1, DR1, PSMD10, DDX5, BCOR
FNBP1L, OSBPL9
RAD51C, CHMP5, ATP6AP2, UBQLN1, DPY30, GTF2E2, FNTA, CGGBP1, CD46, INSIG1, FBXO28, VPS4B, LSM3,
RAP2A, SRP54, HSP90AA1, RAN, ARF4
ABCE1, RAN, COPB1, GTF2A2
ENY2, CCNT2, RAN, TOP2B, ASF1A
MAD2L1, SIAH1
UBE2A, CDKN1B, PDCD10, ITGAV, TIAL1, PRDX3
YWHAZ, CDKN1B, STRAP
TMED2, RAN, COPB1
LAMP2, GOLPH3, DDX5, TOP2B
RAP2A, RAN, ARF4
RAN, KLHL9, DYNLT1
DHX15, SNRPG
ZNF217, CDKN1B, GMNN, UBA3, BCOR, DNAJB6
ABCE1, RAD51C, UBE2A, HSP90AA1, DDX5, HLTF, ATP6V1A, UBE2E3, PSMC6, UBE2D3, DHX29, UBA3, DHX15
FAM3C, CMTM6
CCNT2, MAD2L1, RAN, DYNLT1
DHX15, LSM3
HIST1H2AC, DR1, UBA3, GTF2A2, TOP2B
CCNT2, YWHAZ, PDCD10, PRDX3
CCNT2, CCNC, CPNE3, CSNK1G3
EIF4G2, BCOR
KCNN2, DDX5
WDR61, CSNK1G3
ARL6IP1, UBE2D3, TIAL1, PSMD10, SIAH1, PRDX3
ARF4, STXBP3
SQLE, DHRS7
SYPL1, CPNE3
ITGAV, DNAJB6
MTHFD2, SQLE, ACADL, HIGD1A, DHRS7, UQCRB
CD46, UBA3, SPCS1, FBXO3, ALG13
GABARAPL2, FNTA
SRP54, HSP90AA1, CDKN1B

ATP6V1A, ATP1B3
ZNF217, METTL5, DHX29, TIAL1, G3BP2, SNRNP27, DDX5, HNRNPH1, HLTF
RPL34, RPS21
PDCD10, ITGAV
FUBP3, HLTF
FUBP3, ZNF217, HLTF
RAD51C, PAIP2, SIAH1
ENY2, TBL1XR1, FUBP3, RAN
SIAH1, HLTF
BMI1, CCNT2, TBL1XR1, RBL2, HINT1, DPY30, FUBP3, ZNF217, WDR61, CGGBP1, DR1, NFE2L2, BCOR, ASF1A
MGST3, YWHAZ, HSP90AA1, ZNF217, GOLT1B, RAN, HINT1, CSNK1G3
CCNT2, CCNC, CPNE3
RAD51C, HIST1H2AC, GTF2E2, RBL2, SUB1, DR1, NFE2L2, HLTF, TOP2B, DNAJB6
ZNF217, FAM96A, METAP2, ZC3H15, COPS5, ITGAV, MCEE, SDHD, HSPE1, PTS, TOP2B, ZNHIT3
STRAP, IDH1
GOLPH3, INSIG1
HIST1H2AC, CDKN1B
BMI1, LIMA1, GLO1, SIAH1, HLTF
GTF2E2, ZNF217, HINT1, GTF2A2, HLTF, ZNHIT3
FAM3C, SIAH1
FNBP1L, ARF4
ZNF217, NFE2L2

3, ITGAV, TMEM123, HSPE1, FBXO3, BCOR, ASF1A, GABARAPL2, SRP54, ZC3H15, PAIP2, SUB1, PCNP, SSB, DDX5, UBE2E3, P

P, SSB, DDX5, UBE2E3, PSMC6, LAMP2, CDKN1B, ZNF217, WDR61, UBA3, ARF4, SPCS1, CPNE3, SNRNP27, FBXO34, HNRNPH

RNP27, FBXO34, HNRNPH1, DNAJB6, BMI1, CCNT2, LIMA1, FAM96A, COPS5, FAM3C, PRDX3, FUBP3, AGPAT5, MCEE, COPB1,

AGPAT5, MCEE, COPB1, GTF2A2, ABCE1, RAP2A, TBL1XR1, UBE2A, HSP90AA1, G3BP2, DYNLT1, CCNC, STXBP3, HLTF, EIF4

CNC, STXBP3, HLTF, EIF4G2, TBCA, SIAH1, CAND1, SRP9, UQCRB, SERP1, OSTC, YWHAZ, HPRT1, SUMO2, MRPL13, TMED5, T

UMO2, MRPL13, TMED5, TMED2, DHX15, SEC22C, PTS, PDCD10, GMNN, ATP5F1, SCOC, FAM60A, MED31, MGST3, GOLPH3, P

ED31, MGST3, GOLPH3, PSMD10, DR1, KCNN2, DPM1, SECISBP2L
