# Supplementary material for: The Impact of Cand1 in Prostate Cancer
Source: Cancers (Basel). 2020 Feb 12;12(2):428. doi: 10.3390/cancers12020428 (PMC7072594; doi:10.3390/cancers12020428)
Supplement: Supplementary file 1 [file cancers-12-00428-s001.zip › Supplementary material revision/Supplementary_table_S4.pdf]

Regulation in Cand1^{hi} samples	Entrez Gene ID	Gene Symbol
downregulated	3783	KCNN4
downregulated	8045	RASSF7
downregulated	410	ARSA
downregulated	3386	ICAM4
downregulated	2934	GSN
downregulated	1265	CNN2
downregulated	1307	COL16A1
downregulated	29924	EPN1
downregulated	6892	TAPBP
downregulated	738	C11orf2
downregulated	1186	CLCN7
downregulated	7052	TGM2
downregulated	8425	LTBP4
downregulated	23193	GANAB
downregulated	11156	PTP4A3
downregulated	4782	NFIC
downregulated	6711	SPTBN1
downregulated	6238	RRBP1
downregulated	5119	CHMP1A
downregulated	51816	CECR1
downregulated	396	ARHGDIA
downregulated	5589	PRKCSH
downregulated	5909	RAP1GAP
downregulated	256364	EML3
downregulated	91039	DPP9
downregulated	3487	IGFBP4
downregulated	81	ACTN4
downregulated	3955	LFNG
downregulated	6649	SOD3
downregulated	682	BSG
downregulated	10558	SPTLC1
downregulated	10174	SORBS3
downregulated	5360	PLTP
downregulated	10809	STARD10
downregulated	9807	IP6K1
downregulated	5225	PGC
downregulated	84875	PARP10
downregulated	1066	CES1
downregulated	4240	MFGE8
downregulated	5585	PKN1
downregulated	79007	DBNDD1
downregulated	2022	ENG
downregulated	80024	SLC24A6
downregulated	54756	IL17RD
downregulated	10025	MED16
downregulated	7094	TLN1
downregulated	84164	ASCC2
downregulated	10312	TCIRG1
downregulated	1509	CTSD
downregulated	10362	HMG20B
downregulated	1727	CYB5R3

downregulated	9289	GPR56
downregulated	6778	STAT6
downregulated	4924	NUCB1
downregulated	23646	PLD3
downregulated	11078	TRIOBP
downregulated	29903	CCDC106
downregulated	2296	FOXC1
downregulated	3916	LAMP1
downregulated	9463	PICK1
downregulated	4043	LRPAP1
downregulated	5905	RANGAP1
downregulated	23396	PIP5K1C
downregulated	375690	FAM39E
downregulated	341405	ANKRD33
downregulated	710	SERPING1
downregulated	2318	FLNC
downregulated	800	CALD1
downregulated	126393	HSPB6
downregulated	79586	CHPF
downregulated	4239	MFAP4
downregulated	25999	CLIP3
downregulated	1508	CTSB
downregulated	7791	ZYX
downregulated	114897	C1QTNF1
downregulated	7041	TGFB1I1
upregulated	10797	MTHFD2
upregulated	2961	GTF2E2
upregulated	55832	CAND1
upregulated	483	ATP1B3
upregulated	3251	HPRT1
upregulated	51053	GMNN
upregulated	56967	C14orf132
upregulated	7323	UBE2D3
upregulated	10987	COPS5
upregulated	9791	PTDSS1
upregulated	3781	KCNN2
upregulated	4085	MAD2L1
upregulated	10651	MTX2
upregulated	5889	RAD51C
upregulated	51474	LIMA1
upregulated	51566	ARMCX3
upregulated	55907	CMAS
upregulated	84661	DPY30
upregulated	6726	SRP9
upregulated	58515	SELK
upregulated	10558	SPTLC1
upregulated	4190	MDH1
upregulated	28972	SPCS1
upregulated	221154	EFHA1
upregulated	515	ATP5F1
upregulated	9117	SEC22C
upregulated	9039	UBA3
upregulated	11235	PDCD10

upregulated	51639	SF3B14
upregulated	2958	GTF2A2
upregulated	51014	TMED7
upregulated	648	BMI1
upregulated	10247	HRSP12
upregulated	8334	HIST1H2AC
upregulated	5706	PSMC6
upregulated	6392	SDHD
upregulated	7073	TIAL1
upregulated	55958	KLHL9
upregulated	11345	GABARAPL2
upregulated	10447	FAM3C
upregulated	4709	NDUFB3
upregulated	10542	HBXIP
upregulated	5934	RBL2
upregulated	25874	BRP44
upregulated	10935	PRDX3
upregulated	58478	ENOPH1
upregulated	1315	COPB1
upregulated	51510	CHMP5
upregulated	4179	CD46
upregulated	84191	FAM96A
upregulated	84233	TMEM126A
upregulated	4780	NFE2L2
upregulated	6856	SYPL1
upregulated	50999	TMED5
upregulated	9326	ZNHIT3
upregulated	10959	TMED2
upregulated	113402	SFT2D1
upregulated	58505	OSTC
upregulated	54880	BCOR
upregulated	9525	VPS4B
upregulated	6729	SRP54
upregulated	55863	TMEM126B
upregulated	7534	YWHAZ
upregulated	6993	DYNLT1
upregulated	6713	SQLE
upregulated	10175	CNIH
upregulated	54497	HEATR5B
upregulated	11017	SNRNP27
upregulated	83891	SNX25
upregulated	388962	BOLA3
upregulated	5217	PFN2
upregulated	3094	HINT1
upregulated	4259	MGST3
upregulated	57092	PCNP
upregulated	80349	WDR61
upregulated	55848	C9orf46
upregulated	6309	SC5DL
upregulated	10049	DNAJB6
upregulated	7319	UBE2A
upregulated	64083	GOLPH3
upregulated	60592	SCOC

upregulated	6637	SNRPG
upregulated	55276	PGM2
upregulated	8895	CPNE3
upregulated	2739	GLO1
upregulated	51522	TMEM14C
upregulated	55849	ALG13
upregulated	29979	UBQLN1
upregulated	28978	TMEM14A
upregulated	26273	FBXO3
upregulated	9908	G3BP2
upregulated	7764	ZNF217
upregulated	6814	STXBP3
upregulated	54874	FNBP1L
upregulated	83787	ARMC10
upregulated	9728	SECISBP2L
upregulated	3685	ITGAV
upregulated	6845	VAMP7
upregulated	56650	CLDND1
upregulated	5716	PSMD10
upregulated	1027	CDKN1B
upregulated	25994	HIGD1A
upregulated	8813	DPM1
upregulated	1982	EIF4G2
upregulated	6902	TBCA
upregulated	79718	TBL1XR1
upregulated	33	ACADL
upregulated	51026	GOLT1B
upregulated	285636	C5orf51
upregulated	5718	PSMD12
upregulated	523	ATP6V1A
upregulated	127018	LYPLAL1
upregulated	3187	HNRNPH1
upregulated	29081	METTL5
upregulated	6611	SMS
upregulated	23204	ARL6IP1
upregulated	51635	DHRS7
upregulated	57579	FAM135A
upregulated	3320	HSP90AA1
upregulated	3336	HSPE1
upregulated	7381	UQCRB
upregulated	27258	LSM3
upregulated	64430	C14orf135
upregulated	3920	LAMP2
upregulated	1665	DHX15
upregulated	54505	DHX29
upregulated	51765	MST4
upregulated	10477	UBE2E3
upregulated	83930	STARD3NL
upregulated	55783	FTSJD1
upregulated	122809	SOCS4
upregulated	119032	C10orf32
upregulated	26175	C14orf109
upregulated	10159	ATP6AP2

upregulated	7155	TOP2B
upregulated	8939	FUBP3
upregulated	84992	PIGY
upregulated	2339	FNTA
upregulated	54918	CMTM6
upregulated	171586	ABHD3
upregulated	84693	MCEE
upregulated	56942	C16orf61
upregulated	378	ARF4
upregulated	55854	ZC3H15
upregulated	27230	SERP1
upregulated	114883	OSBPL9
upregulated	28998	MRPL13
upregulated	145815	MGC18216
upregulated	5805	PTS
upregulated	51247	PAIP2
upregulated	1655	DDX5
upregulated	11171	STRAP
upregulated	3417	IDH1
upregulated	55030	FBXO34
upregulated	56943	ENY2
upregulated	6059	ABCE1
upregulated	6596	HLTF
upregulated	152559	PAQR3
upregulated	3638	INSIG1
upregulated	6741	SSB
upregulated	6613	SUMO2
upregulated	25842	ASF1A
upregulated	6227	RPS21
upregulated	10988	METAP2
upregulated	5901	RAN
upregulated	253943	YTHDF3
upregulated	23219	FBXO28
upregulated	5911	RAP2A
upregulated	6477	SIAH1
upregulated	51003	MED31
upregulated	8545	CGGBP1
upregulated	55326	AGPAT5
upregulated	375743	PTAR1
upregulated	6164	RPL34
upregulated	1456	CSNK1G3
upregulated	26996	GPR160
upregulated	27075	TSPAN13
upregulated	51527	C14orf129
upregulated	905	CCNT2
upregulated	6428	SFRS3
upregulated	114908	TMEM123
upregulated	10923	SUB1
upregulated	1810	DR1
upregulated	892	CCNC
upregulated	27249	C2orf25
upregulated	91408	BTF3L4
upregulated	80255	SLC35F5

upregulated	25843	MOBKL3
upregulated	100128266	LOC100128266
upregulated	58516	FAM60A

Gene Name

potassium intermediate/small conductance calcium-activated channel, subfamily N, member 4
Ras association (RalGDS/AF-6) domain family 7
arylsulfatase A
intercellular adhesion molecule 4 (Landsteiner-Wiener blood group)
gelsolin (amyloidosis, Finnish type)
calponin 2
collagen, type XVI, alpha 1
Homo sapiens epsin 1 (EPN1), mRNA.
Homo sapiens TAP binding protein (tapasin) (TAPBP), transcript variant 1, mRNA.
Homo sapiens chromosome 11 open reading frame 2 (C11orf2), mRNA.
chloride channel 7
transglutaminase 2 (C polypeptide, protein-glutamine-gamma-glutamyltransferase)
latent transforming growth factor beta binding protein 4
glucosidase, alpha; neutral AB
protein tyrosine phosphatase type IVA, member 3
nuclear factor I/C (CCAAT-binding transcription factor)
spectrin, beta, non-erythrocytic 1
ribosome binding protein 1 homolog 180kDa (dog)
chromatin modifying protein 1A
cat eye syndrome chromosome region, candidate 1
Rho GDP dissociation inhibitor (GDI) alpha
protein kinase C substrate 80K-H
RAP1 GTPase activating protein
Homo sapiens echinoderm microtubule associated protein like 3 (EML3), mRNA.
Homo sapiens dipeptidyl-peptidase 9 (DPP9), mRNA.
insulin-like growth factor binding protein 4
Homo sapiens actinin, alpha 4 (ACTN4), mRNA.
Homo sapiens LFNG O-fucosylpeptide 3-beta-N-acetylglucosaminyltransferase (LFNG), transcript variant 1, mRNA.
superoxide dismutase 3, extracellular
basigin (Ok blood group)
serine palmitoyltransferase, long chain base subunit 1
sorbin and SH3 domain containing 3
phospholipid transfer protein
Homo sapiens StAR-related lipid transfer (START) domain containing 10 (STARD10), mRNA.
Inositol Hexakisphosphate Kinase 1
progastricsin (pepsinogen C)
PREDICTED: Homo sapiens poly (ADP-ribose) polymerase family, member 10 (PARP10), mRNA.
carboxylesterase 1 (monocyte/macrophage serine esterase 1)
milk fat globule-EGF factor 8 protein
protein kinase N1
Homo sapiens dysbindin (dystrobrevin binding protein 1) domain containing 1 (DBNDD1), transcript variant 1, mRNA.
endoglin (Osler-Rendu-Weber syndrome 1)
Homo sapiens solute carrier family 24 (sodium/potassium/calcium exchanger), member 6 (SLC24A6), mRNA.
Homo sapiens interleukin 17 receptor D (IL17RD), mRNA.
mediator complex subunit 16
talin 1
Homo sapiens activating signal cointegrator 1 complex subunit 2 (ASCC2), mRNA.
T-cell, immune regulator 1, ATPase, H+ transporting, lysosomal V0 subunit A3
cathepsin D
high-mobility group 20B
cytochrome b5 reductase 3

G protein-coupled receptor 56
signal transducer and activator of transcription 6, interleukin-4 induced
nucleobindin 1
phospholipase D family, member 3
Homo sapiens TRIO and F-actin binding protein (TRIOBP), transcript variant 6, mRNA.
coiled-coil domain containing 106
forkhead box C1
lysosomal-associated membrane protein 1
protein interacting with PRKCA 1
low density lipoprotein receptor-related protein associated protein 1
Ran GTPase activating protein 1
Homo sapiens phosphatidylinositol-4-phosphate 5-kinase, type I, gamma (PIP5K1C), mRNA.
Homo sapiens family with sequence similarity 39, member E (FAM39E), mRNA.
Homo sapiens ankyrin repeat domain 33 (ANKRD33), mRNA.
serpin peptidase inhibitor, clade G (C1 inhibitor), member 1, (angioedema, hereditary)
filamin C, gamma (actin binding protein 280)
caldesmon 1
Homo sapiens heat shock protein, alpha-crystallin-related, B6 (HSPB6), mRNA.
chondroitin polymerizing factor
microfibrillar-associated protein 4
CAP-GLY domain containing linker protein 3
Homo sapiens cathepsin B (CTSB), transcript variant 2, mRNA.
zyxin
Homo sapiens C1q and tumor necrosis factor related protein 1 (C1QTNF1), transcript variant 2, mRNA.
transforming growth factor beta 1 induced transcript 1
methylenetetrahydrofolate dehydrogenase (NADP+ dependent) 2, methenyltetrahydrofolate cyclohydrolase
general transcription factor IIE, polypeptide 2, beta 34kDa
cullin-associated and neddylation-dissociated 1
ATPase, Na+/K+ transporting, beta 3 polypeptide
hypoxanthine phosphoribosyltransferase 1 (Lesch-Nyhan syndrome)
geminin, DNA replication inhibitor
chromosome 14 open reading frame 132
ubiquitin-conjugating enzyme E2D 3 (UBC4/5 homolog, yeast)
COP9 constitutive photomorphogenic homolog subunit 5 (Arabidopsis)
phosphatidylserine synthase 1
potassium intermediate/small conductance calcium-activated channel, subfamily N, member 2
MAD2 mitotic arrest deficient-like 1 (yeast)
metaxin 2
Homo sapiens RAD51 homolog C (S. cerevisiae) (RAD51C), transcript variant 2, mRNA.
Homo sapiens LIM domain and actin binding 1 (LIMA1), mRNA.
Homo sapiens armadillo repeat containing, X-linked 3 (ARMCX3), transcript variant 3, mRNA.
Homo sapiens cytidine monophosphate N-acetylneuraminic acid synthetase (CMAS), mRNA.
Homo sapiens dpy-30 homolog (C. elegans) (DPY30), mRNA.
signal recognition particle 9kDa
Homo sapiens selenoprotein K (SELK), mRNA.
serine palmitoyltransferase, long chain base subunit 1
malate dehydrogenase 1, NAD (soluble)
Homo sapiens signal peptidase complex subunit 1 homolog (S. cerevisiae) (SPCS1), mRNA.
Homo sapiens EF-hand domain family, member A1 (EFHA1), mRNA.
Homo sapiens ATP synthase, H+ transporting, mitochondrial F0 complex, subunit B1 (ATP5F1), nuclear gene encodir
Homo sapiens SEC22 vesicle trafficking protein homolog C (S. cerevisiae) (SEC22C), transcript variant 1, mRNA.
ubiquitin like modifier activating enzyme 3
Homo sapiens programmed cell death 10 (PDCD10), transcript variant 2, mRNA.

Homo sapiens splicing factor 3B, 14 kDa subunit (SF3B14), mRNA.
general transcription factor IIA, 2, 12kDa
Homo sapiens transmembrane emp24 protein transport domain containing 7 (TMED7), mRNA.
BMI1 polycomb ring finger oncogene
heat-responsive protein 12
histone cluster 1, H2ac
proteasome (prosome, macropain) 26S subunit, ATPase, 6
succinate dehydrogenase complex, subunit D, integral membrane protein
TIA1 cytotoxic granule-associated RNA binding protein-like 1
Homo sapiens kelch-like 9 (Drosophila) (KLHL9), mRNA.
Homo sapiens GABA(A) receptor-associated protein-like 2 (GABARAPL2), mRNA.
family with sequence similarity 3, member C
NADH dehydrogenase (ubiquinone) 1 beta subcomplex, 3, 12kDa
hepatitis B virus x interacting protein
retinoblastoma-like 2 (p130)
brain protein 44
peroxiredoxin 3
Homo sapiens enolase-phosphatase 1 (ENOPH1), mRNA.
coatomer protein complex, subunit beta 1
Homo sapiens chromatin modifying protein 5 (CHMP5), mRNA.
CD46 molecule, complement regulatory protein
Homo sapiens family with sequence similarity 96, member A (FAM96A), transcript variant 1, mRNA.
Homo sapiens transmembrane protein 126A (TMEM126A), mRNA.
nuclear factor (erythroid-derived 2)-like 2
synaptophysin-like 1
transmembrane emp24 protein transport domain containing 5
Homo sapiens zinc finger, HIT type 3 (ZNHIT3), transcript variant 1, mRNA.
transmembrane emp24 domain trafficking protein 2
Homo sapiens SFT2 domain containing 1 (SFT2D1), mRNA.
Homo sapiens oligosaccharyltransferase complex subunit (OSTC), mRNA.
Homo sapiens BCL6 co-repressor (BCOR), transcript variant 2, mRNA.
Homo sapiens vacuolar protein sorting 4 homolog B (S. cerevisiae) (VPS4B), mRNA.
signal recognition particle 54kDa
Homo sapiens transmembrane protein 126B (TMEM126B), mRNA.
tyrosine 3-monooxygenase/tryptophan 5-monooxygenase activation protein, zeta polypeptide
dynein, light chain, Tctex-type 1
squalene epoxidase
cornichon homolog (Drosophila)
Homo sapiens HEAT repeat containing 5B (HEATR5B), mRNA.
Homo sapiens small nuclear ribonucleoprotein 27kDa (U4/U6.U5) (SNRNP27), mRNA.
Homo sapiens sorting nexin 25 (SNX25), mRNA.
Homo sapiens bolA homolog 3 (E. coli) (BOLA3), transcript variant 2, mRNA.
profilin 2
histidine triad nucleotide binding protein 1
microsomal glutathione S-transferase 3
Homo sapiens PEST proteolytic signal containing nuclear protein (PCNP), mRNA.
Homo sapiens WD repeat domain 61 (WDR61), mRNA.
Homo sapiens chromosome 9 open reading frame 46 (C9orf46), mRNA.
sterol-C5-desaturase (ERG3 delta-5-desaturase homolog, S. cerevisiae)-like
Homo sapiens DnaJ (Hsp40) homolog, subfamily B, member 6 (DNAJB6), transcript variant 1, mRNA.
ubiquitin-conjugating enzyme E2A (RAD6 homolog)
Homo sapiens golgi phosphoprotein 3 (coat-protein) (GOLPH3), mRNA.
Homo sapiens short coiled-coil protein (SCOC), mRNA.

small nuclear ribonucleoprotein polypeptide G
Homo sapiens phosphoglucomutase 2 (PGM2), mRNA.
Homo sapiens copine III (CPNE3), mRNA.
glyoxalase I
Homo sapiens transmembrane protein 14C (TMEM14C), mRNA.
Homo sapiens asparagine-linked glycosylation 13 homolog (S. cerevisiae) (ALG13), mRNA.
Homo sapiens ubiquilin 1 (UBQLN1), transcript variant 1, mRNA.
Homo sapiens transmembrane protein 14A (TMEM14A), mRNA.
Homo sapiens F-box protein 3 (FBXO3), transcript variant 2, mRNA.
GTPase activating protein (SH3 domain) binding protein 2
zinc finger protein 217
syntaxin binding protein 3
Homo sapiens formin binding protein 1-like (FNBP1L), transcript variant 1, mRNA.
Homo sapiens armadillo repeat containing 10 (ARMC10), mRNA.
SECIS binding protein 2 like
integrin, alpha V (vitronectin receptor, alpha polypeptide, antigen CD51)
vesicle associated membrane protein 7
Homo sapiens claudin domain containing 1 (CLDND1), transcript variant 1, mRNA.
proteasome (prosome, macropain) 26S subunit, non-ATPase, 10
cyclin-dependent kinase inhibitor 1B (p27, Kip1)
Homo sapiens HIG1 hypoxia inducible domain family, member 1A (HIGD1A), transcript variant 1, mRNA.
dolichyl-phosphate mannosyltransferase polypeptide 1, catalytic subunit
eukaryotic translation initiation factor 4 gamma, 2
tubulin folding cofactor A
Homo sapiens transducin (beta)-like 1X-linked receptor 1 (TBL1XR1), mRNA.
acyl-Coenzyme A dehydrogenase, long chain
Homo sapiens golgi transport 1 homolog B (S. cerevisiae) (GOLT1B), mRNA.
Homo sapiens chromosome 5 open reading frame 51 (C5orf51), mRNA.
proteasome (prosome, macropain) 26S subunit, non-ATPase, 12
ATPase, H+ transporting, lysosomal 70kDa, V1 subunit A
Homo sapiens lysophospholipase-like 1 (LYPLAL1), mRNA.
heterogeneous nuclear ribonucleoprotein H1
Homo sapiens methyltransferase like 5 (METTL5), mRNA.
spermine synthase
Homo sapiens ADP-ribosylation factor-like 6 interacting protein 1 (ARL6IP1), mRNA.
Homo sapiens dehydrogenase/reductase (SDR family) member 7 (DHRS7), mRNA.
Homo sapiens family with sequence similarity 135, member A (FAM135A), mRNA.
Homo sapiens heat shock protein 90kDa alpha (cytosolic), class A member 1 (HSP90AA1), transcript variant 1, mRN
heat shock 10kDa protein 1 (chaperonin 10)
ubiquinol-cytochrome c reductase binding protein
LSM3 homolog, U6 small nuclear RNA associated (S. cerevisiae)
Homo sapiens chromosome 14 open reading frame 135 (C14orf135), mRNA.
lysosomal-associated membrane protein 2
DEAH (Asp-Glu-Ala-His) box polypeptide 15
Homo sapiens DEAH (Asp-Glu-Ala-His) box polypeptide 29 (DHX29), mRNA.
Homo sapiens serine/threonine protein kinase MST4 (MST4), transcript variant 1, mRNA.
PREDICTED: Homo sapiens ubiquitin-conjugating enzyme E2E 3 (UBC4/5 homolog, yeast), transcript variant 2 (UBE
Homo sapiens STARD3 N-terminal like (STARD3NL), mRNA.
Homo sapiens FtsJ methyltransferase domain containing 1 (FTSJD1), transcript variant 1, mRNA.
Homo sapiens suppressor of cytokine signaling 4 (SOCS4), transcript variant 1, mRNA.
Homo sapiens chromosome 10 open reading frame 32 (C10orf32), mRNA.
Homo sapiens chromosome 14 open reading frame 109 (C14orf109), transcript variant 2, mRNA.
ATPase, H+ transporting, lysosomal accessory protein 2

topoisomerase (DNA) II beta 180kDa
PREDICTED: Homo sapiens far upstream element (FUSE) binding protein 3 (FUBP3), mRNA.
Homo sapiens phosphatidylinositol glycan anchor biosynthesis, class Y (PIGY), transcript variant 2, mRNA.
farnesyltransferase, CAAX box, alpha
Homo sapiens CKLF-like MARVEL transmembrane domain containing 6 (CMTM6), mRNA.
Homo sapiens abhydrolase domain containing 3 (ABHD3), mRNA.
Homo sapiens methylmalonyl CoA epimerase (MCEE), mRNA.
Homo sapiens chromosome 16 open reading frame 61 (C16orf61), mRNA.
ADP-ribosylation factor 4
zinc finger CCCH-type containing 15
stress associated endoplasmic reticulum protein 1
Homo sapiens oxysterol binding protein-like 9 (OSBPL9), transcript variant 7, mRNA.
Homo sapiens mitochondrial ribosomal protein L13 (MRPL13), nuclear gene encoding mitochondrial protein, mRNA.
PREDICTED: Homo sapiens hypothetical protein MGC18216 (MGC18216), mRNA.
6-pyruvoyltetrahydropterin synthase
Homo sapiens poly(A) binding protein interacting protein 2 (PAIP2), transcript variant 2, mRNA.
DEAD (Asp-Glu-Ala-Asp) box polypeptide 5
serine/threonine kinase receptor associated protein
isocitrate dehydrogenase 1 (NADP+), soluble
Homo sapiens F-box protein 34 (FBXO34), mRNA.
Homo sapiens enhancer of yellow 2 homolog (Drosophila) (ENY2), mRNA.
ATP-binding cassette, sub-family E (OABP), member 1
helicase-like transcription factor
Homo sapiens progestin and adipoQ receptor family member III (PAQR3), mRNA.
insulin induced gene 1
Sjogren syndrome antigen B (autoantigen La)
Homo sapiens SMT3 suppressor of mif two 3 homolog 2 (S. cerevisiae) (SUMO2), transcript variant 2, mRNA.
ASF1 anti-silencing function 1 homolog A (S. cerevisiae)
ribosomal protein S21
Homo sapiens methionyl aminopeptidase 2 (METAP2), mRNA.
RAN, member RAS oncogene family
Homo sapiens YTH domain family, member 3 (YTHDF3), mRNA.
Homo sapiens F-box protein 28 (FBXO28), mRNA.
Homo sapiens RAP2A, member of RAS oncogene family (RAP2A), mRNA.
seven in absentia homolog 1 (Drosophila)
Homo sapiens mediator complex subunit 31 (MED31), mRNA.
Homo sapiens CGG triplet repeat binding protein 1 (CGGBP1), transcript variant 1, mRNA.
Homo sapiens 1-acylglycerol-3-phosphate O-acyltransferase 5 (lysophosphatidic acid acyltransferase, epsilon) (AGPA
Homo sapiens protein prenyltransferase alpha subunit repeat containing 1 (PTAR1), mRNA.
ribosomal protein L34
Homo sapiens casein kinase 1, gamma 3 (CSNK1G3), transcript variant 2, mRNA.
Homo sapiens G protein-coupled receptor 160 (GPR160), mRNA.
tetraspanin 13
Homo sapiens chromosome 14 open reading frame 129 (C14orf129), mRNA.
Homo sapiens cyclin T2 (CCNT2), transcript variant b, mRNA.
splicing factor, arginine/serine-rich 3
Homo sapiens transmembrane protein 123 (TMEM123), mRNA.
SUB1 homolog (S. cerevisiae)
down-regulator of transcription 1, TBP-binding (negative cofactor 2)
Homo sapiens cyclin C (CCNC), transcript variant 1, mRNA.
Homo sapiens chromosome 2 open reading frame 25 (C2orf25), mRNA.
Homo sapiens basic transcription factor 3-like 4 (BTF3L4), mRNA.
Homo sapiens solute carrier family 35, member F5 (SLC35F5), mRNA.

MOB family member 4, phocein
PREDICTED: Homo sapiens misc_RNA (LOC100128266), miscRNA.
Homo sapiens family with sequence similarity 60, member A (FAM60A), transcript variant 2, mRNA.
