# Supplementary material for: The Impact of Cand1 in Prostate Cancer
Source: Cancers (Basel). 2020 Feb 12;12(2):428. doi: 10.3390/cancers12020428 (PMC7072594; doi:10.3390/cancers12020428)
Supplement: Supplementary file 1 [file cancers-12-00428-s001.zip › Supplementary material revision/Supplementary_Table_S6.pdf]

GO term

GO:0045159~myosin II binding
GO:0005024~transforming growth factor beta-activated receptor activity
GO:0017166~vinculin binding
GO:0043394~proteoglycan binding
GO:0009268~response to pH
GO:0051693~actin filament capping
GO:0051016~barbed-end actin filament capping
GO:0017049~GTP-Rho binding
GO:0050431~transforming growth factor beta binding
GO:0005539~glycosaminoglycan binding
GO:0030506~ankyrin binding
GO:0046697~decidualization
GO:0017015~regulation of transforming growth factor beta receptor signaling pathway
GO:0048844~artery morphogenesis
GO:0010906~regulation of glucose metabolic process
GO:0072661~protein targeting to plasma membrane
GO:0005080~protein kinase C binding
GO:0001974~blood vessel remodeling
GO:0004190~aspartic-type endopeptidase activity
GO:0005178~integrin binding
GO:0006911~phagocytosis, engulfment
GO:0042475~odontogenesis of dentin-containing tooth
GO:0006695~cholesterol biosynthetic process
GO:0001756~somitogenesis
GO:0050681~androgen receptor binding
GO:0030521~androgen receptor signaling pathway
GO:0030163~protein catabolic process
GO:0001541~ovarian follicle development
GO:0008015~blood circulation
GO:0071260~cellular response to mechanical stimulus
GO:0035914~skeletal muscle cell differentiation
GO:0030374~ligand-dependent nuclear receptor transcription coactivator activity
GO:0022617~extracellular matrix disassembly
GO:0051015~actin filament binding
GO:0043410~positive regulation of MAPK cascade
GO:0006928~movement of cell or subcellular component
GO:0005518~collagen binding
GO:0044267~cellular protein metabolic process
GO:0004197~cysteine-type endopeptidase activity
GO:0019903~protein phosphatase binding
GO:0030336~negative regulation of cell migration
GO:0030512~negative regulation of transforming growth factor beta receptor signaling pathway
GO:0030574~collagen catabolic process
GO:0002576~platelet degranulation
GO:0030855~epithelial cell differentiation
GO:0006936~muscle contraction
GO:0005200~structural constituent of cytoskeleton
GO:0007219~Notch signaling pathway
GO:0001558~regulation of cell growth
GO:0042060~wound healing
GO:0046718~viral entry into host cell

GO:0019904~protein domain specific binding
GO:0006890~retrograde vesicle-mediated transport, Golgi to ER
GO:0006914~autophagy
GO:0007565~female pregnancy
GO:0006457~protein folding
GO:0007179~transforming growth factor beta receptor signaling pathway
GO:0003824~catalytic activity
GO:0005516~calmodulin binding
GO:0046854~phosphatidylinositol phosphorylation
GO:0098641~cadherin binding involved in cell-cell adhesion
GO:0008289~lipid binding
GO:0007229~integrin-mediated signaling pathway
GO:0016337~single organismal cell-cell adhesion
GO:0008201~heparin binding
GO:0051082~unfolded protein binding
GO:0098609~cell-cell adhesion
GO:0003779~actin binding
GO:0044325~ion channel binding
GO:0001666~response to hypoxia
GO:0007155~cell adhesion
GO:0042127~regulation of cell proliferation
GO:0001701~in utero embryonic development
GO:0001934~positive regulation of protein phosphorylation
GO:0030198~extracellular matrix organization
GO:0010628~positive regulation of gene expression
GO:0019221~cytokine-mediated signaling pathway
GO:0051056~regulation of small GTPase mediated signal transduction
GO:0010629~negative regulation of gene expression
GO:0001501~skeletal system development
GO:0016192~vesicle-mediated transport
GO:0007010~cytoskeleton organization
GO:0008152~metabolic process
GO:0016477~cell migration
GO:0051260~protein homooligomerization
GO:0005096~GTPase activator activity
GO:0005215~transporter activity
GO:0016032~viral process
GO:0006508~proteolysis
GO:0008017~microtubule binding
GO:0000977~RNA polymerase II regulatory region sequence-specific DNA binding
GO:0034220~ion transmembrane transport
GO:0004872~receptor activity
GO:0042981~regulation of apoptotic process
GO:0003924~GTPase activity
GO:0005102~receptor binding
GO:0005509~calcium ion binding
GO:0042803~protein homodimerization activity
GO:0003713~transcription coactivator activity
GO:0004252~serine-type endopeptidase activity
GO:0006366~transcription from RNA polymerase II promoter
GO:0000165~MAPK cascade
GO:0015031~protein transport
GO:0008134~transcription factor binding

GO:0031625~ubiquitin protein ligase binding
GO:0043547~positive regulation of GTPase activity
GO:0000122~negative regulation of transcription from RNA polymerase II promoter
GO:0007165~signal transduction
GO:0006357~regulation of transcription from RNA polymerase II promoter
GO:0043065~positive regulation of apoptotic process
GO:0019899~enzyme binding
GO:0045893~positive regulation of transcription, DNA-templated
GO:0051301~cell division
GO:0008283~cell proliferation
GO:0005515~protein binding
GO:0042802~identical protein binding
GO:0044822~poly(A) RNA binding
GO:0043066~negative regulation of apoptotic process
GO:0006468~protein phosphorylation
GO:0008270~zinc ion binding
GO:0045944~positive regulation of transcription from RNA polymerase II promoter
GO:0007275~multicellular organism development
GO:0005524~ATP binding
GO:0055114~oxidation-reduction process
GO:0003700~transcription factor activity, sequence-specific DNA binding
GO:0006351~transcription, DNA-templated
GO:0003677~DNA binding
GO:0006355~regulation of transcription, DNA-templated
GO:0046872~metal ion binding

Fold enrichment	p_{EASE}	p_{FDR}	Gene Symbols
163,1014493	0,012036689	0,325353058	TRIOBP, GSN
69,90062112	0,027863799	0,45788988	LTBP4, ENG
48,93043478	0,039569934	0,544925956	TLN1, SORBS3
44,48221344	0,043441029	0,486339425	CECR1, CTSB
39,98095238	0,048225371	0,944658146	ARSA, CLCN7
36,90549451	0,052139104	0,923112587	GSN, SPTBN1
34,26938776	0,056036977	0,920547729	TRIOBP, GSN
30,58152174	0,062567091	0,593400339	TRIOBP, PKN1
30,58152174	0,062567091	0,593400339	LTBP4, ENG
27,18357488	0,070111568	0,611312134	LTBP4, ENG
24,46521739	0,077596214	0,604064797	SPTBN1, FLNC
23,98857143	0,079094657	0,933727811	BSG, CTSB
23,98857143	0,079094657	0,933727811	LTBP4, ENG
22,8462585	0,082883261	0,920589785	FOXC1, ENG
21,80779221	0,086656504	0,919068914	C1QTNF1, IGFBP4
18,45274725	0,101597086	0,932025271	BSG, SPTBN1
15,95557656	0,014763253	0,339215849	PICK1, PKN1, PRKCSH
14,99285714	0,123557441	0,951289778	TGM2, FOXC1
14,82740448	0,124819944	0,745471394	PGC, CTSD
13,98012422	6,46E-05	0,012518759	TLN1, ACTN4, ICAM4, LTB
13,7077551	0,134338376	0,95790116	GSN, MFGE8
13,08467532	0,021481146	0,977954374	BSG, FOXC1, NFIC
12,62556391	0,144988595	0,96318983	CYB5R3, CES1
12,3018315	0,14850989	0,961559028	FOXC1, LFNG
12,2326087	0,14925298	0,77708109	PKN1, TGFB1I1
11,70174216	0,15550966	0,958465678	MED16, TGFB1I1
11,70174216	0,15550966	0,958465678	PGC, CTSD
11,42312925	0,158988249	0,952244552	FOXC1, LFNG
10,6615873	0,169339507	0,957322355	CYB5R3, SERPING1
10,1360161	0,034472993	0,97521605	CNN2, ENG, ARHGDIA
9,791253644	0,182946098	0,956359876	ANKRD33, HMG20B
9,594202899	0,186296346	0,812699725	ACTN4, PKN1
9,469172932	0,039024308	0,950055132	BSG, GSN, ENG
9,2671278	0,001975844	0,175382271	TRIOBP, TLN1, ACTN4, PI(
8,884656085	0,043790709	0,947648856	SORBS3, C1QTNF1, IGFBI
8,368106312	0,048761817	0,928246222	TLN1, CALD1, ARHGDIA
8,155072464	0,215418652	0,849278142	C1QTNF1, CTSB
8,131719128	0,012659645	0,965164266	GSN, CECR1, MFGE8, IGF
8,021382751	0,218590373	0,842749991	CTSD, CTSB
7,76673568	0,224895964	0,841169713	STAT6, KCNN4
7,575338346	0,058193766	0,912008293	CNN2, ENG, ARHGDIA
7,496428571	0,232042422	0,976728184	TGFB1I1, ENG
7,496428571	0,232042422	0,976728184	CTSD, CTSB
6,986962552	0,067061885	0,926688073	TLN1, ACTN4, SERPING1
6,853877551	0,250856412	0,981785572	CES1, CTSB
6,72576769	0,071654482	0,926627689	TLN1, SORBS3, CALD1
6,672332016	0,072623926	0,601038377	TLN1, SORBS3, SPTBN1
6,257888199	0,081134314	0,927420657	PTP4A3, FOXC1, EPN1
5,997142857	0,281208633	0,988457495	LTBP4, IGFBP4
5,997142857	0,281208633	0,988457495	CNN2, ENG
5,997142857	0,281208633	0,988457495	LAMP1, CTSB

5,881061873	0,009867133	0,320725042	LAMP1, CHMP1A, GSN, PI
5,85087108	0,287132005	0,988428355	PICK1, TAPBP
5,410955961	0,103746029	0,92747154	LAMP1, ARSA, CTSD
5,390690209	0,30748745	0,991109801	COL16A1, EPN1
5,330793651	0,037915459	0,966460036	GANAB, LTBP4, PRKCSH,
5,214906832	0,316034852	0,991487265	ZYX, ENG
5,205365402	0,040207848	0,516885289	PLD3, SPTLC1, MED16, AI
5,177823787	0,040741875	0,491311807	KCNN4, CALD1, SPTBN1,
5,103951368	0,321675272	0,991407269	PIP5K1C, IP6K1
5,061769115	0,006238826	0,33421867	TLN1, BSG, CALD1, SPTBI
4,860639217	0,123771568	0,761023866	STARD10, PLTP, EPN1
4,846176046	0,335576548	0,992529068	ZYX, COL16A1
4,750212164	0,341058108	0,992440406	ICAM4, PIP5K1C
4,587228261	0,135936439	0,759386226	CECR1, SOD3, LRPAP1
4,448221344	0,359457082	0,949973094	LRPAP1, TAPBP
4,425935688	0,025312514	0,965879517	BSG, CALD1, SPTBN1, RA
4,400218955	0,025760224	0,470665423	ACTN4, GSN, CALD1, SPT
4,330126972	0,367217465	0,948931377	ACTN4, PRKCSH
4,184053156	0,157408784	0,955508196	ACTN4, ENG, SOD3
4,181014628	0,002727335	0,762898986	SORBS3, ICAM4, MFGE8,
3,89003861	0,176308967	0,959003768	STAT6, CNN2, ENG
3,848433919	0,179249315	0,95734404	FOXC1, PRKCSH, EPN1
3,777727784	0,408389435	0,996466354	CLIP3, ENG
3,671720117	0,192572432	0,960074972	BSG, ICAM4, COL16A1
3,662377317	0,093089108	0,923822332	GSN, C1QTNF1, CNN2, EN
3,662377317	0,418127673	0,996285621	TGM2, IL17RD
3,580383795	0,425327517	0,996353986	RAP1GAP, ARHGDIA
3,50198123	0,432439549	0,996418664	PARP10, ENG
3,50198123	0,432439549	0,996418664	FOXC1, IGFBP4
3,156390977	0,466719027	0,997835819	CHMP1A, LRPAP1
2,979946761	0,486298619	0,998105122	SPTBN1, CNN2
2,855782313	0,501035825	0,998383909	CES1, ARSA
2,789368771	0,509269184	0,998447833	FOXC1, ENG
2,710573043	0,519372808	0,998561707	C1QTNF1, TGM2
2,630668537	0,310150203	0,924657409	RAP1GAP, RANGAP1, ARI
2,422298752	0,55967499	0,993250931	TCIRG1, STARD10
2,406880076	0,348582735	0,991818226	TLN1, MFGE8, ZYX
2,398857143	0,149796767	0,957889681	CHMP1A, PGC, CTSD, DPI
2,352424749	0,570338568	0,993206237	EML3, CLIP3
2,352424749	0,570338568	0,993206237	ACTN4, FOXC1
2,28462585	0,581101348	0,999456413	TCIRG1, CLCN7
2,254858746	0,585858551	0,993628595	RRBP1, MED16
2,252448021	0,586309217	0,999448247	ACTN4, CTSB
2,091044221	0,613681912	0,994210794	RAP1GAP, TGM2
2,079196945	0,417530221	0,966619415	PICK1, IGFBP4, LRPAP1
2,047298526	0,161979415	0,791187039	NUCB1, ACTN4, GSN, LTB
2,010839786	0,170562644	0,795154656	CHMP1A, RAP1GAP, HSPI
1,973001403	0,635205545	0,995081012	MED16, TGFB1I1
1,91884058	0,64551983	0,995116901	CTSD, CTSB
1,870453913	0,352998165	0,991607331	MED16, FOXC1, TGFB1I1,
1,831188659	0,662871716	0,999870673	SPTBN1, IL17RD
1,821916817	0,485271969	0,99827658	CHMP1A, ACTN4, RRBP1
1,722902633	0,685274956	0,99643208	SORBS3, FOXC1

1,704893198	0,689127764	0,996139263	TRIOBP, RANGAP1
1,698305942	0,411296022	0,9962441	RAP1GAP, SPTBN1, RANG
1,665873016	0,343200745	0,991901339	STAT6, SORBS3, ANKRD3
1,652959272	0,198462946	0,960776322	STAT6, RAP1GAP, PKN1, I
1,631875607	0,544442004	0,998997983	STAT6, MED16, PKN1
1,599238095	0,712468531	0,99995915	TGM2, CLIP3
1,469382426	0,742701795	0,997879691	LAMP1, PICK1
1,39739251	0,629539055	0,999753114	MED16, FOXC1, TGFB1I1
1,37077551	0,76691844	0,999991095	TRIOBP, CHMP1A
1,310850898	0,78209237	0,999992559	FOXC1, IGFBP4
1,308896093	0,006496117	0,27219256	TLN1, RAP1GAP, CALD1, L
1,306553666	0,586229787	0,992675105	STAT6, CHMP1A, PICK1, D
1,083490584	0,675125576	0,996381091	GANAB, ACTN4, RRBP1, S
1,0544427	0,850332525	0,999999499	TGM2, ARHGDIA
1,052130326	0,850964652	0,999999403	PICK1, PKN1
1,046416484	0,701877981	0,996371838	CHMP1A, CECR1, TGFB1I
0,978127275	0,775772021	0,999992188	STAT6, FOXC1, NFIC, ENG
0,920866466	0,886872722	0,999999906	LTBP4, CECR1
0,818234695	0,863415548	0,999852682	TGM2, PKN1, PIP5K1C, IP
0,81042471	0,916392503	0,999999983	CYB5R3, SOD3
0,763742478	0,905603642	0,999963847	STAT6, FOXC1, NFIC
0,736222141	0,916186129	0,999999987	STAT6, CHMP1A, ASCC2,
0,73074126	0,915613544	0,999971924	NUCB1, STAT6, HMG20B,
0,637993921	0,953288547	1	ASCC2, HMG20B, FOXC1,
0,5912329	0,97385718	0,999999729	CHPF, PICK1, TGM2, LFNG

ICAM4, LTBP4, MFGE8, COL16A1

l, ACTN4, PICK1, FLNC
QTNF1, IGFBP4

MFGE8, IGFBP4

P1A, GSN, PICK1, TGM2

4, PRKCSH, LRPAP1

1, MED16, ARSA
1, SPTBN1, CNN2

ALD1, SPTBN1, RANGAP1, CNN2

SPTBN1, RANGAP1, CNN2
CALD1, SPTBN1, CNN2

M4, MFGE8, TGFB1I1, ZYX, MFAP4, COL16A1, ENG

F1, CNN2, ENG

ANGAP1, ARHGDIA

C, CTSD, DPP9, CTSB

I4, GSN, LTBP4, ARSA, PRKCSH
P1GAP, HSPB6, ACTN4, CECR1, ENG

C1, TGFB1I1, NFIC

PTBN1, RANGAP1, ARHGDIA
S3, ANKRD33, NFIC, ENG
GAP, PKN1, RANGAP1, RASSF7, ZYX, IL17RD, IGFBP4

AP, CALD1, LTBP4, PIP5K1C, RANGAP1, STARD10, RASSF7, TAPBP, STAT6, SORBS3, GSN, C1QTNF1, TGM2, ZYX, IP6K1, CCD
P1A, PICK1, DPP9
N4, RRBP1, SPTBN1, ZYX

CR1, TGFB1I1, ZYX, SOD3
1, NFIC, ENG

PIP5K1C, IP6K1, CLCN7

P1A, ASCC2, PKN1, HMG20B, NFIC
6, HMG20B, FOXC1, NFIC
20B, FOXC1, ENG
TGM2, LFNG, SOD3

1, TGM2, ZYX, IP6K1, CCDC106, ARHGDIA, PLD3, BSG, SPTLC1, ACTN4, PICK1, PKN1, HMG20B, SERPING1, PARP10, FLNC, C

PING1, PARP10, FLNC, COL16A1, SOD3, LRPAP1, NUCB1, KCNN4, LAMP1, CHMP1A, HSPB6, MED16, ARSA, CTSD, SPTBN1, F[illegible]

ARSA, CTSD, SPTBN1, FOXC1, CTSB, TGFB1I1, CLIP3, MFAP4, ENG, EPN1
