# Supplementary material for: The Impact of Cand1 in Prostate Cancer
Source: Cancers (Basel). 2020 Feb 12;12(2):428. doi: 10.3390/cancers12020428 (PMC7072594; doi:10.3390/cancers12020428)
Supplement: Supplementary file 1 [file cancers-12-00428-s001.zip › Supplementary material revision/Supplementary_table_S3.pdf]

Study	Probe ID	Entrez Gene ID	Gene Symbol	log2 Fold Regulati
GSE16560	DAP1_3608	2354	FOSB	-0,612911276
GSE16560	DAP1_2352	6436	SFTPA2	-0,609640044
GSE16560	DAP2_0991	8537	BCAS1	-0,582926226
GSE16560	DAP2_1680	3783	KCNN4	-0,568955782
GSE16560	DAP3_2403	1485	CTAG1B	-0,551104594
GSE16560	DAP3_5268	2953	GSTT2	-0,549359439
GSE16560	DAP3_4225	10866	HCP5	-0,549258237
GSE16560	DAP2_3153	2786	GNG4	-0,527461331
GSE16560	DAP3_3706	4330	MN1	-0,517239952
GSE16560	DAP3_1375	27253	PCDH17	-0,513022253
GSE16560	DAP1_5829	4495	MT1G	-0,511126445
GSE16560	DAP2_4227	2330	FMO5	-0,510800989
GSE16560	DAP3_3870	3386	ICAM4	-0,500948192
GSE16560	DAP1_0296	4793	NFKBIB	-0,497180151
GSE16560	DAP3_5177	6779	STATH	-0,478027715
GSE16560	DAP3_1868	410	ARSA	-0,477639584
GSE16560	DAP1_2943	2998	GYS2	-0,463310908
GSE16560	DAP2_3561	1803	DPP4	-0,436799494
GSE16560	DAP3_1693	8045	RASSF7	-0,42886378
GSE16560	DAP3_5091	634	CEACAM1	-0,413695015
GSE16560	DAP1_5854	5080	PAX6	-0,37600828
GSE16560	DAP2_2962	217	ALDH2	-0,36658444
GSE16560	DAP4_1851	3439	IFNA1	-0,363638664
GSE16560	DAP3_5698	717	C2	-0,358046116
GSE16560	DAP4_1170	4494	MT1F	-0,33714565
GSE16560	DAP4_3561	688	KLF5	-0,332440343
GSE16560	DAP4_3988	6304	SATB1	-0,328821804
GSE16560	DAP1_1432	5164	PDK2	-0,327293428
GSE16560	DAP4_2021	4276	MICA	-0,322242539
GSE40272	X12127	7306	TYRP1	-2,606622093
GSE40272	X33466	6005	RHAG	-2,426661734
GSE40272	X24655	11339	OIP5	-2,356292283
GSE40272	X27325	121355	GTSF1	-2,237014271
GSE40272	X14922	1244	ABCC2	-2,050808668
GSE40272	X34378	10874	NMU	-2,039205074
GSE40272	X15839	7306	TYRP1	-1,977973044
GSE40272	X36709	4102	MAGEA3	-1,897214588
GSE40272	X26954	1081	CGA	-1,837656448
GSE40272	X1394	9837	GINS1	-1,80085518
GSE40272	X2848	2078	ERG	-1,776527484
GSE40272	X16909	84528	RHOXF2	-1,736748943
GSE40272	X35082	10642	IGF2BP1	-1,711393235
GSE40272	X2888	199	AIF1	-1,691105708
GSE40272	X17229	3053	SERPIND1	-1,674110465
GSE40272	X27685	3747	KCNC2	-1,658002643
GSE40272	X34642	9928	KIF14	-1,64595296
GSE40272	X13196	4101	MAGEA2	-1,644442918
GSE40272	X30563	10642	IGF2BP1	-1,613270085
GSE40272	X36804	8438	RAD54L	-1,611536469
GSE40272	X14163	5366	PMAIP1	-1,605041755
GSE40272	X12351	221150	SKA3	-1,601806554

GSE40272	X41659	55268	ECHDC2	-1,57592019
GSE40272	X22878	5105	PCK1	-1,530469345
GSE40272	X18917	5467	PPARD	-1,525997357
GSE40272	X26931	90632	C6orf176	-1,52145296
GSE40272	X10406	10371	SEMA3A	-1,505013742
GSE40272	X9647	3783	KCNN4	-1,49612685
GSE40272	X37174	54596	L1TD1	-1,47555074
GSE40272	X5876	55789	DEPDC1B	-1,455882664
GSE40272	X16813	7503	XIST	-1,435859408
GSE40272	X37707	91227	GGTLC2	-1,433968816
GSE40272	X7416	51200	CPA4	-1,422878964
GSE40272	X30290	1361	CPB2	-1,414602537
GSE40272	X34831	7090	TLE3	-1,409902748
GSE40272	X41188	51365	PLA1A	-1,405476744
GSE40272	X11176	5524	PPP2R4	-1,40546723
GSE40272	X40324	255738	PCSK9	-1,400770613
GSE40272	X23421	389421	LIN28B	-1,40027907
GSE40272	X28461	4547	MTTP	-1,398825581
GSE40272	X31355	7157	TP53	-1,397210888
GSE40272	X10053	11156	PTP4A3	-1,392834567
GSE40272	X27777	5225	PGC	-1,38705814
GSE40272	X35386	9582	APOBEC3B	-1,384124207
GSE40272	X6896	310	ANXA7	-1,383252643
GSE40272	X8247	54502	RBM47	-1,375784884
GSE40272	X1459	118424	UBE2J2	-1,36063055
GSE40272	X41103	10840	ALDH1L1	-1,356440275
GSE40272	X24388	3909	LAMA3	-1,345946089
GSE40272	X9780	3795	KHK	-1,338549154
GSE40272	X20456	5478	PPIA	-1,334363108
GSE40272	X38910	219848	FLJ30719	-1,331270613
GSE40272	X31425	1796	DOK1	-1,326197674
GSE40272	X34363	346547	ERVFC1-1	-1,321808668
GSE40272	X41454	5998	RGS3	-1,318747357
GSE40272	X40996	816	CAMK2B	-1,308104651
GSE40272	X30660	254910	LCE5A	-1,306554968
GSE40272	X27143	55117	SLC6A15	-1,304352008
GSE40272	X40860	162835	LOC162835	-1,303866279
GSE40272	X12916	2051	EPHB6	-1,294062368
GSE40272	X14902	5119	CHMP1A	-1,285560782
GSE40272	X20178	54869	EPS8L1	-1,28322833
GSE40272	X41710	254528	C16orf73	-1,282754228
GSE40272	X30637	90768	MGC45800	-1,282127378
GSE40272	X15949	5272	SERPINB9	-1,279132664
GSE40272	X3637	342667	STAC2	-1,275186575
GSE40272	X26144	285905	INTS4L1	-1,273284355
GSE40272	X44056	57082	CASC5	-1,262102008
GSE40272	X23609	4111	MAGEA12	-1,261752643
GSE40272	X42487	392255	GDF6	-1,260780127
GSE40272	X15306	10994	ILVBL	-1,257496829
GSE40272	X23767	131578	LRRC15	-1,25437685
GSE40272	X35304	7036	TFR2	-1,252570825
GSE40272	X15206	8402	SLC25A11	-1,244505814
GSE40272	X13612	26806	SNORD44	-1,240940803

GSE40272	X39233	472	ATM	-1,233697146
GSE40272	X1409	79763	ISOC2	-1,231684989
GSE40272	X17398	151449	GDF7	-1,226843023
GSE40272	X6849	92305	TMEM129	-1,224349894
GSE40272	X7824	310	ANXA7	-1,222986786
GSE40272	X35495	6768	ST14	-1,220802326
GSE40272	X3792	7052	TGM2	-1,215511099
GSE40272	X5212	2011	MARK2	-1,214087209
GSE40272	X27881	91614	DEPDC7	-1,213451374
GSE40272	X34311	7855	FZD5	-1,210773784
GSE40272	X12208	390613	LOC390613	-1,210033298
GSE40272	X29729	390992	HES3	-1,208695032
GSE40272	X3592	1443	CSH2	-1,207419133
GSE40272	X17565	84680	ACCS	-1,20681871
GSE40272	X34465	26267	FBXO10	-1,206107822
GSE40272	X30054	51226	COPZ2	-1,201440803
GSE40272	X4195	26493	OR8B8	-1,200421247
GSE40272	X16723	81624	DIAPH3	-1,196663848
GSE40272	X42318	3547	IGSF1	-1,196515856
GSE40272	X18215	79589	RNF128	-1,196513742
GSE40272	X30730	399983	FLJ33297	-1,1955037
GSE40272	X19330	83999	KREMEN1	-1,195158562
GSE40272	X19538	9793	CKAP5	-1,19498203
GSE40272	X7728	23555	TSPAN15	-1,193323467
GSE40272	X10980	6928	HNF1B	-1,191439218
GSE40272	X13072	440901	LOC440901	-1,190884778
GSE40272	X20089	9052	GPRC5A	-1,188265328
GSE40272	X15079	2356	FPGS	-1,186539112
GSE40272	X20126	3875	KRT18	-1,182337738
GSE40272	X8928	81622	UNC93B1	-1,179571882
GSE40272	X17303	55247	NEIL3	-1,179488372
GSE40272	X28348	63935	PCIF1	-1,178140592
GSE40272	X534	84818	IL17RC	-1,175174947
GSE40272	X10602	4035	LRP1	-1,172519027
GSE40272	X1569	2101	ESRRA	-1,169865751
GSE40272	X20792	7408	VASP	-1,168707717
GSE40272	X26469	4323	MMP14	-1,168056025
GSE40272	X3248	3909	LAMA3	-1,164634249
GSE40272	X38692	9902	MRC2	-1,163920719
GSE40272	X34492	10169	SERF2	-1,163053911
GSE40272	X43478	390063	OR51I1	-1,161506342
GSE40272	X3153	598	BCL2L1	-1,161271142
GSE40272	X38152	23428	SLC7A8	-1,16055074
GSE40272	X2472	440686	HIST2H3PS2	-1,158582981
GSE40272	X18284	5601	MAPK9	-1,157479915
GSE40272	X16890	9076	CLDN1	-1,15540222
GSE40272	X12525	6442	SGCA	-1,155158562
GSE40272	X11667	6911	TBX6	-1,154096195
GSE40272	X22299	51087	YBX2	-1,150770085
GSE40272	X27668	3875	KRT18	-1,150076638
GSE40272	X37700	4001	LMNB1	-1,148638478
GSE40272	X13588	22824	HSPA4L	-1,144663848
GSE40272	X19505	51274	KLF3	-1,142471987

GSE40272	X18960	310	ANXA7	-1,141781712
GSE40272	X38609	926	CD8B	-1,13589482
GSE40272	X24535	400768	LOC400768	-1,135738372
GSE40272	X10365	4145	MATK	-1,135281184
GSE40272	X21979	4582	MUC1	-1,130243658
GSE40272	X10509	4643	MYO1E	-1,128569767
GSE40272	X15399	92086	GGTLC1	-1,127331395
GSE40272	X13383	90381	C15orf42	-1,12726797
GSE40272	X34054	440605	LOC440605	-1,126207717
GSE40272	X5258	120793	OR56A4	-1,124005285
GSE40272	X37947	22955	SCMH1	-1,121192918
GSE40272	X16349	396	ARHGDIA	-1,117561311
GSE40272	X23047	226	ALDOA	-1,116317125
GSE40272	X27268	5696	PSMB8	-1,116211416
GSE40272	X42250	128864	C20orf144	-1,1155074
GSE40272	X4357	222029	DKFZp434L192	-1,115135835
GSE40272	X13268	344167	FOXI3	-1,114918605
GSE40272	X41334	6928	HNF1B	-1,113364165
GSE40272	X37655	10227	MFSD10	-1,113161734
GSE40272	X22174	780	DDR1	-1,112104123
GSE40272	X1070	1840	DTX1	-1,111313953
GSE40272	X26732	30837	SOCS7	-1,111158034
GSE40272	X1324	3145	HMBS	-1,110470402
GSE40272	X2938	53822	FXYD7	-1,110221459
GSE40272	X8283	4150	MAZ	-1,109356765
GSE40272	X15920	5066	PAM	-1,104729915
GSE40272	X28530	389812	LCN15	-1,104695032
GSE40272	X16956	55898	UNC45A	-1,104680761
GSE40272	X7533	9761	MLEC	-1,10451797
GSE40272	X11159	55544	RBM38	-1,103632664
GSE40272	X3981	11186	RASSF1	-1,102276427
GSE40272	X42564	10072	DPP3	-1,102265328
GSE40272	X19723	9118	INA	-1,101883721
GSE40272	X6954	85300	ATCAY	-1,101317653
GSE40272	X30781	221191	PRSS54	-1,100763214
GSE40272	X3141	56999	ADAMTS9	-1,099383721
GSE40272	X19375	9271	PIWIL1	-1,098913848
GSE40272	X9571	310	ANXA7	-1,096243129
GSE40272	X34534	221914	GPC2	-1,095752643
GSE40272	X15699	2570	GABRR2	-1,095062896
GSE40272	X28090	3909	LAMA3	-1,09314482
GSE40272	X18738	128439	SNHG11	-1,090332452
GSE40272	X33939	10994	ILVBL	-1,086528013
GSE40272	X4710	11187	PKP3	-1,085926004
GSE40272	X1493	55506	H2AFY2	-1,085486786
GSE40272	X8643	310	ANXA7	-1,085347252
GSE40272	X34601	5932	RBBP8	-1,085052326
GSE40272	X30262	8291	DYSF	-1,084912262
GSE40272	X34069	1201	CLN3	-1,083978858
GSE40272	X639	80833	APOL3	-1,082211416
GSE40272	X38406	4739	NEDD9	-1,082039112
GSE40272	X21849	410	ARSA	-1,079303911
GSE40272	X5152	112464	PRKCDBP	-1,079051268

GSE40272	X28383	1938	EEF2	-1,078700317
GSE40272	X35412	4588	MUC6	-1,077423362
GSE40272	X27758	4582	MUC1	-1,076229387
GSE40272	X12629	56052	ALG1	-1,074006342
GSE40272	X2400	7094	TLN1	-1,073325053
GSE40272	X34969	3360	HTR4	-1,072147992
GSE40272	X36252	3909	LAMA3	-1,071039112
GSE40272	X35195	3268	AGFG2	-1,070734144
GSE40272	X22724	84034	EMILIN2	-1,069859408
GSE40272	X6672	9545	RAB3D	-1,066899577
GSE40272	X3606	80728	ARHGAP39	-1,06652907
GSE40272	X42050	310	ANXA7	-1,065751586
GSE40272	X24429	55534	MAML3	-1,06455814
GSE40272	X9042	53342	IL17D	-1,064122093
GSE40272	X12084	943	TNFRSF8	-1,063189218
GSE40272	X14550	3909	LAMA3	-1,063011099
GSE40272	X23442	3955	LFNG	-1,061075581
GSE40272	X23191	6710	SPTB	-1,060935518
GSE40272	X5209	57592	ZNF687	-1,060517442
GSE40272	X42978	310	ANXA7	-1,060186047
GSE40272	X33539	57586	SYT13	-1,059862579
GSE40272	X27369	6494	SIPA1	-1,059525899
GSE40272	X26211	5360	PLTP	-1,059302326
GSE40272	X2259	440454	LOC440454	-1,056590909
GSE40272	X17464	440788	LOC440788	-1,05440222
GSE40272	X27419	9051	PSTPIP1	-1,052076638
GSE40272	X32932	5608	MAP2K6	-1,05182241
GSE40272	X35964	3909	LAMA3	-1,051705074
GSE40272	X17080	7288	TULP2	-1,050663848
GSE40272	X34024	5629	PROX1	-1,049588795
GSE40272	X34663	284695	ZNF326	-1,049359408
GSE40272	X23658	10101	NUBP2	-1,048459831
GSE40272	X39083	400832	LOC400832	-1,046849366
GSE40272	X35139	10072	DPP3	-1,046678118
GSE40272	X34988	391667	LOC391667	-1,046186575
GSE40272	X35806	2934	GSN	-1,045169662
GSE40272	X7909	4109	MAGEA10	-1,044600951
GSE40272	X693	2972	BRF1	-1,044471987
GSE40272	X16738	421	ARVCF	-1,043941332
GSE40272	X15486	5250	SLC25A3	-1,043642178
GSE40272	X30894	51282	SCAND1	-1,043504228
GSE40272	X18473	10755	GIPC1	-1,041081924
GSE40272	X1392	9590	AKAP12	-1,040788584
GSE40272	X4402	79845	RNF122	-1,040221987
GSE40272	X2082	11278	KLF12	-1,039421247
GSE40272	X37474	55898	UNC45A	-1,038914376
GSE40272	X27584	266722	HS6ST3	-1,038432347
GSE40272	X4014	81622	UNC93B1	-1,038306554
GSE40272	X12526	5522	PPP2R2C	-1,038010571
GSE40272	X14271	9840	KIAA0748	-1,035633721
GSE40272	X18726	2668	GDNF	-1,035118922
GSE40272	X37192	51069	MRPL2	-1,034860465
GSE40272	X7112	348801	LNP1	-1,034063425

GSE40272	X12769	3224	HOXC8	-1,033052326
GSE40272	X41365	5818	PVRL1	-1,032633721
GSE40272	X34586	8777	MPDZ	-1,031748943
GSE40272	X4352	116154	PHACTR3	-1,030968288
GSE40272	X22544	54498	SMOX	-1,030681818
GSE40272	X1093	253982	ASPHD1	-1,030556025
GSE40272	X7776	92497	LOC92497	-1,029607294
GSE40272	X6889	4035	LRP1	-1,026736786
GSE40272	X28171	6663	SOX10	-1,026724101
GSE40272	X41859	51703	ACSL5	-1,026401691
GSE40272	X35001	4137	MAPT	-1,026214588
GSE40272	X26447	2036	EPB41L1	-1,024792812
GSE40272	X1782	721	C4B	-1,024592495
GSE40272	X34852	441759	LOC441759	-1,024419662
GSE40272	X35515	56999	ADAMTS9	-1,023619979
GSE40272	X15358	388228	SBK1	-1,023258985
GSE40272	X32376	55741	EDEM2	-1,022388478
GSE40272	X8331	51228	GLTP	-1,022163319
GSE40272	X9507	4957	ODF2	-1,021280655
GSE40272	X19025	11060	WWP2	-1,020401691
GSE40272	X12878	22879	MON1B	-1,020150106
GSE40272	X20301	126374	WTIP	-1,01610148
GSE40272	X16650	23600	AMACR	-1,01608351
GSE40272	X5351	6659	SOX4	-1,015429704
GSE40272	X31571	83593	RASSF5	-1,01520296
GSE40272	X11360	84060	C7orf64	-1,014980444
GSE40272	X14380	3909	LAMA3	-1,014944503
GSE40272	X42954	9344	TAOK2	-1,014107294
GSE40272	X31136	219409	GSX1	-1,013874207
GSE40272	X7937	610	HCN2	-1,013801797
GSE40272	X2021	5566	PRKACA	-1,013725687
GSE40272	X31608	8943	AP3D1	-1,013448732
GSE40272	X1721	1,00E+04	AKT3	-1,012603594
GSE40272	X11378	4791	NFKB2	-1,012097252
GSE40272	X6391	116985	ARAP1	-1,011973044
GSE40272	X8277	81572	PDRG1	-1,010286469
GSE40272	X23026	440008	LOC440008	-1,010247357
GSE40272	X16758	202374	STK32A	-1,010161205
GSE40272	X42555	22876	INPP5F	-1,010100951
GSE40272	X22430	389692	MAFA	-1,008785941
GSE40272	X27063	80331	DNAJC5	-1,006703488
GSE40272	X43464	7337	UBE3A	-1,006088266
GSE40272	X43499	345537	LOC345537	-1,003967759
GSE40272	X12331	7375	USP4	-1,002116279
GSE40272	X37945	9463	PICK1	-1,001516385
GSE40272	X9598	7516	XRCC2	-1,000801268
GSE40272	X31016	112939	NACC1	-0,999543869
GSE40272	X28868	25897	RNF19A	-0,998522199
GSE40272	X6306	92922	CCDC102A	-0,998368922
GSE40272	X20914	8864	PER2	-0,997709302
GSE40272	X20346	5909	RAP1GAP	-0,996866808
GSE40272	X42489	400733	LOC400733	-0,994713002
GSE40272	X20833	10072	DPP3	-0,994163848

GSE40272	X10015	3155	HMGCL	-0,994035941
GSE40272	X10733	2099	ESR1	-0,993155391
GSE40272	X37623	9817	KEAP1	-0,991519556
GSE40272	X29298	23476	BRD4	-0,99012685
GSE40272	X28913	23432	GPR161	-0,989391649
GSE40272	X5665	900	CCNG1	-0,988174947
GSE40272	X22815	1938	EEF2	-0,987182347
GSE40272	X34729	4035	LRP1	-0,98684778
GSE40272	X6538	8535	CBX4	-0,986832981
GSE40272	X3016	649	BMP1	-0,986223044
GSE40272	X7288	55117	SLC6A15	-0,985373679
GSE40272	X8868	27022	FOXD3	-0,985275899
GSE40272	X9809	23218	NBEAL2	-0,984602008
GSE40272	X3850	345430	KRT18P41	-0,984339323
GSE40272	X7027	29948	OSGIN1	-0,983064482
GSE40272	X26950	1488	CTBP2	-0,982930233
GSE40272	X27756	23037	PDZD2	-0,982799154
GSE40272	X11485	390529	LOC390529	-0,982640063
GSE40272	X25553	146223	CMTM4	-0,981686575
GSE40272	X1430	51435	SCARA3	-0,981469345
GSE40272	X17104	310	ANXA7	-0,980595666
GSE40272	X3964	155066	ATP6V0E2	-0,980429704
GSE40272	X17195	221711	SYCP2L	-0,980167548
GSE40272	X28713	140730	RIMS4	-0,979672304
GSE40272	X11196	4739	NEDD9	-0,979338266
GSE40272	X37940	6300	MAPK12	-0,977771142
GSE40272	X9842	83459	LOC83459	-0,974520085
GSE40272	X24903	10994	ILVBL	-0,973872622
GSE40272	X37774	389721	rab1c	-0,973776427
GSE40272	X33872	3249	HPN	-0,972779598
GSE40272	X40240	80169	CTC1	-0,972468816
GSE40272	X32530	57140	RNPEPL1	-0,971036998
GSE40272	X13752	4302	MLLT6	-0,970609408
GSE40272	X19753	1371	CPOX	-0,970291226
GSE40272	X27855	254102	EHBP1L1	-0,970067125
GSE40272	X24895	2688	GH1	-0,969883721
GSE40272	X15039	7706	TRIM25	-0,968498943
GSE40272	X10601	4035	LRP1	-0,968486786
GSE40272	X15912	23303	KIF13B	-0,968140592
GSE40272	X41375	2934	GSN	-0,966639535
GSE40272	X12771	79446	WDR25	-0,966543869
GSE40272	X22549	7008	TEF	-0,96627537
GSE40272	X38712	340318	LOC340318	-0,965328753
GSE40272	X33445	4059	BCAM	-0,961842495
GSE40272	X16284	10669	CGREF1	-0,961730444
GSE40272	X35797	10994	ILVBL	-0,960774841
GSE40272	X37662	1509	CTSD	-0,960594609
GSE40272	X543	1938	EEF2	-0,960486258
GSE40272	X25958	28965	SLC27A6	-0,960403805
GSE40272	X20304	6,60E+04	TMEM108	-0,959902748
GSE40272	X39241	22941	SHANK2	-0,959585095
GSE40272	X37983	1057	CELP	-0,958801268
GSE40272	X370	9545	RAB3D	-0,958381078

GSE40272	X9197	1265	CNN2	-0,958354651
GSE40272	X4690	9261	MAPKAPK2	-0,958064482
GSE40272	X6101	6733	SRPK2	-0,957820296
GSE40272	X17247	1938	EEF2	-0,956939746
GSE40272	X23213	90139	TSPAN18	-0,955979387
GSE40272	X16629	64106	NPFFR1	-0,955942389
GSE40272	X9883	145173	B3GALTL	-0,955731501
GSE40272	X19524	1856	DVL2	-0,955618922
GSE40272	X38107	64781	CERK	-0,95520296
GSE40272	X2364	5158	PDE6B	-0,955004757
GSE40272	X16599	7881	KCNAB1	-0,95484408
GSE40272	X31848	441841	LOC441841	-0,95442389
GSE40272	X22287	3549	IHH	-0,954384249
GSE40272	X42221	171017	ZNF384	-0,95354704
GSE40272	X1472	4035	LRP1	-0,953321353
GSE40272	X44415	80144	FRAS1	-0,953174947
GSE40272	X37656	51150	SDF4	-0,95261945
GSE40272	X21246	8567	MADD	-0,952584038
GSE40272	X13220	2581	GALC	-0,952555497
GSE40272	X39206	4176	MCM7	-0,952486258
GSE40272	X3131	64755	C16orf58	-0,95192019
GSE40272	X22630	5892	RAD51D	-0,951520085
GSE40272	X32830	92070	C4orf42	-0,950729915
GSE40272	X15227	28461	IGHV1-69	-0,949986786
GSE40272	X4941	60370	AVPI1	-0,949433932
GSE40272	X39369	4035	LRP1	-0,948390063
GSE40272	X13936	2030	SLC29A1	-0,947995243
GSE40272	X15809	811	CALR	-0,947893235
GSE40272	X8054	3155	HMGCL	-0,947168605
GSE40272	X2630	3728	JUP	-0,947153277
GSE40272	X35363	1636	ACE	-0,94639482
GSE40272	X37217	55080	TAPBPL	-0,946198203
GSE40272	X30203	255512	LOC255512	-0,946169662
GSE40272	X33869	5064	PALM	-0,945805497
GSE40272	X1757	7108	TM7SF2	-0,945791226
GSE40272	X24185	116966	WDR17	-0,945553383
GSE40272	X25831	7052	TGM2	-0,945266913
GSE40272	X13349	8864	PER2	-0,943815011
GSE40272	X27739	23154	NCDN	-0,943306025
GSE40272	X9164	57670	KIAA1549	-0,943302854
GSE40272	X37302	4624	MYH6	-0,942691332
GSE40272	X5175	9509	ADAMTS2	-0,942228858
GSE40272	X15400	6080	SNORA73A	-0,941915962
GSE40272	X34864	2850	GPR27	-0,940557611
GSE40272	X29641	10840	ALDH1L1	-0,940372093
GSE40272	X17061	9569	GTF2IRD1	-0,940152748
GSE40272	X37276	348645	C22orf34	-0,940114693
GSE40272	X18332	10903	MTMR11	-0,93984408
GSE40272	X16657	283475	LOC283475	-0,938914376
GSE40272	X15285	51116	MRPS2	-0,938794926
GSE40272	X34657	1936	EEF1D	-0,938682347
GSE40272	X27077	1788	DNMT3A	-0,937791226
GSE40272	X3721	23286	WWC1	-0,937337209

GSE40272	X15547	64794	DDX31	-0,934578753
GSE40272	X32094	4035	LRP1	-0,934562896
GSE40272	X7268	2319	FLOT2	-0,933650634
GSE40272	X18634	127731	VWA5B1	-0,93355074
GSE40272	X14751	51005	AMDHD2	-0,93342759
GSE40272	X16937	339829	CCDC39	-0,932735201
GSE40272	X44075	84818	IL17RC	-0,932695032
GSE40272	X5731	10570	DPYSL4	-0,931391121
GSE40272	X33867	27343	POLL	-0,93107981
GSE40272	X8700	26048	ZNF500	-0,930878436
GSE40272	X26505	388491	LOC388491	-0,930660148
GSE40272	X37222	64753	CCDC136	-0,929974101
GSE40272	X41908	402505	LOC402505	-0,92907241
GSE40272	X4275	3554	IL1R1	-0,929030127
GSE40272	X24656	146310	RNF151	-0,928971459
GSE40272	X6199	55898	UNC45A	-0,928767442
GSE40272	X880	60343	FAM3A	-0,92823203
GSE40272	X1666	2002	ELK1	-0,927770085
GSE40272	X7651	89941	RHOT2	-0,927678118
GSE40272	X9237	8313	AXIN2	-0,927535412
GSE40272	X394	4035	LRP1	-0,927521142
GSE40272	X2426	54982	CLN6	-0,926811311
GSE40272	X41696	130497	OSR1	-0,925721987
GSE40272	X7690	84164	ASCC2	-0,925464588
GSE40272	X17238	10509	SEMA4B	-0,925386364
GSE40272	X14979	57406	ABHD6	-0,924476216
GSE40272	X9526	221336	BEND6	-0,924196089
GSE40272	X9704	909	CD1A	-0,922769027
GSE40272	X39308	80024	SLC24A6	-0,922766913
GSE40272	X9721	29979	UBQLN1	-0,921852008
GSE40272	X12908	25961	NUDT13	-0,921721987
GSE40272	X18920	54991	C1orf159	-0,921382135
GSE40272	X12453	57186	RALGAPA2	-0,920976744
GSE40272	X37990	2996	GYPE	-0,920803383
GSE40272	X37664	7094	TLN1	-0,920749471
GSE40272	X30602	84767	SPRYD5	-0,919964588
GSE40272	X28489	92359	CRB3	-0,919621036
GSE40272	X28644	10072	DPP3	-0,919495243
GSE40272	X38441	4035	LRP1	-0,918272727
GSE40272	X44337	442365	LOC442365	-0,916539112
GSE40272	X25903	3679	ITGA7	-0,916506871
GSE40272	X41602	54815	GATAD2A	-0,915821353
GSE40272	X800	80144	FRAS1	-0,915496829
GSE40272	X16028	11252	PACSIN2	-0,915483615
GSE40272	X9309	89958	C9orf140	-0,915336152
GSE40272	X19424	116969	ART5	-0,914698732
GSE40272	X38590	1938	EEF2	-0,914488901
GSE40272	X5460	80144	FRAS1	-0,913617865
GSE40272	X27545	6932	TCF7	-0,912867865
GSE40272	X37938	56114	PCDHGA1	-0,912595666
GSE40272	X17575	389310	LOC389310	-0,911758457
GSE40272	X8907	125058	TBC1D16	-0,911239429
GSE40272	X37250	378938	MALAT1	-0,911214059

GSE40272	X16047	2275	FHL3	-0,9105037
GSE40272	X13776	79929	MAP6D1	-0,910332981
GSE40272	X2385	120071	GYLTL1B	-0,910326638
GSE40272	X6867	10585	POMT1	-0,910320825
GSE40272	X20032	5430	POLR2A	-0,910197674
GSE40272	X36463	51816	CECR1	-0,909770085
GSE40272	X39913	91807	MYLK3	-0,908882664
GSE40272	X43878	6928	HNF1B	-0,908692389
GSE40272	X2661	10072	DPP3	-0,907948203
GSE40272	X26444	79763	ISOC2	-0,907373679
GSE40272	X38953	10214	SSX3	-0,90667759
GSE40272	X19543	95	ACY1	-0,906370507
GSE40272	X13046	1956	EGFR	-0,905645877
GSE40272	X16583	399979	SNX19	-0,905099894
GSE40272	X23254	375686	SPATC1	-0,905090381
GSE40272	X20920	80144	FRAS1	-0,905088266
GSE40272	X12600	7329	UBE2I	-0,904613636
GSE40272	X36717	23218	NBEAL2	-0,904003171
GSE40272	X37513	4035	LRP1	-0,902794397
GSE40272	X39680	51444	RNF138	-0,902449789
GSE40272	X5787	152831	KLB	-0,902158034
GSE40272	X8402	92979	MARCH9	-0,90126797
GSE40272	X2674	4013	VWA5A	-0,901143235
GSE40272	X15037	441682	LOC441682	-0,900937104
GSE40272	X30978	347734	SLC35B2	-0,900469345
GSE40272	X31655	55193	PBRM1	-0,900309725
GSE40272	X38177	2705	GJB1	-0,899660677
GSE40272	X21244	23303	KIF13B	-0,899330867
GSE40272	X40761	284297	SSC5D	-0,899271142
GSE40272	X42345	79166	LILRP2	-0,899131078
GSE40272	X20299	5157	PDGFRL	-0,898915962
GSE40272	X34595	80144	FRAS1	-0,898903277
GSE40272	X41058	3603	IL16	-0,89855444
GSE40272	X16043	56999	ADAMTS9	-0,89835518
GSE40272	X14885	29882	ANAPC2	-0,897640063
GSE40272	X11645	1890	TYMP	-0,897636364
GSE40272	X23743	4035	LRP1	-0,896689218
GSE40272	X30208	55160	ARHGEF10L	-0,895760042
GSE40272	X21634	5925	RB1	-0,895210888
GSE40272	X24272	11156	PTP4A3	-0,894827696
GSE40272	X31910	114899	C1QTNF3	-0,893318182
GSE40272	X29901	2296	FOXC1	-0,892715645
GSE40272	X36082	4833	NME4	-0,892541226
GSE40272	X13609	9922	IQSEC1	-0,892276427
GSE40272	X24375	51684	SUFU	-0,892241543
GSE40272	X38779	755	C21orf2	-0,891899577
GSE40272	X5582	83692	CD99L2	-0,891745772
GSE40272	X19195	84720	PIGO	-0,891117865
GSE40272	X33942	9618	TRAF4	-0,89101797
GSE40272	X11280	64753	CCDC136	-0,89096723
GSE40272	X31579	54976	C20orf27	-0,890914905
GSE40272	X12608	4035	LRP1	-0,890906977
GSE40272	X3940	3909	LAMA3	-0,890840909

GSE40272	X2648	57507	ZNF608	-0,889144292
GSE40272	X26806	5600	MAPK11	-0,888779598
GSE40272	X4225	442552	LOC442552	-0,888418076
GSE40272	X26903	675	BRCA2	-0,888339852
GSE40272	X7652	3909	LAMA3	-0,888102537
GSE40272	X31857	4163	MCC	-0,887984144
GSE40272	X43046	613	BCR	-0,887676533
GSE40272	X40297	4035	LRP1	-0,887217759
GSE40272	X5033	4035	LRP1	-0,886907505
GSE40272	X3177	4035	LRP1	-0,886179175
GSE40272	X17292	5089	PBX2	-0,886092495
GSE40272	X9296	25970	SH2B1	-0,885141649
GSE40272	X13737	869	CBLN1	-0,884926533
GSE40272	X43027	54756	IL17RD	-0,884736258
GSE40272	X14096	9894	TELO2	-0,884406977
GSE40272	X2398	1509	CTSD	-0,884157505
GSE40272	X17531	51599	LSR	-0,883981501
GSE40272	X20564	390777	LOC390777	-0,88395666
GSE40272	X40913	5819	PVRL2	-0,883671776
GSE40272	X6656	51540	SCLY	-0,88347093
GSE40272	X3790	25920	COBRA1	-0,883133192
GSE40272	X9551	6932	TCF7	-0,882385307
GSE40272	X28208	1087	CEACAM7	-0,881942389
GSE40272	X39780	10072	DPP3	-0,881160148
GSE40272	X38932	54434	SSH1	-0,880581395
GSE40272	X35413	389655	RPL29P19	-0,880448732
GSE40272	X43035	2747	GLUD2	-0,879489429
GSE40272	X6277	163732	CITED4	-0,879188161
GSE40272	X39239	51776	ZAK	-0,878859937
GSE40272	X27081	78995	C17orf53	-0,878596195
GSE40272	X1660	7798	LUZP1	-0,878522727
GSE40272	X41157	5979	RET	-0,878215116
GSE40272	X15830	340348	TSPAN33	-0,878109937
GSE40272	X15011	64781	CERK	-0,87754704
GSE40272	X1321	4035	LRP1	-0,877495772
GSE40272	X27162	29988	SLC2A8	-0,877385835
GSE40272	X33010	5339	PLEC	-0,87730814
GSE40272	X28382	2934	GSN	-0,876902748
GSE40272	X40919	255031	FLJ35390	-0,876800211
GSE40272	X29825	339666	LOC339666	-0,876595666
GSE40272	X12606	60	ACTB	-0,876408034
GSE40272	X37757	4137	MAPT	-0,875981501
GSE40272	X39520	7094	TLN1	-0,875820296
GSE40272	X15722	28996	HIPK2	-0,875346195
GSE40272	X26235	58525	WIZ	-0,875150634
GSE40272	X18175	4035	LRP1	-0,874958245
GSE40272	X39011	54458	PRR13	-0,874720402
GSE40272	X9991	55731	C17orf63	-0,874121564
GSE40272	X9674	4035	LRP1	-0,874098837
GSE40272	X11679	1938	EEF2	-0,874042812
GSE40272	X16282	398	ARHGDIG	-0,874016385
GSE40272	X12205	57175	CORO1B	-0,873354651
GSE40272	X4255	2934	GSN	-0,873007928

GSE40272	X37253	10200	MPHOSPH6	-0,872917019
GSE40272	X16970	2874	GPS2	-0,872707188
GSE40272	X2494	1185	CLCN6	-0,872377907
GSE40272	X4725	64006	ERVK-6	-0,87194926
GSE40272	X23028	6468	FBXW4	-0,871743129
GSE40272	X42530	54880	BCOR	-0,870971987
GSE40272	X32303	6096	RORB	-0,870948203
GSE40272	X33546	3916	LAMP1	-0,870761628
GSE40272	X11245	9827	RGP1	-0,870245772
GSE40272	X42128	23481	PES1	-0,870239429
GSE40272	X18151	26855	RNU2-2	-0,869334038
GSE40272	X29161	4035	LRP1	-0,869195032
GSE40272	X29237	389390	LOC389390	-0,868931818
GSE40272	X8230	81876	RAB1B	-0,868459302
GSE40272	X3178	4035	LRP1	-0,868399049
GSE40272	X36732	3145	HMBS	-0,868280655
GSE40272	X16318	4035	LRP1	-0,868262685
GSE40272	X393	7094	TLN1	-0,868212474
GSE40272	X19384	10439	OLFM1	-0,867906977
GSE40272	X15508	389722	CNTNAP3B-LIKE	-0,867713531
GSE40272	X20132	84904	C9orf100	-0,867707717
GSE40272	X24888	442069	LOC442069	-0,867700317
GSE40272	X13876	440703	LOC440703	-0,867575581
GSE40272	X16605	55823	VPS11	-0,867560782
GSE40272	X12868	54884	RETSAT	-0,867077167
GSE40272	X26330	51005	AMDHD2	-0,866639535
GSE40272	X2227	146760	RTN4RL1	-0,866426004
GSE40272	X28386	441804	LOC441804	-0,86635148
GSE40272	X640	2010	EMD	-0,866317653
GSE40272	X14314	4035	LRP1	-0,865705603
GSE40272	X8143	5929	RBBP5	-0,865697674
GSE40272	X38855	1488	CTBP2	-0,865223573
GSE40272	X25684	80144	FRAS1	-0,864298097
GSE40272	X43146	5339	PLEC	-0,864146934
GSE40272	X6826	1889	ECE1	-0,863846723
GSE40272	X12704	65009	NDRG4	-0,863766913
GSE40272	X7296	116984	ARAP2	-0,863038584
GSE40272	X17166	30819	KCNIP2	-0,862510042
GSE40272	X4074	10169	SERF2	-0,86234778
GSE40272	X38575	260429	PRSS33	-0,862345666
GSE40272	X35914	374875	HSD11B1L	-0,862168605
GSE40272	X6031	81562	LMAN2L	-0,861815011
GSE40272	X8757	5673	PSG5	-0,861632664
GSE40272	X24765	80199	FUZ	-0,861245772
GSE40272	X10899	90313	TP53I13	-0,861216173
GSE40272	X12165	9238	TBRG4	-0,861194503
GSE40272	X17606	375295	LOC375295	-0,860717759
GSE40272	X18787	29995	LMCD1	-0,860600951
GSE40272	X12210	389636	LOC389636	-0,860570296
GSE40272	X13972	27097	TAF5L	-0,860512685
GSE40272	X14313	4035	LRP1	-0,86032981
GSE40272	X35805	1509	CTSD	-0,859695032
GSE40272	X33576	51451	LCMT1	-0,859270085

GSE40272	X32840	3909	LAMA3	-0,859257928
GSE40272	X42013	10555	AGPAT2	-0,858853594
GSE40272	X22295	55721	IQCC	-0,858817125
GSE40272	X1322	4035	LRP1	-0,858637421
GSE40272	X34146	388823	LOC388823	-0,858016385
GSE40272	X10352	28991	COMMD5	-0,857758985
GSE40272	X6026	2889	RAPGEF1	-0,857451374
GSE40272	X23124	29903	CCDC106	-0,857335095
GSE40272	X11195	55145	THAP1	-0,857298626
GSE40272	X20949	4056	LTC4S	-0,857252114
GSE40272	X41650	26262	TSPAN17	-0,85620296
GSE40272	X41673	2067	ERCC1	-0,855960359
GSE40272	X32051	80144	FRAS1	-0,855672304
GSE40272	X42089	388515	LOC388515	-0,854941332
GSE40272	X13386	4035	LRP1	-0,854764799
GSE40272	X14774	4069	LYZ	-0,854748414
GSE40272	X38122	84885	ZDHHC12	-0,854360994
GSE40272	X5962	4035	LRP1	-0,853825581
GSE40272	X42095	140775	SMCR8	-0,85370296
GSE40272	X23097	84222	TMEM191A	-0,853593552
GSE40272	X33128	1066	CES1	-0,852813425
GSE40272	X806	6453	ITSN1	-0,852281184
GSE40272	X41483	442327	LOC442327	-0,852092495
GSE40272	X43047	80144	FRAS1	-0,852077696
GSE40272	X5915	5781	PTPN11	-0,851935518
GSE40272	X21819	9229	DLGAP1	-0,851913319
GSE40272	X16727	391705	LOC391705	-0,851866279
GSE40272	X39126	8061	FOSL1	-0,851707717
GSE40272	X23347	6901	TAZ	-0,851349894
GSE40272	X12607	4035	LRP1	-0,851215645
GSE40272	X14657	348932	SLC6A18	-0,850945032
GSE40272	X34711	80789	INTS5	-0,850919133
GSE40272	X11922	117584	RFFL	-0,850906977
GSE40272	X18711	4242	MFNG	-0,850886892
GSE40272	X20646	148490	LOC148490	-0,850561311
GSE40272	X44120	9150	CTDP1	-0,850382135
GSE40272	X12169	23598	PATZ1	-0,850330338
GSE40272	X42304	4035	LRP1	-0,849587738
GSE40272	X5669	64072	CDH23	-0,849147463
GSE40272	X15816	254773	LYG2	-0,849095666
GSE40272	X23780	80144	FRAS1	-0,84855074
GSE40272	X12947	4733	DRG1	-0,848233615
GSE40272	X17733	6405	SEMA3F	-0,848073467
GSE40272	X35808	7094	TLN1	-0,84769926
GSE40272	X11939	6872	TAF1	-0,847647463
GSE40272	X24696	116985	ARAP1	-0,847331395
GSE40272	X17802	5589	PRKCSH	-0,847145349
GSE40272	X43966	10360	NPM3	-0,847049683
GSE40272	X34120	9973	CCS	-0,846955603
GSE40272	X7199	63894	VIPAR	-0,846926533
GSE40272	X23553	55700	MAP7D1	-0,846838266
GSE40272	X33778	10421	CD2BP2	-0,846629493
GSE40272	X11985	63027	SLC22A23	-0,846437632

GSE40272	X28008	7464	CORO2A	-0,845959302
GSE40272	X26527	2934	GSN	-0,845821353
GSE40272	X335	22933	SIRT2	-0,845233087
GSE40272	X31806	64854	USP46	-0,844727273
GSE40272	X542	5430	POLR2A	-0,844400634
GSE40272	X34649	26523	EIF2C1	-0,844331924
GSE40272	X36733	1938	EEF2	-0,844146406
GSE40272	X9414	83549	UCK1	-0,843683932
GSE40272	X11530	4035	LRP1	-0,842959831
GSE40272	X26508	1445	CSK	-0,842571353
GSE40272	X35427	311	ANXA11	-0,842454017
GSE40272	X38709	2703	GJA8	-0,842216173
GSE40272	X36492	286077	FAM83H	-0,842022199
GSE40272	X44442	92659	LOC92659	-0,842012685
GSE40272	X44285	8612	PPAP2C	-0,841931818
GSE40272	X23467	10840	ALDH1L1	-0,840539112
GSE40272	X13502	340947	LOC340947	-0,840285412
GSE40272	X19024	23476	BRD4	-0,840264271
GSE40272	X37981	26088	GGA1	-0,840168076
GSE40272	X4572	51257	MARCH2	-0,839755285
GSE40272	X1462	284361	C19orf63	-0,839570825
GSE40272	X11299	84264	HAGHL	-0,839538584
GSE40272	X44009	4035	LRP1	-0,839345137
GSE40272	X13683	20	ABCA2	-0,8390037
GSE40272	X17279	63943	FKBPL	-0,838993129
GSE40272	X36585	4035	LRP1	-0,838883721
GSE40272	X41691	50512	PODXL2	-0,838862579
GSE40272	X8322	11282	MGAT4B	-0,838791755
GSE40272	X30559	10220	GDF11	-0,838745772
GSE40272	X37653	2534	FYN	-0,838578753
GSE40272	X37893	92799	SHKBP1	-0,838562368
GSE40272	X461	5214	PFKP	-0,837494715
GSE40272	X12919	339779	C2orf53	-0,837465645
GSE40272	X15950	56850	GRIPAP1	-0,837285941
GSE40272	X31166	1938	EEF2	-0,837283827
GSE40272	X41664	27239	GPR162	-0,837021142
GSE40272	X1606	388507	ZNF788	-0,836680233
GSE40272	X4870	64601	VPS16	-0,836664376
GSE40272	X28238	3145	HMBS	-0,83655074
GSE40272	X1153	23224	SYNE2	-0,835332981
GSE40272	X11171	3757	KCNH2	-0,834730973
GSE40272	X34434	55313	CPPED1	-0,834557082
GSE40272	X37372	55735	DNAJC11	-0,833815539
GSE40272	X2208	51548	SIRT6	-0,833447674
GSE40272	X37173	441742	LOC441742	-0,833406448
GSE40272	X43622	3145	HMBS	-0,833341438
GSE40272	X20392	6927	HNF1A	-0,833093023
GSE40272	X28483	131973	LOC131973	-0,832645349
GSE40272	X21812	1499	CTNNB1	-0,831663319
GSE40272	X1471	4035	LRP1	-0,831530127
GSE40272	X20183	11076	TPPP	-0,831438161
GSE40272	X15810	10554	AGPAT1	-0,830204017
GSE40272	X35154	79960	PHF17	-0,830124207

GSE40272	X23184	140807	KRT72	-0,829966702
GSE40272	X30790	10072	DPP3	-0,82994926
GSE40272	X38906	6452	SH3BP2	-0,829930761
GSE40272	X33943	84993	UBL7	-0,829670719
GSE40272	X7582	388552	BLOC1S3	-0,829291226
GSE40272	X8993	23216	TBC1D1	-0,829158562
GSE40272	X26455	9993	DGCR2	-0,828733087
GSE40272	X21462	4163	MCC	-0,828591966
GSE40272	X14083	10807	SDCCAG3	-0,828478858
GSE40272	X31017	4035	LRP1	-0,828379493
GSE40272	X33603	8844	KSR1	-0,828147992
GSE40272	X22436	1983	EIF5	-0,828139535
GSE40272	X12858	56999	ADAMTS9	-0,827966173
GSE40272	X25260	116729	DYSFIP1	-0,827559725
GSE40272	X5184	7094	TLN1	-0,827531712
GSE40272	X272	26577	PCOLCE2	-0,827488372
GSE40272	X14020	2067	ERCC1	-0,826675476
GSE40272	X1081	951	CD37	-0,826640592
GSE40272	X41488	775	CACNA1C	-0,826630021
GSE40272	X42425	4299	AFF1	-0,826407505
GSE40272	X36484	3156	HMGCR	-0,826376321
GSE40272	X24652	79672	FN3KRP	-0,825811839
GSE40272	X9931	1307	COL16A1	-0,825415433
GSE40272	X18848	2802	GOLGA3	-0,825228858
GSE40272	X25900	10922	FASTK	-0,825114693
GSE40272	X11979	10994	ILVBL	-0,825084567
GSE40272	X7671	7074	TIAM1	-0,824967759
GSE40272	X33512	84364	ARFGAP2	-0,824786469
GSE40272	X25205	6550	SLC9A3	-0,824553911
GSE40272	X2895	119764	OR4X2	-0,824371564
GSE40272	X22150	10072	DPP3	-0,823878436
GSE40272	X35246	5625	PRODH	-0,823806554
GSE40272	X20034	441404	LOC441404	-0,82307241
GSE40272	X22814	1938	EEF2	-0,823055497
GSE40272	X40766	80727	TTYH3	-0,822198732
GSE40272	X12468	284904	SEC14L4	-0,822136892
GSE40272	X21785	780	DDR1	-0,821475687
GSE40272	X2614	25803	SPDEF	-0,820855708
GSE40272	X3523	389816	LRRC26	-0,820612051
GSE40272	X34697	55168	MRPS18A	-0,820524313
GSE40272	X28875	388474	RPL7AP66	-0,820067653
GSE40272	X25860	6799	SULT1A2	-0,81979334
GSE40272	X20985	5296	PIK3R2	-0,819698203
GSE40272	X30071	8501	SLC43A1	-0,81954704
GSE40272	X31441	596	BCL2	-0,819521142
GSE40272	X30156	4782	NFIC	-0,819510571
GSE40272	X34257	64847	SPATA20	-0,819212474
GSE40272	X24230	2972	BRF1	-0,818977273
GSE40272	X7457	30813	VSX1	-0,818678118
GSE40272	X15327	221687	RNF182	-0,818554968
GSE40272	X13764	10910	SUGT1	-0,818371036
GSE40272	X16621	51599	LSR	-0,818271142
GSE40272	X18833	22877	MLXIP	-0,817512685

GSE40272	X23770	78987	CRELD1	-0,816836681
GSE40272	X16683	347265	KRT8P11	-0,816739958
GSE40272	X22757	4297	MLL	-0,816577696
GSE40272	X24971	3909	LAMA3	-0,816301268
GSE40272	X12336	2874	GPS2	-0,81613055
GSE40272	X18978	2055	CLN8	-0,815899577
GSE40272	X34976	23498	HAAO	-0,814654863
GSE40272	X20687	22885	ABLIM3	-0,814479387
GSE40272	X34561	444	ASPH	-0,814384778
GSE40272	X37134	147646	LOC147646	-0,814001586
GSE40272	X34659	1855	DVL1	-0,813370507
GSE40272	X21490	382	ARF6	-0,81215592
GSE40272	X41813	3069	HDLBP	-0,812141649
GSE40272	X3615	65990	FAM173A	-0,812112579
GSE40272	X43378	4046	LSP1	-0,811770085
GSE40272	X541	2934	GSN	-0,811734144
GSE40272	X35151	5585	PKN1	-0,811161205
GSE40272	X6111	1938	EEF2	-0,810849894
GSE40272	X30927	1468	SLC25A10	-0,810717759
GSE40272	X10847	9817	KEAP1	-0,81015592
GSE40272	X816	1307	COL16A1	-0,809451903
GSE40272	X35368	54880	BCOR	-0,809264271
GSE40272	X10058	4123	MAN2C1	-0,808829281
GSE40272	X12457	4035	LRP1	-0,808356765
GSE40272	X32282	388760	LOC388760	-0,807798097
GSE40272	X9173	30827	CXXC1	-0,807293869
GSE40272	X7188	1104	RCC1	-0,806276427
GSE40272	X15031	56342	PPAN	-0,806248414
GSE40272	X19924	256880	LOC256880	-0,805642178
GSE40272	X14680	1455	CSNK1G2	-0,805476744
GSE40272	X9722	64924	SLC30A5	-0,805196089
GSE40272	X15391	2934	GSN	-0,805139535
GSE40272	X23451	8864	PER2	-0,804727273
GSE40272	X3326	1938	EEF2	-0,80470666
GSE40272	X21872	90993	CREB3L1	-0,804319239
GSE40272	X13152	80305	TRABD	-0,80384778
GSE40272	X37490	10421	CD2BP2	-0,803730444
GSE40272	X27556	91695	RRP7B	-0,803253171
GSE40272	X16687	440368	LOC440368	-0,803045455
GSE40272	X7433	23308	ICOSLG	-0,802764799
GSE40272	X39003	9967	THRAP3	-0,802716173
GSE40272	X2639	6499	SKIV2L	-0,80232611
GSE40272	X38344	9441	MED26	-0,801779598
GSE40272	X19639	57224	NHSL1	-0,801378964
GSE40272	X2250	4035	LRP1	-0,801300211
GSE40272	X12839	8864	PER2	-0,801146406
GSE40272	X7810	2582	GALE	-0,800486786
GSE40272	X26726	3623	INHA	-0,800364165
GSE40272	X2317	26470	SEZ6L2	-0,799714059
GSE40272	X108	54498	SMOX	-0,799328753
GSE40272	X31470	57147	SCYL3	-0,799033298
GSE40272	X17245	4035	LRP1	-0,79902167
GSE40272	X10020	64428	NARFL	-0,798765328

GSE40272	X35847	10994	ILVBL	-0,798637421
GSE40272	X10083	5433	POLR2D	-0,798342495
GSE40272	X39609	10554	AGPAT1	-0,798187632
GSE40272	X13735	57644	MYH7B	-0,79801797
GSE40272	X31913	10588	MTHFS	-0,797003171
GSE40272	X13236	6484	ST3GAL4	-0,796488901
GSE40272	X14518	339448	C1orf174	-0,795782241
GSE40272	X33949	5430	POLR2A	-0,795515856
GSE40272	X42312	401578	LOC401578	-0,795352008
GSE40272	X24777	79144	PPDPF	-0,794026956
GSE40272	X19316	6461	SHB	-0,793994715
GSE40272	X31814	80144	FRAS1	-0,793702431
GSE40272	X11655	3614	IMPDH1	-0,793294926
GSE40272	X29276	3340	NDST1	-0,792885835
GSE40272	X6639	153090	DAB2IP	-0,792783298
GSE40272	X22535	7551	ZNF3	-0,79242759
GSE40272	X12012	79932	KIAA0319L	-0,792307611
GSE40272	X30866	3993	LLGL2	-0,79178277
GSE40272	X22324	80325	ABTB1	-0,791696089
GSE40272	X20018	7205	TRIP6	-0,791618922
GSE40272	X33868	57655	GRAMD1A	-0,791473044
GSE40272	X33016	1889	ECE1	-0,791404334
GSE40272	X33914	10749	KIF1C	-0,791067653
GSE40272	X41914	140801	RPL10L	-0,790639006
GSE40272	X4287	84766	EFCAB4B	-0,790512685
GSE40272	X38209	3300	DNAJB2	-0,790413319
GSE40272	X8197	29781	NCAPH2	-0,790224101
GSE40272	X15389	1938	EEF2	-0,790208774
GSE40272	X42854	5873	RAB27A	-0,790001057
GSE40272	X14440	353333	KRTAP10-10	-0,789405391
GSE40272	X24766	25915	NDUFAF3	-0,789158034
GSE40272	X33057	23678	SGK3	-0,788739958
GSE40272	X27981	2593	GAMT	-0,788382664
GSE40272	X20123	2067	ERCC1	-0,788222516
GSE40272	X29592	3707	ITPKB	-0,787930761
GSE40272	X5968	310	ANXA7	-0,7875074
GSE40272	X38562	26819	SNORD32A	-0,787410677
GSE40272	X5761	3425	IDUA	-0,787040169
GSE40272	X35391	89882	TPD52L3	-0,786892706
GSE40272	X13802	10882	C1QL1	-0,786714059
GSE40272	X41523	5590	PRKCZ	-0,785840381
GSE40272	X16026	8864	PER2	-0,785638478
GSE40272	X17146	84557	MAP1LC3A	-0,785622093
GSE40272	X21623	80014	WWC2	-0,785265328
GSE40272	X28207	389049	LOC389049	-0,78515592
GSE40272	X19178	151742	PPM1L	-0,785111522
GSE40272	X15392	4035	LRP1	-0,784878436
GSE40272	X33488	80256	KIAA1539	-0,784816068
GSE40272	X22634	390121	LOC390121	-0,783975687
GSE40272	X31715	818	CAMK2G	-0,783947146
GSE40272	X5253	23417	MLYCD	-0,783913848
GSE40272	X17224	3216	HOXB6	-0,783715116
GSE40272	X12598	200186	CRTC2	-0,783692389

GSE40272	X9810	54475	NLE1	-0,783150634
GSE40272	X24043	7260	TSSC1	-0,782780655
GSE40272	X30468	5992	RFX4	-0,782371036
GSE40272	X34696	10994	ILVBL	-0,782367865
GSE40272	X20514	6132	RPL8	-0,782216173
GSE40272	X12458	4035	LRP1	-0,782189746
GSE40272	X42948	55898	UNC45A	-0,782167548
GSE40272	X101	6472	SHMT2	-0,781571353
GSE40272	X37287	51734	SEPX1	-0,781513214
GSE40272	X14456	2934	GSN	-0,781408034
GSE40272	X15102	23095	KIF1B	-0,781368393
GSE40272	X10918	1384	CRAT	-0,780950846
GSE40272	X19540	55851	PSENEN	-0,780439746
GSE40272	X28914	388906	LOC388906	-0,780347252
GSE40272	X6541	27085	MTBP	-0,779962474
GSE40272	X24139	399933	LOC399933	-0,779614165
GSE40272	X34514	10527	IPO7	-0,779469345
GSE40272	X32192	8085	MLL2	-0,779376321
GSE40272	X20030	2934	GSN	-0,779340909
GSE40272	X9823	2934	GSN	-0,779256871
GSE40272	X32188	80144	FRAS1	-0,779184461
GSE40272	X3887	22838	RNF44	-0,77862685
GSE40272	X5672	55898	UNC45A	-0,778618393
GSE40272	X42396	7335	UBE2V1	-0,778354651
GSE40272	X31692	164	AP1G1	-0,777937104
GSE40272	X4278	79759	ZNF668	-0,777893235
GSE40272	X8892	3145	HMBS	-0,77778277
GSE40272	X4995	23095	KIF1B	-0,777768499
GSE40272	X22825	80856	KIAA1715	-0,777651691
GSE40272	X4701	8864	PER2	-0,777381078
GSE40272	X7254	10907	TXNL4A	-0,777248414
GSE40272	X21738	4035	LRP1	-0,776814482
GSE40272	X19758	1459	CSNK2A2	-0,7767426
GSE40272	X17338	84957	RELT	-0,776613108
GSE40272	X5866	129303	TMEM150A	-0,776458774
GSE40272	X34255	23181	DIP2A	-0,776413319
GSE40272	X29116	4791	NFKB2	-0,776306025
GSE40272	X23368	81	ACTN4	-0,776151691
GSE40272	X34161	389458	LOC389458	-0,776036998
GSE40272	X17248	5430	POLR2A	-0,775466173
GSE40272	X5795	5297	PI4KA	-0,775162262
GSE40272	X9442	23001	WDFY3	-0,775013742
GSE40272	X37580	8425	LTBP4	-0,774634249
GSE40272	X21299	440036	LOC440036	-0,77463055
GSE40272	X16535	5830	PEX5	-0,774556554
GSE40272	X11878	347732	CATSPER3	-0,774481501
GSE40272	X23835	126119	JOSD2	-0,774314482
GSE40272	X29345	5802	PTPRS	-0,774204017
GSE40272	X28040	349633	C11orf34	-0,773842495
GSE40272	X20950	64601	VPS16	-0,773709831
GSE40272	X11333	22870	PPP6R1	-0,773653805
GSE40272	X30994	89872	AQP10	-0,773555497
GSE40272	X36227	8890	EIF2B4	-0,773511099

GSE40272	X19385	84222	TMEM191A	-0,7735
GSE40272	X11697	10999	SLC27A4	-0,77325
GSE40272	X8281	6917	TCEA1	-0,772921776
GSE40272	X13089	1627	DBN1	-0,772787526
GSE40272	X6209	64946	CENPH	-0,772672833
GSE40272	X14462	1938	EEF2	-0,772223573
GSE40272	X30099	27320	TNRC18B	-0,772070296
GSE40272	X22918	402280	LOC402280	-0,771993129
GSE40272	X1575	441915	LOC441915	-0,771991015
GSE40272	X27878	5792	PTPRF	-0,77145666
GSE40272	X40427	53947	A4GALT	-0,771381078
GSE40272	X13385	7094	TLN1	-0,771274841
GSE40272	X20810	4035	LRP1	-0,771156448
GSE40272	X11678	5430	POLR2A	-0,770969873
GSE40272	X9313	118426	LOH12CR1	-0,7699963
GSE40272	X28334	50674	NEUROG3	-0,769850951
GSE40272	X23099	3155	HMGCL	-0,769645877
GSE40272	X21188	706	TSPO	-0,769595137
GSE40272	X41153	3268	AGFG2	-0,769275899
GSE40272	X8675	1855	DVL1	-0,769075053
GSE40272	X12200	23135	KDM6B	-0,768906448
GSE40272	X9744	116984	ARAP2	-0,76854334
GSE40272	X2390	10312	TCIRG1	-0,767922833
GSE40272	X17354	387643	LOC387643	-0,767907505
GSE40272	X10268	8720	MBTPS1	-0,767241543
GSE40272	X17989	23556	PIGN	-0,767046512
GSE40272	X15652	5680	PSG11	-0,766930761
GSE40272	X17306	84524	ZC3H8	-0,766774313
GSE40272	X39518	4035	LRP1	-0,766719345
GSE40272	X37350	55671	SMEK1	-0,766634249
GSE40272	X1080	10552	ARPC1A	-0,766378436
GSE40272	X35301	441033	LOC441033	-0,76583351
GSE40272	X3110	40	ACCN1	-0,765502114
GSE40272	X5800	23163	GGA3	-0,765502114
GSE40272	X37649	283899	INO80E	-0,765223044
GSE40272	X27991	22906	TRAK1	-0,765162262
GSE40272	X10898	23215	PRRC2C	-0,764638478
GSE40272	X14777	2629	GBA	-0,763567125
GSE40272	X41330	54503	ZDHHC13	-0,763098309
GSE40272	X22409	170958	ZNF525	-0,763004228
GSE40272	X25562	79006	METRN	-0,762422833
GSE40272	X40998	6300	MAPK12	-0,762348309
GSE40272	X15874	9770	RASSF2	-0,762273784
GSE40272	X40430	26608	TBL2	-0,761338266
GSE40272	X1561	6687	SPG7	-0,760966173
GSE40272	X41336	5051	PAFAH2	-0,760854123
GSE40272	X16864	81628	TSC22D4	-0,7607426
GSE40272	X28197	64794	DDX31	-0,76069556
GSE40272	X2518	375775	PNPLA7	-0,760604651
GSE40272	X5487	84971	ATG4D	-0,76057241
GSE40272	X24661	6525	SMTN	-0,760266913
GSE40272	X12502	441054	C4orf47	-0,760215645
GSE40272	X38199	9583	ENTPD4	-0,759943446

GSE40272	X38396	54815	GATAD2A	-0,759791226
GSE40272	X30228	64400	AKTIP	-0,759669662
GSE40272	X6208	9344	TAOK2	-0,759615222
GSE40272	X43387	11060	WWP2	-0,759489958
GSE40272	X29942	392539	LOC392539	-0,759029598
GSE40272	X6888	83985	SPNS1	-0,758852537
GSE40272	X34880	4035	LRP1	-0,758781712
GSE40272	X22744	10629	TAF6L	-0,758762685
GSE40272	X17954	51011	FAHD2A	-0,758475159
GSE40272	X8745	4035	LRP1	-0,758317125
GSE40272	X33952	7094	TLN1	-0,757669662
GSE40272	X39051	27434	POLM	-0,75758351
GSE40272	X22014	91039	DPP9	-0,757494715
GSE40272	X4868	55227	LRRC1	-0,757216702
GSE40272	X42574	286102	TMED10P1	-0,756369979
GSE40272	X19108	219293	ATAD3C	-0,755715116
GSE40272	X2427	10972	TMED10	-0,755483087
GSE40272	X7481	23224	SYNE2	-0,755252114
GSE40272	X1573	57085	AGTRAP	-0,754921776
GSE40272	X42450	57662	CAMSAP3	-0,754514799
GSE40272	X35149	203074	PRSS55	-0,754513742
GSE40272	X20960	4035	LRP1	-0,754179175
GSE40272	X42936	55362	TMEM63B	-0,754096195
GSE40272	X20009	23786	BCL2L13	-0,753955603
GSE40272	X23312	27	ABL2	-0,753895349
GSE40272	X19551	203228	C9orf72	-0,75387315
GSE40272	X23611	6649	SOD3	-0,753839323
GSE40272	X17641	8864	PER2	-0,753789641
GSE40272	X41465	4052	LTBP1	-0,753192918
GSE40272	X8413	8864	PER2	-0,752968816
GSE40272	X16976	54904	WHSC1L1	-0,752522199
GSE40272	X33862	171019	ADAMTS19	-0,75243129
GSE40272	X7628	3191	HNRNPL	-0,752233087
GSE40272	X21886	1509	CTSD	-0,751363108
GSE40272	X19562	84065	TMEM222	-0,751187104
GSE40272	X16652	6041	RNASEL	-0,751045983
GSE40272	X42566	3268	AGFG2	-0,750266385
GSE40272	X37479	80222	TARS2	-0,749997886
GSE40272	X5928	23001	WDFY3	-0,749896406
GSE40272	X29947	3386	ICAM4	-0,749642706
GSE40272	X5490	5534	PPP3R1	-0,749605708
GSE40272	X28552	6502	SKP2	-0,748938689
GSE40272	X5639	26809	SNORD42A	-0,748704017
GSE40272	X42833	6853	SYN1	-0,748370507
GSE40272	X39731	10916	MAGED2	-0,748253171
GSE40272	X16718	84132	USP42	-0,747958774
GSE40272	X28406	55079	FEZF2	-0,747538584
GSE40272	X18914	1956	EGFR	-0,747444503
GSE40272	X5287	442407	LOC442407	-0,747186575
GSE40272	X5182	4035	LRP1	-0,747156448
GSE40272	X35041	377711	LOC377711	-0,74671723
GSE40272	X35800	6899	TBX1	-0,746709831
GSE40272	X38322	207	AKT1	-0,746683404

GSE40272	X32207	401848	LOC401848	-0,746642178
GSE40272	X10742	57060	PCBP4	-0,746615222
GSE40272	X41225	4035	LRP1	-0,746542283
GSE40272	X14879	80256	KIAA1539	-0,746424947
GSE40272	X2211	90627	STARD13	-0,746138478
GSE40272	X453	140460	ASB7	-0,746095666
GSE40272	X22816	5430	POLR2A	-0,745723044
GSE40272	X25317	112950	MED8	-0,745588795
GSE40272	X829	37	ACADVL	-0,745500529
GSE40272	X30435	2778	GNAS	-0,745292283
GSE40272	X20727	30832	ZNF354C	-0,745291226
GSE40272	X11407	84501	SPIRE2	-0,745235201
GSE40272	X12552	66004	LYNX1	-0,745034355
GSE40272	X24637	142678	MIB2	-0,744929175
GSE40272	X8994	439952	LOC439952	-0,744774841
GSE40272	X13730	9479	MAPK8IP1	-0,744352008
GSE40272	X6232	1855	DVL1	-0,744311311
GSE40272	X22050	23299	BICD2	-0,744271142
GSE40272	X30762	10726	NUDC	-0,744238901
GSE40272	X40168	9203	ZMYM3	-0,74414852
GSE40272	X38465	126382	NR2C2AP	-0,743908562
GSE40272	X9759	11052	CPSF6	-0,743609937
GSE40272	X31434	4166	CHST6	-0,743493658
GSE40272	X4675	79934	ADCK4	-0,743410148
GSE40272	X507	152992	METTL19	-0,743078753
GSE40272	X6395	79036	C19orf50	-0,742818182
GSE40272	X10128	5097	PCDH1	-0,742704545
GSE40272	X26525	1938	EEF2	-0,742660677
GSE40272	X15663	9022	CLIC3	-0,742367865
GSE40272	X34233	9463	PICK1	-0,74197463
GSE40272	X35273	118472	ZNF511	-0,740540698
GSE40272	X34796	26470	SEZ6L2	-0,740471987
GSE40272	X37967	875	CBS	-0,740210888
GSE40272	X16768	23001	WDFY3	-0,73993129
GSE40272	X42576	79050	NOC4L	-0,739912791
GSE40272	X9946	388554	LOC388554	-0,739868922
GSE40272	X24898	127262	TPRG1L	-0,739696617
GSE40272	X39854	10867	TSPAN9	-0,739658562
GSE40272	X31824	23095	KIF1B	-0,739564482
GSE40272	X1276	205	AK4	-0,73929704
GSE40272	X37968	9989	PPP4R1	-0,739110465
GSE40272	X27067	775	CACNA1C	-0,738872093
GSE40272	X42334	441626	LOC441626	-0,738812368
GSE40272	X4299	4108	MAGEA9	-0,738573996
GSE40272	X6398	55031	USP47	-0,738408562
GSE40272	X17957	4145	MATK	-0,737885307
GSE40272	X28469	23001	WDFY3	-0,737450317
GSE40272	X8278	94030	LRRC4B	-0,73731871
GSE40272	X23860	286887	KRT6C	-0,73705074
GSE40272	X44151	11078	TRIOBP	-0,73665222
GSE40272	X8235	9289	GPR56	-0,736066068
GSE40272	X2374	10858	CYP46A1	-0,736037526
GSE40272	X25406	9074	CLDN6	-0,7357463

GSE40272	X22503	26995	TRUB2	-0,73552537
GSE40272	X38747	6322	SCML1	-0,73519556
GSE40272	X9523	57153	SLC44A2	-0,734955074
GSE40272	X28683	54993	ZSCAN2	-0,734904863
GSE40272	X3588	1455	CSNK1G2	-0,734786998
GSE40272	X9616	9322	TRIP10	-0,734553911
GSE40272	X758	79165	LENG1	-0,734104123
GSE40272	X25156	80243	PREX2	-0,734063953
GSE40272	X9788	56654	NPDC1	-0,733864693
GSE40272	X34587	27348	TOR1B	-0,733593552
GSE40272	X15947	2068	ERCC2	-0,733479387
GSE40272	X41393	118	ADD1	-0,733326638
GSE40272	X13814	1459	CSNK2A2	-0,732551268
GSE40272	X29772	54890	ALKBH5	-0,732533298
GSE40272	X1566	2874	GPS2	-0,732395349
GSE40272	X11529	7094	TLN1	-0,732362579
GSE40272	X34055	401367	LOC401367	-0,732305497
GSE40272	X16170	4035	LRP1	-0,732302326
GSE40272	X27182	9054	NFS1	-0,732052854
GSE40272	X8463	2275	FHL3	-0,73177167
GSE40272	X367	388966	LOC388966	-0,731325581
GSE40272	X31812	114799	ESCO1	-0,730363636
GSE40272	X29404	54498	SMOX	-0,729804968
GSE40272	X44388	51005	AMDHD2	-0,72892759
GSE40272	X9108	337975	KRTAP20-1	-0,728844609
GSE40272	X39483	4880	NPPC	-0,728801268
GSE40272	X13009	4249	MGAT5	-0,728692389
GSE40272	X33480	5097	PCDH1	-0,728596195
GSE40272	X17056	57801	HES4	-0,728587209
GSE40272	X9179	26099	C1orf144	-0,727838795
GSE40272	X33920	55670	PEX26	-0,727660148
GSE40272	X34440	23779	ARHGAP8	-0,727400634
GSE40272	X19365	55627	SMPD4	-0,727301268
GSE40272	X22628	2618	GART	-0,727027484
GSE40272	X28917	7278	TUBA3C	-0,726636892
GSE40272	X16651	5575	PRKAR1B	-0,7264963
GSE40272	X1252	84820	POLR2J4	-0,726363108
GSE40272	X34411	374655	ZNF710	-0,725689746
GSE40272	X1155	23095	KIF1B	-0,724960888
GSE40272	X25091	80761	UPK3B	-0,72465222
GSE40272	X36070	388275	HNRNPA1P7	-0,724052326
GSE40272	X15004	284613	CYB561D1	-0,72337685
GSE40272	X33575	3268	AGFG2	-0,723364165
GSE40272	X34887	5300	PIN1	-0,722934461
GSE40272	X34589	65083	NOL6	-0,722872093
GSE40272	X40752	153090	DAB2IP	-0,722866279
GSE40272	X25449	4035	LRP1	-0,722786469
GSE40272	X10043	441316	LOC441316	-0,722661205
GSE40272	X42951	57404	CYP20A1	-0,722659091
GSE40272	X10628	23361	ZNF629	-0,722328753
GSE40272	X15140	351	APP	-0,722188161
GSE40272	X19020	51295	ECSIT	-0,722107294
GSE40272	X21471	7678	ZNF124	-0,721578753

GSE40272	X19658	442197	OR2I1P	-0,721408562
GSE40272	X23555	7412	VCAM1	-0,720993129
GSE40272	X14268	401642	LOC401642	-0,720252114
GSE40272	X23013	2912	GRM2	-0,719942389
GSE40272	X27995	54936	ADPRHL2	-0,719900634
GSE40272	X37468	3340	NDST1	-0,719844609
GSE40272	X5936	388847	LOC388847	-0,719807611
GSE40272	X15242	4035	LRP1	-0,719298626
GSE40272	X15162	30837	SOCS7	-0,719162262
GSE40272	X23220	9679	FAM53B	-0,719160677
GSE40272	X38305	387882	C12orf75	-0,719062368
GSE40272	X6340	203228	C9orf72	-0,718665433
GSE40272	X2115	64418	TMEM168	-0,718451374
GSE40272	X8494	55702	CCDC94	-0,718313425
GSE40272	X37760	5339	PLEC	-0,718301797
GSE40272	X13219	25	ABL1	-0,718238372
GSE40272	X161	55120	FANCL	-0,717920719
GSE40272	X4676	114038	LINC00313	-0,717885835
GSE40272	X20959	2934	GSN	-0,71767019
GSE40272	X8168	3886	KRT35	-0,717411734
GSE40272	X5822	55898	UNC45A	-0,716939746
GSE40272	X3955	89927	C16orf45	-0,716689746
GSE40272	X26673	7410	VAV2	-0,71646723
GSE40272	X1074	29924	EPN1	-0,716373679
GSE40272	X36736	4035	LRP1	-0,715640063
GSE40272	X11235	55756	INTS9	-0,715548626
GSE40272	X3581	5530	PPP3CA	-0,71534408
GSE40272	X17788	4037	LRP3	-0,715051797
GSE40272	X30723	221178	SPATA13	-0,71487315
GSE40272	X3561	2779	GNAT1	-0,714858879
GSE40272	X4742	23181	DIP2A	-0,714735729
GSE40272	X2933	55352	C17orf79	-0,714517442
GSE40272	X36086	64866	CDCP1	-0,714372622
GSE40272	X22414	126208	ZNF787	-0,713960888
GSE40272	X35048	83696	TRAPPC9	-0,71384778
GSE40272	X11703	192683	SCAMP5	-0,713408034
GSE40272	X42063	149175	MANEAL	-0,713186047
GSE40272	X8517	63941	NECAB3	-0,712997357
GSE40272	X28126	84892	C3orf39	-0,712957717
GSE40272	X11996	51333	ZNF771	-0,712924419
GSE40272	X22784	53349	ZFYVE1	-0,712802854
GSE40272	X7596	286260	LOC286260	-0,71246723
GSE40272	X33667	22876	INPP5F	-0,712365222
GSE40272	X1415	613	BCR	-0,712088795
GSE40272	X32375	10432	RBM14	-0,711208245
GSE40272	X1640	7411	VBP1	-0,71070296
GSE40272	X40004	7551	ZNF3	-0,710235201
GSE40272	X25592	10826	C5orf4	-0,710023256
GSE40272	X24748	2781	GNAZ	-0,709984672
GSE40272	X277	64864	RFX7	-0,709779598
GSE40272	X14560	84255	SLC37A3	-0,709639006
GSE40272	X30867	7975	MAFK	-0,709635835
GSE40272	X1085	126321	C19orf28	-0,709460359

GSE40272	X23586	23235	SIK2	-0,708503171
GSE40272	X37973	8636	SSNA1	-0,708380021
GSE40272	X32745	10994	ILVBL	-0,708297569
GSE40272	X5733	8662	EIF3B	-0,708229387
GSE40272	X27537	1185	CLCN6	-0,708216173
GSE40272	X5646	91445	RNF185	-0,708100423
GSE40272	X34051	29781	NCAPH2	-0,707940275
GSE40272	X15876	9627	SNCAIP	-0,707713002
GSE40272	X31821	23095	KIF1B	-0,707091966
GSE40272	X23069	23095	KIF1B	-0,706477801
GSE40272	X6890	4035	LRP1	-0,706413848
GSE40272	X43384	10025	MED16	-0,706054968
GSE40272	X24670	4035	LRP1	-0,705688689
GSE40272	X43509	5822	PWP2	-0,705645349
GSE40272	X44441	54732	TMED9	-0,705335624
GSE40272	X27272	5663	PSEN1	-0,705201374
GSE40272	X41526	4627	MYH9	-0,704781712
GSE40272	X21106	388623	LOC388623	-0,704653805
GSE40272	X14082	23096	IQSEC2	-0,704636364
GSE40272	X23731	22903	BTBD3	-0,704450317
GSE40272	X21045	23001	WDFY3	-0,704180233
GSE40272	X35192	389695	LOC389695	-0,704028541
GSE40272	X31567	254359	ZDHHC24	-0,703954545
GSE40272	X9742	51280	GOLM1	-0,703928118
GSE40272	X31381	4059	BCAM	-0,703875264
GSE40272	X1754	63941	NECAB3	-0,703748414
GSE40272	X11273	156	ADRBK1	-0,703705074
GSE40272	X27875	442328	LOC442328	-0,703562368
GSE40272	X13450	4752	NEK3	-0,70339482
GSE40272	X12201	56109	PCDHGA6	-0,702381607
GSE40272	X18159	80700	UBXN6	-0,702332452
GSE40272	X27454	7094	TLN1	-0,701942389
GSE40272	X38869	955	ENTPD6	-0,701633721
GSE40272	X7748	133584	EGFLAM	-0,701285412
GSE40272	X40870	64718	UNKL	-0,701179175
GSE40272	X35913	208	AKT2	-0,701099894
GSE40272	X30527	27165	GLS2	-0,700868922
GSE40272	X15014	5433	POLR2D	-0,700756871
GSE40272	X19635	6542	SLC7A2	-0,700371564
GSE40272	X4248	1073	CFL2	-0,699974101
GSE40272	X23932	79025	C20orf195	-0,699726744
GSE40272	X3479	5106	PCK2	-0,699696089
GSE40272	X36602	389856	USP27X	-0,699662791
GSE40272	X32646	7067	THRA	-0,699647992
GSE40272	X38382	442646	LOC442646	-0,699547569
GSE40272	X41376	7094	TLN1	-0,699433404
GSE40272	X5060	3249	HPN	-0,699402748
GSE40272	X9444	30819	KCNIP2	-0,69907611
GSE40272	X8613	23001	WDFY3	-0,698988901
GSE40272	X7271	22888	UBOX5	-0,698894292
GSE40272	X3294	79014	C16orf67	-0,698893235
GSE40272	X27011	89781	HPS4	-0,698726744
GSE40272	X3862	1020	CDK5	-0,698659619

GSE40272	X14167	4130	MAP1A	-0,69865222
GSE40272	X8508	9392	TGFBRAP1	-0,698211945
GSE40272	X22911	10524	KAT5	-0,698163319
GSE40272	X25614	23598	PATZ1	-0,697621036
GSE40272	X38385	29068	ZBTB44	-0,697389535
GSE40272	X13122	23168	RTF1	-0,697221987
GSE40272	X19188	5499	PPP1CA	-0,697206131
GSE40272	X26619	5372	PMM1	-0,697204545
GSE40272	X2221	56970	ATXN7L3	-0,697141121
GSE40272	X30933	7572	ZNF24	-0,696715116
GSE40272	X16603	8420	SNHG3	-0,696462474
GSE40272	X17448	84219	WDR24	-0,696358351
GSE40272	X10109	1982	EIF4G2	-0,696273784
GSE40272	X18257	56999	ADAMTS9	-0,696129493
GSE40272	X2314	55902	ACSS2	-0,696069767
GSE40272	X9952	221078	NSUN6	-0,695905391
GSE40272	X29712	4298	MLLT1	-0,695420719
GSE40272	X6210	10726	NUDC	-0,695264271
GSE40272	X6664	27231	ITGB1BP3	-0,695153805
GSE40272	X13968	55743	CHFR	-0,695113636
GSE40272	X13701	1855	DVL1	-0,69496723
GSE40272	X16573	23001	WDFY3	-0,694942918
GSE40272	X26864	64748	LPPR2	-0,694783827
GSE40272	X1030	5376	PMP22	-0,694187104
GSE40272	X34869	5253	PHF2	-0,693972516
GSE40272	X4257	400470	LOC400470	-0,693961416
GSE40272	X17526	1459	CSNK2A2	-0,693306554
GSE40272	X10084	9657	IQCB1	-0,693240486
GSE40272	X16233	5252	PHF1	-0,693102537
GSE40272	X17799	11168	PSIP1	-0,692263742
GSE40272	X2632	64837	KLC2	-0,692169662
GSE40272	X42163	10734	STAG3	-0,692160148
GSE40272	X40984	138429	PIP5KL1	-0,692022727
GSE40272	X39618	163	AP2B1	-0,691913319
GSE40272	X6546	54715	RBFOX1	-0,691417019
GSE40272	X34048	10994	ILVBL	-0,691385835
GSE40272	X26945	83986	ITFG3	-0,691011628
GSE40272	X34978	5331	PLCB3	-0,690915962
GSE40272	X26012	442248	LOC442248	-0,690730444
GSE40272	X23084	150379	PNPLA5	-0,690709302
GSE40272	X22732	54929	TMEM161A	-0,690458774
GSE40272	X19827	56061	UBFD1	-0,690129493
GSE40272	X1440	10163	WASF2	-0,690042812
GSE40272	X17037	255022	CALHM1	-0,6900074
GSE40272	X9112	8178	ELL	-0,689631607
GSE40272	X33732	10809	STARD10	-0,688901163
GSE40272	X5736	56853	CELF4	-0,688786998
GSE40272	X33097	8994	LIMD1	-0,688645877
GSE40272	X22984	949	SCARB1	-0,688405391
GSE40272	X33442	5802	PTPRS	-0,688403805
GSE40272	X43221	2011	MARK2	-0,688152748
GSE40272	X31383	6720	SREBF1	-0,68808351
GSE40272	X38640	441280	LOC441280	-0,687744186

GSE40272	X12701	6525	SMTN	-0,687390592
GSE40272	X7992	23193	GANAB	-0,687106765
GSE40272	X34590	288	ANK3	-0,686504757
GSE40272	X23355	7542	ZFPL1	-0,686426533
GSE40272	X7208	5306	PITPNA	-0,686299154
GSE40272	X23339	2801	GOLGA2	-0,686190803
GSE40272	X14861	11274	USP18	-0,685947146
GSE40272	X8605	64342	HS1BP3	-0,68563055
GSE40272	X35493	6625	SNRNP70	-0,685356765
GSE40272	X35423	7293	TNFRSF4	-0,685084567
GSE40272	X3245	54108	CHRAC1	-0,685074524
GSE40272	X18176	4035	LRP1	-0,684889535
GSE40272	X8377	439976	LOC439976	-0,68470296
GSE40272	X42092	91828	EXOC3L4	-0,684580867
GSE40272	X1556	55333	SYNJ2BP	-0,684571353
GSE40272	X6074	157922	CAMSAP1	-0,684561839
GSE40272	X8292	11160	ERLIN2	-0,684318182
GSE40272	X16068	351	APP	-0,684276427
GSE40272	X11384	8314	BAP1	-0,684201374
GSE40272	X17227	59	ACTA2	-0,683940803
GSE40272	X1028	10521	DDX17	-0,683733087
GSE40272	X9908	5297	PI4KA	-0,683663319
GSE40272	X40881	1364	CLDN4	-0,683539641
GSE40272	X40823	4899	NRF1	-0,683315539
GSE40272	X30980	25844	YIPF3	-0,68313055
GSE40272	X26063	64423	INF2	-0,682266913
GSE40272	X31245	54108	CHRAC1	-0,682188161
GSE40272	X40645	51282	SCAND1	-0,68161945
GSE40272	X37351	5905	RANGAP1	-0,681426004
GSE40272	X23634	114757	CYGB	-0,681363108
GSE40272	X24829	1459	CSNK2A2	-0,681357294
GSE40272	X11009	25953	PNKD	-0,681245243
GSE40272	X41250	2194	FASN	-0,680661205
GSE40272	X5559	57186	RALGAPA2	-0,680067125
GSE40272	X40541	9509	ADAMTS2	-0,679854123
GSE40272	X41909	128	ADH5	-0,679599894
GSE40272	X12353	4952	OCRL	-0,679538055
GSE40272	X40727	23095	KIF1B	-0,679310782
GSE40272	X37381	1031	CDKN2C	-0,679040169
GSE40272	X39376	4952	OCRL	-0,67894926
GSE40272	X27602	55909	BIN3	-0,678442918
GSE40272	X31748	6714	SRC	-0,678092495
GSE40272	X23729	84152	PPP1R1B	-0,677869979
GSE40272	X17764	400675	LOC400675	-0,677858879
GSE40272	X163	3761	KCNJ4	-0,677233087
GSE40272	X12759	10608	MXD4	-0,677179704
GSE40272	X24051	1613	DAPK3	-0,677153277
GSE40272	X14986	389322	HNRNPKP1	-0,676970402
GSE40272	X12229	5791	PTPRE	-0,67687685
GSE40272	X12705	23373	CRTC1	-0,676824524
GSE40272	X35842	23001	WDFY3	-0,676653805
GSE40272	X30240	7094	TLN1	-0,676146406
GSE40272	X4246	23218	NBEAL2	-0,675995772

GSE40272	X7589	8668	EIF3I	-0,67567019
GSE40272	X24141	80319	CXXC4	-0,675664376
GSE40272	X17380	167359	NIM1	-0,675534355
GSE40272	X5343	4734	NEDD4	-0,675160148
GSE40272	X10096	84629	TNRC18	-0,675082981
GSE40272	X26545	112936	VPS26B	-0,674908034
GSE40272	X7678	3609	ILF3	-0,674874207
GSE40272	X42456	22955	SCMH1	-0,674737844
GSE40272	X4070	55823	VPS11	-0,674704545
GSE40272	X42855	9846	GAB2	-0,674642706
GSE40272	X37478	26001	RNF167	-0,67459778
GSE40272	X27189	57474	ZNF490	-0,674399577
GSE40272	X29312	4035	LRP1	-0,674354651
GSE40272	X38872	80144	FRAS1	-0,674174947
GSE40272	X38058	22933	SIRT2	-0,674090909
GSE40272	X132	57706	DENND1A	-0,674078753
GSE40272	X8411	8848	TSC22D1	-0,673654863
GSE40272	X12307	79147	FKRP	-0,673523256
GSE40272	X22572	441485	LOC441485	-0,673306025
GSE40272	X21932	51286	CEND1	-0,672327167
GSE40272	X17924	351	APP	-0,672197146
GSE40272	X36092	22827	PUF60	-0,672087209
GSE40272	X28449	5396	PRRX1	-0,67177537
GSE40272	X44160	4035	LRP1	-0,67170296
GSE40272	X19180	3782	KCNN3	-0,6709963
GSE40272	X35594	286128	ZFP41	-0,670784884
GSE40272	X36020	1849	DUSP7	-0,670751586
GSE40272	X41467	29781	NCAPH2	-0,670481501
GSE40272	X13694	64866	CDCP1	-0,670158562
GSE40272	X20046	342371	ATXN1L	-0,670099366
GSE40272	X18852	351	APP	-0,670085095
GSE40272	X37622	2874	GPS2	-0,670051797
GSE40272	X14874	54751	FBLIM1	-0,669957717
GSE40272	X20917	23001	WDFY3	-0,66983351
GSE40272	X38693	84108	PCGF6	-0,669727273
GSE40272	X12102	10523	CHERP	-0,669598837
GSE40272	X20761	7310	ZRSR1	-0,669327167
GSE40272	X41191	404744	AAA1	-0,669313953
GSE40272	X32254	84942	WDR73	-0,669113108
GSE40272	X5864	3850	KRT3	-0,668942389
GSE40272	X3876	1312	COMT	-0,668874207
GSE40272	X29397	8720	MBTPS1	-0,66879704
GSE40272	X22305	376412	RNF126P1	-0,66852907
GSE40272	X11907	5573	PRKAR1A	-0,668436575
GSE40272	X11638	27315	PGAP2	-0,668207188
GSE40272	X24899	55898	UNC45A	-0,667716173
GSE40272	X17772	53635	PTOV1	-0,667643235
GSE40272	X40999	9604	RNF14	-0,667335624
GSE40272	X24197	84519	ACRBP	-0,667231501
GSE40272	X16439	81926	FAM108A1	-0,667184989
GSE40272	X32350	10777	ARPP21	-0,666714059
GSE40272	X32380	29967	LRP12	-0,6664963
GSE40272	X28366	388407	C17orf82	-0,666495243

GSE40272	X4106	4035	LRP1	-0,666336152
GSE40272	X28569	343531	C1orf224	-0,666304968
GSE40272	X1743	4041	LRP5	-0,665383192
GSE40272	X6652	3985	LIMK2	-0,665154863
GSE40272	X3434	5202	PFDN2	-0,664931818
GSE40272	X29334	389473	LOC389473	-0,664884778
GSE40272	X7587	4237	MFAP2	-0,664771142
GSE40272	X21576	5792	PTPRF	-0,664612051
GSE40272	X35765	7837	PXDN	-0,664415433
GSE40272	X42051	4952	OCRL	-0,664307082
GSE40272	X31143	610	HCN2	-0,664057611
GSE40272	X3526	55009	C19orf24	-0,663955074
GSE40272	X12867	10072	DPP3	-0,663751586
GSE40272	X15305	9448	MAP4K4	-0,66357981
GSE40272	X38592	4035	LRP1	-0,663463002
GSE40272	X22371	26994	RNF11	-0,663299683
GSE40272	X32264	65265	C8orf33	-0,663264799
GSE40272	X721	84335	AKT1S1	-0,663264271
GSE40272	X22218	1174	AP1S1	-0,663089852
GSE40272	X359	54815	GATAD2A	-0,663073996
GSE40272	X36725	5339	PLEC	-0,662643235
GSE40272	X7591	816	CAMK2B	-0,662570296
GSE40272	X7818	4035	LRP1	-0,66238055
GSE40272	X631	23224	SYNE2	-0,6622426
GSE40272	X41407	8704	B4GALT2	-0,662146934
GSE40272	X27208	5763	PTMS	-0,662056025
GSE40272	X14678	11021	RAB35	-0,661942918
GSE40272	X23356	9961	MVP	-0,661695032
GSE40272	X7659	1,00E+04	AKT3	-0,661588795
GSE40272	X17284	23499	MACF1	-0,661288584
GSE40272	X26085	8864	PER2	-0,661256342
GSE40272	X9824	4035	LRP1	-0,661202431
GSE40272	X24924	140460	ASB7	-0,66116649
GSE40272	X30973	84271	POLDIP3	-0,660960359
GSE40272	X10088	9144	SYNGR2	-0,660884778
GSE40272	X38581	2022	ENG	-0,660656977
GSE40272	X25093	9535	GMFG	-0,660417548
GSE40272	X43330	1981	EIF4G1	-0,660361522
GSE40272	X7299	79794	C12orf49	-0,659993658
GSE40272	X34967	7436	VLDLR	-0,659803383
GSE40272	X965	79080	CCDC86	-0,659267442
GSE40272	X25222	7275	TUB	-0,659236258
GSE40272	X29124	23498	HAAO	-0,658975159
GSE40272	X4577	55349	CHDH	-0,658751586
GSE40272	X22736	58513	EPS15L1	-0,658561839
GSE40272	X38372	9260	PDLIM7	-0,658332452
GSE40272	X7327	84572	GNPTG	-0,658328753
GSE40272	X38875	7389	UROD	-0,658061839
GSE40272	X9696	25870	SUMF2	-0,657838266
GSE40272	X22735	26100	WIPI2	-0,657544926
GSE40272	X22465	4756	NEO1	-0,657530127
GSE40272	X35507	6323	SCN1A	-0,657077696
GSE40272	X28905	84875	PARP10	-0,657073467

GSE40272	X31722	6887	TAL2	-0,656596195
GSE40272	X15424	9294	S1PR2	-0,656553383
GSE40272	X22203	10093	ARPC4	-0,65644186
GSE40272	X28111	9145	SYNGR1	-0,656160148
GSE40272	X21850	7581	ZNF33A	-0,656032241
GSE40272	X7881	23001	WDFY3	-0,655827167
GSE40272	X15680	4440	MSI1	-0,655654863
GSE40272	X26618	10755	GIPC1	-0,655613636
GSE40272	X4256	4035	LRP1	-0,655596195
GSE40272	X1156	6892	TAPBP	-0,655449789
GSE40272	X40906	1435	CSF1	-0,655412791
GSE40272	X29798	23476	BRD4	-0,65502537
GSE40272	X29831	266675	BEST4	-0,654948732
GSE40272	X5174	55806	HR	-0,654533827
GSE40272	X38749	23001	WDFY3	-0,654298626
GSE40272	X16122	10383	TUBB2C	-0,65422463
GSE40272	X4926	91966	CXorf40A	-0,654002114
GSE40272	X743	254863	C17orf61	-0,653989958
GSE40272	X8547	71	ACTG1	-0,65385518
GSE40272	X30471	57158	JPH2	-0,653825053
GSE40272	X11300	7188	TRAF5	-0,653750529
GSE40272	X5193	64145	ZFYVE20	-0,653645877
GSE40272	X42534	56114	PCDHGA1	-0,65358351
GSE40272	X41834	8717	TRADD	-0,653301268
GSE40272	X15925	92285	ZNF585B	-0,652518499
GSE40272	X12528	10400	PEMT	-0,651463002
GSE40272	X32748	23095	KIF1B	-0,651268499
GSE40272	X21996	256364	EML3	-0,651255285
GSE40272	X4296	23001	WDFY3	-0,650864165
GSE40272	X37944	7511	XPNPEP1	-0,65080814
GSE40272	X5220	8496	PPFIBP1	-0,650617336
GSE40272	X41689	81890	QTRT1	-0,650470402
GSE40272	X16778	6926	TBX3	-0,650419662
GSE40272	X16789	400726	LOC400726	-0,65027167
GSE40272	X31346	8971	H1FX	-0,649954545
GSE40272	X33237	91526	ANKRD44	-0,649936047
GSE40272	X42268	1523	CUX1	-0,649868393
GSE40272	X19023	93589	CACNA2D4	-0,649840909
GSE40272	X41618	127540	HMGB4	-0,649532241
GSE40272	X17403	55572	FOXRED1	-0,649511628
GSE40272	X17784	6305	SBF1	-0,649296512
GSE40272	X34177	5297	PI4KA	-0,649062896
GSE40272	X24417	351	APP	-0,649036469
GSE40272	X6102	51031	GLOD4	-0,648569239
GSE40272	X41335	1175	AP2S1	-0,648553911
GSE40272	X6089	10526	IPO8	-0,648309197
GSE40272	X12388	55627	SMPD4	-0,64797463
GSE40272	X1219	310	ANXA7	-0,647342495
GSE40272	X18785	1398	CRK	-0,64709778
GSE40272	X10756	400664	LOC400664	-0,64705814
GSE40272	X34240	55846	ITFG2	-0,646990486
GSE40272	X24701	3693	ITGB5	-0,646926533
GSE40272	X168	816	CAMK2B	-0,646722516

GSE40272	X21156	64943	NT5DC2	-0,646667548
GSE40272	X29034	23095	KIF1B	-0,64639852
GSE40272	X1298	8861	LDB1	-0,646280655
GSE40272	X44141	23383	MAU2	-0,645997357
GSE40272	X19953	949	SCARB1	-0,645994186
GSE40272	X37814	442174	LOC442174	-0,645862579
GSE40272	X13018	389860	PAGE2B	-0,645587209
GSE40272	X38457	440476	LOC440476	-0,64557241
GSE40272	X28066	51308	REEP2	-0,645395349
GSE40272	X31408	10106	CTDSP2	-0,644961416
GSE40272	X18260	4204	MECP2	-0,644895877
GSE40272	X4987	59338	PLEKHA1	-0,644694503
GSE40272	X35093	4240	MFGE8	-0,644615222
GSE40272	X9944	3297	HSF1	-0,644302854
GSE40272	X36348	442503	LOC442503	-0,64427537
GSE40272	X5034	4035	LRP1	-0,64427167
GSE40272	X26482	89870	TRIM15	-0,644184461
GSE40272	X9335	79705	LRRK1	-0,644168605
GSE40272	X13534	1509	CTSD	-0,644123679
GSE40272	X6213	2886	GRB7	-0,644091966
GSE40272	X32258	29904	EEF2K	-0,644014271
GSE40272	X12894	51226	COPZ2	-0,643763214
GSE40272	X23893	23519	ANP32D	-0,643201374
GSE40272	X22588	5576	PRKAR2A	-0,642831395
GSE40272	X6426	4795	NFKBIL1	-0,64278277
GSE40272	X6330	3972	LHB	-0,642739958
GSE40272	X17871	3304	HSPA1B	-0,642491015
GSE40272	X12249	9522	SCAMP1	-0,642488372
GSE40272	X6661	9784	SNX17	-0,642477273
GSE40272	X34872	2048	EPHB2	-0,642349366
GSE40272	X16310	4794	NFKBIE	-0,642229387
GSE40272	X9690	389710	LOC389710	-0,642091966
GSE40272	X5449	1727	CYB5R3	-0,641883721
GSE40272	X7656	6584	SLC22A5	-0,641826638
GSE40272	X6324	124245	ZC3H18	-0,641752643
GSE40272	X23744	4035	LRP1	-0,641259514
GSE40272	X27225	26115	TANC2	-0,641221987
GSE40272	X30915	4952	OCRL	-0,641090381
GSE40272	X3508	2516	NR5A1	-0,64104334
GSE40272	X19318	80776	B9D2	-0,640837738
GSE40272	X6815	93953	ACRC	-0,640776427
GSE40272	X35953	344165	LOC344165	-0,640690275
GSE40272	X23730	29803	REPIN1	-0,640439218
GSE40272	X4792	131185	LOC131185	-0,640214059
GSE40272	X11185	114609	TIRAP	-0,640182875
GSE40272	X5407	29801	ZDHHC8	-0,640078753
GSE40272	X1454	50700	RDH8	-0,639761099
GSE40272	X33463	1945	EFNA4	-0,639744186
GSE40272	X9628	387644	LINC00202	-0,639382664
GSE40272	X24269	8045	RASSF7	-0,63848203
GSE40272	X24274	84224	NBPF3	-0,638479915
GSE40272	X1560	84164	ASCC2	-0,63834778
GSE40272	X5483	55577	NAGK	-0,63802167

GSE40272	X30288	2820	GPD2	-0,637966702
GSE40272	X43081	4035	LRP1	-0,637954017
GSE40272	X41221	6651	SON	-0,63762685
GSE40272	X248	8085	MLL2	-0,637352537
GSE40272	X33444	79683	ZDHHC14	-0,637233087
GSE40272	X34410	84861	KLHL22	-0,637204545
GSE40272	X28700	27153	ZNF777	-0,63717389
GSE40272	X5431	6239	RREB1	-0,637016385
GSE40272	X27986	84656	GLYR1	-0,636239958
GSE40272	X9513	3081	HGD	-0,636149577
GSE40272	X23425	2909	ARHGAP35	-0,636027484
GSE40272	X23891	3732	CD82	-0,636027484
GSE40272	X8621	84255	SLC37A3	-0,635953488
GSE40272	X39862	80139	ZNF703	-0,635660677
GSE40272	X27378	10134	BCAP31	-0,635536469
GSE40272	X27440	284338	PRR19	-0,634454545
GSE40272	X21813	388799	C20orf107	-0,634390592
GSE40272	X16169	4035	LRP1	-0,634343023
GSE40272	X27455	5430	POLR2A	-0,634075053
GSE40272	X11425	4952	OCRL	-0,633608879
GSE40272	X9790	29123	ANKRD11	-0,633548626
GSE40272	X30877	5524	PPP2R4	-0,633420719
GSE40272	X17922	5788	PTPRC	-0,633330338
GSE40272	X35837	3762	KCNJ5	-0,633261628
GSE40272	X8429	113178	SCAMP4	-0,632910677
GSE40272	X1220	4952	OCRL	-0,632354651
GSE40272	X37131	55547	HAB1	-0,632354123
GSE40272	X2004	266655	LINC00094	-0,632332452
GSE40272	X12314	5297	PI4KA	-0,631613636
GSE40272	X4900	23001	WDFY3	-0,630791755
GSE40272	X33602	5338	PLD2	-0,630689218
GSE40272	X30270	54436	SH3TC1	-0,630567653
GSE40272	X38851	84331	FAM195A	-0,630413848
GSE40272	X5517	10963	STIP1	-0,630384249
GSE40272	X40009	125144	C17orf76-AS1	-0,630306025
GSE40272	X3076	4952	OCRL	-0,630304968
GSE40272	X34724	4043	LRPAP1	-0,630279598
GSE40272	X27707	5297	PI4KA	-0,630127907
GSE40272	X20450	5087	PBX1	-0,629862051
GSE40272	X27903	89910	UBE3B	-0,629469345
GSE40272	X28543	79089	TMUB2	-0,629437632
GSE40272	X33941	51160	VPS28	-0,629259514
GSE40272	X10340	3936	LCP1	-0,629110465
GSE40272	X25691	27	ABL2	-0,628807611
GSE40272	X4806	6573	SLC19A1	-0,628751586
GSE40272	X16996	351	APP	-0,628604651
GSE40272	X43634	4046	LSP1	-0,628360994
GSE40272	X17097	7094	TLN1	-0,628339852
GSE40272	X36034	7799	PRDM2	-0,628263214
GSE40272	X5382	1819	DRG2	-0,627859937
GSE40272	X16046	1459	CSNK2A2	-0,627803383
GSE40272	X29845	80149	ZC3H12A	-0,627628964
GSE40272	X26332	285601	GPR150	-0,627613636

GSE40272	X6343	10902	BRD8	-0,627578753
GSE40272	X3856	391039	CFL1P2	-0,627299683
GSE40272	X14503	1741	DLG3	-0,62728277
GSE40272	X17518	7392	USF2	-0,626867865
GSE40272	X12880	6238	RRBP1	-0,626499471
GSE40272	X25699	3136	HLA-H	-0,626396934
GSE40272	X12163	10494	STK25	-0,626365222
GSE40272	X33967	5865	RAB3B	-0,626290698
GSE40272	X17277	348825	TPRXL	-0,626012156
GSE40272	X7524	7536	SF1	-0,625942389
GSE40272	X22759	79068	FTO	-0,62580814
GSE40272	X29828	63941	NECAB3	-0,625723044
GSE40272	X18278	1325	CORT	-0,625662791
GSE40272	X11717	55191	NADSYN1	-0,624705603
GSE40272	X18849	351	APP	-0,624537526
GSE40272	X32565	64592	CLONE795723	-0,624384778
GSE40272	X15833	9375	TM9SF2	-0,624375264
GSE40272	X44338	441686	LOC441686	-0,623889535
GSE40272	X37756	6687	SPG7	-0,623827696
GSE40272	X7798	54956	PARP16	-0,62351797
GSE40272	X29945	5826	ABCD4	-0,623252643
GSE40272	X6383	170961	ANKRD24	-0,623164905
GSE40272	X31184	56957	OTUD7B	-0,623100423
GSE40272	X34698	401330	LOC401330	-0,622983615
GSE40272	X37767	401367	LOC401367	-0,622530655
GSE40272	X4243	23187	PHLDB1	-0,622450846
GSE40272	X16019	7922	SLC39A7	-0,622322939
GSE40272	X38410	401330	LOC401330	-0,621912262
GSE40272	X6394	114049	WBSCR22	-0,62182611
GSE40272	X22797	56834	GPR137	-0,621743658
GSE40272	X6272	10295	BCKDK	-0,621739958
GSE40272	X14723	1488	CTBP2	-0,621566068
GSE40272	X37273	23547	LILRA4	-0,621495772
GSE40272	X23275	3487	IGFBP4	-0,621388478
GSE40272	X4285	442123	LOC442123	-0,620763214
GSE40272	X5401	6043	SNORA63	-0,620717759
GSE40272	X27078	6468	FBXW4	-0,619910148
GSE40272	X30679	9811	CTIF	-0,619642706
GSE40272	X42533	435	ASL	-0,619071882
GSE40272	X15800	51684	SUFU	-0,61904704
GSE40272	X6150	7072	TIA1	-0,618708774
GSE40272	X4628	196264	MPZL3	-0,618665433
GSE40272	X23350	345375	LOC345375	-0,618339323
GSE40272	X19618	283578	TMED8	-0,61802167
GSE40272	X42010	1384	CRAT	-0,617875264
GSE40272	X10750	2934	GSN	-0,617736786
GSE40272	X7918	5611	DNAJC3	-0,617372622
GSE40272	X10095	23030	KDM4B	-0,617079281
GSE40272	X29029	23648	SSBP3	-0,616909091
GSE40272	X13625	23476	BRD4	-0,616877907
GSE40272	X5589	1656	DDX6	-0,616090909
GSE40272	X7217	26502	NARF	-0,61602907
GSE40272	X78	25855	BRMS1	-0,616016385

GSE40272	X30995	22827	PUF60	-0,615763214
GSE40272	X15302	163859	C1orf55	-0,615760042
GSE40272	X8067	23020	SNRNP200	-0,615660677
GSE40272	X44073	2972	BRF1	-0,615263214
GSE40272	X28667	581	BAX	-0,614754228
GSE40272	X38147	8398	PLA2G6	-0,614656448
GSE40272	X42272	2787	GNG5	-0,614329281
GSE40272	X22523	56927	GPR108	-0,614156977
GSE40272	X4239	2907	GRINA	-0,614150106
GSE40272	X12917	63898	SH2D4A	-0,614028541
GSE40272	X11301	9962	SLC23A2	-0,613903805
GSE40272	X10671	5892	RAD51D	-0,613837209
GSE40272	X11672	8751	ADAM15	-0,613820296
GSE40272	X174	7402	UTRN	-0,61372093
GSE40272	X10743	3985	LIMK2	-0,61358351
GSE40272	X30711	196120	LOC196120	-0,613501586
GSE40272	X3226	11149	BVES	-0,613385835
GSE40272	X2664	58508	MLL3	-0,613204545
GSE40272	X9789	114757	CYGB	-0,613188161
GSE40272	X8182	80121	FLJ23022	-0,612930233
GSE40272	X37181	64834	ELOVL1	-0,612834567
GSE40272	X30507	10664	CTCF	-0,612235729
GSE40272	X30119	117584	RFFL	-0,611078224
GSE40272	X30399	1639	DCTN1	-0,610960888
GSE40272	X43536	23396	PIP5K1C	-0,610817653
GSE40272	X9529	3275	PRMT2	-0,610763214
GSE40272	X6121	55558	PLXNA3	-0,61069556
GSE40272	X19617	26108	PYGO1	-0,610654334
GSE40272	X13362	81566	CSRNP2	-0,61005444
GSE40272	X43782	29925	GMPPB	-0,609880021
GSE40272	X9534	80196	RNF34	-0,609835095
GSE40272	X18074	54869	EPS8L1	-0,609498943
GSE40272	X11469	442646	LOC442646	-0,609491543
GSE40272	X34049	84960	KIAA1984	-0,609479915
GSE40272	X2158	55954	ZMAT5	-0,609424947
GSE40272	X19642	125875	CLDND2	-0,609276427
GSE40272	X32141	8106	PABPN1	-0,60894186
GSE40272	X26567	80144	FRAS1	-0,608732558
GSE40272	X2916	391723	HELT	-0,608729915
GSE40272	X11671	576	BAI2	-0,608686575
GSE40272	X36044	89887	ZNF628	-0,608653277
GSE40272	X28091	4134	MAP4	-0,608649577
GSE40272	X19777	351	APP	-0,608465116
GSE40272	X23138	57862	ZNF410	-0,608221987
GSE40272	X34862	55004	LAMTOR1	-0,608078753
GSE40272	X27246	4323	MMP14	-0,608034884
GSE40272	X33486	27	ABL2	-0,608007928
GSE40272	X18863	221937	FOXK1	-0,607913848
GSE40272	X544	5430	POLR2A	-0,607865222
GSE40272	X5922	85007	AGXT2L2	-0,607715645
GSE40272	X28551	581	BAX	-0,607093023
GSE40272	X1672	126119	JOSD2	-0,607028013
GSE40272	X42571	55291	PPP6R3	-0,607006871

GSE40272	X17098	4035	LRP1	-0,60667759
GSE40272	X7794	9703	KIAA0100	-0,60665222
GSE40272	X12199	219	ALDH1B1	-0,606650106
GSE40272	X12413	6159	RPL29	-0,606365751
GSE40272	X30238	1509	CTSD	-0,606187104
GSE40272	X30885	10171	RCL1	-0,606131607
GSE40272	X22896	9527	GOSR1	-0,60577537
GSE40272	X20579	3185	HNRNPF	-0,605602008
GSE40272	X31112	7090	TLE3	-0,605510571
GSE40272	X13311	11237	RNF24	-0,605458245
GSE40272	X20281	85363	TRIM5	-0,605418605
GSE40272	X20679	23001	WDFY3	-0,605390063
GSE40272	X6747	23095	KIF1B	-0,605189218
GSE40272	X17053	22994	AZI1	-0,604899577
GSE40272	X34238	79641	ROGDI	-0,604887949
GSE40272	X31713	2580	GAK	-0,604747886
GSE40272	X14327	440271	LOC440271	-0,604374207
GSE40272	X43400	6610	SMPD2	-0,604294397
GSE40272	X20576	81794	ADAMTS10	-0,604284884
GSE40272	X15383	9246	UBE2L6	-0,604108879
GSE40272	X11310	79144	PPDPF	-0,603994715
GSE40272	X15918	1889	ECE1	-0,603558668
GSE40272	X31158	8241	RBM10	-0,603514799
GSE40272	X14076	401065	LOC401065	-0,603462474
GSE40272	X1576	9524	TECR	-0,602688689
GSE40272	X1696	442145	LOC442145	-0,602249471
GSE40272	X13687	79643	CHMP6	-0,601926533
GSE40272	X20031	7094	TLN1	-0,601701903
GSE40272	X24944	347088	GPR144	-0,60159408
GSE40272	X35577	23185	LARP4B	-0,60091649
GSE40272	X14060	5326	PLAGL2	-0,600655391
GSE40272	X37828	1107	CHD3	-0,600528541
GSE40272	X42018	23001	WDFY3	-0,600415962
GSE40272	X10730	9149	DYRK1B	-0,599772199
GSE40272	X14955	51816	CECR1	-0,599682875
GSE40272	X1570	401911	RPL29P33	-0,599478858
GSE40272	X32509	2002	ELK1	-0,599352537
GSE40272	X37160	8751	ADAM15	-0,59934408
GSE40272	X11966	8209	C21orf33	-0,599106237
GSE40272	X18710	1995	ELAVL3	-0,599033298
GSE40272	X7342	55898	UNC45A	-0,598842495
GSE40272	X16281	3297	HSF1	-0,598805497
GSE40272	X4668	441221	LOC441221	-0,59845666
GSE40272	X41985	23095	KIF1B	-0,598309197
GSE40272	X41308	27125	AFF4	-0,598122622
GSE40272	X41066	5795	PTPRJ	-0,598010042
GSE40272	X22617	3329	HSPD1	-0,597845666
GSE40272	X3767	146547	PRSS36	-0,597511628
GSE40272	X13956	115098	CCDC124	-0,597005285
GSE40272	X16280	120	ADD3	-0,596847252
GSE40272	X22910	25	ABL1	-0,596752114
GSE40272	X12583	394	ARHGAP5	-0,596124207
GSE40272	X8641	4952	OCRL	-0,59610148

GSE40272	X28376	79092	CARD14	-0,595738372
GSE40272	X12593	23476	BRD4	-0,595106765
GSE40272	X15940	57396	CLK4	-0,594819767
GSE40272	X35768	23001	WDFY3	-0,594662791
GSE40272	X15381	64601	VPS16	-0,594562896
GSE40272	X14176	351	APP	-0,59436945
GSE40272	X17507	4234	METTL1	-0,594274841
GSE40272	X40977	9230	RAB11B	-0,594224101
GSE40272	X36945	6576	SLC25A1	-0,593944503
GSE40272	X11680	5430	POLR2A	-0,593867865
GSE40272	X37966	4152	MBD1	-0,593842495
GSE40272	X22990	4214	MAP3K1	-0,593820825
GSE40272	X25880	79368	FCRL2	-0,593086681
GSE40272	X4994	10067	SCAMP3	-0,592390592
GSE40272	X15967	6314	ATXN7	-0,592291226
GSE40272	X30977	57216	VANGL2	-0,592138478
GSE40272	X17469	55527	FEM1A	-0,591552854
GSE40272	X16422	144983	HNRNPA1L2	-0,59132241
GSE40272	X20293	26505	CNNM3	-0,591290169
GSE40272	X11428	351	APP	-0,591257928
GSE40272	X42153	4035	LRP1	-0,591063425
GSE40272	X41100	5297	PI4KA	-0,591024313
GSE40272	X32117	51700	CYB5R2	-0,590901691
GSE40272	X23791	6294	SAFB	-0,590680761
GSE40272	X30663	11316	COPE	-0,590352008
GSE40272	X36047	79007	DBNDD1	-0,590321353
GSE40272	X37317	23129	PLXND1	-0,590051797
GSE40272	X23274	84305	WIBG	-0,590036469
GSE40272	X707	8668	EIF3I	-0,589904863
GSE40272	X8989	11155	LDB3	-0,589236258
GSE40272	X15024	257364	SNX33	-0,58915222
GSE40272	X37933	5606	MAP2K3	-0,588665962
GSE40272	X36004	79924	ADM2	-0,588512685
GSE40272	X24410	284415	VSTM1	-0,588354123
GSE40272	X8243	5297	PI4KA	-0,58830444
GSE40272	X35405	29882	ANAPC2	-0,588229915
GSE40272	X7886	5339	PLEC	-0,58822093
GSE40272	X1001	79575	ABHD8	-0,588059197
GSE40272	X18724	83692	CD99L2	-0,587938689
GSE40272	X33882	8573	CASK	-0,58734408
GSE40272	X26946	1509	CTSD	-0,587103594
GSE40272	X13145	6778	STAT6	-0,586973044
GSE40272	X2201	55152	DALRD3	-0,586729915
GSE40272	X40106	11313	LYPLA2	-0,586386364
GSE40272	X23299	4781	NFIB	-0,585374207
GSE40272	X34390	3892	KRT86	-0,585314482
GSE40272	X21665	2130	EWSR1	-0,584909619
GSE40272	X33611	5037	PEBP1	-0,584799683
GSE40272	X635	1019	CDK4	-0,584525899
GSE40272	X4105	7094	TLN1	-0,584411734
GSE40272	X19301	2909	ARHGAP35	-0,584042283
GSE40272	X764	1265	CNN2	-0,583795983
GSE40272	X14461	1509	CTSD	-0,583200317

GSE40272	X11148	56243	KIAA1217	-0,583128436
GSE40272	X29921	8396	PIP4K2B	-0,583090909
GSE40272	X9413	80255	SLC35F5	-0,583078224
GSE40272	X12817	23234	DNAJC9	-0,583054968
GSE40272	X30046	7023	TFAP4	-0,58282241
GSE40272	X7935	29984	RHOD	-0,582751057
GSE40272	X27022	9334	B4GALT5	-0,582245772
GSE40272	X11408	55250	ELP2	-0,582026427
GSE40272	X41589	7916	PRRC2A	-0,581865222
GSE40272	X3127	140460	ASB7	-0,581850423
GSE40272	X22375	79947	DHDDS	-0,581832452
GSE40272	X12806	23341	DNAJC16	-0,581319767
GSE40272	X37768	56949	XAB2	-0,580899577
GSE40272	X18809	10902	BRD8	-0,58073203
GSE40272	X9379	3611	ILK	-0,580658034
GSE40272	X6750	29763	PACSIN3	-0,580504757
GSE40272	X4695	91300	C19orf22	-0,580467759
GSE40272	X5147	794	CALB2	-0,580006342
GSE40272	X35795	4784	NFIX	-0,579636364
GSE40272	X20879	11078	TRIOBP	-0,578858351
GSE40272	X43465	3933	LCN1	-0,578646934
GSE40272	X33697	3156	HMGCR	-0,578578753
GSE40272	X28352	442403	LOC442403	-0,578528541
GSE40272	X27234	3190	HNRNPK	-0,578453488
GSE40272	X24379	64866	CDCP1	-0,578365222
GSE40272	X26273	351	APP	-0,578347252
GSE40272	X40929	90161	HS6ST2	-0,57822463
GSE40272	X23967	285444	LOC285444	-0,577935518
GSE40272	X10784	5709	PSMD3	-0,577780655
GSE40272	X44386	56910	STARD7	-0,577674947
GSE40272	X9365	146206	RLTPR	-0,577649577
GSE40272	X42844	6813	STXBP2	-0,577617865
GSE40272	X21498	54434	SSH1	-0,577469873
GSE40272	X35366	23476	BRD4	-0,577182347
GSE40272	X38584	8704	B4GALT2	-0,577127378
GSE40272	X2023	5150	PDE7A	-0,576988372
GSE40272	X33582	83475	DOHH	-0,576961416
GSE40272	X503	4651	MYO10	-0,576951374
GSE40272	X12048	5089	PBX2	-0,576936575
GSE40272	X43962	551	AVP	-0,576936575
GSE40272	X38253	91219	FAM108A6P	-0,576469345
GSE40272	X16293	84920	ALG10	-0,576462474
GSE40272	X16971	3782	KCNN3	-0,576312368
GSE40272	X3171	2783	GNB2	-0,576196617
GSE40272	X43846	23112	TNRC6B	-0,576133721
GSE40272	X24159	151987	PPP4R2	-0,575983615
GSE40272	X11068	11338	U2AF2	-0,575511099
GSE40272	X18026	4035	LRP1	-0,57522833
GSE40272	X42294	9817	KEAP1	-0,575063425
GSE40272	X7040	4035	LRP1	-0,575035941
GSE40272	X13144	401180	LOC401180	-0,5750037
GSE40272	X32636	442206	GPR166P	-0,574976744
GSE40272	X35664	4952	OCRL	-0,574609937

GSE40272	X41136	26043	UBXN7	-0,574600423
GSE40272	X9778	5297	PI4KA	-0,574273784
GSE40272	X5671	535	ATP6V0A1	-0,573758457
GSE40272	X37425	147991	DPY19L3	-0,573430761
GSE40272	X3630	55898	UNC45A	-0,573304968
GSE40272	X15223	84922	FIZ1	-0,573162791
GSE40272	X30722	23181	DIP2A	-0,572896934
GSE40272	X9199	2909	ARHGAP35	-0,572724101
GSE40272	X24729	81698	C15orf5	-0,572708245
GSE40272	X12356	351	APP	-0,57266649
GSE40272	X37744	9781	RNF144A	-0,57260148
GSE40272	X4455	26469	PTPN18	-0,572379493
GSE40272	X8202	5609	MAP2K7	-0,572290698
GSE40272	X2225	7402	UTRN	-0,572201903
GSE40272	X37272	135	ADORA2A	-0,570696089
GSE40272	X34565	80790	CMIP	-0,570673362
GSE40272	X30198	51763	INPP5K	-0,570621564
GSE40272	X34736	4952	OCRL	-0,570606237
GSE40272	X8266	27243	CHMP2A	-0,570590909
GSE40272	X716	23677	SH3BP4	-0,570487315
GSE40272	X33766	51	ACOX1	-0,570244715
GSE40272	X9580	338785	KRT79	-0,569962474
GSE40272	X14212	351	APP	-0,569876321
GSE40272	X9349	8396	PIP4K2B	-0,569855708
GSE40272	X15906	283578	TMED8	-0,569553383
GSE40272	X38579	3665	IRF7	-0,569469873
GSE40272	X27421	6618	SNAPC2	-0,569456131
GSE40272	X16576	5316	PKNOX1	-0,569022727
GSE40272	X19983	6795	AURKC	-0,568911734
GSE40272	X20952	11335	CBX3	-0,568696617
GSE40272	X33767	23499	MACF1	-0,568448732
GSE40272	X28300	23759	PPIL2	-0,568438689
GSE40272	X13536	7094	TLN1	-0,56819186
GSE40272	X34032	9746	CLSTN3	-0,568178647
GSE40272	X26240	79701	C17orf101	-0,567996829
GSE40272	X9445	54461	FBXW5	-0,567937632
GSE40272	X863	9150	CTDP1	-0,567915962
GSE40272	X38328	10423	CDIPT	-0,567383192
GSE40272	X41159	348995	NUP43	-0,567219873
GSE40272	X21488	11142	PKIG	-0,566519556
GSE40272	X23723	84798	C19orf48	-0,566374207
GSE40272	X37578	1912	PHC2	-0,566324524
GSE40272	X2625	5297	PI4KA	-0,566285941
GSE40272	X38686	11168	PSIP1	-0,565739429
GSE40272	X31064	6844	VAMP2	-0,56562315
GSE40272	X28137	118460	EXOSC6	-0,565561311
GSE40272	X41797	9618	TRAF4	-0,565209302
GSE40272	X8391	8926	SNURF	-0,564908034
GSE40272	X24041	23133	PHF8	-0,564615751
GSE40272	X28707	79813	EHMT1	-0,56457981
GSE40272	X19186	8720	MBTPS1	-0,564475159
GSE40272	X12098	9416	DDX23	-0,564228858
GSE40272	X31973	4839	NOP2	-0,564133192

GSE40272	X38695	79031	PDCL3	-0,5639963
GSE40272	X19423	954	ENTPD2	-0,56394926
GSE40272	X229	54880	BCOR	-0,563919662
GSE40272	X41160	55002	TMCO3	-0,563788055
GSE40272	X9016	29123	ANKRD11	-0,563733615
GSE40272	X6367	26154	ABCA12	-0,563026956
GSE40272	X40991	23400	ATP13A2	-0,563026956
GSE40272	X22842	9049	AIP	-0,562969345
GSE40272	X39527	51228	GLTP	-0,562789112
GSE40272	X15825	8672	EIF4G3	-0,562765328
GSE40272	X8746	4035	LRP1	-0,562696617
GSE40272	X18272	201163	FLCN	-0,562427061
GSE40272	X5893	5352	PLOD2	-0,562302326
GSE40272	X38902	79966	SCD5	-0,562169662
GSE40272	X345	26226	FBXW4P1	-0,562033827
GSE40272	X1189	23001	WDFY3	-0,561742072
GSE40272	X22561	351	APP	-0,561356765
GSE40272	X24587	114049	WBSCR22	-0,561347252
GSE40272	X16008	54867	TMEM214	-0,561179704
GSE40272	X3098	1774	DNASE1L1	-0,560953488
GSE40272	X43232	7094	TLN1	-0,560904334
GSE40272	X36928	29934	SNX12	-0,560465645
GSE40272	X460	10454	TAB1	-0,560169662
GSE40272	X18891	9859	CEP170	-0,56013055
GSE40272	X13833	1500	CTNND1	-0,559875793
GSE40272	X355	55227	LRRC1	-0,5595
GSE40272	X13521	7317	UBA1	-0,559451374
GSE40272	X7341	10922	FASTK	-0,558758457
GSE40272	X30338	23633	KPNA6	-0,558469873
GSE40272	X22388	283554	GPR137C	-0,558264799
GSE40272	X27552	3241	HPCAL1	-0,558200317
GSE40272	X29955	55719	FAM178A	-0,557819239
GSE40272	X26776	80856	KIAA1715	-0,557607294
GSE40272	X13608	4719	NDUFS1	-0,557364165
GSE40272	X20664	5465	PPARA	-0,557207188
GSE40272	X23405	55073	LRRC37A4	-0,556958774
GSE40272	X9535	6601	SMARCC2	-0,556484672
GSE40272	X30239	6185	RPN2	-0,556348837
GSE40272	X33235	54958	TMEM160	-0,556233087
GSE40272	X27845	3251	HPRT1	-0,556171247
GSE40272	X6510	7067	THRA	-0,556112051
GSE40272	X14990	10365	KLF2	-0,556002643
GSE40272	X17732	23030	KDM4B	-0,555954017
GSE40272	X19780	351	APP	-0,555825053
GSE40272	X39332	9817	KEAP1	-0,555434989
GSE40272	X38876	55827	DCAF6	-0,555088795
GSE40272	X23741	5430	POLR2A	-0,554917019
GSE40272	X17688	64223	MLST8	-0,554900634
GSE40272	X5335	10362	HMG20B	-0,554742072
GSE40272	X20241	23646	PLD3	-0,55457981
GSE40272	X5422	391014	LOC391014	-0,554536469
GSE40272	X41657	57447	NDRG2	-0,554318182
GSE40272	X18802	4924	NUCB1	-0,554087209

GSE40272	X11018	55069	C7orf42	-0,553711416
GSE40272	X10465	5297	PI4KA	-0,553643763
GSE40272	X16882	6625	SNRNP70	-0,553621036
GSE40272	X1386	6928	HNF1B	-0,553598309
GSE40272	X34047	6405	SEMA3F	-0,553528541
GSE40272	X23030	8189	SYMPK	-0,553422833
GSE40272	X5502	158056	MAMDC4	-0,552692389
GSE40272	X10500	351	APP	-0,552681818
GSE40272	X19696	6460	SH3GLP3	-0,552510042
GSE40272	X14998	150372	NFAM1	-0,552281712
GSE40272	X28827	8396	PIP4K2B	-0,552045455
GSE40272	X7135	51548	SIRT6	-0,55180444
GSE40272	X31272	22800	RRAS2	-0,551755285
GSE40272	X13866	147040	KCTD11	-0,551414905
GSE40272	X16743	255057	C19orf26	-0,551126321
GSE40272	X5376	441468	LOC441468	-0,551113636
GSE40272	X25704	10106	CTDSP2	-0,550252643
GSE40272	X2295	57120	GOPC	-0,55005074
GSE40272	X40447	7094	TLN1	-0,550008985
GSE40272	X38788	1628	DBP	-0,549140063
GSE40272	X42253	54622	ARL15	-0,548904334
GSE40272	X41222	79134	TMEM185B	-0,548822939
GSE40272	X28084	7090	TLE3	-0,548597252
GSE40272	X9578	1306	COL15A1	-0,548356237
GSE40272	X6785	4952	OCRL	-0,547331924
GSE40272	X37812	6182	MRPL12	-0,547321882
GSE40272	X31130	4641	MYO1C	-0,547265856
GSE40272	X36458	23499	MACF1	-0,546575581
GSE40272	X23237	9024	BRSK2	-0,546362579
GSE40272	X22372	84304	NUDT22	-0,546317125
GSE40272	X19112	191	AHCY	-0,54607611
GSE40272	X16320	7094	TLN1	-0,545994715
GSE40272	X16586	35	ACADS	-0,545911734
GSE40272	X27652	1453	CSNK1D	-0,545839323
GSE40272	X12605	5430	POLR2A	-0,545444503
GSE40272	X30614	4214	MAP3K1	-0,545060782
GSE40272	X28421	23001	WDFY3	-0,544968816
GSE40272	X24669	60	ACTB	-0,544735201
GSE40272	X27553	29888	STRN4	-0,544593023
GSE40272	X6696	285671	RNF180	-0,544569239
GSE40272	X27691	9563	H6PD	-0,544223044
GSE40272	X31387	390704	LOC390704	-0,544041755
GSE40272	X28140	353324	SPATA12	-0,543985201
GSE40272	X9350	8775	NAPA	-0,543754757
GSE40272	X36459	1174	AP1S1	-0,543357294
GSE40272	X2377	10488	CREB3	-0,542938161
GSE40272	X35773	2621	GAS6	-0,542838795
GSE40272	X26739	51043	ZBTB7B	-0,542582452
GSE40272	X10436	114049	WBSCR22	-0,542513214
GSE40272	X18977	158801	NKAPP1	-0,542207188
GSE40272	X4908	64601	VPS16	-0,541697674
GSE40272	X42411	51154	MRTO4	-0,541654334
GSE40272	X36343	5297	PI4KA	-0,541646406

GSE40272	X37020	84971	ATG4D	-0,54162685
GSE40272	X38087	11333	PDAP1	-0,541255814
GSE40272	X38512	6687	SPG7	-0,541150106
GSE40272	X19844	81567	TXNDC5	-0,540939746
GSE40272	X9983	58508	MLL3	-0,540859408
GSE40272	X13454	6942	TCF20	-0,540796512
GSE40272	X31317	23770	FKBP8	-0,540509514
GSE40272	X6221	122525	C14orf28	-0,540419662
GSE40272	X14682	387266	KRTAP5-3	-0,540180233
GSE40272	X33658	114907	FBXO32	-0,539676533
GSE40272	X41500	83544	DNAL1	-0,539486786
GSE40272	X30773	10401	PIAS3	-0,539417019
GSE40272	X26811	113452	TMEM54	-0,538603066
GSE40272	X11797	8694	DGAT1	-0,538362579
GSE40272	X292	4952	OCRL	-0,538337209
GSE40272	X10991	54802	TRIT1	-0,538057082
GSE40272	X36358	23001	WDFY3	-0,537914905
GSE40272	X9504	60558	GUF1	-0,537709302
GSE40272	X8366	9391	CIAO1	-0,537558668
GSE40272	X5225	6141	RPL18	-0,537526956
GSE40272	X10039	285741	LOC285741	-0,537442389
GSE40272	X30513	29979	UBQLN1	-0,53732981
GSE40272	X21000	23499	MACF1	-0,5372463
GSE40272	X11457	5434	POLR2E	-0,53721723
GSE40272	X4780	375513	GUSBP4	-0,536828224
GSE40272	X35058	286336	FAM78A	-0,536715116
GSE40272	X5717	28964	GIT1	-0,536437104
GSE40272	X20958	5430	POLR2A	-0,536046512
GSE40272	X41232	4952	OCRL	-0,535850423
GSE40272	X35302	60314	C12orf10	-0,535549683
GSE40272	X1487	54785	C17orf59	-0,535086152
GSE40272	X17686	977	CD151	-0,534437104
GSE40272	X3744	10794	ZNF460	-0,53412685
GSE40272	X39224	5528	PPP2R5D	-0,533473573
GSE40272	X22908	8522	GAS7	-0,532165433
GSE40272	X33913	7430	EZR	-0,532163848
GSE40272	X20440	440357	LOC440357	-0,532008985
GSE40272	X22806	64236	PDLIM2	-0,532005814
GSE40272	X10241	5822	PWP2	-0,531992072
GSE40272	X41774	10945	KDELR1	-0,531755814
GSE40272	X2946	54880	BCOR	-0,531519027
GSE40272	X26299	55206	SBNO1	-0,531113636
GSE40272	X22412	6132	RPL8	-0,530903805
GSE40272	X19882	4035	LRP1	-0,530602537
GSE40272	X12585	23145	SSPO	-0,530596195
GSE40272	X33297	9567	GTPBP1	-0,529791226
GSE40272	X29413	6510	SLC1A5	-0,529694503
GSE40272	X18271	90522	YIF1B	-0,529684461
GSE40272	X34395	11101	ATE1	-0,528965116
GSE40272	X21038	5130	PCYT1A	-0,52830074
GSE40272	X42663	65979	PHACTR4	-0,528252643
GSE40272	X8380	441221	LOC441221	-0,528039641
GSE40272	X18953	7094	TLN1	-0,5277537

GSE40272	X26937	51132	RLIM	-0,527210359
GSE40272	X11905	23001	WDFY3	-0,527096723
GSE40272	X39004	4946	OAZ1	-0,526872093
GSE40272	X38807	79068	FTO	-0,526459831
GSE40272	X17643	2987	GUK1	-0,525979387
GSE40272	X38179	54946	SLC41A3	-0,525910148
GSE40272	X502	55227	LRRC1	-0,525813425
GSE40272	X17164	3983	ABLIM1	-0,525728858
GSE40272	X40937	2976	GTF3C2	-0,525719873
GSE40272	X24163	442328	LOC442328	-0,525016385
GSE40272	X2551	7343	UBTF	-0,524122622
GSE40272	X1455	1613	DAPK3	-0,523987315
GSE40272	X27633	80725	SRCIN1	-0,523986786
GSE40272	X34466	284187	DKFZp761P0212	-0,523551268
GSE40272	X27568	6576	SLC25A1	-0,523341966
GSE40272	X41890	284749	LOC284749	-0,523071353
GSE40272	X8895	7094	TLN1	-0,522832981
GSE40272	X13699	57105	CYSLTR2	-0,521696089
GSE40272	X38591	4035	LRP1	-0,521318182
GSE40272	X11695	57720	GPR107	-0,521312368
GSE40272	X40721	57062	DDX24	-0,520983087
GSE40272	X43764	8502	PKP4	-0,520874207
GSE40272	X20331	7260	TSSC1	-0,52017389
GSE40272	X39439	25970	SH2B1	-0,520078224
GSE40272	X42285	4928	NUP98	-0,519995772
GSE40272	X517	2776	GNAQ	-0,519336681
GSE40272	X33563	22913	RALY	-0,518815011
GSE40272	X14907	23499	MACF1	-0,518637421
GSE40272	X38561	400729	LOC400729	-0,518487315
GSE40272	X29286	401607	LOC401607	-0,518267442
GSE40272	X7930	8425	LTBP4	-0,518248943
GSE40272	X30388	5936	RBM4	-0,518211945
GSE40272	X16298	28973	MRPS18B	-0,517326638
GSE40272	X39276	3712	IVD	-0,516719873
GSE40272	X5628	23001	WDFY3	-0,516383192
GSE40272	X38212	4173	MCM4	-0,516258457
GSE40272	X24588	64924	SLC30A5	-0,515714588
GSE40272	X19840	441133	KRT18P38	-0,515628964
GSE40272	X7945	347735	SERINC2	-0,515278013
GSE40272	X10370	84679	SLC9A7	-0,514528013
GSE40272	X19091	8926	SNURF	-0,514526956
GSE40272	X14629	11338	U2AF2	-0,51444926
GSE40272	X22434	6709	SPTAN1	-0,514104123
GSE40272	X30202	10749	KIF1C	-0,513997357
GSE40272	X15269	57666	FBRSL1	-0,513376321
GSE40272	X1554	23499	MACF1	-0,513141121
GSE40272	X35555	4952	OCRL	-0,512469873
GSE40272	X13284	351	APP	-0,511865222
GSE40272	X1597	10521	DDX17	-0,511864693
GSE40272	X3489	1454	CSNK1E	-0,511801268
GSE40272	X26148	81857	MED25	-0,510929704
GSE40272	X11617	886	CCKAR	-0,510757928
GSE40272	X38589	4035	LRP1	-0,51065592

GSE40272	X24216	1975	EIF4B	-0,510111522
GSE40272	X16414	6601	SMARCC2	-0,509267442
GSE40272	X8816	23353	SUN1	-0,50894926
GSE40272	X34403	4849	CNOT3	-0,506915962
GSE40272	X14767	8455	ATRN	-0,505822939
GSE40272	X546	27341	RRP7A	-0,505683404
GSE40272	X5705	2496	FTH1P1	-0,505137949
GSE40272	X25330	118987	PDZD8	-0,504382664
GSE40272	X36036	1523	CUX1	-0,504272727
GSE40272	X20935	388454	LOC388454	-0,504193446
GSE40272	X33997	81	ACTN4	-0,504054968
GSE40272	X15690	5725	PTBP1	-0,503631078
GSE40272	X5192	80772	GLTPD1	-0,503504228
GSE40272	X10848	4054	LTBP3	-0,503337209
GSE40272	X32083	80700	UBXN6	-0,503187104
GSE40272	X4721	6418	SET	-0,503117336
GSE40272	X33327	22839	DLGAP4	-0,502296512
GSE40272	X39034	10314	LANCL1	-0,502221987
GSE40272	X11727	1973	EIF4A1	-0,502001057
GSE40272	X5032	10994	ILVBL	-0,501755814
GSE40272	X1130	390354	RPL18AP3	-0,501727273
GSE40272	X23349	9567	GTPBP1	-0,501376321
GSE40272	X17470	440245	LOC440245	-0,500639006
GSE40272	X14086	116369	SLC26A8	-0,500264271
GSE40272	X41719	22839	DLGAP4	-0,499439218
GSE40272	X4059	60672	MIIP	-0,498706131
GSE40272	X6262	131540	ZDHHC19	-0,498593552
GSE40272	X21400	64320	RNF25	-0,498197674
GSE40272	X39623	283651	HMGN2P46	-0,498179175
GSE40272	X38687	8751	ADAM15	-0,498091438
GSE40272	X6575	2819	GPD1	-0,497986786
GSE40272	X44444	170463	SSBP4	-0,497246829
GSE40272	X19353	64924	SLC30A5	-0,497045455
GSE40272	X12152	162632	LOC162632	-0,496730973
GSE40272	X2198	8932	MBD2	-0,496337209
GSE40272	X8896	5430	POLR2A	-0,496247357
GSE40272	X17337	3609	ILF3	-0,494972516
GSE40272	X43229	7094	TLN1	-0,493886892
GSE40272	X20022	10922	FASTK	-0,493721459
GSE40272	X18463	23295	MGRN1	-0,493639535
GSE40272	X19065	60672	MIIP	-0,493413848
GSE40272	X64	23499	MACF1	-0,493330338
GSE40272	X18733	64319	FBRS	-0,492719345
GSE40272	X24757	23001	WDFY3	-0,492479387
GSE40272	X30160	3609	ILF3	-0,49202907
GSE40272	X24981	5351	PLOD1	-0,49202537
GSE40272	X30441	1119	CHKA	-0,491896406
GSE40272	X31362	23399	CTDNEP1	-0,491582981
GSE40272	X42722	55001	TTC22	-0,491489429
GSE40272	X7926	7536	SF1	-0,491133721
GSE40272	X8456	9830	TRIM14	-0,490599894
GSE40272	X44260	7001	PRDX2	-0,489884249
GSE40272	X13158	9381	OTOF	-0,489707717

GSE40272	X11277	5878	RAB5C	-0,489604651
GSE40272	X31486	1903	S1PR3	-0,489603066
GSE40272	X15604	64423	INF2	-0,489261628
GSE40272	X36114	51386	EIF3L	-0,489178647
GSE40272	X26932	9807	IP6K1	-0,488477273
GSE40272	X27446	11083	DIDO1	-0,488453488
GSE40272	X34348	5395	PMS2	-0,488313953
GSE40272	X5505	51150	SDF4	-0,487689746
GSE40272	X38603	137994	LETM2	-0,487626321
GSE40272	X37385	5297	PI4KA	-0,487516385
GSE40272	X9552	79629	OCEL1	-0,487113108
GSE40272	X21714	6598	SMARCB1	-0,486998943
GSE40272	X27978	440359	CSDAP1	-0,486759514
GSE40272	X26787	10477	UBE2E3	-0,48662685
GSE40272	X19436	80005	DOCK5	-0,486201374
GSE40272	X13939	6618	SNAPC2	-0,485996829
GSE40272	X3609	8525	DGKZ	-0,485803383
GSE40272	X9863	54882	ANKHD1	-0,485225687
GSE40272	X35080	196463	PLBD2	-0,483646406
GSE40272	X18876	5576	PRKAR2A	-0,483337738
GSE40272	X34731	6908	TBP	-0,482922304
GSE40272	X14085	10312	TCIRG1	-0,482463002
GSE40272	X13414	51341	ZBTB7A	-0,482328224
GSE40272	X35902	9777	TM9SF4	-0,48216649
GSE40272	X41829	1059	CENPB	-0,4820037
GSE40272	X9202	55643	BTBD2	-0,48180444
GSE40272	X34971	2039	EPB49	-0,481375264
GSE40272	X38814	143243	LOC143243	-0,480913848
GSE40272	X16601	83862	TMEM120A	-0,480858351
GSE40272	X25110	10174	SORBS3	-0,480591438
GSE40272	X545	400470	LOC400470	-0,480332452
GSE40272	X1362	112770	C1orf85	-0,480091966
GSE40272	X41303	10102	TSFM	-0,479497357
GSE40272	X8888	1742	DLG4	-0,479302854
GSE40272	X26059	4054	LTBP3	-0,479010571
GSE40272	X41919	10494	STK25	-0,478865222
GSE40272	X32379	160	AP2A1	-0,478725159
GSE40272	X12285	389224	LOC389224	-0,47851797
GSE40272	X13187	5780	PTPN9	-0,478454545
GSE40272	X31289	4125	MAN2B1	-0,478326638
GSE40272	X41635	5315	PKM2	-0,478108351
GSE40272	X36552	1198	CLK3	-0,477896406
GSE40272	X21384	10456	HAX1	-0,477859937
GSE40272	X11471	2355	FOSL2	-0,477214588
GSE40272	X12121	11215	AKAP11	-0,476861522
GSE40272	X3877	57805	KIAA1967	-0,476684461
GSE40272	X15468	375748	C9orf102	-0,476233087
GSE40272	X7437	11143	KAT7	-0,476191332
GSE40272	X13455	3098	HK1	-0,47605444
GSE40272	X15941	148022	TICAM1	-0,475919133
GSE40272	X4932	4952	OCRL	-0,474822939
GSE40272	X43519	11167	FSTL1	-0,474154863
GSE40272	X845	2017	CTTN	-0,473489429

GSE40272	X43259	125950	RAVER1	-0,473350423
GSE40272	X35166	1453	CSNK1D	-0,473180761
GSE40272	X24570	54461	FBXW5	-0,472544397
GSE40272	X7716	4952	OCRL	-0,471975159
GSE40272	X25585	1394	CRHR1	-0,471206131
GSE40272	X10244	11100	HNRNPUL1	-0,471205603
GSE40272	X26855	5479	PPIB	-0,471056025
GSE40272	X34849	91355	LRP5L	-0,470849366
GSE40272	X9152	9043	SPAG9	-0,47042019
GSE40272	X30222	7376	NR1H2	-0,469946617
GSE40272	X11774	115557	ARHGEF25	-0,469938689
GSE40272	X1836	389630	FAM90A15	-0,469909619
GSE40272	X5871	9811	CTIF	-0,469677061
GSE40272	X8116	57111	RAB25	-0,469385307
GSE40272	X37663	5430	POLR2A	-0,469339323
GSE40272	X6362	64924	SLC30A5	-0,469128964
GSE40272	X34839	5297	PI4KA	-0,469070296
GSE40272	X27327	283680	LOC283680	-0,469037526
GSE40272	X40552	11337	GABARAP	-0,468969873
GSE40272	X4035	23049	SMG1	-0,468881078
GSE40272	X3366	94239	H2AFV	-0,468831924
GSE40272	X3285	5636	PRPSAP2	-0,468103594
GSE40272	X1451	8148	TAF15	-0,46794926
GSE40272	X8275	23770	FKBP8	-0,467925476
GSE40272	X4560	64419	MTMR14	-0,467162791
GSE40272	X38844	23353	SUN1	-0,466908034
GSE40272	X36084	26061	HACL1	-0,466709302
GSE40272	X20816	310	ANXA7	-0,466553911
GSE40272	X31122	53349	ZFYVE1	-0,466184461
GSE40272	X10712	4134	MAP4	-0,465709302
GSE40272	X33331	811	CALR	-0,465607822
GSE40272	X3325	1509	CTSD	-0,465566068
GSE40272	X6547	79026	AHNAK	-0,465516385
GSE40272	X19021	27430	MAT2B	-0,465062896
GSE40272	X15951	1509	CTSD	-0,464794397
GSE40272	X8862	760	CA2	-0,464516913
GSE40272	X34845	6820	SULT2B1	-0,462187104
GSE40272	X24077	115817	DHRS1	-0,460589323
GSE40272	X22973	11215	AKAP11	-0,459643235
GSE40272	X43952	80700	UBXN6	-0,459510042
GSE40272	X36094	1500	CTNND1	-0,459506342
GSE40272	X3320	5802	PTPRS	-0,459315011
GSE40272	X37368	84818	IL17RC	-0,459064482
GSE40272	X26515	158301	LOC158301	-0,458307611
GSE40272	X13727	338427	SNORD108	-0,457995243
GSE40272	X41963	9394	HS6ST1	-0,457866808
GSE40272	X12527	4294	MAP3K10	-0,457593552
GSE40272	X16487	6712	SPTBN2	-0,457371036
GSE40272	X38893	8668	EIF3I	-0,457240486
GSE40272	X40868	84232	MAF1	-0,457239429
GSE40272	X8386	9409	PEX16	-0,45660148
GSE40272	X769	22839	DLGAP4	-0,456487315
GSE40272	X1446	2776	GNAQ	-0,454965116

GSE40272	X4652	51398	C19orf56	-0,454035412
GSE40272	X5730	3184	HNRNPD	-0,453701374
GSE40272	X26651	79148	MMP28	-0,453420719
GSE40272	X11189	4082	MARCKS	-0,453218816
GSE40272	X33542	10658	CELF1	-0,452821353
GSE40272	X13509	441620	LOC441620	-0,452165962
GSE40272	X2612	1186	CLCN7	-0,452145349
GSE40272	X15012	10280	SIGMAR1	-0,451745243
GSE40272	X8883	103	ADAR	-0,451357294
GSE40272	X16620	142940	TRUB1	-0,451198203
GSE40272	X39703	131909	FAM172BP	-0,450336152
GSE40272	X37974	11277	TREX1	-0,448690803
GSE40272	X38601	7332	UBE2L3	-0,448383192
GSE40272	X23788	1936	EEF1D	-0,446674947
GSE40272	X38867	1967	EIF2B1	-0,446548626
GSE40272	X1776	178	AGL	-0,446309197
GSE40272	X20441	391809	LOC391809	-0,446137949
GSE40272	X7509	441236	LOC441236	-0,44457241
GSE40272	X13973	85014	TMEM141	-0,444448732
GSE40272	X1684	317762	CCDC85C	-0,444349366
GSE40272	X33594	125058	TBC1D16	-0,444267442
GSE40272	X13339	79095	C9orf16	-0,444075053
GSE40272	X32204	51077	FCF1	-0,443400634
GSE40272	X19812	26012	NELF	-0,442102008
GSE40272	X361	440434	LOC440434	-0,441770085
GSE40272	X15006	389	RHOC	-0,441335624
GSE40272	X35502	2120	ETV6	-0,440936047
GSE40272	X37280	2975	GTF3C1	-0,440561839
GSE40272	X6828	56904	SH3GLB2	-0,439739429
GSE40272	X15933	1456	CSNK1G3	-0,439608351
GSE40272	X5860	4952	OCRL	-0,437378964
GSE40272	X38201	10868	USP20	-0,43670296
GSE40272	X4807	4154	MBNL1	-0,436508985
GSE40272	X1470	60	ACTB	-0,436441332
GSE40272	X29928	9685	CLINT1	-0,436087738
GSE40272	X41611	147740	CPLP	-0,434853066
GSE40272	X31851	3006	HIST1H1C	-0,434633192
GSE40272	X33385	11140	CDC37	-0,433739958
GSE40272	X17691	116985	ARAP1	-0,433645349
GSE40272	X19945	5252	PHF1	-0,432611522
GSE40272	X27650	23196	FAM120A	-0,432463531
GSE40272	X38339	4952	OCRL	-0,431722516
GSE40272	X39939	91368	CDKN2AIPNL	-0,43157611
GSE40272	X6427	196513	DCP1B	-0,431394292
GSE40272	X11839	6711	SPTBN1	-0,431027484
GSE40272	X20684	9043	SPAG9	-0,43093129
GSE40272	X13755	441597	LOC441597	-0,430819767
GSE40272	X9107	23513	SCRIB	-0,430436047
GSE40272	X41099	6711	SPTBN1	-0,430192389
GSE40272	X13690	4134	MAP4	-0,429102008
GSE40272	X37936	25873	RPL36	-0,427951374
GSE40272	X32260	7343	UBTF	-0,427543869
GSE40272	X43655	84284	NTPCR	-0,427502643

GSE40272	X42501	1636	ACE	-0,427262685
GSE40272	X20518	84193	SETD3	-0,426628964
GSE40272	X6077	11054	OGFR	-0,426268499
GSE40272	X2483	23001	WDFY3	-0,426218288
GSE40272	X40973	4826	NNAT	-0,424858351
GSE40272	X32120	1173	AP2M1	-0,424496829
GSE40272	X8904	441619	LOC441619	-0,424379493
GSE40272	X26828	10809	STARD10	-0,423102537
GSE40272	X6156	10762	NUP50	-0,422932347
GSE40272	X20880	5297	PI4KA	-0,42214482
GSE40272	X16469	23143	LRCH1	-0,422077696
GSE40272	X34837	23476	BRD4	-0,42147093
GSE40272	X32455	57532	NUFIP2	-0,420821882
GSE40272	X8644	4952	OCRL	-0,420307611
GSE40272	X24630	10558	SPTLC1	-0,41982241
GSE40272	X24680	6458	SH3GL1P1	-0,418871036
GSE40272	X4671	389863	LOC389863	-0,418006871
GSE40272	X878	22820	COPG	-0,415945032
GSE40272	X17051	79735	TBC1D17	-0,415715645
GSE40272	X42562	2990	GUSB	-0,41459778
GSE40272	X26189	1975	EIF4B	-0,41330444
GSE40272	X4702	10521	DDX17	-0,413068182
GSE40272	X7484	51657	STYXL1	-0,411131078
GSE40272	X19888	310	ANXA7	-0,410603594
GSE40272	X32220	57605	PITPNM2	-0,410309197
GSE40272	X8915	26173	INTS1	-0,410285412
GSE40272	X24064	84266	ALKBH7	-0,409052326
GSE40272	X38101	3029	HAGH	-0,407218816
GSE40272	X587	388739	ZC3H11B	-0,405450317
GSE40272	X43562	170394	PWWP2B	-0,405266385
GSE40272	X30946	84163	GTF2IRD2	-0,404126321
GSE40272	X23688	682	BSG	-0,402299683
GSE40272	X25065	904	CCNT1	-0,402266913
GSE40272	X5379	8733	GPAA1	-0,401872622
GSE40272	X9980	85403	EAF1	-0,401536469
GSE40272	X31214	27341	RRP7A	-0,401374207
GSE40272	X1286	23499	MACF1	-0,40115592
GSE40272	X38631	9665	KIAA0430	-0,40014852
GSE40272	X37942	4771	NF2	-0,397579281
GSE40272	X28255	253260	RICTOR	-0,395801797
GSE40272	X38395	139431	FTH1P14	-0,394008457
GSE40272	X30068	2969	GTF2I	-0,391912262
GSE40272	X4964	23648	SSBP3	-0,391618393
GSE40272	X28104	5594	MAPK1	-0,390139006
GSE40272	X2174	738	C11orf2	-0,389595666
GSE40272	X22901	23499	MACF1	-0,389508457
GSE40272	X38813	390533	LOC390533	-0,388414376
GSE40272	X38873	57488	ESYT2	-0,387971459
GSE40272	X38204	10618	TGOLN2	-0,387419133
GSE40272	X7045	284064	RPL29P31	-0,385052854
GSE40272	X25209	4784	NFIX	-0,384183404
GSE40272	X20876	57698	KIAA1598	-0,383764271
GSE40272	X42122	8918	HERC2P6	-0,38309408

GSE40272	X43720	23558	WBP2	-0,382418076
GSE40272	X4237	5566	PRKACA	-0,382250529
GSE40272	X14888	55765	C1orf106	-0,38173203
GSE40272	X31384	10160	FARP1	-0,380901163
GSE40272	X27557	7178	TPT1	-0,380243658
GSE40272	X16993	4952	OCRL	-0,379622093
GSE40272	X20121	25792	CIZ1	-0,378943446
GSE40272	X14008	6142	RPL18A	-0,376967759
GSE40272	X28185	79171	RBM42	-0,374647463
GSE40272	X35506	387522	TMEM189-UBE2V1	-0,373588795
GSE40272	X26744	374291	NDUFS7	-0,372687632
GSE40272	X1773	2872	MKNK2	-0,37171723
GSE40272	X28482	1452	CSNK1A1	-0,371090381
GSE40272	X7500	4713	NDUFB7	-0,370407505
GSE40272	X27393	8079	MLF2	-0,369747357
GSE40272	X11193	4782	NFIC	-0,369507928
GSE40272	X28384	5430	POLR2A	-0,369498943
GSE40272	X31439	55611	OTUB1	-0,367524841
GSE40272	X27922	339077	LOC339077	-0,366088795
GSE40272	X25208	6598	SMARCB1	-0,365944503
GSE40272	X4259	54998	AURKAIP1	-0,364943975
GSE40272	X17562	513	ATP5D	-0,364146406
GSE40272	X31587	4782	NFIC	-0,359035412
GSE40272	X988	54882	ANKHD1	-0,356963531
GSE40272	X10308	6430	SRSF5	-0,353471459
GSE40272	X22776	23499	MACF1	-0,351967759
GSE40272	X37577	831	CAST	-0,351505814
GSE40272	X38026	26220	DGCR5	-0,351504757
GSE40272	X30771	79090	TRAPPC6A	-0,347726216
GSE40272	X40546	527	ATP6V0C	-0,346060254
GSE40272	X42110	2767	GNA11	-0,34509778
GSE40272	X5497	54461	FBXW5	-0,344050211
GSE40272	X33548	6830	SUPT6H	-0,343803911
GSE40272	X13194	8815	BANF1	-0,343396934
GSE40272	X18702	51282	SCAND1	-0,338220402
GSE40272	X23418	9817	KEAP1	-0,337983615
GSE40272	X34373	92714	ARRDC1	-0,335906977
GSE40272	X32140	147687	ZNF417	-0,331207188
GSE40272	X27064	28974	C19orf53	-0,3277426
GSE70768	ILMN_1722713	2192	FBLN1	-0,705905423
GSE70768	ILMN_1664464	5730	PTGDS	-0,699680137
GSE70768	ILMN_1677636	1311	COMP	-0,694612953
GSE70768	ILMN_1700541	2192	FBLN1	-0,685261012
GSE70768	ILMN_3230608	728841	NBPF8	-0,645852254
GSE70768	ILMN_1745607	2	A2M	-0,638612926
GSE70768	ILMN_1670305	710	SERPING1	-0,630403793
GSE70768	ILMN_1774287	629	CFB	-0,624864363
GSE70768	ILMN_1669362	3489	IGFBP6	-0,624562152
GSE70768	ILMN_1740938	348	APOE	-0,615886873
GSE70768	ILMN_1783909	1292	COL6A2	-0,610793516
GSE70768	ILMN_2115490	400818	NBPF20	-0,610342342
GSE70768	ILMN_1788874	12	SERPINA3	-0,603101172
GSE70768	ILMN_2359945	1066	CES1	-0,599701522

GSE70768	ILMN_2380237	114897	C1QTNF1	-0,583260843
GSE70768	ILMN_1665219	8425	LTBP4	-0,568794478
GSE70768	ILMN_1723684	2532	DARC	-0,55876952
GSE70768	ILMN_1717990	800	CALD1	-0,549076459
GSE70768	ILMN_2193325	8510	MMP23B	-0,546128207
GSE70768	ILMN_2379644	972	CD74	-0,546110962
GSE70768	ILMN_2206746	633	BGN	-0,545679023
GSE70768	ILMN_1747593	3671	ISLR	-0,534617367
GSE70768	ILMN_1715748	2318	FLNC	-0,533465552
GSE70768	ILMN_1677198	715	C1R	-0,529459715
GSE70768	ILMN_1773389	5360	PLTP	-0,527582737
GSE70768	ILMN_1736567	972	CD74	-0,516908787
GSE70768	ILMN_1679401	54795	TRPM4	-0,516115709
GSE70768	ILMN_1738116	338773	TMEM119	-0,511129186
GSE70768	ILMN_1795484	5225	PGC	-0,510713228
GSE70768	ILMN_1714861	968	CD68	-0,510511785
GSE70768	ILMN_1736178	165	AEBP1	-0,505332645
GSE70768	ILMN_3228294	729279	LOC729279	-0,500382911
GSE70768	ILMN_1690034	6649	SOD3	-0,490840603
GSE70768	ILMN_1780170	347	APOD	-0,489945972
GSE70768	ILMN_1769129	6363	CCL19	-0,488278383
GSE70768	ILMN_1781149	11185	INMT	-0,485005573
GSE70768	ILMN_1660306	8516	ITGA8	-0,483506617
GSE70768	ILMN_2115125	1490	CTGF	-0,483299792
GSE70768	ILMN_1789733	25999	CLIP3	-0,482536783
GSE70768	ILMN_1721283	126393	HSPB6	-0,474982736
GSE70768	ILMN_1742450	6892	TAPBP	-0,473676367
GSE70768	ILMN_1688780	6275	S100A4	-0,472128063
GSE70768	ILMN_2098126	6352	CCL5	-0,471751036
GSE70768	ILMN_1704286	53827	FXYD5	-0,470056184
GSE70768	ILMN_1704985	1593	CYP27A1	-0,467829778
GSE70768	ILMN_1684306	6275	S100A4	-0,46773109
GSE70768	ILMN_3192001	6358	CCL14	-0,467200783
GSE70768	ILMN_1743620	5918	RARRES1	-0,465577791
GSE70768	ILMN_2309848	53827	FXYD5	-0,465388954
GSE70768	ILMN_1701613	5920	RARRES3	-0,463195763
GSE70768	ILMN_2376205	4050	LTB	-0,458594389
GSE70768	ILMN_1745623	283229	EFCAB4A	-0,458036918
GSE70768	ILMN_3302499	730990	LOC730990	-0,45391822
GSE70768	ILMN_3225102	148203	ZNF738	-0,450017275
GSE70768	ILMN_1687335	2316	FLNA	-0,449792461
GSE70768	ILMN_1813561	85477	SCIN	-0,445612691
GSE70768	ILMN_1756071	4240	MFGE8	-0,443346008
GSE70768	ILMN_2066060	3128	HLA-DRB6	-0,440567472
GSE70768	ILMN_1669599	55667	DENND4C	-0,439117067
GSE70768	ILMN_1772359	7805	LAPTM5	-0,437963047
GSE70768	ILMN_1763891	284297	FLJ35258	-0,43641478
GSE70768	ILMN_2180239	9980	DOPEY2	-0,435580086
GSE70768	ILMN_2387471	80164	FLJ22184	-0,435005369
GSE70768	ILMN_2109708	1890	ECGF1	-0,434467243
GSE70768	ILMN_1773352	6352	CCL5	-0,434108355
GSE70768	ILMN_1790859	219348	PLAC9	-0,433668215
GSE70768	ILMN_3213185	645452	LOC645452	-0,431156572

GSE70768	ILMN_2155719	440673	NBPF10	-0,431059797
GSE70768	ILMN_3181480	338999	FLJ36131	-0,430286415
GSE70768	ILMN_3274790	648921	LOC648921	-0,429794302
GSE70768	ILMN_1707070	5118	PCOLCE	-0,42899706
GSE70768	ILMN_1774982	148170	CDC42EP5	-0,42837969
GSE70768	ILMN_3231550	100131718	LOC100131718	-0,426120553
GSE70768	ILMN_1674063	4939	OAS2	-0,42450284
GSE70768	ILMN_3243238	64499	TPSB2	-0,422499444
GSE70768	ILMN_2216265	83752	LONP2	-0,42201193
GSE70768	ILMN_1786847	7053	TGM3	-0,421106483
GSE70768	ILMN_1769779	11156	PTP4A3	-0,420898713
GSE70768	ILMN_2066066	3128	HLA-DRB6	-0,42029808
GSE70768	ILMN_1672536	2192	FBLN1	-0,419754771
GSE70768	ILMN_2189222	57563	KLHL8	-0,418834331
GSE70768	ILMN_1662340	140467	ZNF358	-0,418812438
GSE70768	ILMN_1752592	3126	HLA-DRB4	-0,417632351
GSE70768	ILMN_1676256	7177	TPSAB1	-0,417554353
GSE70768	ILMN_2075794	126205	NLRP8	-0,413863942
GSE70768	ILMN_1746919	643882	LOC643882	-0,41247692
GSE70768	ILMN_2411282	5768	QSOX1	-0,410671981
GSE70768	ILMN_3228688	730415	LOC730415	-0,406604456
GSE70768	ILMN_2334296	10068	IL18BP	-0,406494499
GSE70768	ILMN_3215461	100131989	LOC100131989	-0,406148574
GSE70768	ILMN_1760778	2022	ENG	-0,406064757
GSE70768	ILMN_2326712	25825	BACE2	-0,405730453
GSE70768	ILMN_1740609	6359	CCL15	-0,404934626
GSE70768	ILMN_1770290	1265	CNN2	-0,40451607
GSE70768	ILMN_1661335	6711	SPTBN1	-0,40406868
GSE70768	ILMN_1803810	6238	RRBP1	-0,401136703
GSE70768	ILMN_1654396	3689	ITGB2	-0,400887167
GSE70768	ILMN_2207505	3952	LEP	-0,400211187
GSE70768	ILMN_1805377	7784	ZP3	-0,400145815
GSE70768	ILMN_1801307	8743	TNFSF10	-0,39955637
GSE70768	ILMN_1770800	127435	PODN	-0,399200285
GSE70768	ILMN_1766914	4239	MFAP4	-0,397891102
GSE70768	ILMN_3287996	400446	LOC400446	-0,397144713
GSE70768	ILMN_1680948	25802	LMOD1	-0,396820399
GSE70768	ILMN_3247256	646996	LOC646996	-0,396808809
GSE70768	ILMN_1809928	1292	COL6A2	-0,396467985
GSE70768	ILMN_1699829	1490	CTGF	-0,396190222
GSE70768	ILMN_1663080	3955	LFNG	-0,396087704
GSE70768	ILMN_1687384	2537	IFI6	-0,394784306
GSE70768	ILMN_1673421	440704	LOC440704	-0,394444548
GSE70768	ILMN_2404154	5265	SERPINA1	-0,393959695
GSE70768	ILMN_2262044	84875	PARP10	-0,393928951
GSE70768	ILMN_1774602	2199	FBLN2	-0,393491536
GSE70768	ILMN_1764788	7133	TNFRSF1B	-0,393203835
GSE70768	ILMN_1815895	649143	LOC649143	-0,393202517
GSE70768	ILMN_1677200	26999	CYFIP2	-0,39314924
GSE70768	ILMN_2089175	94056	SYAP1	-0,392260567
GSE70768	ILMN_1801040	6693	SPN	-0,392145271
GSE70768	ILMN_3243175	100132727	LOC100132727	-0,391515005
GSE70768	ILMN_2359710	11156	PTP4A3	-0,391453464

GSE70768	ILMN_1761464	972	CD74	-0,391277637
GSE70768	ILMN_1734742	396	ARHGDIA	-0,390903027
GSE70768	ILMN_1667748	341405	ANKRD33	-0,390151188
GSE70768	ILMN_1680279	25862	USP49	-0,389708937
GSE70768	ILMN_1673363	976	CD97	-0,389684116
GSE70768	ILMN_2396272	27250	PDCD4	-0,389628395
GSE70768	ILMN_1803855	23017	FAIM2	-0,389579086
GSE70768	ILMN_2162367	11144	DMC1	-0,388760755
GSE70768	ILMN_1711073	653489	LOC653489	-0,388053174
GSE70768	ILMN_1768812	53826	FXYD6	-0,387016654
GSE70768	ILMN_2389876	7041	TGFB1I1	-0,38697817
GSE70768	ILMN_1686623	1436	CSF1R	-0,386709796
GSE70768	ILMN_1675130	4782	NFIC	-0,385680408
GSE70768	ILMN_1754220	8175	SF3A2	-0,385508147
GSE70768	ILMN_2286014	117155	CATSPER2	-0,385391413
GSE70768	ILMN_1651610	730525	LOC730525	-0,383965633
GSE70768	ILMN_3294213	401098	LOC401098	-0,383481751
GSE70768	ILMN_1760027	7454	WAS	-0,383087519
GSE70768	ILMN_1665865	3487	IGFBP4	-0,38294846
GSE70768	ILMN_1685433	1295	COL8A1	-0,38214714
GSE70768	ILMN_1779875	7070	THY1	-0,380830172
GSE70768	ILMN_1726666	2878	GPX3	-0,380344984
GSE70768	ILMN_1790052	8511	MMP23A	-0,37991266
GSE70768	ILMN_1764109	715	C1R	-0,379783874
GSE70768	ILMN_1654072	6376	CX3CL1	-0,379702701
GSE70768	ILMN_3239135	100132391	LOC100132391	-0,379621281
GSE70768	ILMN_3294222	100132673	LOC100132673	-0,379338626
GSE70768	ILMN_3176146	100128918	LOC100128918	-0,378295934
GSE70768	ILMN_1788180	5872	RAB13	-0,378080771
GSE70768	ILMN_3265143	100129502	LOC100129502	-0,377384029
GSE70768	ILMN_1669323	25825	BACE2	-0,376533104
GSE70768	ILMN_2113535	51449	PCYOX1	-0,374575886
GSE70768	ILMN_2169801	7177	TPSAB1	-0,373973043
GSE70768	ILMN_1703650	10318	TNIP1	-0,373567509
GSE70768	ILMN_2186806	3134	HLA-F	-0,372849865
GSE70768	ILMN_1772644	256364	EML3	-0,372026405
GSE70768	ILMN_1789338	10174	SORBS3	-0,371633736
GSE70768	ILMN_1709795	5880	RAC2	-0,371536383
GSE70768	ILMN_2368530	9235	IL32	-0,371073596
GSE70768	ILMN_1727532	56944	OLFML3	-0,370325569
GSE70768	ILMN_1767446	57484	RNF150	-0,369949903
GSE70768	ILMN_1796409	713	C1QB	-0,369651754
GSE70768	ILMN_2224907	201895	C4orf34	-0,369608004
GSE70768	ILMN_3294134	389765	LOC389765	-0,369432688
GSE70768	ILMN_2367707	5585	PKN1	-0,369274057
GSE70768	ILMN_1812105	8396	PIP5K2B	-0,368874012
GSE70768	ILMN_2376458	1438	CSF2RA	-0,368390671
GSE70768	ILMN_2249473	10558	SPTLC1	-0,367797579
GSE70768	ILMN_2383107	27072	VPS41	-0,367605546
GSE70768	ILMN_1687848	730	C7	-0,367216344
GSE70768	ILMN_1695311	3108	HLA-DMA	-0,367046512
GSE70768	ILMN_1705750	7052	TGM2	-0,36685622
GSE70768	ILMN_2359742	1508	CTSB	-0,366818313

GSE70768	ILMN_1808777	30846	EHD2	-0,366249866
GSE70768	ILMN_1677691	648852	LOC648852	-0,365700816
GSE70768	ILMN_1671295	83643	CCDC3	-0,365540424
GSE70768	ILMN_1725534	81	ACTN4	-0,364857018
GSE70768	ILMN_1717052	10809	STARD10	-0,364744884
GSE70768	ILMN_1796316	4318	MMP9	-0,364491335
GSE70768	ILMN_3249658	100134868	LOC100134868	-0,364350357
GSE70768	ILMN_2107004	2825	GPR1	-0,364092783
GSE70768	ILMN_1651832	10938	EHD1	-0,364017769
GSE70768	ILMN_1752755	7450	VWF	-0,363911746
GSE70768	ILMN_1664068	57222	ERGIC1	-0,363451556
GSE70768	ILMN_1694731	1186	CLCN7	-0,363338396
GSE70768	ILMN_2374352	79007	DBNDD1	-0,362811776
GSE70768	ILMN_1772981	29924	EPN1	-0,362717129
GSE70768	ILMN_1662358	4599	MX1	-0,362322291
GSE70768	ILMN_1654320	644250	LOC644250	-0,361823876
GSE70768	ILMN_1693789	250	ALPP	-0,361456776
GSE70768	ILMN_1751851	51816	CECR1	-0,36078365
GSE70768	ILMN_2085862	51296	SLC15A3	-0,360710514
GSE70768	ILMN_2363392	8740	TNFSF14	-0,36037865
GSE70768	ILMN_1800451	10025	MED16	-0,360065274
GSE70768	ILMN_2175912	3689	ITGB2	-0,35981408
GSE70768	ILMN_2048811	80224	NUBPL	-0,35938372
GSE70768	ILMN_1781626	716	C1S	-0,358916162
GSE70768	ILMN_2359907	968	CD68	-0,358741876
GSE70768	ILMN_3249244	113277	TMEM106A	-0,358193638
GSE70768	ILMN_1678841	10537	UBD	-0,357668571
GSE70768	ILMN_2141482	5176	SERPINF1	-0,357036634
GSE70768	ILMN_2053921	832	CAPZB	-0,356975857
GSE70768	ILMN_1814039	653086	LOC653086	-0,356202854
GSE70768	ILMN_1762204	148823	C1orf150	-0,356160406
GSE70768	ILMN_3251723	87178	PNPT1	-0,356070274
GSE70768	ILMN_1699931	10870	HCST	-0,35606774
GSE70768	ILMN_2196078	114836	SLAMF6	-0,354322538
GSE70768	ILMN_2392286	9807	IP6K1	-0,353262845
GSE70768	ILMN_1728684	27043	PELP1	-0,353001287
GSE70768	ILMN_3203444	100132535	LOC100132535	-0,35260008
GSE70768	ILMN_1789639	2331	FMOD	-0,352313283
GSE70768	ILMN_1703316	255783	LOC255783	-0,351116245
GSE70768	ILMN_2222101	55728	N4BP2	-0,350808619
GSE70768	ILMN_1778374	682	BSG	-0,350333654
GSE70768	ILMN_2345292	5348	FXYD1	-0,350136294
GSE70768	ILMN_1776519	5909	RAP1GAP	-0,349720975
GSE70768	ILMN_3238053	100129211	LOC100129211	-0,349567532
GSE70768	ILMN_2117809	503632	DUXAP3	-0,34894065
GSE70768	ILMN_1790782	10025	MED16	-0,34843072
GSE70768	ILMN_1715583	23246	BOP1	-0,348132643
GSE70768	ILMN_3237404	100132585	LOC100132585	-0,347472793
GSE70768	ILMN_2252309	29952	DPP7	-0,347217293
GSE70768	ILMN_2130180	283345	RPL13L	-0,34619323
GSE70768	ILMN_1701877	558	AXL	-0,345612942
GSE70768	ILMN_2380938	9066	SYT7	-0,345558099
GSE70768	ILMN_2405684	330	BIRC3	-0,345242507

GSE70768	ILMN_2402392	1295	COL8A1	-0,344946313
GSE70768	ILMN_2390919	2199	FBLN2	-0,34420311
GSE70768	ILMN_2407851	54756	IL17RD	-0,342565055
GSE70768	ILMN_2054554	285605	DTWD2	-0,342406791
GSE70768	ILMN_2183687	54923	LIME1	-0,342245474
GSE70768	ILMN_1713829	9536	PTGES	-0,342065974
GSE70768	ILMN_3281039	642909	LOC642909	-0,3412454
GSE70768	ILMN_1731353	79586	CHPF	-0,341056288
GSE70768	ILMN_1692956	643949	LOC643949	-0,340816907
GSE70768	ILMN_1744604	1535	CYBA	-0,340732051
GSE70768	ILMN_1767556	11067	C10orf10	-0,340614679
GSE70768	ILMN_1680134	10498	CARM1	-0,340395073
GSE70768	ILMN_1709439	5119	CHMP1A	-0,339672662
GSE70768	ILMN_1675709	55738	ARFGAP1	-0,339158946
GSE70768	ILMN_1801043	2934	GSN	-0,337492283
GSE70768	ILMN_2299450	22953	P2RX2	-0,337360886
GSE70768	ILMN_3239445	100128927	ZBTB42	-0,337335677
GSE70768	ILMN_2313889	91120	ZNF682	-0,33636043
GSE70768	ILMN_1673069	91039	DPP9	-0,33571496
GSE70768	ILMN_1690114	401494	PTPLAD2	-0,33567633
GSE70768	ILMN_3211132	645173	LOC645173	-0,335522827
GSE70768	ILMN_1711439	11117	EMILIN1	-0,334691499
GSE70768	ILMN_3200362	727908	LOC727908	-0,334258627
GSE70768	ILMN_1713749	11151	CORO1A	-0,334099033
GSE70768	ILMN_2327860	4118	MAL	-0,333189679
GSE70768	ILMN_2055634	9220	TIAF1	-0,333175486
GSE70768	ILMN_2122511	147372	CCBE1	-0,332415058
GSE70768	ILMN_1721349	84061	MAGT1	-0,332273599
GSE70768	ILMN_3242038	493869	GPX8	-0,331699992
GSE70768	ILMN_1777794	5589	PRKCSH	-0,331528915
GSE70768	ILMN_3223500	728903	LOC728903	-0,331411179
GSE70768	ILMN_2415979	85452	KIAA1751	-0,330779263
GSE70768	ILMN_2077094	738	C11orf2	-0,330589541
GSE70768	ILMN_2371169	7791	ZYX	-0,330561155
GSE70768	ILMN_2123559	374986	FAM73A	-0,330218484
GSE70768	ILMN_1730670	10272	FSTL3	-0,329823936
GSE70768	ILMN_2329735	1893	ECM1	-0,329499649
GSE70768	ILMN_3261345	100130053	LOC100130053	-0,329324525
GSE70768	ILMN_2342455	27068	PPA2	-0,329302999
GSE70768	ILMN_1769508	54965	PIGX	-0,329191674
GSE70768	ILMN_1684554	1307	COL16A1	-0,32908048
GSE70768	ILMN_1785528	374666	FAM39DP	-0,328717964
GSE70768	ILMN_3240838	293	SLC25A6	-0,328672997
GSE70768	ILMN_1651788	4296	MAP3K11	-0,32821412
GSE70768	ILMN_1656287	9806	SPOCK2	-0,327862345
GSE70768	ILMN_2129161	2615	LRRC32	-0,327654501
GSE70768	ILMN_3246255	100133516	LOC100133516	-0,326584103
GSE70768	ILMN_1745374	3430	IFI35	-0,326430693
GSE70768	ILMN_1739441	23193	GANAB	-0,326123416
GSE70768	ILMN_1754103	5010	CLDN11	-0,325811109
GSE70768	ILMN_2041222	284085	FLJ40504	-0,325686115
GSE70768	ILMN_2059173	79939	SLC35E1	-0,325665229
GSE70768	ILMN_1727402	3059	HCLS1	-0,325470086

GSE70768	ILMN_3239629	729679	LOC729679	-0,32538949
GSE70768	ILMN_1669114	65266	WNK4	-0,325373515
GSE70768	ILMN_2053538	54933	RHBDL2	-0,325297399
GSE70768	ILMN_1655952	375690	FAM39E	-0,324426147
GSE70768	ILMN_1806122	57680	CHD8	-0,324259105
GSE70768	ILMN_1653203	30008	EFEMP2	-0,323845722
GSE70768	ILMN_1701655	80024	SLC24A6	-0,322515648
GSE70768	ILMN_2381537	27297	CRCP	-0,322095341
GSE70768	ILMN_3239361	100133298	LOC100133298	-0,321975218
GSE70769	ILMN_1660086	4629	MYH11	-1,005844456
GSE70769	ILMN_1766914	4239	MFAP4	-0,780085388
GSE70769	ILMN_2368834	4629	MYH11	-0,768319741
GSE70769	ILMN_1713995	6337	SCNN1A	-0,756332207
GSE70769	ILMN_1717990	800	CALD1	-0,743563999
GSE70769	ILMN_1792363	9622	KLK4	-0,738149104
GSE70769	ILMN_1665219	8425	LTBP4	-0,692025407
GSE70769	ILMN_1721876	7077	TIMP2	-0,649155663
GSE70769	ILMN_1688642	10319	LAMC3	-0,604777275
GSE70769	ILMN_1808587	463	ZFHX3	-0,60195566
GSE70769	ILMN_1789733	25999	CLIP3	-0,599353997
GSE70769	ILMN_2099301	25777	UNC84B	-0,595504409
GSE70769	ILMN_1670305	710	SERPING1	-0,586634012
GSE70769	ILMN_1715748	2318	FLNC	-0,582499261
GSE70769	ILMN_3241234	730278	LOC730278	-0,570383555
GSE70769	ILMN_1795300	389337	FLJ41603	-0,557746474
GSE70769	ILMN_1776953	10398	MYL9	-0,556873169
GSE70769	ILMN_1674236	3315	HSPB1	-0,555831781
GSE70769	ILMN_1772981	29924	EPN1	-0,55330607
GSE70769	ILMN_1732060	392	ARHGAP1	-0,541806343
GSE70769	ILMN_1777794	5589	PRKCSH	-0,539807203
GSE70769	ILMN_1808238	348093	RBPMS2	-0,534384294
GSE70769	ILMN_1674533	55503	TRPV6	-0,529873918
GSE70769	ILMN_1721283	126393	HSPB6	-0,523386589
GSE70769	ILMN_1752968	3913	LAMB2	-0,521408951
GSE70769	ILMN_1719543	4094	MAF	-0,514533262
GSE70769	ILMN_1768510	23324	MAN2B2	-0,507559274
GSE70769	ILMN_1701875	7791	ZYX	-0,507340974
GSE70769	ILMN_1675062	10398	MYL9	-0,500331762
GSE70769	ILMN_2352097	9289	GPR56	-0,494878672
GSE70769	ILMN_1773567	3911	LAMA5	-0,492358769
GSE70769	ILMN_2308582	1727	CYB5R3	-0,480851398
GSE70769	ILMN_1811702	2896	GRN	-0,480678731
GSE70769	ILMN_1751851	51816	CECR1	-0,478052362
GSE70769	ILMN_1770290	1265	CNN2	-0,473767397
GSE70769	ILMN_2347097	55554	KLK15	-0,471588299
GSE70769	ILMN_1729482	55554	KLK15	-0,46917006
GSE70769	ILMN_1738401	2296	FOXC1	-0,467002368
GSE70769	ILMN_1781983	162	AP1B1	-0,465625903
GSE70769	ILMN_1748281	5602	MAPK10	-0,464674504
GSE70769	ILMN_1763228	4209	MEF2D	-0,461865306
GSE70769	ILMN_3246900	92249	LOC92249	-0,460165728
GSE70769	ILMN_1702501	6196	RPS6KA2	-0,456215507
GSE70769	ILMN_1786197	7025	NR2F1	-0,456204268

GSE70769	ILMN_1651315	10362	HMG20B	-0,450258424
GSE70769	ILMN_1722634	4924	NUCB1	-0,433319659
GSE70769	ILMN_1696911	2501	FTHL8	-0,432941086
GSE70769	ILMN_1672504	8566	PDXK	-0,429529548
GSE70769	ILMN_1750101	6282	S100A11	-0,428850591
GSE70769	ILMN_1783566	79748	LMAN1L	-0,42610063
GSE70769	ILMN_1679919	84164	ASCC2	-0,422542472
GSE70769	ILMN_1679797	104	ADARB1	-0,418748754
GSE70769	ILMN_2130635	80020	FOXRED2	-0,417808595
GSE70769	ILMN_1751097	64764	CREB3L2	-0,417668651
GSE70769	ILMN_1757552	284119	PTRF	-0,417010668
GSE70769	ILMN_1731353	79586	CHPF	-0,412658794
GSE70769	ILMN_1729234	1200	TPP1	-0,412393896
GSE70769	ILMN_2308849	91663	MYADM	-0,409859017
GSE70769	ILMN_1779171	9905	SGSM2	-0,409366486
GSE70769	ILMN_1719986	113791	PIK3IP1	-0,409298573
GSE70769	ILMN_1666976	23646	PLD3	-0,409013872
GSE70769	ILMN_1689456	26137	ZBTB20	-0,402931956
GSE70769	ILMN_1725534	81	ACTN4	-0,399668272
GSE70769	ILMN_1737833	1822	ATN1	-0,398769667
GSE70769	ILMN_1666376	81844	TRIM56	-0,39701323
GSE70769	ILMN_1660341	4043	LRPAP1	-0,392264327
GSE70769	ILMN_1724250	2896	GRN	-0,391943019
GSE70769	ILMN_1759023	7466	WFS1	-0,390389239
GSE70769	ILMN_1803219	147798	TMC4	-0,389218565
GSE70769	ILMN_1662198	5905	RANGAP1	-0,387188265
GSE70769	ILMN_1782292	3916	LAMP1	-0,386764549
GSE70769	ILMN_1740441	1727	CYB5R3	-0,385049631
GSE70769	ILMN_1743763	93643	TJAP1	-0,383463887
GSE70769	ILMN_2380237	114897	C1QTNF1	-0,379927908
GSE70769	ILMN_1694731	1186	CLCN7	-0,378272632
GSE70769	ILMN_1659206	5914	RARA	-0,377876277
GSE70769	ILMN_1727574	152485	ZNF827	-0,37688534
GSE70769	ILMN_1652797	400451	FAM174B	-0,375829325
GSE70769	ILMN_1655952	375690	FAM39E	-0,374627748
GSE70769	ILMN_1687921	339123	JMJD8	-0,374204529
GSE70769	ILMN_1708143	8933	FAM127A	-0,373933065
GSE70769	ILMN_1806030	5493	PPL	-0,372738539
GSE70769	ILMN_2359742	1508	CTSB	-0,372087651
GSE70769	ILMN_2400874	57410	SCYL1	-0,370452963
GSE70769	ILMN_1668514	23396	PIP5K1C	-0,369693553
GSE70769	ILMN_1779185	23384	CYTSA	-0,36919643
GSE70769	ILMN_1704793	339344	MYPOP	-0,368801032
GSE70769	ILMN_1800425	6548	SLC9A1	-0,368386481
GSE70769	ILMN_2389876	7041	TGFB1I1	-0,368308537
GSE70769	ILMN_3249366	339123	JMJD8	-0,368229394
GSE70769	ILMN_1696027	642333	LOC642333	-0,366292656
GSE70769	ILMN_1749478	85012	TCEAL3	-0,364782934
GSE70769	ILMN_1682567	29903	CCDC106	-0,363563139
GSE70769	ILMN_1800354	1471	CST3	-0,362898144
GSE70769	ILMN_1667748	341405	ANKRD33	-0,358124163
GSE70769	ILMN_1667883	8563	THOC5	-0,357762955
GSE70769	ILMN_2371169	7791	ZYX	-0,357264715

GSE70769	ILMN_1763447	23654	PLXNB2	-0,356599769
GSE70769	ILMN_1763198	6778	STAT6	-0,355708545
GSE70769	ILMN_1676665	404093	CUEDC1	-0,355538532
GSE70769	ILMN_1709032	79443	FYCO1	-0,3552015
GSE70769	ILMN_1775330	388115	C15orf52	-0,353529355
GSE70769	ILMN_1712095	4303	FOXO4	-0,352711546
GSE70769	ILMN_1784005	644615	LOC644615	-0,35205942
GSE70769	ILMN_1696643	7094	TLN1	-0,347220812
GSE70769	ILMN_1735788	11078	TRIOBP	-0,346370023
GSE70769	ILMN_1684554	1307	COL16A1	-0,344768303
GSE70769	ILMN_1726589	57124	CD248	-0,344667553
GSE70769	ILMN_2373982	9463	PICK1	-0,343700471
GSE70769	ILMN_1711994	10312	TCIRG1	-0,341336013
GSE70769	ILMN_1786024	171568	POLR3H	-0,337796947
GSE70769	ILMN_1764228	1601	DAB2	-0,337594303
GSE70769	ILMN_1726456	6520	SLC3A2	-0,337425157
GSE70769	ILMN_1733248	340371	NRBP2	-0,331758903
GSE70769	ILMN_1739450	4779	NFE2L1	-0,329626248
GSE70769	ILMN_1655990	55755	CDK5RAP2	-0,328313968
GSE70769	ILMN_1761463	79180	EFHD2	-0,327620687
GSE70769	ILMN_2350574	91663	MYADM	-0,327604936
GSE70769	ILMN_2097793	57659	ZBTB4	-0,325940951
GSE70769	ILMN_1690653	10263	CDK2AP2	-0,325380161
GSE70769	ILMN_1803559	642477	FLJ39632	-0,325026482
GSE70769	ILMN_1674038	1509	CTSD	-0,322678358

raw p value	pFDR
6,55E-04	0,025034302
2,79E-04	0,017124331
1,09E-04	0,010012856
0,00110878	0,034655925
6,41E-04	0,024927587
1,21E-05	0,002978487
2,12E-04	0,015539329
1,14E-04	0,010261049
0,001645283	0,044728393
4,57E-05	0,006375751
0,001696059	0,045306908
6,75E-05	0,007976403
5,96E-04	0,024242052
0,002039133	0,049716011
0,001955136	0,049030025
3,73E-04	0,019484281
7,40E-04	0,026489678
0,001676611	0,045180259
3,93E-04	0,019930822
8,80E-05	0,008861597
2,52E-05	0,004191273
0,001041682	0,033684696
7,84E-04	0,027529344
0,002066492	0,049835973
0,001834122	0,046758707
9,43E-05	0,009056788
4,11E-04	0,020260029
7,10E-04	0,026145281
7,11E-04	0,026145281
1,50E-05	0,001231244
2,15E-04	0,006520489
1,88E-05	0,00142228
3,14E-05	0,001939867
8,37E-04	0,015493983
1,43E-04	0,005056423
7,32E-04	0,014224695
0,004318149	0,044057139
0,001575175	0,023179586
0,002108681	0,027855599
0,00518137	0,049326557
0,003203311	0,036586739
0,002653811	0,032466731
3,85E-07	1,57E-04
0,002010906	0,026907114
0,002134374	0,028111635
5,71E-04	0,012176757
0,001238766	0,020036162
0,001163808	0,019303069
1,91E-04	0,006022449
0,004520108	0,045144306
0,001506015	0,022568821

2,70E-04	0,007600483
0,001596097	0,023356286
2,96E-07	1,27E-04
0,001451619	0,022038492
0,003194183	0,036538699
0,005228055	0,04957882
6,57E-04	0,013299131
0,002084942	0,027656832
0,005018088	0,04825685
5,40E-06	6,87E-04
0,004177223	0,043323191
0,001802361	0,025151746
4,01E-07	1,60E-04
9,56E-05	0,003872167
5,08E-05	0,002582144
8,17E-04	0,015269911
0,001404079	0,021574092
0,001778967	0,024981814
3,26E-06	5,07E-04
1,19E-05	0,001066623
9,05E-04	0,01638305
0,001610974	0,023481426
1,07E-05	0,001003929
2,75E-04	0,007651372
1,80E-04	0,005807147
1,34E-04	0,004865203
4,29E-04	0,010145058
0,003595563	0,039274342
1,41E-06	3,25E-04
1,04E-04	0,004101057
4,98E-07	1,80E-04
0,004316455	0,044057139
9,92E-05	0,003980781
3,65E-06	5,43E-04
3,75E-06	5,50E-04
3,20E-04	0,008374494
2,70E-07	1,25E-04
6,28E-05	0,00297339
1,11E-07	9,12E-05
2,04E-06	3,79E-04
0,001998063	0,02679967
0,004282553	0,043964518
0,00463557	0,045763928
4,06E-05	0,002284185
3,18E-04	0,00835234
0,002725661	0,033019266
0,002448002	0,03081204
0,003550083	0,038949484
1,92E-07	1,05E-04
0,001315607	0,020768119
0,002379201	0,030210695
2,77E-06	4,55E-04
0,002466579	0,030951043

5,87E-04	0,012347473
8,83E-07	2,43E-04
8,09E-05	0,00347139
7,35E-05	0,003273769
3,49E-04	0,008866778
2,17E-04	0,006538307
4,99E-05	0,00255176
3,26E-05	0,00197257
0,001007817	0,017563463
4,64E-04	0,010662758
3,17E-06	5,00E-04
2,72E-09	1,54E-05
0,002468609	0,030962498
9,43E-04	0,01685263
2,96E-05	0,001882462
1,08E-04	0,004173632
4,37E-05	0,002372014
0,004646906	0,045845351
0,003914114	0,04148648
9,62E-04	0,017050591
2,00E-05	0,001493338
6,50E-05	0,003042768
4,24E-06	5,89E-04
2,55E-06	4,32E-04
0,001123068	0,018927714
0,002556211	0,031637634
0,001236611	0,020027944
5,85E-06	7,20E-04
2,03E-05	0,001504204
2,63E-04	0,007469042
0,001453315	0,02205041
4,75E-05	0,002469869
8,57E-06	8,69E-04
1,67E-06	3,54E-04
2,84E-09	1,54E-05
3,11E-05	0,001929977
1,59E-05	0,001271059
0,00468061	0,046045291
1,42E-08	3,87E-05
1,34E-05	0,001153897
0,001645421	0,023775293
8,82E-07	2,43E-04
1,91E-04	0,006024593
8,34E-04	0,015461534
7,43E-08	7,36E-05
4,70E-04	0,010733718
1,83E-04	0,005859569
3,19E-04	0,008356089
0,005184623	0,049346975
1,13E-05	0,0010264
0,001721069	0,024454003
0,002062416	0,027431544
2,07E-05	0,001531483

9,55E-04	0,016963597
0,004641142	0,045798632
2,40E-04	0,007015557
3,76E-04	0,009305519
4,42E-04	0,010303274
1,23E-04	0,004584159
0,001663157	0,023894678
0,004715106	0,046262045
9,30E-07	2,51E-04
4,36E-05	0,002369675
1,59E-05	0,001271059
8,26E-06	8,55E-04
6,94E-06	7,89E-04
4,19E-05	0,002309922
2,00E-05	0,001493338
2,89E-05	0,001866567
0,001317661	0,020768119
2,69E-04	0,007582082
2,71E-06	4,52E-04
8,70E-05	0,003633354
6,81E-07	2,26E-04
4,22E-06	5,88E-04
0,002951104	0,03477618
4,54E-04	0,010499577
1,30E-06	3,11E-04
2,78E-07	1,25E-04
0,005134062	0,049054412
2,84E-05	0,001853066
4,01E-05	0,002271484
1,36E-05	0,001163626
1,69E-04	0,005589448
1,16E-05	0,001047143
0,002809233	0,033629258
0,001834165	0,025373107
4,98E-05	0,00255176
1,05E-04	0,004112788
0,001424909	0,021796405
8,74E-05	0,0036389
7,69E-04	0,014693293
9,39E-04	0,016823018
0,002828842	0,033791349
3,96E-04	0,009682712
4,36E-04	0,010246217
2,23E-05	0,001589936
1,72E-04	0,005640263
5,43E-05	0,002685209
5,26E-04	0,011631738
8,89E-05	0,003663147
5,72E-05	0,002796463
0,003653527	0,039638719
1,55E-04	0,00531987
4,57E-05	0,002421285
0,001659384	0,023851436

6,50E-07	2,19E-04
0,001719694	0,02445007
3,22E-04	0,008428463
5,55E-07	1,96E-04
5,63E-05	0,002766075
3,60E-05	0,002087579
0,003776759	0,040411233
1,12E-05	0,0010264
1,70E-04	0,005596861
4,15E-04	0,009972409
0,001511825	0,02262101
5,59E-05	0,002757279
0,001622018	0,02359607
1,48E-05	0,0012271
0,003614715	0,039415874
0,003172873	0,036331092
7,55E-05	0,003322643
1,82E-04	0,005859569
5,06E-05	0,002578156
3,65E-04	0,009118993
4,32E-04	0,010199607
4,68E-04	0,010712584
9,46E-06	9,29E-04
7,99E-06	8,41E-04
6,21E-04	0,012803766
1,14E-04	0,004330838
0,001586342	0,02326732
0,003191709	0,03651978
6,69E-05	0,003086377
0,001299804	0,020651518
4,05E-04	0,009828813
0,001080951	0,018469462
4,39E-04	0,010291188
2,33E-05	0,001631936
0,001415935	0,021715596
3,31E-05	0,001989127
0,002735779	0,033060917
8,65E-07	2,43E-04
5,07E-04	0,011347845
2,12E-04	0,006473832
2,00E-04	0,00621794
1,84E-04	0,005882942
5,36E-04	0,011772227
9,87E-05	0,003963071
0,000325685	0,008477337
2,74E-04	0,007631192
2,85E-04	0,007759204
8,76E-04	0,016017809
0,001505971	0,022568821
1,12E-08	3,58E-05
3,47E-08	5,53E-05
0,001350991	0,021135472
3,55E-04	0,008975952

5,25E-04	0,01161901
8,91E-05	0,003667905
5,21E-04	0,011567186
3,12E-04	0,008285523
1,23E-04	0,004577354
1,61E-05	0,001277097
1,07E-09	9,56E-06
5,02E-06	6,56E-04
0,001125098	0,0189374
0,003068169	0,035591387
0,005042044	0,048445384
7,09E-07	2,29E-04
0,002605758	0,032063786
1,45E-05	0,001208197
4,70E-06	6,32E-04
8,86E-05	0,003655625
7,58E-05	0,003331099
2,11E-05	0,001543562
1,09E-05	0,001016299
2,25E-05	0,001599003
3,02E-05	0,001903029
3,22E-05	0,001964194
0,00483648	0,047028192
8,28E-04	0,015382717
0,005086976	0,048750913
0,004178842	0,043329225
0,002986076	0,034967748
7,07E-05	0,003197124
1,41E-04	0,004993368
1,70E-07	1,01E-04
1,60E-05	0,001271059
3,81E-05	0,002179315
1,75E-06	3,58E-04
3,04E-06	4,86E-04
7,07E-04	0,013892214
2,48E-04	0,007181343
2,71E-04	0,007609606
0,003631562	0,039507038
0,002879518	0,034222313
5,76E-04	0,012214826
4,89E-07	1,80E-04
1,47E-04	0,005133223
2,50E-04	0,007214493
6,32E-06	7,54E-04
4,15E-05	0,002295678
0,002225451	0,02891788
0,00271891	0,032968933
2,83E-04	0,007759204
2,85E-04	0,007759204
4,85E-05	0,002511431
2,30E-04	0,006830579
4,12E-05	0,002295678
3,56E-05	0,002082716

2,15E-05	0,001561322
0,002219489	0,028865673
6,48E-06	7,62E-04
2,14E-05	0,00155716
0,001349939	0,021128483
8,07E-04	0,015146987
7,18E-07	2,30E-04
3,23E-06	5,06E-04
2,31E-05	0,001621293
0,002550969	0,031616682
0,005021568	0,048279886
2,92E-05	0,001866567
5,87E-04	0,012347473
2,99E-04	0,00802704
0,00139948	0,021539802
2,62E-05	0,001751934
1,74E-04	0,005710542
4,87E-08	5,81E-05
0,001008768	0,017572821
3,96E-05	0,002250044
5,04E-04	0,011292187
8,65E-05	0,003620006
0,001824258	0,025306679
1,90E-04	0,006020961
4,22E-05	0,002318163
1,60E-04	0,005409546
2,77E-06	4,55E-04
9,52E-04	0,016928334
7,01E-07	2,29E-04
2,91E-04	0,00787259
0,001861876	0,025605253
8,21E-05	0,003498171
2,03E-07	1,09E-04
2,32E-04	0,006865487
2,43E-07	1,19E-04
0,003334208	0,037586179
8,33E-06	8,57E-04
1,15E-05	0,001040261
1,29E-05	0,001122176
8,19E-05	0,003496598
2,16E-04	0,006532318
0,003437674	0,038291557
5,96E-06	7,30E-04
1,52E-04	0,005268767
1,75E-04	0,005725516
1,97E-05	0,001477876
1,25E-05	0,001100853
1,50E-07	1,01E-04
0,004090941	0,0428072
2,31E-04	0,006834019
1,26E-04	0,004674132
8,18E-04	0,015284899
4,03E-05	0,002279811

0,00514102	0,049089301
4,06E-07	1,60E-04
1,59E-07	1,01E-04
1,52E-06	3,40E-04
7,34E-05	0,003273769
3,88E-04	0,009536714
0,004651961	0,045874903
1,12E-05	0,0010264
0,001458081	0,022106452
5,73E-04	0,0121996
0,003095172	0,035752302
1,00E-05	9,62E-04
0,003636056	0,039511585
1,78E-04	0,005792816
4,94E-07	1,80E-04
0,001757152	0,02476917
4,45E-04	0,010352855
0,003243446	0,036912635
0,004128607	0,043089194
2,07E-04	0,006360711
8,86E-05	0,003655625
0,005248648	0,049690921
8,28E-04	0,01538217
0,002267891	0,029323929
2,24E-04	0,006689908
3,55E-04	0,008975952
2,33E-04	0,006866107
4,73E-07	1,80E-04
1,09E-04	0,004200837
7,79E-06	8,29E-04
0,003034831	0,03538835
5,77E-04	0,012214826
4,71E-04	0,010746062
1,20E-04	0,004520192
4,67E-05	0,002454888
8,75E-05	0,003638921
8,89E-04	0,016169339
8,63E-04	0,015840193
8,79E-05	0,003644185
2,83E-04	0,007759204
0,003069293	0,035594531
0,003473523	0,038488703
1,57E-04	0,005339261
0,00124166	0,020068391
5,20E-04	0,011542969
0,003624405	0,039470296
0,002150827	0,028300541
0,004071744	0,042707216
0,00133277	0,020918579
1,50E-04	0,005220536
8,72E-06	8,78E-04
0,002276914	0,029414981
1,12E-05	0,0010264

1,35E-04	0,004874403
1,17E-04	0,00442011
6,87E-06	7,85E-04
0,002206435	0,02875621
0,00212682	0,028045319
7,81E-04	0,014818336
8,28E-04	0,01538217
0,001942273	0,026336862
0,003915436	0,04148648
1,69E-07	1,01E-04
6,23E-04	0,01282632
0,001641236	0,023736113
9,66E-04	0,017066695
0,002549003	0,031601104
1,27E-04	0,004678451
1,09E-04	0,004200837
9,43E-05	0,003823755
8,78E-05	0,00364409
1,04E-05	9,89E-04
5,08E-04	0,011373556
2,72E-05	0,001791338
0,003249898	0,036976619
0,001043543	0,01798205
2,87E-05	0,001864815
0,001229738	0,019948081
5,60E-04	0,012055378
9,65E-04	0,017065388
0,003592097	0,039255732
2,77E-04	0,007698371
0,001351334	0,021135472
0,003700664	0,03998117
0,00152996	0,022754778
1,20E-04	0,004520192
0,001719127	0,02445007
5,65E-05	0,002769697
0,001958992	0,026490989
0,00463938	0,04579139
7,08E-05	0,003200438
2,12E-05	0,00154523
0,001336157	0,020956959
0,001586361	0,02326732
1,83E-05	0,001392836
0,001910481	0,02604851
6,04E-04	0,012577491
0,001758495	0,024780263
0,001108931	0,018788974
1,33E-06	3,11E-04
9,93E-04	0,01739428
0,002741081	0,033078498
4,99E-05	0,00255176
1,26E-07	9,51E-05
1,23E-05	0,001083856
3,19E-04	0,008357302

1,85E-04	0,005907665
0,001308753	0,020716799
0,003699258	0,039975681
0,001089623	0,018560672
6,69E-05	0,003086377
7,75E-04	0,014764852
9,18E-04	0,016581833
0,004920059	0,047664005
1,47E-05	0,001223516
1,12E-04	0,004291356
0,004257088	0,043795131
1,38E-04	0,00493993
0,001199274	0,019652056
8,07E-05	0,00347139
0,001378077	0,021345322
0,004717064	0,046271065
0,004816845	0,04690304
0,004054542	0,042595646
8,78E-04	0,01604366
2,04E-04	0,006298026
0,001798198	0,025109389
5,45E-04	0,011872371
0,003470114	0,038466303
0,002953274	0,034782604
1,22E-05	0,001083856
2,13E-04	0,006473832
0,001470902	0,022210119
8,89E-05	0,003663147
0,001877915	0,025754265
0,003982891	0,042001399
3,91E-08	5,53E-05
5,91E-04	0,012391163
1,20E-04	0,004520192
0,004175035	0,043320001
4,75E-05	0,002469869
0,002310572	0,029673229
1,38E-05	0,001168161
1,74E-04	0,005702482
2,84E-04	0,007759204
0,003882014	0,041220608
0,005255402	0,049744292
7,51E-05	0,003316898
0,004071874	0,042707216
8,07E-06	8,48E-04
3,14E-05	0,001938712
5,10E-04	0,011387241
5,96E-04	0,012454438
1,31E-04	0,004794554
2,95E-05	0,001878444
3,32E-06	5,11E-04
3,31E-04	0,008531228
3,48E-06	5,22E-04
0,00335662	0,037728312

3,59E-04	0,009017286
0,002330396	0,029818296
1,14E-04	0,004332165
0,00203235	0,027112609
0,003398774	0,03808679
0,001553999	0,022966602
0,001142626	0,019069743
2,46E-04	0,007151821
4,88E-06	6,43E-04
3,27E-06	5,07E-04
4,81E-06	6,42E-04
3,64E-06	5,42E-04
0,002464446	0,030944532
0,00344513	0,038329654
0,00100042	0,017461876
1,73E-05	0,001340171
2,41E-04	0,007039857
4,45E-04	0,010350471
6,25E-05	0,002966695
3,68E-04	0,009156433
8,83E-05	0,003654516
0,001913868	0,02607075
0,001199577	0,019652056
1,35E-04	0,004878962
1,50E-04	0,005236424
0,001787587	0,025021877
8,13E-05	0,003482339
3,63E-04	0,009093356
0,001607139	0,023448548
0,002877812	0,034211167
6,08E-05	0,002924537
0,00146455	0,022178925
3,33E-05	0,001997493
8,11E-04	0,015197903
8,21E-05	0,003498171
3,05E-05	0,001914476
6,73E-04	0,0134934
2,21E-04	0,00665431
2,70E-07	1,25E-04
7,93E-04	0,014958655
8,24E-06	8,55E-04
0,003064661	0,035591387
6,65E-04	0,013400091
2,28E-04	0,006772639
1,46E-04	0,005110857
7,30E-04	0,014205812
0,001379692	0,021354067
5,38E-04	0,011786355
5,77E-05	0,002815762
9,85E-07	2,61E-04
4,66E-04	0,010680699
2,02E-06	3,78E-04
4,39E-05	0,00237714

0,001313363	0,020745553
0,002730893	0,033037724
3,78E-05	0,002168435
0,002344361	0,029921995
1,29E-05	0,001124128
1,43E-04	0,005059531
0,001507534	0,022579552
1,01E-05	9,66E-04
7,86E-05	0,003413946
1,68E-04	0,005575242
0,001540861	0,02284063
6,64E-04	0,013380138
0,00340377	0,03813318
1,62E-06	3,50E-04
3,34E-05	0,001997493
0,001264578	0,020273251
1,31E-06	3,11E-04
8,71E-05	0,003634002
8,40E-04	0,015538615
0,004246814	0,043728635
0,002141955	0,02820315
0,004837559	0,047028419
0,001639795	0,023727325
0,002095795	0,027734734
1,06E-05	0,001003929
1,38E-04	0,00493993
6,38E-04	0,013033696
0,004603224	0,045625713
4,52E-05	0,002410418
0,001105336	0,018771646
0,002175648	0,028478286
9,87E-06	9,56E-04
0,003107972	0,035837818
9,23E-04	0,016653552
0,001532256	0,022773704
6,88E-04	0,013684083
0,001182502	0,019510932
2,68E-04	0,007573075
1,40E-07	1,01E-04
0,002363794	0,03009227
2,90E-05	0,001866567
1,44E-04	0,005076355
0,004897378	0,047464925
0,000219516	0,006606841
5,73E-04	0,012199818
9,07E-05	0,003710916
0,003868127	0,041102541
3,95E-04	0,009681935
1,53E-04	0,005277706
8,50E-04	0,015665073
7,90E-07	2,31E-04
6,96E-05	0,003165786
1,61E-07	1,01E-04

0,002527257	0,031418958
4,93E-04	0,011134576
5,86E-04	0,012342533
0,001601337	0,023409897
9,78E-04	0,017228877
1,67E-07	1,01E-04
2,29E-05	0,001612054
2,12E-04	0,006473832
0,002354107	0,030003244
2,54E-05	0,001709835
2,52E-04	0,007270392
3,48E-05	0,002050321
0,002280283	0,029441425
0,001360881	0,021217735
0,00101611	0,017645855
0,004040781	0,042481132
0,001400216	0,021539802
9,87E-04	0,017336748
2,23E-04	0,006675064
3,24E-04	0,008459166
0,003768424	0,040370532
1,52E-04	0,005270661
8,96E-04	0,016253823
0,004793047	0,046748741
1,63E-05	0,001283741
0,002921256	0,034552428
0,001600964	0,023409897
9,46E-04	0,016856745
1,06E-04	0,004117345
3,13E-06	4,96E-04
6,66E-05	0,003085695
9,97E-04	0,017433268
0,001192453	0,019596709
9,75E-04	0,017188545
9,21E-06	9,13E-04
0,002173774	0,028470627
4,90E-05	0,002527141
2,82E-04	0,007759204
1,83E-05	0,001392836
1,56E-04	0,005322108
0,002753043	0,03315805
7,77E-04	0,014772651
5,81E-04	0,012276461
8,93E-05	0,003672458
5,70E-04	0,012167981
0,002589442	0,031915916
2,40E-06	4,17E-04
5,35E-04	0,011763648
1,15E-04	0,004360192
0,00501177	0,048222952
7,50E-06	8,21E-04
1,59E-04	0,005386936
1,71E-04	0,005634328

3,86E-06	5,58E-04
6,74E-04	0,01350011
0,001159966	0,019258118
0,001654224	0,02381569
4,80E-04	0,010896177
5,78E-05	0,00281815
7,12E-05	0,003202345
7,53E-04	0,014499707
2,65E-05	0,001761829
6,24E-05	0,002966695
7,74E-06	8,29E-04
0,00169841	0,024232532
5,92E-04	0,012403501
5,19E-05	0,002607757
0,004390976	0,044432451
0,005118692	0,048950456
0,005287176	0,049960108
2,00E-05	0,001493338
0,00294651	0,034740424
0,002664202	0,032513486
2,87E-05	0,001864815
6,01E-06	7,30E-04
4,53E-04	0,010478695
0,001132931	0,018991869
0,001649418	0,02377568
3,57E-04	0,00899617
3,30E-05	0,001985972
3,19E-05	0,001953832
0,002019415	0,026972362
7,38E-07	2,31E-04
3,00E-04	0,008030125
1,27E-04	0,004678451
0,004934739	0,047755387
3,32E-04	0,008541105
5,14E-05	0,002590842
0,004369836	0,044336631
0,003762492	0,040343791
5,77E-04	0,012214826
0,001760268	0,024797396
2,44E-04	0,007110825
0,001226934	0,019931635
0,002985321	0,034967748
1,88E-06	3,66E-04
5,34E-04	0,011748472
0,004339011	0,044137222
0,001544176	0,022873018
0,004191525	0,043410208
1,63E-04	0,005463718
7,25E-04	0,014150598
1,47E-06	3,35E-04
0,004726658	0,046324367
4,41E-04	0,010291188
2,61E-04	0,007447042

4,54E-05	0,002413998
2,58E-04	0,007407658
0,002660729	0,032495221
1,81E-04	0,005841792
1,61E-04	0,005409546
1,07E-07	9,12E-05
2,71E-04	0,007609606
0,001558458	0,02301722
0,003067986	0,035591387
1,05E-04	0,004112788
0,002885138	0,034261687
1,55E-06	3,40E-04
0,00102809	0,017777655
0,001522544	0,022727947
2,13E-04	0,006473832
0,002917691	0,034537771
0,002785778	0,033447355
6,75E-04	0,013511038
0,00437496	0,044340892
1,50E-04	0,005235277
1,55E-04	0,00531987
4,93E-04	0,011129617
3,28E-04	0,008498288
8,18E-05	0,003495025
0,002457746	0,030908484
0,001088147	0,018549713
0,003894885	0,041337059
9,39E-06	9,25E-04
6,43E-06	7,62E-04
0,005050226	0,048492621
4,88E-04	0,011028096
0,002857698	0,034053857
0,003229582	0,036783045
2,92E-05	0,001866567
0,003585867	0,039206885
0,001009749	0,017576503
4,30E-05	0,002351749
0,002987771	0,034967748
1,82E-06	3,63E-04
1,66E-04	0,005525897
0,001891061	0,02589469
7,98E-05	0,003448841
0,002675795	0,032628141
0,002646603	0,032396337
5,27E-04	0,011631738
5,31E-06	6,78E-04
3,29E-04	0,008506843
2,85E-04	0,007759204
2,22E-04	0,006655488
0,004744116	0,046444376
0,005111829	0,048915426
5,13E-06	6,62E-04
5,82E-05	0,002828204

2,43E-04	0,007094663
6,28E-05	0,00297339
4,92E-05	0,002534058
0,00137707	0,021345322
4,07E-04	0,009842211
0,003823042	0,040769354
0,00216032	0,028352767
0,002567093	0,031737053
0,001221068	0,019858075
0,002466689	0,030951043
7,77E-06	8,29E-04
0,001564447	0,023067435
9,45E-04	0,01685263
0,001945044	0,0263584
2,57E-04	0,007369432
2,05E-07	1,09E-04
1,95E-06	3,70E-04
3,29E-06	5,08E-04
8,18E-06	8,55E-04
6,23E-05	0,002965203
0,001357613	0,021196471
2,59E-04	0,007407658
0,001477574	0,022274557
2,42E-04	0,00707096
0,005048261	0,048490649
2,06E-04	0,006351359
0,001961028	0,026502441
0,002464781	0,030944532
7,43E-04	0,014366294
0,002396995	0,030384677
2,62E-05	0,001751934
1,37E-04	0,00491802
2,34E-05	0,001631936
2,94E-06	4,74E-04
0,00200112	0,026832603
0,001320928	0,020777184
0,001081985	0,018472949
3,28E-04	0,008502437
0,002294538	0,02955694
1,80E-04	0,005807147
9,60E-05	0,0038798
4,66E-04	0,010679977
8,29E-05	0,003515744
9,84E-08	8,64E-05
4,15E-06	5,83E-04
1,76E-04	0,005735638
0,001741836	0,024662543
0,001765206	0,024827703
1,81E-06	3,63E-04
7,00E-05	0,003178087
1,01E-04	0,004043151
1,52E-04	0,005270661
6,53E-05	0,003046679

0,00143833	0,021946324
0,003095452	0,035752302
2,44E-05	0,001676124
1,57E-04	0,005339261
1,08E-05	0,001009308
0,00163159	0,023665653
0,004409441	0,044526934
8,28E-05	0,003514642
0,003634151	0,039511585
4,40E-07	1,70E-04
1,31E-04	0,004788126
5,56E-04	0,012019926
1,21E-04	0,004530468
0,003562823	0,039036452
4,68E-04	0,010705797
1,06E-04	0,004125438
8,11E-06	8,50E-04
1,13E-04	0,004314575
0,003239342	0,036875347
0,004301639	0,044022306
2,82E-05	0,001839391
0,001480528	0,02230255
2,04E-04	0,006298026
3,65E-04	0,009118993
0,004477116	0,044886035
2,24E-04	0,006689908
0,004749515	0,046487013
2,34E-06	0,000411684
5,74E-04	0,012206185
6,24E-04	0,012841012
4,34E-04	0,010215739
0,001671077	0,023957078
1,17E-07	9,12E-05
2,97E-05	0,001882462
0,004049346	0,042551087
0,002624357	0,032230315
0,002642187	0,032386787
0,001363392	0,021224826
0,00404049	0,042481132
2,16E-06	3,97E-04
3,95E-04	0,009682467
2,62E-04	0,007462687
8,38E-07	2,43E-04
0,002121084	0,027986248
2,99E-04	0,008020929
9,02E-04	0,016341619
7,44E-06	8,19E-04
2,83E-04	0,007759204
1,88E-04	0,005994917
1,38E-05	0,001168161
0,004839722	0,047035194
4,84E-06	6,43E-04
3,83E-04	0,009456708

0,00426101	0,043795131
5,95E-04	0,012448848
6,88E-04	0,013684083
1,99E-04	0,006191578
0,003946509	0,041749129
0,002929164	0,034618376
1,30E-04	0,004756747
0,003062325	0,035588358
1,41E-05	0,001183429
1,48E-06	3,36E-04
7,65E-05	0,00335817
0,00349619	0,038586294
1,98E-06	3,73E-04
2,18E-06	3,97E-04
0,003978305	0,041964524
0,00104259	0,017972571
0,002476456	0,031012437
5,74E-04	0,012206185
1,04E-04	0,004103487
5,29E-05	0,002642859
6,11E-04	0,012648831
0,004663705	0,045932222
2,76E-06	4,55E-04
3,00E-04	0,008039945
6,28E-04	0,012908933
0,003435969	0,038291557
0,001629015	0,023647584
9,29E-04	0,016717931
3,15E-04	0,008303463
8,33E-04	0,015452174
5,32E-04	0,01171802
0,003060341	0,035587889
4,15E-05	0,002295678
2,51E-04	0,007246812
1,03E-04	0,004082771
2,33E-04	0,00687271
6,57E-04	0,013299131
1,84E-04	0,005894564
4,22E-05	0,002318163
0,001882632	0,025808638
1,69E-04	0,005594526
9,02E-04	0,016341619
1,84E-05	0,001392836
0,004165131	0,043267631
0,002311113	0,029673229
0,001068279	0,018302682
0,001234821	0,020023242
9,66E-04	0,017066695
5,03E-04	0,011290289
1,24E-04	0,004605171
7,74E-04	0,014758005
0,003358731	0,037742512
0,004288975	0,04398676

0,002090937	0,027698834
0,005122649	0,048966369
3,03E-04	0,008083667
0,003571129	0,039093722
0,004840368	0,047035194
4,24E-05	0,002328808
8,82E-04	0,016080802
2,18E-05	0,001565683
3,84E-04	0,009471105
3,16E-04	0,008314475
9,38E-04	0,016823018
0,00316454	0,036264793
4,02E-06	5,74E-04
6,71E-05	0,003091112
0,002446713	0,03080452
8,94E-04	0,016230795
2,98E-04	0,008012556
6,47E-04	0,013154321
0,00319876	0,036572893
0,001640096	0,023727325
3,55E-04	0,008975952
0,001338777	0,020975899
3,24E-04	0,008459166
0,001112576	0,01881068
6,50E-05	0,003042768
0,00113327	0,018991869
0,004711488	0,046262045
0,003939071	0,041687333
3,49E-05	0,002054443
1,61E-04	0,005409546
7,59E-06	8,23E-04
8,61E-05	0,003611058
0,00441031	0,044526934
4,46E-05	0,002396026
1,53E-05	0,00124424
2,96E-07	1,27E-04
0,001216411	0,019833021
0,001425739	0,021801625
0,001720289	0,02445072
0,003631944	0,039507038
6,59E-04	0,013308319
6,57E-06	7,62E-04
0,004308241	0,044050124
0,001172227	0,019382212
0,004648862	0,045854495
0,003862172	0,041088269
3,75E-04	0,009304035
0,004818344	0,04690304
8,16E-04	0,015268608
1,12E-04	0,004291356
2,66E-05	0,001764572
0,003030796	0,035350561
4,72E-05	0,002463345

6,52E-05	0,003045123
0,001158072	0,019248187
1,66E-04	0,005536442
7,57E-04	0,014526523
0,003879605	0,041204846
1,74E-04	0,005705655
8,12E-04	0,015210917
5,62E-04	0,012061308
0,001722375	0,024459517
0,001751687	0,024711688
2,22E-05	0,001585299
0,002615919	0,032153285
0,003355991	0,037728312
1,98E-04	0,006169312
0,001102737	0,018755364
5,61E-04	0,012058374
0,001328002	0,020851081
0,002781533	0,033405393
3,94E-04	0,009666818
0,001676117	0,023991307
2,27E-04	0,00674015
2,96E-05	0,001882462
0,005219768	0,049543858
7,17E-06	8,02E-04
1,41E-04	0,004993368
3,30E-04	0,008531228
3,18E-05	0,001953832
4,59E-04	0,01057452
0,001934416	0,0262543
0,001309732	0,020722957
8,50E-04	0,015665073
0,00129903	0,020651518
0,001151987	0,019176536
1,56E-04	0,005322108
1,45E-04	0,005098506
7,92E-04	0,014953713
7,32E-04	0,014224695
7,45E-04	0,014396277
1,27E-04	0,004691206
7,46E-04	0,014403052
0,004588642	0,045563188
0,004371554	0,044336631
0,003646055	0,039583206
2,84E-06	4,64E-04
6,44E-04	0,013101756
0,002233272	0,028985678
0,001436515	0,02193219
2,89E-04	0,007847427
0,004473109	0,044866059
6,14E-05	0,002941117
1,15E-04	0,004377714
0,002937731	0,034673627
6,77E-05	0,003106515

3,84E-05	0,002192026
0,003818724	0,04073306
8,77E-07	2,43E-04
9,41E-04	0,016836698
0,004581073	0,045521965
0,001807166	0,02518723
2,09E-04	0,006393518
0,001435426	0,021927172
1,52E-04	0,005270661
0,002308967	0,029673229
0,002129626	0,028069027
0,002512297	0,031313984
0,004258368	0,043795131
0,001449376	0,022032446
2,33E-06	0,000411684
4,65E-04	0,010662758
0,004223399	0,043586852
0,004142933	0,04316791
3,30E-07	1,39E-04
8,82E-05	0,003653999
4,83E-04	0,010938256
2,58E-05	0,00173298
0,002741238	0,033078498
0,002468992	0,030962498
0,002117673	0,027949521
0,002163618	0,028365037
1,63E-04	0,005463718
1,73E-05	0,001338065
0,003439322	0,038291557
3,54E-04	0,008954783
2,42E-05	0,00167025
0,002613343	0,032140027
0,001448215	0,022032446
0,00142118	0,021774239
0,003312189	0,037464683
6,05E-04	0,012581609
2,52E-04	0,007276255
0,004373742	0,044340892
9,98E-04	0,017438748
8,55E-05	0,003591706
0,002526703	0,031418958
0,003690818	0,039923211
0,003233403	0,036817156
0,004174631	0,043320001
0,003563268	0,039036452
0,002321323	0,029746753
5,64E-04	0,012069922
0,00137428	0,021314731
4,29E-04	0,010144675
1,56E-04	0,005322108
1,80E-05	0,001380111
8,34E-08	7,90E-05
6,92E-05	0,003155149

0,004425377	0,044644282
0,001974616	0,026613398
3,35E-05	0,001998978
0,003428285	0,038263473
1,05E-04	0,004110075
5,32E-05	0,002651813
0,003867073	0,041102541
0,004130166	0,043095368
1,29E-07	9,58E-05
0,0010749	0,018401362
0,003698942	0,039975681
1,53E-05	0,00124424
2,92E-04	0,007884729
8,22E-06	8,55E-04
0,001433712	0,021908494
6,51E-04	0,013212306
8,51E-05	0,003580622
1,76E-04	0,005735638
0,002015935	0,026950126
0,001647409	0,023775293
4,33E-04	0,010205018
0,002690359	0,032755865
0,002303625	0,029639706
5,07E-06	6,59E-04
0,004160657	0,043260875
7,38E-05	0,003280167
5,74E-04	0,012206185
4,18E-04	0,009992372
1,28E-05	0,001116061
0,001024751	0,017740587
0,002603448	0,032044208
0,003484273	0,038527525
7,45E-04	0,014401012
4,00E-04	0,009757706
0,001118534	0,018872719
6,63E-04	0,013370653
0,002702724	0,032844303
0,002729962	0,033037724
1,38E-04	0,004951133
7,90E-04	0,01493436
9,04E-05	0,003700939
0,001129241	0,018952869
0,005052523	0,048504221
1,56E-05	0,001265534
0,001834173	0,025373107
0,0037539	0,040292468
0,002656358	0,032472829
0,001762594	0,024809113
0,0012279	0,019940053
0,002341952	0,029921995
1,52E-04	0,005270661
0,001168599	0,019338557
0,00516502	0,049234032

0,001871942	0,025704006
0,004313506	0,044050124
0,001623839	0,02359944
2,98E-04	0,008006245
5,98E-05	0,002884243
2,00E-04	0,00621794
7,07E-05	0,003197124
0,001805835	0,025184451
0,00181512	0,025258577
1,58E-04	0,005379686
0,004430953	0,044644282
0,003780676	0,040424009
0,001468178	0,022199084
4,44E-06	6,04E-04
6,09E-04	0,012641815
2,81E-07	1,25E-04
0,00350447	0,038620723
0,00215379	0,028317129
7,30E-04	0,014205812
5,82E-04	0,012279129
0,001400295	0,021539802
1,92E-04	0,006043514
1,07E-04	0,004163076
9,95E-06	9,59E-04
0,001666402	0,023921437
0,004843306	0,047043223
3,20E-04	0,008384205
0,001138829	0,019042637
1,22E-04	0,004562111
0,002920703	0,034552428
2,67E-05	0,001764845
2,19E-04	0,006591427
0,003449521	0,038337186
5,83E-06	7,20E-04
6,11E-05	0,002932428
0,002876548	0,034207985
0,001006541	0,01754809
1,44E-04	0,005077801
0,002378078	0,030205047
1,55E-04	0,005322108
2,98E-05	0,001883356
0,002006623	0,026874612
0,001687224	0,024111551
0,004079883	0,042750956
0,001168718	0,019338557
4,31E-05	0,002354334
0,001817745	0,025263805
0,00501025	0,048222952
0,002918872	0,034542571
4,10E-06	5,82E-04
8,54E-04	0,015726012
0,003737276	0,040191506
0,004153012	0,043222377

6,33E-06	7,54E-04
0,001164321	0,019303069
3,36E-04	0,008626328
3,77E-05	0,002167711
7,20E-04	0,014075675
0,004276756	0,043925254
1,31E-04	0,004774047
0,004227371	0,043598989
0,001251342	0,02015177
1,15E-05	0,001037438
2,73E-05	0,001793417
5,38E-04	0,011786355
0,000176947	0,005757422
0,002243521	0,029101749
0,001593616	0,023333191
0,002082177	0,027636613
1,84E-08	4,10E-05
1,75E-04	0,005718737
3,27E-04	0,008488641
7,09E-06	7,98E-04
2,90E-04	0,007855215
0,001593827	0,023333191
0,003333113	0,037586179
0,002462247	0,030930157
4,26E-04	0,010101098
1,39E-04	0,004974003
4,96E-08	5,81E-05
0,00500797	0,048222011
1,98E-04	0,006182214
0,00461524	0,045669692
0,004218778	0,043551851
0,001794231	0,025061851
2,64E-05	0,001757859
3,38E-06	5,13E-04
0,001228788	0,019940761
3,57E-05	0,002082716
1,59E-04	0,005386936
3,25E-04	0,008477337
1,50E-06	3,40E-04
1,55E-06	3,40E-04
0,003468434	0,038466303
0,00257209	0,03176919
0,001894968	0,02590398
1,17E-04	0,004418184
3,75E-04	0,009304035
1,68E-04	0,005564865
7,13E-04	0,013975994
2,69E-08	5,25E-05
7,35E-05	0,003273769
4,16E-04	0,009985253
8,09E-04	0,015169896
0,00169456	0,02419311
1,39E-07	1,01E-04

2,23E-04	0,006680424
0,00197795	0,026629479
0,002555552	0,031637634
0,005229196	0,04957882
4,82E-04	0,010938256
1,92E-04	0,006043514
3,61E-04	0,009040081
9,67E-05	0,003903316
6,14E-06	7,39E-04
7,31E-04	0,01421294
3,45E-06	5,19E-04
4,93E-05	0,002534058
7,82E-04	0,014830676
0,003560774	0,039036452
0,001218015	0,019839004
0,005011612	0,048222952
3,87E-04	0,009532953
2,27E-07	1,19E-04
0,003417358	0,03822436
4,01E-04	0,009759681
0,004763893	0,046545917
9,28E-04	0,016717931
0,002322961	0,029746753
0,002060608	0,027415694
5,61E-05	0,002759222
2,00E-06	3,76E-04
2,62E-04	0,007450983
1,17E-04	0,004416739
0,003427559	0,038263473
4,11E-06	5,82E-04
2,10E-04	0,006411917
0,002803932	0,033583853
2,03E-05	0,001507424
7,55E-04	0,01451881
6,42E-04	0,013101756
0,001924899	0,026172987
2,15E-04	0,006519425
7,38E-05	0,003280167
4,03E-04	0,009804907
5,76E-04	0,012214826
1,79E-04	0,005797805
0,002799246	0,033572859
3,59E-04	0,009018869
4,15E-04	0,00997453
4,41E-04	0,010291188
1,03E-04	0,004085683
4,37E-05	0,002371746
1,75E-04	0,005718737
7,72E-04	0,014727512
5,54E-06	6,97E-04
3,41E-05	0,002032448
0,001442196	0,021977819
2,13E-04	0,006473832

9,63E-04	0,017063875
1,98E-05	0,001481202
0,003950175	0,041767024
0,002898249	0,03438429
0,004539838	0,045260189
3,58E-04	0,009013238
0,003484828	0,038527525
0,00483242	0,047019514
3,06E-04	0,008148423
9,83E-04	0,017295325
5,73E-04	0,01220151
0,001622552	0,023596133
0,002562319	0,031686822
0,002400854	0,030424927
7,20E-04	0,014075384
0,001601219	0,023409897
0,001418805	0,021747848
5,50E-04	0,011952792
0,002729074	0,033037724
1,34E-05	0,001151746
2,26E-05	0,00159964
2,99E-05	0,001883356
0,001926807	0,02618295
0,003224338	0,036754061
5,42E-05	0,002685163
7,56E-04	0,014526523
5,90E-04	0,012379986
6,33E-04	0,012983688
6,00E-04	0,012516413
0,003622899	0,039466469
8,77E-05	0,00364231
1,20E-04	0,004519526
2,94E-04	0,007923834
0,004126821	0,043080646
3,66E-04	0,009122502
3,11E-05	0,001929977
0,001274281	0,020373852
0,004659052	0,045924498
0,005073948	0,048678429
0,004260502	0,043795131
0,001274276	0,020373852
9,99E-05	0,004003347
2,98E-04	0,008010879
0,002570084	0,031756406
1,66E-05	0,001297315
0,001615248	0,023536014
5,69E-04	0,012160583
2,77E-04	0,007697044
1,65E-06	3,53E-04
1,79E-04	0,005792816
9,84E-04	0,017302304
7,10E-05	0,003200508
0,001030134	0,017792285

2,79E-05	0,001821904
0,003588086	0,039221525
0,004301848	0,044022306
0,004955543	0,047885375
4,55E-05	0,002418132
0,004531617	0,045214564
2,34E-04	0,006878806
0,001103927	0,018761291
0,004454047	0,044763926
3,47E-04	0,008824545
1,20E-04	0,004520192
0,003955894	0,041805776
3,42E-04	0,008746876
0,003618586	0,03944843
0,001337929	0,020969983
5,16E-05	0,002595405
4,07E-05	0,002284185
0,001099897	0,018714212
0,001786904	0,025021877
0,001695187	0,024194301
9,97E-04	0,017434949
0,003482857	0,038527525
0,005243397	0,049672876
6,83E-04	0,013641033
0,002724099	0,033009317
0,001124467	0,0189374
2,43E-04	0,007086625
0,002009658	0,026898495
0,001352036	0,021139027
0,004617131	0,045669692
6,40E-04	0,013081285
0,004286398	0,043985926
0,002795334	0,033544009
3,83E-04	0,009456708
1,02E-05	9,73E-04
2,32E-06	0,000411684
3,07E-06	4,88E-04
0,004189129	0,04339548
4,66E-05	0,002454062
2,96E-07	1,27E-04
1,08E-04	0,004178917
0,002546768	0,031582189
0,00353233	0,038821639
4,16E-04	0,009989073
2,53E-04	0,007297007
0,001960931	0,026502441
3,65E-04	0,009109573
9,94E-04	0,017403249
0,001735166	0,024590606
8,79E-06	8,83E-04
0,005213184	0,049523578
3,50E-04	0,008889481
0,004818276	0,04690304

0,004060879	0,042642106
0,001784091	0,025021877
6,02E-04	0,01255853
4,37E-04	0,010251693
2,53E-05	0,001707921
0,003826579	0,040797302
6,37E-04	0,013027684
4,01E-04	0,009759681
0,001689473	0,024135954
1,46E-04	0,005110857
0,001374013	0,021314731
1,34E-04	0,004847912
9,40E-05	0,003816287
0,001535075	0,02279192
8,08E-05	0,00347139
8,99E-04	0,016302792
0,004429547	0,044644282
0,001632114	0,023665653
0,001308751	0,020716799
0,002535093	0,031490015
1,41E-04	0,004993368
0,002821137	0,033717389
1,57E-05	0,001270376
0,004576648	0,045521965
5,58E-04	0,0120336
4,52E-05	0,002410418
0,00188476	0,025824284
2,07E-04	0,006360711
9,68E-04	0,017088492
4,64E-04	0,010648788
4,18E-04	0,009992372
2,24E-04	0,006686662
7,18E-04	0,014053956
0,001202405	0,019655014
0,001317733	0,020768119
4,88E-04	0,011028096
1,84E-06	3,66E-04
2,31E-05	0,001621293
9,48E-04	0,016890477
0,003228135	0,036775973
3,97E-05	0,002253253
9,27E-07	2,51E-04
0,005079816	0,04872423
0,00461805	0,045669692
0,003417915	0,03822436
0,003341363	0,037613762
0,001964659	0,026535403
1,44E-05	0,001208197
0,001144639	0,01909618
1,09E-04	0,004200343
0,00146508	0,022178925
9,12E-04	0,016497282
3,07E-05	0,001919648

0,003978452	0,041964524
0,003073488	0,035615356
0,004708264	0,046256047
0,00327033	0,037161631
0,00467802	0,046029983
0,001026637	0,017760055
9,88E-04	0,017336748
9,18E-04	0,016573771
1,64E-04	0,005488906
1,12E-05	0,0010264
0,002711949	0,032924787
3,69E-05	0,002124084
0,001709126	0,02435422
0,003470637	0,038466303
0,001202265	0,019655014
5,03E-06	6,56E-04
2,74E-04	0,007634833
4,22E-04	0,010048386
2,90E-05	0,001866567
0,001186487	0,019538685
7,83E-06	8,29E-04
7,01E-04	0,013816656
4,09E-04	0,00988419
0,001190313	0,019579511
6,71E-04	0,013489501
0,001829325	0,025345402
0,003154369	0,036185484
1,79E-04	0,005799286
0,001238609	0,020036162
0,0037174	0,040089514
2,72E-04	0,007617476
6,30E-04	0,012932089
7,79E-04	0,01478302
1,36E-04	0,004905178
5,02E-05	0,002559682
8,23E-04	0,015353242
6,53E-06	7,62E-04
0,003255606	0,03702265
0,005187791	0,049366577
0,002006667	0,026874612
0,001523529	0,022734408
1,27E-05	0,001115057
0,001151597	0,019176536
1,58E-05	0,001271059
8,20E-04	0,015322631
0,001949545	0,026401073
1,18E-05	0,001061429
2,11E-05	0,00154523
2,66E-04	0,007518484
2,24E-04	0,006689908
0,001607892	0,023451846
4,51E-04	0,010459831
0,003593761	0,039264289

6,88E-04	0,013684083
3,47E-04	0,008833663
6,18E-04	0,012741219
0,003997178	0,042112182
7,21E-04	0,014080753
2,31E-06	0,000411684
6,14E-04	0,012676115
8,65E-04	0,015860623
0,002422275	0,030600633
0,002008868	0,026896004
0,002359225	0,030056421
0,002365941	0,030106589
0,004963778	0,04790003
3,93E-04	0,009655384
0,002111168	0,027880183
0,001371182	0,021303779
0,001830407	0,025352507
0,003723081	0,040114248
0,005118786	0,048950456
0,004579993	0,045521965
0,001979174	0,026634533
3,38E-04	0,008677538
0,00108942	0,018560672
9,97E-04	0,017433268
6,90E-05	0,003150412
3,15E-04	0,008303463
0,005041331	0,048445384
0,00331075	0,037458868
4,54E-04	0,010499577
6,48E-06	7,62E-04
0,001395015	0,021516466
0,004966759	0,047908361
8,64E-07	2,43E-04
8,87E-04	0,016145962
2,97E-04	0,008005569
0,001519056	0,022698709
0,00528002	0,049924262
0,002639673	0,032365309
4,51E-05	0,002410418
0,001734831	0,024590606
0,001922085	0,026150688
1,46E-05	0,001214474
0,00151315	0,022629764
2,04E-04	0,006298026
0,001964294	0,026535403
4,75E-04	0,010812604
0,002310686	0,029673229
0,001709082	0,02435422
3,63E-04	0,009093356
0,002937096	0,034673627
9,11E-04	0,016485881
4,12E-05	0,002295678
9,78E-04	0,017228877

0,001149034	0,019155156
6,75E-04	0,013511038
4,90E-05	0,002527132
0,003211448	0,03664209
1,59E-05	0,001271059
0,002729878	0,033037724
2,75E-04	0,007651372
0,00226081	0,029273677
7,18E-05	0,003220404
0,004672099	0,045981879
0,001297472	0,020648298
0,001305084	0,020687854
9,51E-04	0,016928334
7,30E-04	0,014205812
7,42E-04	0,014366294
7,02E-04	0,013825838
0,002778278	0,033375307
5,18E-04	0,011520963
0,004885974	0,047385332
4,18E-04	0,009992372
0,001310214	0,020722957
0,002203728	0,028736201
0,002472473	0,030994418
0,004336636	0,044125511
4,25E-04	0,010084929
8,77E-04	0,016034867
0,003435788	0,038291557
7,17E-05	0,003220404
1,82E-05	0,001392836
0,002770024	0,033294102
0,001138159	0,019042637
1,24E-04	0,004612877
2,14E-04	0,006512592
4,67E-04	0,010690728
9,58E-04	0,016998476
2,06E-04	0,00635911
0,002385045	0,030267654
1,22E-05	0,001083856
0,002366344	0,030106589
0,004962154	0,04790003
9,28E-04	0,016717931
3,51E-04	0,008905894
0,004812789	0,046900214
7,91E-04	0,014950988
0,004605579	0,045629652
0,00231034	0,029673229
0,001750989	0,024711688
4,24E-04	0,010084929
0,004584029	0,045536162
5,60E-04	0,012055378
2,86E-04	0,007769726
3,24E-05	0,001970335
0,002257788	0,029252731

0,004182384	0,043345768
1,87E-04	0,005969942
1,02E-04	0,004076022
0,002953991	0,034782604
0,001183878	0,019524128
0,002273984	0,029385655
0,005138642	0,049077118
0,004323929	0,044057139
0,004252371	0,043766556
7,23E-07	2,30E-04
2,71E-04	0,007609606
0,001147607	0,01913852
5,32E-04	0,011721604
0,003780447	0,040424009
0,001317138	0,020768119
0,002296618	0,029566639
0,002173127	0,028470518
4,80E-05	0,002485274
4,03E-04	0,009799538
0,001783984	0,025021877
0,005160684	0,049204246
3,55E-04	0,008975952
0,001362328	0,021224826
1,04E-04	0,004095153
5,22E-04	0,011582641
0,004915752	0,047632641
2,73E-04	0,00762624
4,82E-06	6,42E-04
0,002843843	0,033906886
0,001401335	0,021546795
0,001022214	0,017724208
0,002647499	0,032398409
0,003128185	0,036014957
3,37E-04	0,008663696
4,89E-05	0,002526561
0,005092308	0,048781028
0,0015829	0,0232544
1,30E-04	0,004770434
0,001908108	0,026024114
0,001996896	0,02679967
0,003317504	0,037481244
0,005248581	0,049690921
0,004117632	0,043028072
0,001686204	0,024104704
0,002436867	0,030715287
0,001359855	0,021216589
3,30E-04	0,00852095
1,97E-04	0,006161377
0,005067902	0,048630898
0,001066196	0,018294399
8,11E-04	0,015200244
9,59E-05	0,0038798
7,15E-04	0,014009871

1,30E-04	0,004770434
0,00346016	0,038426674
0,001014626	0,017640707
0,003152875	0,036178889
0,001602754	0,02342293
0,002405787	0,030470105
0,004497982	0,045014181
6,08E-04	0,012639164
1,56E-06	3,40E-04
1,06E-04	0,00412822
2,99E-06	4,82E-04
1,29E-04	0,00474455
0,002509977	0,031300605
0,0024185	0,030578951
5,63E-04	0,012069922
7,86E-04	0,014882775
0,003200511	0,036573513
3,81E-05	0,002179315
0,004327539	0,044070848
0,003477981	0,03851823
0,001400191	0,021539802
2,68E-04	0,00757574
3,10E-04	0,008216898
2,53E-04	0,007298502
4,08E-05	0,002285797
0,001258771	0,020240478
0,00299964	0,035088221
0,003518037	0,038712308
1,06E-04	0,004139016
2,15E-04	0,006519425
0,002426538	0,030619749
6,46E-04	0,013149017
0,004963521	0,04790003
3,62E-05	0,002091933
0,001603878	0,023426388
0,001610623	0,023481426
0,001985576	0,026677878
0,004600117	0,045625713
0,004085772	0,042792531
4,14E-04	0,009972409
0,001262035	0,020265359
0,002390545	0,030327004
5,95E-04	0,012448848
0,001907995	0,026024114
5,64E-04	0,012069922
0,002434281	0,030691371
0,001644763	0,023775293
0,003494817	0,0385807
0,003625352	0,039470296
0,002037343	0,027162949
0,004950755	0,047870248
2,98E-04	0,008012556
5,79E-04	0,012245694

0,003795443	0,040527535
0,004409734	0,044526934
0,002892702	0,034333201
9,65E-04	0,017065388
5,58E-04	0,012038809
1,62E-04	0,005437884
0,001044783	0,017996454
0,001363164	0,021224826
0,000556966	0,012025919
0,003353326	0,037710316
3,44E-04	0,008779164
9,34E-04	0,016784777
5,01E-04	0,011267972
0,00309085	0,03572362
0,00107192	0,018357401
2,37E-04	0,006957223
0,004300157	0,044022306
0,003465588	0,038466303
0,003730133	0,040148235
2,49E-05	0,001693963
2,18E-06	3,97E-04
8,36E-05	0,003536734
0,001184723	0,019530823
6,35E-05	0,003004509
7,67E-04	0,014672831
5,41E-04	0,011834327
0,003962551	0,041863512
0,001537022	0,02279891
0,001422212	0,021774239
0,003377351	0,037894385
5,78E-05	0,00281815
6,26E-05	0,002969723
0,004147962	0,043200097
0,001763761	0,02481521
0,004697186	0,046177768
8,97E-04	0,016261879
0,003392849	0,038029959
4,14E-05	0,002295678
3,57E-04	0,008998966
0,002815719	0,033688795
0,00202037	0,026977021
0,001115676	0,018831632
0,002344373	0,029921995
0,003133677	0,036040925
6,00E-04	0,012513933
0,001575852	0,023181892
9,34E-04	0,016784777
0,003274929	0,037204392
2,12E-04	0,006473832
2,10E-04	0,006411917
7,77E-04	0,014772651
0,003684607	0,039885092
1,91E-04	0,006024593

0,003558229	0,039019636
4,28E-06	5,92E-04
5,92E-04	0,012401212
0,001524883	0,022734408
6,24E-04	0,012836055
0,003492988	0,038570065
0,002661247	0,032495221
4,63E-05	0,002438348
0,004293107	0,044012005
6,85E-04	0,013662559
0,002091399	0,027698834
0,004079181	0,042750956
0,002834182	0,033832436
0,004389934	0,044432451
0,001066598	0,018294399
0,001977319	0,026629479
9,14E-04	0,016525274
0,001931841	0,026227345
2,56E-04	0,007369432
0,001134126	0,018999057
0,001170508	0,019360979
0,002031777	0,027112609
0,005209778	0,049512338
4,41E-05	0,002382911
5,65E-05	0,002769697
0,003367941	0,037807857
0,005097642	0,048800639
5,99E-04	0,012507238
3,86E-05	0,00219759
0,001835582	0,025384717
0,00147767	0,022274557
2,39E-05	0,001656458
6,55E-04	0,013279859
0,003511741	0,038661921
3,74E-05	0,002150716
1,72E-06	3,58E-04
1,08E-04	0,004167086
0,004289657	0,04398676
2,45E-05	0,001676124
5,61E-04	0,012058374
2,14E-04	0,006504651
1,95E-04	0,006103403
0,004236065	0,043644536
0,001634646	0,023694657
0,001836497	0,025389482
0,004230685	0,043619232
3,97E-04	0,009705451
5,26E-04	0,011623799
0,003486236	0,038527525
0,001585212	0,02326732
6,05E-04	0,012583736
0,001843932	0,025468558
2,72E-04	0,00761885

0,003999178	0,042123287
1,27E-04	0,004691206
0,00475809	0,046530153
0,0042999	0,044022306
0,001981227	0,026646067
5,40E-04	0,011807297
0,002881596	0,03423788
0,004310379	0,044050124
1,13E-05	0,0010264
0,004302688	0,044022306
0,001997986	0,02679967
2,68E-04	0,007573075
6,78E-07	2,26E-04
0,003850067	0,04099866
8,51E-04	0,015668624
0,004455892	0,044763926
3,26E-04	0,008477337
8,16E-04	0,015268608
0,001265258	0,020273251
7,49E-04	0,014450483
1,55E-04	0,005321469
0,003052302	0,0355177
7,09E-04	0,013915804
5,49E-04	0,011948678
0,00350033	0,038612848
7,58E-04	0,014543309
0,004150836	0,043219923
0,001658253	0,023842872
0,002391084	0,030327004
0,005215726	0,049537166
2,49E-04	0,007213766
7,71E-05	0,00337013
0,004136314	0,04312921
7,86E-04	0,014882775
5,50E-06	6,94E-04
2,49E-04	0,007213766
0,001551638	0,022939322
5,91E-04	0,012391163
0,001049508	0,018056885
0,001023519	0,017728772
6,11E-05	0,002932428
0,005172451	0,049283773
0,001526491	0,022741149
1,60E-04	0,005400576
0,00433404	0,044125511
0,005179839	0,049322516
0,001304937	0,020687854
4,39E-04	0,010291188
1,52E-04	0,005270661
0,001585677	0,02326732
0,001931556	0,026227345
0,003026379	0,035317539
3,33E-04	0,008570208

0,001390525	0,021484405
4,25E-04	0,010088567
9,38E-04	0,016823018
0,004323681	0,044057139
0,001946724	0,02637313
4,71E-05	0,002458825
0,001793954	0,025061851
0,003445401	0,038329654
8,12E-04	0,01520184
0,001682264	0,024063833
0,003672726	0,039785485
3,00E-04	0,008040175
5,27E-04	0,011631738
8,83E-04	0,016091292
2,91E-04	0,00787259
0,004978757	0,047988148
0,001076964	0,018422534
0,001942251	0,026336862
7,96E-04	0,014994559
0,004622195	0,045689369
4,39E-04	0,010291188
0,004100242	0,042883582
0,001107065	0,018771646
0,001489719	0,022387997
0,004166227	0,043268926
5,25E-04	0,01161901
0,005275096	0,049909489
0,002699773	0,03283065
0,004276709	0,043925254
4,26E-05	0,002334633
0,004152656	0,043222377
0,001627889	0,023642872
0,001373294	0,021314731
2,59E-04	0,007413927
8,24E-04	0,015357341
8,43E-04	0,015594173
0,001128818	0,018952869
4,83E-04	0,010945766
0,002975949	0,03490276
2,90E-04	0,007855215
5,34E-04	0,011749934
5,13E-06	6,62E-04
9,17E-05	0,003739465
4,34E-04	0,010215739
0,001626638	0,023632412
0,002697419	0,032810988
0,001164387	0,019303069
0,004310581	0,044050124
0,00186114	0,025603035
0,002945843	0,034740424
0,004408803	0,044526934
0,001159054	0,019250152
0,00495581	0,047885375

2,90E-06	4,70E-04
0,004728229	0,046324648
0,004972304	0,047951138
0,001307468	0,020711193
0,003753563	0,040292468
0,003620916	0,039454522
2,80E-04	0,007735047
0,004040084	0,042481132
0,001903469	0,025976754
3,75E-04	0,009305519
8,28E-04	0,01538217
0,004520762	0,045144306
3,08E-04	0,008176057
0,001583392	0,0232544
7,01E-04	0,013813889
0,002380755	0,030221812
0,003687131	0,039893015
0,002322259	0,029746753
0,002377126	0,030201568
0,002960971	0,034837167
0,002792675	0,033521135
0,001469198	0,022199283
0,003619602	0,039449849
0,003306751	0,037456486
1,56E-04	0,005322108
0,002089675	0,027694876
1,37E-04	0,004938084
6,21E-04	0,012795968
2,82E-04	0,007759204
0,002143386	0,028213653
0,003174403	0,036331092
0,001084318	0,018505696
0,003410563	0,038170886
0,002394399	0,030360405
1,11E-04	0,004239262
7,91E-04	0,014953713
3,07E-04	0,008161353
0,001671574	0,023957078
4,94E-04	0,011134909
0,005005745	0,048211005
5,79E-04	0,012241441
0,00131948	0,020768523
0,003003285	0,035112418
0,002444313	0,030783005
7,79E-05	0,00339329
0,00259672	0,031979066
0,003425642	0,038263473
0,001729736	0,024543788
5,92E-04	0,012401157
5,46E-05	0,002695226
5,84E-04	0,012311296
0,00163677	0,023708445
4,08E-05	0,002285797

4,48E-04	0,010398045
0,001300518	0,020651518
2,15E-04	0,006513156
4,54E-04	0,010505805
3,50E-04	0,008889481
9,30E-04	0,016723162
0,001336106	0,020956959
0,002102483	0,027798459
0,005108911	0,048898012
0,001623714	0,02359944
0,002054682	0,027353185
7,28E-04	0,014192911
6,69E-04	0,013459969
0,004756589	0,046530153
1,27E-04	0,004678451
0,002085818	0,027660213
0,003603781	0,039325529
8,85E-04	0,016113681
0,004467408	0,044829065
8,34E-04	0,015461534
0,002036502	0,027159869
0,002194738	0,02866933
0,001450314	0,022033696
0,001751965	0,024711688
0,002196233	0,028672035
5,53E-04	0,011999145
2,45E-05	0,001676453
0,001730498	0,024543788
3,07E-04	0,008161353
0,002082896	0,02763793
0,001219204	0,019839004
0,003435431	0,038291557
6,70E-04	0,013480137
1,87E-04	0,005973147
6,49E-04	0,013183055
0,00455805	0,045401115
0,003284092	0,03728947
5,22E-04	0,011582641
1,63E-04	0,005463718
0,002557079	0,03163959
0,002311225	0,029673229
0,002903845	0,034406175
0,003653838	0,039638719
1,97E-04	0,006161377
0,004173948	0,043320001
0,002656497	0,032472829
0,001394564	0,021516466
5,67E-04	0,012117436
0,00231943	0,029731417
0,00306778	0,035591387
0,005012398	0,048222952
7,07E-04	0,013892214
4,21E-04	0,010043777

0,001831806	0,025362448
2,72E-04	0,007613874
0,002442512	0,030769022
7,94E-04	0,014979276
0,004755176	0,046530153
0,002757976	0,033194079
1,07E-04	0,004154984
4,38E-04	0,010280709
0,004393968	0,044452625
0,003499144	0,038609332
0,00124787	0,020125971
0,001054162	0,018122969
6,72E-04	0,01349285
0,001818332	0,025263805
4,97E-04	0,011186736
0,003646021	0,039583206
5,51E-04	0,01196545
0,002831472	0,033813702
0,004982041	0,048003471
7,58E-06	8,23E-04
6,12E-04	0,012657493
0,001828611	0,025343382
4,74E-04	0,010790451
1,04E-04	0,004103487
0,001019839	0,017689924
0,004214654	0,04353839
2,71E-04	0,007609606
0,002800897	0,033582054
6,26E-04	0,012868205
5,36E-04	0,011767563
0,004928583	0,047725977
1,65E-04	0,005521668
4,36E-04	0,010251693
0,004163972	0,043265678
3,15E-04	0,008303463
4,07E-04	0,009842211
4,68E-05	0,002454888
0,001885919	0,025832217
0,004369042	0,044336631
0,00112634	0,0189374
0,004763657	0,046545917
0,002769732	0,033294102
6,53E-04	0,013252761
0,001618239	0,023564177
0,002732913	0,033053188
0,002890977	0,03432188
6,56E-04	0,013291068
0,003120954	0,035959592
0,001499098	0,022498591
8,97E-05	0,003687375
9,39E-04	0,016823018
0,004932503	0,0477534
0,002559654	0,031662659

0,002543138	0,031549285
0,001299597	0,020651518
0,001673769	0,023973111
2,34E-04	0,006879052
0,002623694	0,032230315
0,003310301	0,037458868
0,004433081	0,044650533
5,53E-04	0,011999145
4,72E-04	0,010752547
0,004507069	0,045064623
4,86E-04	0,010996548
5,68E-04	0,012137467
3,60E-04	0,009031112
0,004841525	0,047036177
0,004428868	0,044644282
0,002573048	0,03176919
0,003626859	0,039471001
0,004216476	0,043547112
9,30E-04	0,016723162
0,001009143	0,017572821
0,001592172	0,023333191
0,004594355	0,04560975
0,001900568	0,025945864
0,003567424	0,039062766
0,00189687	0,025918464
0,003795817	0,040527535
1,24E-04	0,00462176
0,003641939	0,039557801
0,001529892	0,022754778
0,005085687	0,048749053
2,06E-04	0,006347481
3,02E-04	0,008078368
0,004286613	0,043985926
7,95E-04	0,014990935
0,00143674	0,02193219
0,005091631	0,048781028
0,002904257	0,034406175
0,003337282	0,037592756
0,001865823	0,025643697
0,004740736	0,046421491
0,004379987	0,044351479
6,72E-04	0,013489501
2,08E-04	0,006393518
0,004352006	0,044218926
0,001751931	0,024711688
0,003062369	0,035588358
3,06E-04	0,008157315
0,002012172	0,026907889
0,00117313	0,019389949
3,81E-04	0,009415446
0,004203029	0,043475742
0,002551836	0,031618632
7,63E-05	0,003352357

0,001010398	0,01758093
0,003678353	0,039836755
0,002482936	0,031076115
0,004532216	0,045214564
0,004976495	0,047981165
0,005144922	0,049105512
0,003152982	0,036178889
0,002836079	0,033832436
0,002639708	0,032365309
0,001606526	0,023448548
1,11E-05	0,001024278
0,001124965	0,0189374
0,001918852	0,026122662
0,002803855	0,033583853
0,002333881	0,029839382
0,003366911	0,037805816
0,00228264	0,029463311
0,004089803	0,0428072
0,003298609	0,037392715
0,002092446	0,027698834
2,81E-04	0,007751197
0,00180854	0,025190624
7,46E-04	0,014408568
0,001032064	0,017811796
5,18E-05	0,002604444
0,004537685	0,045248855
0,00495102	0,047870248
0,001547258	0,022882163
0,005221646	0,049551127
0,002953561	0,034782604
0,002970075	0,034879204
0,005082123	0,048735866
0,001310537	0,020722957
0,001469686	0,022199283
0,002038272	0,027166206
0,001449204	0,022032446
0,002522938	0,031400318
0,002681229	0,032667575
5,75E-04	0,012214826
4,24E-04	0,010081807
0,001106097	0,018771646
0,001113777	0,018816132
0,002543406	0,031549285
0,004499904	0,0450233
3,06E-05	0,001915156
0,003319999	0,037481244
0,001747498	0,024703447
7,68E-05	0,003364229
5,85E-05	0,002837575
0,002161722	0,028354466
0,001391139	0,021486439
0,002351947	0,029992883
0,001218819	0,019839004

0,001122621	0,018927333
0,00466777	0,045959583
0,004556081	0,045391652
8,64E-04	0,015857839
8,43E-04	0,015594173
0,003580316	0,039165418
0,004758958	0,046530153
0,001693973	0,024192478
0,00242388	0,030609623
0,003774669	0,040398574
0,00277413	0,033334466
0,003318668	0,037481244
0,001240194	0,02005198
0,001236909	0,020027944
0,001271614	0,020353128
0,00129043	0,02057299
0,0017852	0,025021877
8,72E-04	0,015953486
0,004063348	0,042657971
0,001900623	0,025945864
0,002198556	0,028690769
0,003050981	0,035516784
7,64E-04	0,014638178
0,001816635	0,025263805
0,002931492	0,034634759
0,001064155	0,018273606
0,001955022	0,026461413
0,002679509	0,032655552
0,00157783	0,023203326
0,004343928	0,044167066
4,06E-04	0,009835591
0,002156824	0,028340286
0,004076253	0,042733022
2,91E-04	0,007875447
0,002327349	0,029790072
0,004232272	0,043619232
0,001439636	0,021946324
0,003602283	0,039322658
0,001513426	0,022629764
6,93E-04	0,013724255
0,005178491	0,049320227
0,002161708	0,028354466
0,003724689	0,040114248
0,003098214	0,035762334
0,001715168	0,024409086
1,32E-04	0,004810941
0,001460623	0,022137465
0,002190925	0,028636321
0,00341966	0,038225654
1,67E-04	0,005552262
0,005048932	0,048490649
0,003610904	0,039383965
0,00412638	0,043080646

0,001895229	0,02590398
2,78E-04	0,007702264
0,00235064	0,029984793
0,003132869	0,036040925
0,004488714	0,044957809
4,43E-04	0,01031814
0,002278889	0,029431956
1,05E-05	9,92E-04
0,001250263	0,020141666
2,33E-04	0,00687271
0,002533063	0,031473572
0,00421889	0,043551851
0,002692219	0,032759963
0,003133327	0,036040925
0,00386042	0,041079444
0,002421415	0,030600633
0,002739378	0,033078498
0,003585748	0,039206885
0,001112746	0,01881068
0,002735315	0,033060917
0,004467333	0,044829065
0,004204789	0,043475742
9,99E-05	0,004003347
4,40E-04	0,010291188
0,004036644	0,042467703
0,002416983	0,030568455
0,004115357	0,043021465
0,002584491	0,03187253
0,001298003	0,020649366
0,001767592	0,024853421
0,002408375	0,030482475
3,93E-04	0,009656822
0,001858029	0,025571453
0,001059933	0,018215151
2,73E-04	0,00762624
0,004618848	0,045669692
2,83E-04	0,007759204
0,001140029	0,01904781
0,001005547	0,017544498
0,002742892	0,033084044
0,001320963	0,020777184
0,002493803	0,031177086
1,93E-04	0,006055447
0,004186005	0,043373204
0,005284315	0,049943673
0,001132624	0,018991869
7,63E-04	0,014610922
0,001019267	0,017686879
0,005093971	0,048784879
0,001212608	0,019799998
0,004604133	0,045625713
0,002218123	0,028856324
0,003553487	0,03897723

0,003544864	0,038924547
6,94E-04	0,013734888
0,003914817	0,04148648
0,005124801	0,048976433
0,003625908	0,039470296
5,30E-04	0,011699133
0,004245874	0,043728635
0,003818581	0,04073306
0,002569656	0,031756406
0,004569445	0,045494415
0,003963237	0,041863512
0,001305531	0,020687854
0,002730243	0,033037724
7,69E-05	0,003364229
0,004785128	0,046684697
2,47E-04	0,007178612
0,004634975	0,045763928
0,002940736	0,034699901
0,002802836	0,033583853
0,001491223	0,022395495
0,002285354	0,029472729
0,003740358	0,040205241
0,004778415	0,04665711
0,002151725	0,028300541
0,002573259	0,03176919
0,001395931	0,021523135
0,004065126	0,042666581
0,004205868	0,043475742
0,001684514	0,024088282
0,003773612	0,040396969
0,002575124	0,031783409
0,002323297	0,029746753
0,00352632	0,038765156
0,001373866	0,021314731
0,003501494	0,038614365
0,002579013	0,031813784
0,001735652	0,024590606
0,004324144	0,044057139
0,001536084	0,02279258
0,004601948	0,045625713
0,003925182	0,041560093
0,002475898	0,031012437
0,004225528	0,043590067
0,00298701	0,034967748
0,003347677	0,037665803
0,004224238	0,043586852
2,93E-04	0,007912851
0,001672277	0,02395944
0,002835816	0,033832436
0,003015708	0,035211452
0,002217541	0,028856324
3,40E-04	0,008716565
0,004713173	0,046262045

0,003487405	0,038527525
0,002176916	0,028478286
1,04E-04	0,004103487
0,002674262	0,032618377
0,002457677	0,030908484
2,84E-04	0,007759204
1,96E-04	0,006141048
0,003952002	0,04177456
0,004632334	0,045762839
0,003521214	0,038728136
0,001303518	0,020678024
0,003779979	0,040424009
4,95E-04	0,011164485
0,004403741	0,044500959
0,004600156	0,045625713
5,72E-04	0,012188658
0,005211999	0,049522888
0,003318042	0,037481244
2,85E-04	0,007759204
0,002368523	0,030118042
8,55E-04	0,015738799
2,82E-04	0,007759204
0,002151893	0,028300541
0,004147306	0,043200097
0,001409795	0,021632066
0,00135267	0,021141526
0,002453461	0,030872029
0,004092635	0,042814077
0,004450155	0,044754424
0,003911878	0,041484741
3,26E-04	0,008484423
0,003602346	0,039322658
8,61E-04	0,015823685
0,001569293	0,023120972
0,003222392	0,036748139
0,002586202	0,031884795
0,003325261	0,03751784
0,001186112	0,019538685
0,001647047	0,023775293
0,003067537	0,035591387
0,004177288	0,043323191
0,002210669	0,028794261
0,001528786	0,022752499
0,003415596	0,038217611
0,002489493	0,031140682
0,00329062	0,037335046
0,005197717	0,049439911
0,004698915	0,04618457
4,60E-04	0,010578988
0,003349387	0,037675528
0,002206554	0,02875621
5,12E-04	0,011426534
0,005206515	0,049502454

0,003738517	0,040195154
0,003965792	0,041880565
0,002088086	0,027682058
0,004478283	0,044887637
0,003011246	0,035183003
0,004363832	0,044318858
4,21E-04	0,010043777
0,004297276	0,044022306
0,001554567	0,022967372
0,004966793	0,047908361
4,37E-04	0,010251693
0,001110626	0,018793982
0,004011452	0,042242579
0,00281094	0,033640656
0,004351241	0,044218926
9,28E-04	0,016717931
0,004398324	0,044486586
0,003404961	0,038136935
0,00328179	0,037272835
0,00445175	0,044754424
0,003158817	0,036217847
7,66E-04	0,014662644
4,52E-04	0,010473851
0,001258763	0,020240478
0,001293755	0,02061018
0,00457863	0,045521965
0,00118264	0,019510932
0,003609243	0,039375488
0,004046049	0,042526474
0,001370065	0,021293858
0,003289884	0,037335046
0,001562059	0,023039854
0,001354961	0,021169892
0,001378792	0,021347549
0,003489223	0,038538044
0,00265628	0,032472829
0,003102436	0,035792929
0,003721006	0,040110054
0,002531057	0,031457426
3,40E-07	1,39E-05
2,97E-05	5,74E-04
4,69E-04	0,005354007
6,48E-06	1,64E-04
6,11E-07	2,27E-05
1,15E-05	2,64E-04
1,25E-08	9,30E-07
0,002035004	0,016877408
2,31E-05	4,66E-04
1,92E-04	0,002625626
3,24E-06	9,23E-05
4,18E-04	0,004891921
0,003642309	0,026271146
1,06E-04	0,001612446

4,91E-06	1,31E-04
2,37E-05	4,76E-04
1,14E-05	2,64E-04
2,29E-04	0,003012816
1,20E-06	4,04E-05
3,39E-06	9,57E-05
9,63E-06	2,28E-04
2,04E-06	6,32E-05
0,002055648	0,017003919
1,35E-05	3,02E-04
3,16E-07	1,32E-05
5,48E-06	1,44E-04
0,002271954	0,018429155
4,76E-06	1,28E-04
0,007714309	0,046322071
5,77E-07	2,16E-05
2,05E-04	0,002762548
1,14E-13	6,93E-11
9,19E-09	7,27E-07
3,62E-04	0,0043475
0,001526618	0,013458301
4,39E-05	7,87E-04
7,25E-04	0,007546525
7,77E-05	0,00125915
2,37E-04	0,0030924
0,001872612	0,015799183
2,49E-11	6,14E-09
8,59E-05	0,001363396
6,39E-07	2,36E-05
8,62E-08	4,51E-06
8,49E-04	0,008547689
1,37E-04	0,002000202
6,85E-05	0,001130419
5,16E-04	0,005782885
4,03E-07	1,62E-05
5,63E-05	9,65E-04
6,01E-04	0,006526248
6,75E-05	0,001117472
3,50E-05	6,56E-04
1,55E-04	0,002204961
0,001198099	0,011115545
1,23E-04	0,00182043
4,71E-05	8,34E-04
0,004648667	0,031703249
7,23E-07	2,63E-05
1,61E-08	1,14E-06
2,07E-05	4,27E-04
2,78E-07	1,19E-05
6,66E-04	0,007061723
2,26E-05	4,59E-04
1,35E-06	4,46E-05
0,005277147	0,034820193
3,44E-04	0,004192843

0,00457012	0,031293846
4,79E-05	8,45E-04
1,29E-04	0,001897486
1,13E-05	2,62E-04
0,003124592	0,023465141
2,79E-04	0,003544198
5,47E-04	0,00605356
2,60E-05	5,14E-04
2,28E-05	4,62E-04
8,57E-04	0,008607667
3,69E-04	0,004420096
6,20E-06	1,58E-04
4,43E-04	0,005122897
1,01E-06	3,48E-05
2,25E-11	5,71E-09
4,93E-04	0,00557171
1,52E-04	0,002172887
0,001522174	0,01343414
4,23E-07	1,67E-05
4,26E-05	7,69E-04
4,40E-05	7,90E-04
1,29E-06	4,30E-05
2,52E-04	0,003256891
3,49E-06	9,83E-05
1,42E-05	3,15E-04
7,60E-05	0,001235203
1,31E-06	4,35E-05
5,82E-04	0,006349659
8,72E-08	4,56E-06
5,21E-05	9,04E-04
5,41E-04	0,006006055
5,02E-05	8,78E-04
8,65E-05	0,001371831
2,33E-04	0,003052723
0,001810181	0,015395969
1,68E-05	3,59E-04
7,80E-05	0,001261629
3,70E-05	6,86E-04
3,89E-04	0,004620309
0,001657418	0,014378366
3,33E-04	0,004087251
6,10E-04	0,006601397
1,37E-07	6,67E-06
1,07E-05	2,48E-04
1,39E-06	4,56E-05
0,001242296	0,011442037
2,03E-06	6,29E-05
0,001200325	0,011131293
4,98E-07	1,91E-05
1,36E-05	3,03E-04
2,17E-04	0,002894101
2,02E-04	0,002727523
6,31E-05	0,001056984

4,84E-05	8,53E-04
4,60E-09	4,10E-07
7,40E-10	9,52E-08
6,71E-04	0,00710691
2,97E-06	8,60E-05
3,19E-05	6,09E-04
3,49E-07	1,42E-05
0,001158392	0,010842755
3,34E-04	0,004087251
8,42E-04	0,008496684
0,001185479	0,011023864
4,67E-06	1,26E-04
2,16E-05	4,42E-04
5,33E-10	7,35E-08
6,41E-05	0,001071665
3,13E-04	0,003889242
0,001243425	0,011450201
1,39E-07	6,77E-06
3,13E-04	0,003889242
0,001753338	0,015042277
8,20E-05	0,001311074
0,002354533	0,018956034
1,28E-06	4,26E-05
7,08E-05	0,001163048
7,47E-05	0,001216901
5,19E-05	9,00E-04
8,56E-04	0,008596826
1,15E-07	5,78E-06
2,33E-09	2,33E-07
4,24E-05	7,67E-04
1,63E-06	5,21E-05
7,18E-05	0,001178368
3,21E-04	0,003972859
2,74E-17	1,62E-13
5,88E-04	0,006412217
1,09E-08	8,34E-07
2,77E-08	1,78E-06
3,29E-04	0,004048616
6,48E-05	0,001079201
2,50E-05	4,97E-04
0,002529953	0,01999749
1,07E-04	0,001623086
1,32E-05	2,97E-04
1,81E-04	0,002506106
7,53E-09	6,12E-07
0,002693637	0,020982879
1,94E-04	0,002657345
1,06E-04	0,001611026
3,06E-04	0,003823825
0,007327041	0,044699954
8,67E-05	0,001374272
0,002111924	0,017402505
5,30E-04	0,005909947

4,54E-06	1,23E-04
1,28E-04	0,001892159
7,79E-04	0,007994096
1,85E-07	8,55E-06
3,93E-06	1,09E-04
0,008067954	0,04788659
5,00E-04	0,00563742
0,001359256	0,012274801
1,12E-06	3,81E-05
1,22E-04	0,001819321
2,96E-05	5,72E-04
4,50E-08	2,66E-06
0,001573642	0,01380086
4,51E-10	6,42E-08
0,002529903	0,01999749
0,001284868	0,011758619
0,002336422	0,018829441
2,62E-04	0,003371267
2,55E-06	7,61E-05
0,00122861	0,011357527
2,49E-08	1,64E-06
1,51E-04	0,002158707
4,02E-04	0,004736826
8,14E-04	0,008261043
4,30E-05	7,75E-04
0,001273859	0,011683958
0,002694895	0,02098922
7,56E-04	0,007803246
1,65E-09	1,76E-07
0,001341018	0,012154953
2,00E-05	4,14E-04
0,00347481	0,025360644
1,35E-05	3,01E-04
3,86E-05	7,10E-04
1,19E-09	1,36E-07
5,73E-11	1,23E-08
4,02E-06	1,11E-04
5,88E-04	0,006409575
1,11E-09	1,30E-07
0,00333994	0,024623151
4,74E-07	1,83E-05
7,18E-04	0,007493067
0,007193174	0,044115711
5,00E-05	8,76E-04
0,001324187	0,012034666
1,13E-09	1,30E-07
8,13E-06	1,98E-04
0,001537824	0,013539427
9,34E-06	2,22E-04
4,65E-06	1,26E-04
2,65E-05	5,22E-04
9,76E-04	0,009500354
3,81E-04	0,004546991

6,36E-04	0,006822034
0,004542931	0,031157266
0,002608756	0,020463143
0,001672613	0,014483634
1,57E-06	5,05E-05
1,04E-06	3,57E-05
6,13E-08	3,43E-06
5,46E-06	1,43E-04
2,09E-08	1,42E-06
1,23E-04	0,001825858
0,001299524	0,01186521
1,53E-06	4,96E-05
2,42E-07	1,07E-05
5,97E-07	2,23E-05
6,90E-04	0,007267999
8,01E-06	1,96E-04
4,15E-06	1,14E-04
0,001481532	0,013149012
1,49E-07	7,18E-06
6,54E-04	0,006972785
0,001242108	0,011442037
5,78E-04	0,006311931
1,06E-06	3,61E-05
6,66E-07	2,44E-05
0,003576611	0,025910319
2,69E-06	7,91E-05
0,001788646	0,015245692
0,007857679	0,046968415
0,001067817	0,010167464
6,81E-09	5,67E-07
1,45E-04	0,002088884
0,001990063	0,016588997
8,62E-11	1,70E-08
7,04E-05	0,001157544
1,18E-04	0,001760126
1,03E-04	0,001568774
2,86E-05	5,54E-04
0,004368184	0,030252537
0,005994788	0,038414538
1,18E-04	0,001759162
7,44E-04	0,00770221
4,88E-04	0,005528131
7,25E-06	1,80E-04
5,22E-11	1,13E-08
2,65E-04	0,00339641
9,12E-06	2,18E-04
0,003337586	0,024609629
2,90E-07	1,23E-05
2,03E-05	4,19E-04
3,31E-04	0,004068835
0,001069449	0,010178912
6,06E-05	0,001023502
1,71E-05	3,65E-04

9,18E-04	0,009087822
0,003317993	0,024530135
1,41E-04	0,002045186
2,03E-04	0,002745538
9,69E-05	0,001497372
2,07E-04	0,002780151
1,94E-10	3,21E-08
0,00128445	0,011757065
2,32E-04	0,00304943
1,36E-04	0,016882991
1,78E-04	0,019439782
9,33E-05	0,013579379
4,90E-04	0,034305533
1,29E-06	0,001152854
5,23E-04	0,035505411
3,39E-04	0,027998024
7,22E-04	0,042358384
1,30E-04	0,016429027
1,80E-04	0,019484542
1,43E-04	0,017266589
1,26E-05	0,004226654
8,10E-05	0,012443541
2,26E-04	0,022206068
1,22E-04	0,015760865
5,08E-07	7,67E-04
8,44E-04	0,046503963
1,58E-04	0,018352647
1,20E-04	0,015601692
2,27E-04	0,022213247
2,06E-04	0,021106946
2,81E-05	0,006992473
9,57E-04	0,049982263
4,94E-04	0,034317837
6,92E-05	0,011523876
8,03E-04	0,044891022
1,03E-04	0,014398836
2,21E-05	0,005964272
4,65E-04	0,033513331
7,49E-04	0,043227023
7,80E-04	0,044317107
2,99E-04	0,026094038
9,34E-06	0,003535583
1,78E-04	0,019439782
1,01E-05	0,003672926
3,31E-04	0,027861269
3,12E-04	0,026702677
6,32E-04	0,039216081
8,14E-05	0,01246144
9,55E-04	0,049982263
5,94E-05	0,010417307
3,13E-06	0,001751528
5,05E-06	0,002411591
5,93E-04	0,037986282

1,87E-04	0,019927339
5,24E-04	0,035505411
8,88E-04	0,047765231
5,23E-05	0,00951812
1,36E-04	0,016892705
2,73E-04	0,024809457
1,23E-04	0,015865113
4,62E-04	0,033513331
4,40E-04	0,032646375
3,36E-04	0,027973661
8,92E-04	0,047826835
3,08E-04	0,026550941
8,19E-05	0,01247171
3,02E-05	0,00708452
4,76E-04	0,03378353
8,09E-05	0,012443541
2,02E-05	0,005729914
3,72E-04	0,029190575
2,15E-05	0,005847929
6,63E-05	0,01120797
4,88E-06	0,002354376
6,05E-07	7,75E-04
2,16E-04	0,021595182
2,51E-04	0,023542712
1,78E-04	0,019439782
6,10E-04	0,03854472
1,70E-06	0,001295115
2,48E-04	0,023488274
1,24E-05	0,004226654
3,98E-04	0,030412786
2,05E-05	0,005729914
7,31E-05	0,011730084
5,02E-04	0,034476343
9,09E-05	0,013402036
3,23E-04	0,02739287
7,23E-05	0,011638237
6,99E-04	0,041601568
1,05E-04	0,014518195
4,56E-05	0,008837876
6,58E-05	0,01120797
1,11E-04	0,015115844
2,46E-04	0,02344981
8,16E-04	0,045485195
2,94E-04	0,025757313
4,37E-04	0,032533679
2,48E-04	0,023488274
1,18E-04	0,015492309
3,73E-04	0,029190575
1,67E-04	0,018864685
3,17E-05	0,00721118
2,99E-06	0,001751528
7,33E-04	0,042800566
1,28E-05	0,004226654

6,67E-04	0,040629127
2,84E-04	0,025373872
5,01E-05	0,00937219
2,81E-04	0,025262574
2,73E-04	0,024809457
4,16E-05	0,008263491
5,10E-05	0,009373727
5,07E-04	0,034745851
7,05E-04	0,041723078
3,53E-04	0,028507442
1,05E-04	0,014554497
8,85E-05	0,013165715
7,03E-04	0,041662442
6,05E-04	0,0383575
4,98E-05	0,009356118
3,31E-05	0,007354242
7,03E-04	0,041662442
2,97E-04	0,025957956
5,68E-04	0,037379805
2,35E-04	0,022688356
1,02E-04	0,014338997
5,77E-04	0,037633883
1,92E-04	0,020193039
4,61E-04	0,033513331
1,54E-04	0,017959918
