# Supplementary material for: The Impact of Cand1 in Prostate Cancer
Source: Cancers (Basel). 2020 Feb 12;12(2):428. doi: 10.3390/cancers12020428 (PMC7072594; doi:10.3390/cancers12020428)
Supplement: Supplementary file 1 [file cancers-12-00428-s001.zip › Supplementary material revision/Supplementary_table_S2.pdf]

Study	Probe ID	Entrez Gene ID	Gene Symbol
GSE16560	DAP3_4247	4751	NEK2
GSE16560	DAP1_1286	8357	HIST1H3H
GSE16560	DAP4_0822	3781	KCNN2
GSE16560	DAP1_2789	55832	CAND1
GSE16560	DAP1_4872	10615	SPAG5
GSE16560	DAP1_1629	4001	LMNB1
GSE16560	DAP4_5741	7153	TOP2A
GSE16560	DAP3_3833	23397	NCAPH
GSE16560	DAP3_4041	332	BIRC5
GSE16560	DAP1_4061	1033	CDKN3
GSE16560	DAP4_2995	3110	HLXB9
GSE16560	DAP1_5683	5999	RGS4
GSE16560	DAP1_4207	547	KIF1A
GSE16560	DAP1_3670	11065	UBE2C
GSE16560	DAP4_4256	10609	SC65
GSE16560	DAP1_2729	6241	RRM2
GSE16560	DAP4_3996	9232	PTTG1
GSE16560	DAP2_3709	51053	GMNN
GSE16560	DAP2_3992	1063	CENPF
GSE16560	DAP3_2180	1719	DHFR
GSE16560	DAP1_5047	1397	CRIP2
GSE16560	DAP1_2811	9319	TRIP13
GSE16560	DAP1_4002	891	CCNB1
GSE16560	DAP4_5736	5387	PMS2L3
GSE16560	DAP3_6035	51203	NUSAP1
GSE16560	DAP4_5055	9768	KIAA0101
GSE16560	DAP3_1787	4173	MCM4
GSE16560	DAP1_2954	4950	OCLN
GSE16560	DAP3_0240	3092	HIP1
GSE16560	DAP4_5744	5707	PSMD1
GSE16560	DAP4_1493	11130	ZWINT
GSE16560	DAP1_0021	2146	EZH2
GSE16560	DAP1_4305	3251	HPRT1
GSE16560	DAP1_2136	9787	DLG7
GSE16560	DAP2_3314	4085	MAD2L1
GSE16560	DAP3_4902	10499	NCOA2
GSE16560	DAP1_2721	2961	GTF2E2
GSE16560	DAP3_3377	9791	PTDSS1
GSE16560	DAP3_0270	2176	FANCC
GSE16560	DAP3_1711	9735	KNTC1
GSE16560	DAP3_1685	56967	C14orf132
GSE16560	DAP3_3105	4217	MAP3K5
GSE16560	DAP3_1740	7323	UBE2D3
GSE16560	DAP3_3526	3013	HIST1H2AD
GSE16560	DAP1_4068	7083	TK1
GSE16560	DAP3_2174	10987	COPS5
GSE16560	DAP3_4872	8667	EIF3S3
GSE16560	DAP1_3693	483	ATP1B3
GSE16560	DAP4_3280	7341	SUMO1
GSE16560	DAP2_0885	2305	FOXM1
GSE16560	DAP3_4248	4947	OAZ2

GSE16560	DAP1_5291	1287	COL4A5
GSE16560	DAP1_1044	10797	MTHFD2
GSE40272	X21134	3043	HBB
GSE40272	X31415	6401	SELE
GSE40272	X19928	6716	SRD5A2
GSE40272	X3112	6943	TCF21
GSE40272	X24202	712	C1QA
GSE40272	X35880	5067	CNTN3
GSE40272	X30188	33	ACADL
GSE40272	X26593	64123	ELTD1
GSE40272	X34760	1113	CHGA
GSE40272	X38453	9560	CCL4L1
GSE40272	X33581	6581	SLC22A3
GSE40272	X40266	126	ADH1C
GSE40272	X29965	3671	ISLR
GSE40272	X40568	2953	GSTT2
GSE40272	X30185	1359	CPA3
GSE40272	X31671	221400	TDRD6
GSE40272	X22881	64123	ELTD1
GSE40272	X8792	6566	SLC16A1
GSE40272	X37427	140458	ASB5
GSE40272	X29823	130271	PLEKHH2
GSE40272	X17314	55303	GIMAP4
GSE40272	X40954	155060	LOC155060
GSE40272	X29222	92344	GORAB
GSE40272	X35939	157381	LOC157381
GSE40272	X7398	285889	LOC285889
GSE40272	X36981	3003	GZMK
GSE40272	X18190	2359	FPR3
GSE40272	X41619	283383	GPR133
GSE40272	X35702	400944	LOC400944
GSE40272	X20897	6713	SQLE
GSE40272	X17377	85028	SNHG12
GSE40272	X12500	256236	NAPSB
GSE40272	X32789	222236	NAPEPLD
GSE40272	X18193	60492	CCDC90B
GSE40272	X10442	284033	LOC284033
GSE40272	X13583	84289	ING5
GSE40272	X16810	2139	EYA2
GSE40272	X7360	3925	STMN1
GSE40272	X12886	27018	NGFRAP1
GSE40272	X20621	2953	GSTT2
GSE40272	X44054	3638	INSIG1
GSE40272	X37110	25805	BAMBI
GSE40272	X13265	133923	ZNF474
GSE40272	X7210	84306	PDCD2L
GSE40272	X2730	1434	CSE1L
GSE40272	X36616	83596	BCL2L12
GSE40272	X29110	84889	SLC7A3
GSE40272	X40224	11104	KATNA1
GSE40272	X27134	4060	LUM
GSE40272	X8662	8334	HIST1H2AC
GSE40272	X9267	1300	COL10A1

GSE40272	X32818	22849	CPEB3
GSE40272	X28738	894	CCND2
GSE40272	X12091	4709	NDUFB3
GSE40272	X35742	51184	GPN3
GSE40272	X19295	51510	CHMP5
GSE40272	X40698	51192	CKLF
GSE40272	X19479	65985	AACS
GSE40272	X20891	11232	POLG2
GSE40272	X41871	25900	IFFO1
GSE40272	X37239	388753	C1orf31
GSE40272	X3456	158586	ZXDB
GSE40272	X35915	7283	TUBG1
GSE40272	X14111	54567	DLL4
GSE40272	X44309	81611	ANP32E
GSE40272	X29220	55778	ZNF839
GSE40272	X31101	5445	PON2
GSE40272	X17970	221656	KDM1B
GSE40272	X33226	85015	USP45
GSE40272	X5886	7485	WRB
GSE40272	X28046	11274	USP18
GSE40272	X24516	375033	PEAR1
GSE40272	X43247	51338	MS4A4A
GSE40272	X39586	55832	CAND1
GSE40272	X40836	6908	TBP
GSE40272	X30817	3976	LIF
GSE40272	X24147	825	CAPN3
GSE40272	X26884	26785	SNORD63
GSE40272	X29441	8089	YEATS4
GSE40272	X40420	283747	TCEB1P2
GSE40272	X25570	57584	ARHGAP21
GSE40272	X1904	5889	RAD51C
GSE40272	X40423	10063	COX17
GSE40272	X26438	169200	TMEM64
GSE40272	X22069	23317	DNAJC13
GSE40272	X40271	55353	LAPTM4B
GSE40272	X32526	285343	C3orf23
GSE40272	X29918	51097	SCCPDH
GSE40272	X26207	80263	TRIM45
GSE40272	X34262	10741	RBBP9
GSE40272	X33081	7556	ZNF10
GSE40272	X19311	83468	GLT8D2
GSE40272	X29677	117155	CATSPER2
GSE40272	X37402	389166	LOC389166
GSE40272	X41149	85459	KIAA1731
GSE40272	X30968	23382	AHCYL2
GSE40272	X40859	6637	SNRPG
GSE40272	X41708	81625	GALIG
GSE40272	X1489	8624	PSMG1
GSE40272	X38617	64968	MRPS6
GSE40272	X44340	79657	RPAP3
GSE40272	X173	6307	SC4MOL
GSE40272	X29844	51575	ESF1
GSE40272	X25959	55012	PPP2R3C

GSE40272	X29046	51559	NT5DC3
GSE40272	X40394	92597	MOBKL1A
GSE40272	X20855	57213	SPRYD7
GSE40272	X35874	55773	TBC1D23
GSE40272	X29376	54898	ELOVL2
GSE40272	X24504	7626	ZNF75D
GSE40272	X36643	55112	WDR60
GSE40272	X36087	3682	ITGAE
GSE40272	X41286	1960	EGR3
GSE40272	X32877	3251	HPRT1
GSE40272	X32874	6908	TBP
GSE40272	X39494	83940	TATDN1
GSE40272	X32268	154141	MBOAT1
GSE40272	X24853	1073	CFL2
GSE40272	X35059	6476	SI
GSE40272	X33254	10038	PARP2
GSE40272	X16268	282679	AQP11
GSE40272	X3550	22948	CCT5
GSE40272	X33721	285961	SEPT7L
GSE40272	X36532	51406	NOL7
GSE40272	X23402	390551	LOC390551
GSE40272	X29165	3251	HPRT1
GSE40272	X22875	23554	TSPAN12
GSE40272	X31665	27031	NPHP3
GSE40272	X36353	79135	APOO
GSE40272	X10398	339804	C2orf74
GSE40272	X18406	29781	NCAPH2
GSE40272	X25541	28978	TMEM14A
GSE40272	X30186	150094	SIK1
GSE40272	X36708	115106	HAUS1
GSE40272	X35988	134492	NUDCD2
GSE40272	X32869	51635	DHRS7
GSE40272	X34571	92126	DSEL
GSE40272	X21388	148646	C1orf188
GSE40272	X38728	6362	CCL18
GSE40272	X14688	286410	ATP11C
GSE40272	X41049	10782	ZNF274
GSE40272	X14645	152189	CMTM8
GSE40272	X44134	157317	CYCSP55
GSE40272	X23239	11164	NUDT5
GSE40272	X29644	3958	LGALS3
GSE40272	X29316	90850	ZNF598
GSE40272	X38719	254778	C8orf46
GSE40272	X32277	126669	SHE
GSE40272	X37903	113510	HELQ
GSE40272	X21951	5217	PFN2
GSE40272	X21480	1278	COL1A2
GSE40272	X20274	104	ADARB1
GSE40272	X40813	55253	TYW1
GSE40272	X3464	51388	NIP7
GSE40272	X15562	11193	WBP4
GSE40272	X14433	131076	CCDC58
GSE40272	X25446	54460	MRPS21

GSE40272	X29448	5411	PNN
GSE40272	X33861	151556	GPR155
GSE40272	X39583	55860	ACTR10
GSE40272	X28319	115353	LRRC42
GSE40272	X29536	7694	ZNF135
GSE40272	X3376	79693	YRDC
GSE40272	X42803	10512	SEMA3C
GSE40272	X39564	51136	RNFT1
GSE40272	X40470	4706	NDUFAB1
GSE40272	X18236	1407	CRY1
GSE40272	X19166	4986	OPRK1
GSE40272	X16773	84233	TMEM126A
GSE40272	X25801	83943	IMMP2L
GSE40272	X25971	9908	G3BP2
GSE40272	X22106	55599	RNPC3
GSE40272	X30308	144811	LACC1
GSE40272	X3949	26504	CNNM4
GSE40272	X5227	91409	CCDC74B
GSE40272	X33994	90557	CCDC74A
GSE40272	X26712	9510	ADAMTS1
GSE40272	X4435	254428	SLC41A1
GSE40272	X23507	80006	C5orf44
GSE40272	X19966	55863	TMEM126B
GSE40272	X4445	1848	DUSP6
GSE40272	X10725	51023	MRPS18C
GSE40272	X42213	120	ADD3
GSE40272	X31896	2043	EPHA4
GSE40272	X5848	8803	SUCLA2
GSE40272	X29050	25829	TMEM184B
GSE40272	X40379	9912	ARHGAP44
GSE40272	X40359	22903	BTBD3
GSE40272	X28314	5732	PTGER2
GSE40272	X4182	56145	PCDHA3
GSE40272	X19707	2222	FDFT1
GSE40272	X33626	121260	SLC15A4
GSE40272	X21332	120376	C11orf93
GSE40272	X39695	54802	TRIT1
GSE40272	X11166	64216	TFB2M
GSE40272	X17616	10438	C1D
GSE40272	X23435	91452	ACBD5
GSE40272	X37356	122809	SOCS4
GSE40272	X39945	84693	MCEE
GSE40272	X12414	115426	UHRF2
GSE40272	X44239	64770	CCDC14
GSE40272	X36586	3251	HPRT1
GSE40272	X31535	10438	C1D
GSE40272	X8963	96459	FNIP1
GSE40272	X25728	1728	NQO1
GSE40272	X40583	51194	IPO11
GSE40272	X12610	3015	H2AFZ
GSE40272	X42072	65981	CAPRIN2
GSE40272	X33890	10287	RGS19
GSE40272	X38636	1466	CSRP2

GSE40272	X11573	5110	PCMT1
GSE40272	X28389	25994	HIGD1A
GSE40272	X9366	22833	KIAA0894
GSE40272	X10702	390	RND3
GSE40272	X40625	9731	CEP104
GSE40272	X39880	60493	FASTKD5
GSE40272	X43095	6047	RNF4
GSE40272	X15157	9652	TTC37
GSE40272	X17311	5552	SRGN
GSE40272	X17256	51362	CDC40
GSE40272	X17065	441198	LOC441198
GSE40272	X39319	1955	MEGF9
GSE40272	X17196	23294	ANKS1A
GSE40272	X7720	25842	ASF1A
GSE40272	X43184	6596	HLTF
GSE40272	X31512	203523	ZNF449
GSE40272	X27512	64788	LMF1
GSE40272	X4320	7360	UGP2
GSE40272	X9434	840	CASP7
GSE40272	X3666	23450	SF3B3
GSE40272	X10253	27107	ZBTB11
GSE40272	X42199	65062	TMEM237
GSE40272	X28965	9873	FCHSD2
GSE40272	X8590	25840	METTL7A
GSE40272	X15394	8894	EIF2S2
GSE40272	X33613	284412	LOC284412
GSE40272	X28904	129531	MITD1
GSE40272	X35389	54850	FBXL12
GSE40272	X16944	51186	WBP5
GSE40272	X44198	55027	HEATR3
GSE40272	X41958	222161	DKFZP586I1420
GSE40272	X35382	6453	ITSN1
GSE40272	X9111	54977	SLC25A38
GSE40272	X38471	10159	ATP6AP2
GSE40272	X11112	55683	KIAA1310
GSE40272	X18536	55746	NUP133
GSE40272	X27640	5189	PEX1
GSE40272	X34830	55148	UBR7
GSE40272	X2970	81034	SLC25A32
GSE40272	X37229	10561	IFI44
GSE40272	X17687	55814	BDP1
GSE40272	X33194	85440	DOCK7
GSE40272	X17769	708	C1QBP
GSE40272	X23417	9267	CYTH1
GSE40272	X37761	91272	BOD1
GSE40272	X25723	5283	PIGH
GSE40272	X39180	5230	PGK1
GSE40272	X17997	51096	UTP18
GSE40272	X22101	8569	MKNK1
GSE40272	X3636	84276	NICN1
GSE40272	X41314	10462	CLEC10A
GSE40272	X22862	10447	FAM3C
GSE40272	X40217	1738	DLD

GSE40272	X35085	48	ACO1
GSE40272	X7534	11266	DUSP12
GSE40272	X30173	64135	IFIH1
GSE40272	X22105	346990	H2AFZP2
GSE40272	X21968	6789	STK4
GSE40272	X14831	55629	PNRC2
GSE40272	X44341	285812	LOC285812
GSE40272	X20134	146880	LOC146880
GSE40272	X20903	55823	VPS11
GSE40272	X32717	135114	HINT3
GSE40272	X13410	10935	PRDX3
GSE40272	X24727	55276	PGM2
GSE40272	X42233	64786	TBC1D15
GSE40272	X40244	51426	POLK
GSE40272	X41732	2744	GLS
GSE40272	X23040	57584	ARHGAP21
GSE40272	X40817	57826	RAP2C
GSE40272	X2464	55907	CMAS
GSE40272	X18872	93487	MAPK1IP1L
GSE40272	X26282	25966	C2CD2
GSE40272	X32414	10440	TIMM17A
GSE40272	X31303	5718	PSMD12
GSE40272	X17423	54414	SIAE
GSE40272	X30092	6389	SDHA
GSE40272	X25105	51522	TMEM14C
GSE40272	X40063	51020	HDDC2
GSE40272	X22746	8505	PARG
GSE40272	X2346	9917	FAM20B
GSE40272	X4460	28972	SPCS1
GSE40272	X3386	7813	EVI5
GSE40272	X8033	6310	ATXN1
GSE40272	X24736	56967	C14orf132
GSE40272	X32904	83440	ADPGK
GSE40272	X34537	119016	AGAP4
GSE40272	X30821	157567	ANKRD46
GSE40272	X34114	51053	GMNN
GSE40272	X11501	25869	DKFZP564M1416
GSE40272	X3838	66036	MTMR9
GSE40272	X32610	1808	DPYSL2
GSE40272	X15638	51669	TMEM66
GSE40272	X34455	2857	GPR34
GSE40272	X39779	25909	AHCTF1
GSE40272	X31985	9079	LDB2
GSE40272	X14393	51315	KRCC1
GSE40272	X3147	55353	LAPTM4B
GSE40272	X8209	10857	PGRMC1
GSE40272	X40527	64333	ARHGAP9
GSE40272	X39008	27258	LSM3
GSE40272	X5843	29097	CNIH4
GSE40272	X43414	483	ATP1B3
GSE40272	X33817	51022	GLRX2
GSE40272	X2376	10434	LYPLA1
GSE40272	X34943	583	BBS2

GSE40272	X11264	143684	FAM76B
GSE40272	X42922	4152	MBD1
GSE40272	X32631	51103	NDUFAF1
GSE40272	X39919	171586	ABHD3
GSE40272	X40416	8925	HERC1
GSE40272	X2161	3091	HIF1A
GSE40272	X42809	90806	ANGEL2
GSE40272	X34674	34	ACADM
GSE40272	X33191	51065	RPS27L
GSE40272	X39390	11098	PRSS23
GSE40272	X16327	6726	SRP9
GSE40272	X31344	57533	TBC1D14
GSE40272	X23008	55848	C9orf46
GSE40272	X35716	60561	RINT1
GSE40272	X5909	85403	EAF1
GSE40272	X2501	257039	LOC257039
GSE40272	X21004	51121	RPL26L1
GSE40272	X21679	5868	RAB5A
GSE40272	X5877	4189	DNAJB9
GSE40272	X37484	81176	OR7E126P
GSE40272	X31741	38	ACAT1
GSE40272	X7545	134492	NUDCD2
GSE40272	X44536	534	ATP6V1G2
GSE40272	X31027	5281	PIGF
GSE40272	X22791	64983	MRPL32
GSE40272	X4766	57185	NIPAL3
GSE40272	X8964	10484	SEC23A
GSE40272	X27459	29103	DNAJC15
GSE40272	X35886	5156	PDGFRA
GSE40272	X3070	7159	TP53BP2
GSE40272	X32757	4820	NKTR
GSE40272	X39147	146443	LOC146443
GSE40272	X15522	1102	RCBTB2
GSE40272	X35888	83608	C18orf21
GSE40272	X18957	567	B2M
GSE40272	X37556	286053	NSMCE2
GSE40272	X31562	134266	GRPEL2
GSE40272	X26406	6814	STXBP3
GSE40272	X8965	23421	ITGB3BP
GSE40272	X43959	1491	CTH
GSE40272	X20202	10057	ABCC5
GSE40272	X21453	56945	MRPS22
GSE40272	X38282	54955	C1orf109
GSE40272	X3368	51626	DYNC2LI1
GSE40272	X33000	440248	HERC2P9
GSE40272	X25773	26273	FBXO3
GSE40272	X41243	84216	TMEM117
GSE40272	X16361	11342	RNF13
GSE40272	X22399	123606	NIPA1
GSE40272	X43002	254887	ZDHHC23
GSE40272	X12984	5934	RBL2
GSE40272	X33080	54916	C14orf101
GSE40272	X12212	10989	IMMT

GSE40272	X36509	59342	SCPEP1
GSE40272	X3903	7342	UBP1
GSE40272	X31374	51003	MED31
GSE40272	X41485	55454	CSGALNACT2
GSE40272	X18378	84617	TUBB6
GSE40272	X31174	10487	CAP1
GSE40272	X22225	22920	KIFAP3
GSE40272	X39597	4677	NARS
GSE40272	X35436	1601	DAB2
GSE40272	X4621	515	ATP5F1
GSE40272	X27082	8195	MKKS
GSE40272	X23186	9532	BAG2
GSE40272	X17645	64965	MRPS9
GSE40272	X25204	60496	AASDHPPT
GSE40272	X32914	8509	NDST2
GSE40272	X5882	23379	KIAA0947
GSE40272	X1901	286044	LOC286044
GSE40272	X35665	11328	FKBP9
GSE40272	X29167	567	B2M
GSE40272	X29096	10010	TANK
GSE40272	X36681	8976	WASL
GSE40272	X32137	6611	SMS
GSE40272	X33393	57579	FAM135A
GSE40272	X33781	51649	MRPS23
GSE40272	X35910	481	ATP1B1
GSE40272	X10917	11120	BTN2A1
GSE40272	X36871	51175	TUBE1
GSE40272	X43001	23291	FBXW11
GSE40272	X35385	8669	EIF3J
GSE40272	X3755	23512	SUZ12
GSE40272	X35102	137886	UBXN2B
GSE40272	X23652	7319	UBE2A
GSE40272	X40545	3845	KRAS
GSE40272	X4298	3376	IARS
GSE40272	X11616	23141	ANKLE2
GSE40272	X44330	8613	PPAP2B
GSE40272	X42912	64782	AEN
GSE40272	X16451	2058	EPRS
GSE40272	X16016	56647	BCCIP
GSE40272	X36440	4817	NIT1
GSE40272	X36646	9252	RPS6KA5
GSE40272	X20780	55625	ZDHHC7
GSE40272	X29916	27122	DKK3
GSE40272	X41551	64116	SLC39A8
GSE40272	X2580	81533	ITFG1
GSE40272	X10684	10253	SPRY2
GSE40272	X28881	147007	TMEM199
GSE40272	X16225	4179	CD46
GSE40272	X29335	84946	LTV1
GSE40272	X44171	91050	CCDC149
GSE40272	X17384	55709	KBTBD4
GSE40272	X9078	7884	SLBP
GSE40272	X38774	8813	DPM1

GSE40272	X2753	84661	DPY30
GSE40272	X26933	26589	MRPL46
GSE40272	X33712	25936	NSL1
GSE40272	X9255	6399	TRAPPC2
GSE40272	X37228	55783	FTSJD1
GSE40272	X40097	29107	NXT1
GSE40272	X7015	9315	C5orf13
GSE40272	X28966	10682	EBP
GSE40272	X7641	10247	HRSP12
GSE40272	X2797	64065	PERP
GSE40272	X42716	51729	WBP11
GSE40272	X12852	23636	NUP62
GSE40272	X25344	128486	FITM2
GSE40272	X21748	439965	FAM35B2
GSE40272	X23440	57003	CCDC47
GSE40272	X4617	7150	TOP1
GSE40272	X15807	51729	WBP11
GSE40272	X21579	63876	PKNOX2
GSE40272	X43976	64601	VPS16
GSE40272	X29162	567	B2M
GSE40272	X21269	51569	UFM1
GSE40272	X32968	55177	FAM82A2
GSE40272	X18604	25938	HEATR5A
GSE40272	X36239	55257	C20orf20
GSE40272	X40219	378	ARF4
GSE40272	X30667	222521	C6orf224
GSE40272	X20079	515	ATP5F1
GSE40272	X34795	51501	C11orf73
GSE40272	X37079	10981	RAB32
GSE40272	X38018	119032	C10orf32
GSE40272	X40316	55854	ZC3H15
GSE40272	X42814	55596	ZCCHC8
GSE40272	X42070	8935	SKAP2
GSE40272	X41348	83940	TATDN1
GSE40272	X28329	960	CD44
GSE40272	X28123	4708	NDUFB2
GSE40272	X37400	1185	CLCN6
GSE40272	X34816	2034	EPAS1
GSE40272	X14566	11261	CHP
GSE40272	X13602	5527	PPP2R5C
GSE40272	X32227	154865	IQUB
GSE40272	X32999	4565	MT-TI
GSE40272	X39211	839	CASP6
GSE40272	X13877	7126	TNFAIP1
GSE40272	X42362	9869	SETDB1
GSE40272	X29508	51444	RNF138
GSE40272	X33210	2926	GRSF1
GSE40272	X39819	10436	EMG1
GSE40272	X30907	55625	ZDHHC7
GSE40272	X28450	55752	SEPT11
GSE40272	X7920	81558	FAM117A
GSE40272	X44361	84447	SYVN1
GSE40272	X23306	27316	RBMX

GSE40272	X16894	39	ACAT2
GSE40272	X35398	84319	C3orf26
GSE40272	X4593	2961	GTF2E2
GSE40272	X17972	50999	TMED5
GSE40272	X3575	54733	SLC35F2
GSE40272	X6843	665	BNIP3L
GSE40272	X24332	6637	SNRPG
GSE40272	X30963	571	BACH1
GSE40272	X26751	285622	NBPF22P
GSE40272	X12497	10542	HBXIP
GSE40272	X15087	57667	KIAA1546
GSE40272	X22921	84908	FAM136A
GSE40272	X40494	201965	RWDD4
GSE40272	X28152	51606	ATP6V1H
GSE40272	X39210	4092	SMAD7
GSE40272	X32018	88523	LOC88523
GSE40272	X41555	5805	PTS
GSE40272	X37845	143884	CWF19L2
GSE40272	X9621	85457	KIAA1737
GSE40272	X42625	54101	RIPK4
GSE40272	X43415	152559	PAQR3
GSE40272	X22320	80349	WDR61
GSE40272	X16015	55002	TMCO3
GSE40272	X18239	51754	TMEM8B
GSE40272	X28908	57498	KIDINS220
GSE40272	X42757	85364	ZCCHC3
GSE40272	X10327	114876	OSBPL1A
GSE40272	X32894	9557	CHD1L
GSE40272	X1205	5621	PRNP
GSE40272	X40950	10810	WASF3
GSE40272	X27698	10549	PRDX4
GSE40272	X30962	54800	KLHL24
GSE40272	X38207	10286	BCAS2
GSE40272	X18366	9588	PRDX6
GSE40272	X13897	7307	U2AF1
GSE40272	X16452	22900	CARD8
GSE40272	X31076	7465	WEE1
GSE40272	X3411	9861	PSMD6
GSE40272	X29307	567	B2M
GSE40272	X40337	84311	MRPL45
GSE40272	X35865	83700	JAM3
GSE40272	X7011	440567	UQCRHL
GSE40272	X16561	9553	MRPL33
GSE40272	X32637	51335	NGRN
GSE40272	X5045	4564	MT-TH
GSE40272	X36388	57245	FLJ20209
GSE40272	X40307	3150	HMGN1
GSE40272	X17271	10153	CEBPZ
GSE40272	X16179	283768	GOLGA8G
GSE40272	X16084	8777	MPDZ
GSE40272	X5091	64431	ACTR6
GSE40272	X44112	57560	IFT80
GSE40272	X31238	27020	NPTN

GSE40272	X25342	79726	WDR59
GSE40272	X3454	8653	DDX3Y
GSE40272	X24099	221178	SPATA13
GSE40272	X30511	11157	LSM6
GSE40272	X19157	5048	PAFAH1B1
GSE40272	X12716	140468	COX11P1
GSE40272	X22873	10150	MBNL2
GSE40272	X11371	10048	RANBP9
GSE40272	X25703	55251	PCMTD2
GSE40272	X40824	5311	PKD2
GSE40272	X28448	56984	PSMG2
GSE40272	X39800	51192	CKLF
GSE40272	X23655	27309	ZNF330
GSE40272	X40241	203547	VMA21
GSE40272	X40802	84146	ZNF644
GSE40272	X38217	79073	TMEM109
GSE40272	X39093	55204	GOLPH3L
GSE40272	X4271	57494	RIMKLB
GSE40272	X39551	6905	TBCE
GSE40272	X25769	836	CASP3
GSE40272	X31121	4724	NDUFS4
GSE40272	X10790	9589	WTAP
GSE40272	X25776	51300	TIMMDC1
GSE40272	X29182	9520	NPEPPS
GSE40272	X25507	55032	SLC35A5
GSE40272	X36502	3491	CYR61
GSE40272	X16337	9020	MAP3K14
GSE40272	X7284	1478	CSTF2
GSE40272	X11723	10447	FAM3C
GSE40272	X8015	84833	USMG5
GSE40272	X21938	84932	RAB2B
GSE40272	X8743	10342	TFG
GSE40272	X43601	7913	DEK
GSE40272	X1254	2005	ELK4
GSE40272	X22683	83941	TM2D1
GSE40272	X30131	27235	COQ2
GSE40272	X28142	5716	PSMD10
GSE40272	X40303	567	B2M
GSE40272	X1816	1739	DLG1
GSE40272	X25608	29058	C20orf30
GSE40272	X18869	9693	RAPGEF2
GSE40272	X10922	84317	CCDC115
GSE40272	X5406	123803	NTAN1
GSE40272	X9472	56995	TULP4
GSE40272	X18249	8767	RIPK2
GSE40272	X38412	29968	PSAT1
GSE40272	X21496	64216	TFB2M
GSE40272	X13671	549	AUH
GSE40272	X15567	599	BCL2L2
GSE40272	X11114	6189	RPS3A
GSE40272	X901	4289	MKLN1
GSE40272	X26290	18	ABAT
GSE40272	X28535	8087	FXR1

GSE40272	X8023	6678	SPARC
GSE40272	X16745	90693	CCDC126
GSE40272	X40417	26521	TIMM8B
GSE40272	X44230	202018	TAPT1
GSE40272	X25273	79868	ALG13
GSE40272	X7889	10920	COPS8
GSE40272	X17217	1353	COX11
GSE40272	X18199	8443	GNPAT
GSE40272	X28177	23678	SGK3
GSE40272	X41448	57037	ANKMY2
GSE40272	X19890	5504	PPP1R2
GSE40272	X19056	81609	SNX27
GSE40272	X36846	220793	FUNDC2P1
GSE40272	X22790	5901	RAN
GSE40272	X20368	51603	METTL13
GSE40272	X41771	51218	GLRX5
GSE40272	X1903	259282	BOD1L
GSE40272	X15578	4670	HNRNPM
GSE40272	X34308	27248	ERLEC1
GSE40272	X39568	4568	MT-L2
GSE40272	X42798	81037	CLPTM1L
GSE40272	X13586	9639	ARHGEF10
GSE40272	X39317	8850	KAT2B
GSE40272	X35941	29970	SCHIP1
GSE40272	X15905	26056	RAB11FIP5
GSE40272	X31229	389203	C4orf52
GSE40272	X1645	339287	MSL1
GSE40272	X9250	94121	SYTL4
GSE40272	X26091	5393	EXOSC9
GSE40272	X33413	6908	TBP
GSE40272	X32483	567	B2M
GSE40272	X20273	9589	WTAP
GSE40272	X6263	7419	VDAC3
GSE40272	X17866	285148	IAH1
GSE40272	X37827	9854	C2CD2L
GSE40272	X12538	6605	SMARCE1
GSE40272	X41596	57092	PCNP
GSE40272	X15329	8930	MBD4
GSE40272	X32954	54625	PARP14
GSE40272	X44224	5515	PPP2CA
GSE40272	X6159	54537	FAM35A
GSE40272	X25712	2186	BPTF
GSE40272	X33133	665	BNIP3L
GSE40272	X30210	81689	ISCA1
GSE40272	X32842	23189	KANK1
GSE40272	X43960	159090	FAM122B
GSE40272	X25925	139341	FUNDC1
GSE40272	X35611	23411	SIRT1
GSE40272	X7209	81606	LBH
GSE40272	X39896	56930	LOC56930
GSE40272	X36591	567	B2M
GSE40272	X26328	27154	BRPF3
GSE40272	X43381	123803	NTAN1

GSE40272	X30001	144348	ZNF664
GSE40272	X33438	3320	HSP90AA1
GSE40272	X37923	10049	DNAJB6
GSE40272	X14311	80145	THOC7
GSE40272	X36683	163126	EID2
GSE40272	X1973	51201	ZDHHC2
GSE40272	X4756	196446	C12orf28
GSE40272	X10546	219854	TMEM218
GSE40272	X1474	115426	UHRF2
GSE40272	X6060	23133	PHF8
GSE40272	X31787	83604	TMEM47
GSE40272	X23926	80311	KLHL15
GSE40272	X32069	29081	METTL5
GSE40272	X39629	6227	RPS21
GSE40272	X39200	64778	FNDC3B
GSE40272	X42065	84640	USP38
GSE40272	X32704	55323	LARP6
GSE40272	X5804	84312	BRMS1L
GSE40272	X22876	25850	ZNF345
GSE40272	X35607	1955	MEGF9
GSE40272	X33231	55967	NDUFA12
GSE40272	X20867	57045	TWSG1
GSE40272	X44092	10973	ASCC3
GSE40272	X39375	567	B2M
GSE40272	X31951	567	B2M
GSE40272	X14827	10063	COX17
GSE40272	X36675	114790	STK11IP
GSE40272	X42642	28998	MRPL13
GSE40272	X5521	25923	ATL3
GSE40272	X39699	7026	NR2F2
GSE40272	X30525	5889	RAD51C
GSE40272	X19469	283768	GOLGA8G
GSE40272	X36582	54505	DHX29
GSE40272	X36665	51765	MST4
GSE40272	X22060	1371	CPOX
GSE40272	X37109	23067	SETD1B
GSE40272	X42074	284058	KIAA1267
GSE40272	X36234	8812	CCNK
GSE40272	X24840	63933	CCDC90A
GSE40272	X36543	136319	MTPN
GSE40272	X11076	6883	TAF12
GSE40272	X10330	80010	RMI1
GSE40272	X21130	92140	MTDH
GSE40272	X38085	9324	HMGN3
GSE40272	X29481	83695	C12orf32
GSE40272	X40341	329	BIRC2
GSE40272	X25468	55717	WDR11
GSE40272	X14901	4122	MAN2A2
GSE40272	X21021	29093	MRPL22
GSE40272	X18052	23511	NUP188
GSE40272	X29133	5683	PSMA2
GSE40272	X27130	9861	PSMD6
GSE40272	X41434	114135	LOC114135

GSE40272	X15041	4528	MTIF2
GSE40272	X35649	1635	DCTD
GSE40272	X35242	1112	FOXN3
GSE40272	X40631	55970	GNG12
GSE40272	X38800	80298	MTERFD3
GSE40272	X25047	54700	RRN3
GSE40272	X37641	390948	LOC390948
GSE40272	X29715	11282	MGAT4B
GSE40272	X28360	5464	PPA1
GSE40272	X7985	10969	EBNA1BP2
GSE40272	X24745	10116	FEM1B
GSE40272	X39381	10772	SRSF10
GSE40272	X25042	79837	PIP4K2C
GSE40272	X39173	128989	C22orf25
GSE40272	X25722	30851	TAX1BP3
GSE40272	X43923	283489	ZNF828
GSE40272	X33818	53371	NUP54
GSE40272	X42254	55218	EXD2
GSE40272	X6177	51639	SF3B14
GSE40272	X15082	57162	PELI1
GSE40272	X20082	114971	PTPMT1
GSE40272	X28330	1027	CDKN1B
GSE40272	X15038	51292	GMPR2
GSE40272	X28628	205428	C3orf58
GSE40272	X6191	7417	VDAC2
GSE40272	X43585	8675	STX16
GSE40272	X23880	60592	SCOC
GSE40272	X25660	1432	MAPK14
GSE40272	X10140	26063	DECR2
GSE40272	X13338	151445	LOC151445
GSE40272	X17826	3251	HPRT1
GSE40272	X37358	29978	UBQLN2
GSE40272	X33841	130916	MTERFD2
GSE40272	X21713	64215	DNAJC1
GSE40272	X40053	10449	ACAA2
GSE40272	X30965	9637	FEZ2
GSE40272	X36024	5111	PCNA
GSE40272	X15893	7411	VBP1
GSE40272	X40190	79863	RBFA
GSE40272	X26038	51660	BRP44L
GSE40272	X38436	4052	LTBP1
GSE40272	X40385	58486	ZBED5
GSE40272	X27150	26053	AUTS2
GSE40272	X8663	55120	FANCL
GSE40272	X40432	10092	ARPC5
GSE40272	X38624	8453	CUL2
GSE40272	X31111	6477	SIAH1
GSE40272	X14029	5682	PSMA1
GSE40272	X19351	79139	DERL1
GSE40272	X38568	55253	TYW1
GSE40272	X25970	64219	PJA1
GSE40272	X41054	51199	NIN
GSE40272	X38497	444	ASPH

GSE40272	X36909	26994	RNF11
GSE40272	X3264	8943	AP3D1
GSE40272	X26460	25934	NIPSNAP3A
GSE40272	X13130	401029	LOC401029
GSE40272	X33470	26273	FBXO3
GSE40272	X22466	51735	RAPGEF6
GSE40272	X39018	9821	RB1CC1
GSE40272	X22856	9553	MRPL33
GSE40272	X9555	291	SLC25A4
GSE40272	X16927	55915	LANCL2
GSE40272	X14913	1675	CFD
GSE40272	X11810	10927	SPIN1
GSE40272	X24737	5332	PLCB4
GSE40272	X14836	10632	ATP5L
GSE40272	X43758	8835	SOCS2
GSE40272	X3577	56647	BCCIP
GSE40272	X30930	988	CDC5L
GSE40272	X24835	5827	PXMP2
GSE40272	X40453	9100	USP10
GSE40272	X42275	51258	MRPL51
GSE40272	X30597	4664	NAB1
GSE40272	X17577	1399	CRKL
GSE40272	X14988	57179	KIAA1191
GSE40272	X32411	440026	TMEM41B
GSE40272	X9319	1783	DYNC1LI2
GSE40272	X16251	158358	KIAA2026
GSE40272	X21891	388962	BOLA3
GSE40272	X12615	664	BNIP3
GSE40272	X19763	374969	CCDC23
GSE40272	X16210	114224	PRO2852
GSE40272	X38072	54872	PIGG
GSE40272	X16641	51719	CAB39
GSE40272	X38662	27336	HTATSF1
GSE40272	X21781	2990	GUSB
GSE40272	X44089	528	ATP6V1C1
GSE40272	X27500	439983	CTGLF2
GSE40272	X30293	51026	GOLT1B
GSE40272	X25471	2	A2M
GSE40272	X40343	55795	PCID2
GSE40272	X24347	158427	TSTD2
GSE40272	X8996	6392	SDHD
GSE40272	X42435	2274	FHL2
GSE40272	X28568	23168	RTF1
GSE40272	X23852	6297	SALL2
GSE40272	X22869	5062	PAK2
GSE40272	X40091	145567	TTC7B
GSE40272	X17115	57209	ZNF248
GSE40272	X5907	515	ATP5F1
GSE40272	X3238	90411	MCFD2
GSE40272	X34922	7170	TPM3
GSE40272	X32265	90488	C12orf23
GSE40272	X27368	28957	MRPS28
GSE40272	X29555	137695	TMEM68

GSE40272	X21709	54927	CHCHD3
GSE40272	X26457	800	CALD1
GSE40272	X43591	5828	PEX2
GSE40272	X36908	140461	ASB8
GSE40272	X33360	3475	IFRD1
GSE40272	X19457	1051	CEBPB
GSE40272	X38994	4694	NDUFA1
GSE40272	X14263	144438	LOC144438
GSE40272	X2574	29116	MYLIP
GSE40272	X11686	10971	YWHAQ
GSE40272	X19771	55075	UACA
GSE40272	X25033	10092	ARPC5
GSE40272	X37735	55508	SLC35E3
GSE40272	X27408	7763	ZFAND5
GSE40272	X10199	5431	POLR2B
GSE40272	X27219	80315	CPEB4
GSE40272	X38335	23258	DENND5A
GSE40272	X1832	65982	ZSCAN18
GSE40272	X12557	51633	OTUD6B
GSE40272	X21494	171546	SPTSSA
GSE40272	X35811	10434	LYPLA1
GSE40272	X32879	567	B2M
GSE40272	X27704	6845	VAMP7
GSE40272	X31206	8674	VAMP4
GSE40272	X17559	22	ABCB7
GSE40272	X4417	4190	MDH1
GSE40272	X32070	81689	ISCA1
GSE40272	X26699	23256	SCFD1
GSE40272	X6682	81566	CSRNP2
GSE40272	X16837	8050	PDHX
GSE40272	X25536	9709	HERPUD1
GSE40272	X13211	155061	ZNF746
GSE40272	X21301	7286	TUFT1
GSE40272	X7146	54726	OTUD4
GSE40272	X22745	79901	CYBRD1
GSE40272	X42512	80853	JHDM1D
GSE40272	X40573	85369	FAM40A
GSE40272	X31366	26258	PLDN
GSE40272	X5313	9223	MAGI1
GSE40272	X23562	4698	NDUFA5
GSE40272	X36730	567	B2M
GSE40272	X19035	23014	FBXO21
GSE40272	X9389	7073	TIAL1
GSE40272	X43434	9403	SEP15
GSE40272	X33719	84078	KBTBD7
GSE40272	X40557	153396	TMEM161B
GSE40272	X38285	253769	WDR27
GSE40272	X30146	54617	INO80
GSE40272	X24742	23158	TBC1D9
GSE40272	X19857	84987	C12orf62
GSE40272	X7104	545	ATR
GSE40272	X36628	1831	TSC22D3
GSE40272	X20249	1347	COX7A2

GSE40272	X6055	55727	BTBD7
GSE40272	X17320	4780	NFE2L2
GSE40272	X7219	23621	BACE1
GSE40272	X23623	10049	DNAJB6
GSE40272	X42674	8543	LMO4
GSE40272	X1486	3148	HMGB2
GSE40272	X13113	84572	GNPTG
GSE40272	X34580	8727	CTNNAL1
GSE40272	X30040	27244	SESN1
GSE40272	X24098	9559	VPS26A
GSE40272	X41169	3843	IPO5
GSE40272	X20290	6880	TAF9
GSE40272	X44270	4522	MTHFD1
GSE40272	X39491	2339	FNTA
GSE40272	X4053	10808	HSPH1
GSE40272	X30311	55312	RFK
GSE40272	X13895	56983	POGLUT1
GSE40272	X23433	4750	NEK1
GSE40272	X11929	79612	NAA16
GSE40272	X19938	4891	SLC11A2
GSE40272	X18321	151903	CCDC12
GSE40272	X30940	5376	PMP22
GSE40272	X14588	54942	FAM206A
GSE40272	X9891	84919	PPP1R15B
GSE40272	X11248	3032	HADHB
GSE40272	X4479	286472	RAC1P4
GSE40272	X9520	51031	GLOD4
GSE40272	X42033	1655	DDX5
GSE40272	X32755	26985	AP3M1
GSE40272	X12979	130355	C2orf76
GSE40272	X4762	11044	PAPD7
GSE40272	X40612	55744	C7orf44
GSE40272	X39314	27430	MAT2B
GSE40272	X29244	10730	YME1L1
GSE40272	X34422	3336	HSPE1
GSE40272	X31667	10190	TXNDC9
GSE40272	X14296	6281	S100A10
GSE40272	X14509	200030	NBPF11
GSE40272	X11067	9354	UBE4A
GSE40272	X5609	9039	UBA3
GSE40272	X14291	10664	CTCF
GSE40272	X38567	908	CCT6A
GSE40272	X17020	4047	LSS
GSE40272	X22107	57092	PCNP
GSE40272	X31293	11142	PKIG
GSE40272	X35470	6100	RP9
GSE40272	X17101	567	B2M
GSE40272	X8483	5214	PFKP
GSE40272	X23931	6773	STAT2
GSE40272	X37607	79717	PPCS
GSE40272	X31410	10560	SLC19A2
GSE40272	X23327	10099	TSPAN3
GSE40272	X18108	1039	CDR2

GSE40272	X3373	5425	POLD2
GSE40272	X41345	440709	LOC440709
GSE40272	X13041	64745	METTL17
GSE40272	X6836	4615	MYD88
GSE40272	X22848	9793	CKAP5
GSE40272	X3291	11168	PSIP1
GSE40272	X15755	7307	U2AF1
GSE40272	X38772	22908	SACM1L
GSE40272	X35381	55149	MTPAP
GSE40272	X43733	3981	LIG4
GSE40272	X36469	4905	NSF
GSE40272	X15245	567	B2M
GSE40272	X31023	567	B2M
GSE40272	X36839	56160	NDNL2
GSE40272	X35981	29079	MED4
GSE40272	X42167	202459	OSTCL
GSE40272	X1523	23484	LEPROTL1
GSE40272	X30095	567	B2M
GSE40272	X17456	6856	SYPL1
GSE40272	X26446	29097	CNIH4
GSE40272	X20906	10175	CNIH
GSE40272	X31316	440066	LOC440066
GSE40272	X33365	5861	RAB1A
GSE40272	X21397	871	SERPINH1
GSE40272	X27639	3685	ITGAV
GSE40272	X17124	283232	TMEM80
GSE40272	X24841	8895	CPNE3
GSE40272	X3855	128	ADH5
GSE40272	X23691	25853	DCAF12
GSE40272	X17009	3655	ITGA6
GSE40272	X23061	50804	MYEF2
GSE40272	X24363	8204	NRIP1
GSE40272	X20443	392487	LOC392487
GSE40272	X43039	51258	MRPL51
GSE40272	X20634	8678	BECN1
GSE40272	X37206	128	ADH5
GSE40272	X24608	133619	PRRC1
GSE40272	X3549	830	CAPZA2
GSE40272	X23568	57570	TRMT5
GSE40272	X31059	23760	PITPNB
GSE40272	X13606	440026	TMEM41B
GSE40272	X31776	64089	SNX16
GSE40272	X4609	10180	RBM6
GSE40272	X28722	2782	GNB1
GSE40272	X2905	7257	TSNAX
GSE40272	X31172	65991	FUNDC2
GSE40272	X16676	23067	SETD1B
GSE40272	X6624	79886	C9orf82
GSE40272	X42184	11171	STRAP
GSE40272	X12134	11258	DCTN3
GSE40272	X38833	9943	OXSR1
GSE40272	X38957	22950	SLC4A1AP
GSE40272	X36785	83930	STARD3NL

GSE40272	X42813	55030	FBXO34
GSE40272	X10166	79188	TMEM43
GSE40272	X4614	51015	ISOC1
GSE40272	X3552	58515	SELK
GSE40272	X31060	25959	KANK2
GSE40272	X24480	64769	MEAF6
GSE40272	X14519	54495	TMX3
GSE40272	X22005	127687	C1orf122
GSE40272	X33284	58493	C9orf80
GSE40272	X14840	51307	FAM53C
GSE40272	X37245	64860	ARMCX5
GSE40272	X37459	6744	SSFA2
GSE40272	X20266	51256	TBC1D7
GSE40272	X2990	57492	ARID1B
GSE40272	X34832	79145	CHCHD7
GSE40272	X42068	9562	MINPP1
GSE40272	X1830	3949	LDLR
GSE40272	X26704	6787	NEK4
GSE40272	X37613	55857	PLK1S1
GSE40272	X28517	29929	ALG6
GSE40272	X22015	150275	CCDC117
GSE40272	X26270	10671	DCTN6
GSE40272	X33397	23045	FRYL
GSE40272	X35455	56683	C21orf59
GSE40272	X22824	25926	NOL11
GSE40272	X15261	145553	MDP1
GSE40272	X7100	130814	PQLC3
GSE40272	X31139	84084	RAB6C
GSE40272	X10548	4952	OCRL
GSE40272	X39042	10432	RBM14
GSE40272	X3268	115416	C7orf30
GSE40272	X21195	83891	SNX25
GSE40272	X29504	29880	ALG5
GSE40272	X7943	3150	HMGN1
GSE40272	X41417	10933	MORF4L1
GSE40272	X2092	84248	FYTTD1
GSE40272	X39726	440702	LOC440702
GSE40272	X43819	51142	CHCHD2
GSE40272	X15804	116829	N4BP2L2-IT1
GSE40272	X17300	22828	SCAF8
GSE40272	X38593	10376	TUBA1B
GSE40272	X26869	91754	NEK9
GSE40272	X3517	8892	EIF2B2
GSE40272	X10875	5236	PGM1
GSE40272	X19510	283377	SPRYD4
GSE40272	X25816	22948	CCT5
GSE40272	X30176	23515	MORC3
GSE40272	X38284	9819	TSC22D2
GSE40272	X975	6788	STK3
GSE40272	X24851	5500	PPP1CB
GSE40272	X11000	55193	PBRM1
GSE40272	X11851	23201	FAM168A
GSE40272	X40245	84128	WDR75

GSE40272	X27352	6772	STAT1
GSE40272	X34610	2040	STOM
GSE40272	X27635	5532	PPP3CB
GSE40272	X9260	4697	NDUFA4
GSE40272	X11687	7388	UQCRH
GSE40272	X36872	54529	ASNSD1
GSE40272	X40649	8440	NCK2
GSE40272	X9020	6840	SVIL
GSE40272	X38447	567	B2M
GSE40272	X38694	1660	DHX9
GSE40272	X39980	23016	EXOSC7
GSE40272	X35581	7105	TSPAN6
GSE40272	X43653	123169	LEO1
GSE40272	X9266	847	CAT
GSE40272	X9894	22928	SEPHS2
GSE40272	X35824	54148	MRPL39
GSE40272	X35683	27018	NGFRAP1
GSE40272	X39393	10559	SLC35A1
GSE40272	X22216	3490	IGFBP7
GSE40272	X32044	23514	KIAA0146
GSE40272	X30414	4292	MLH1
GSE40272	X32871	81542	TMX1
GSE40272	X35849	6902	TBCA
GSE40272	X22158	4236	MFAP1
GSE40272	X32828	51239	ANKRD39
GSE40272	X19997	54780	NSMCE4A
GSE40272	X14627	5162	PDHB
GSE40272	X27320	64940	STAG3L4
GSE40272	X13052	51626	DYNC2LI1
GSE40272	X38003	55610	CCDC132
GSE40272	X11432	91694	LONRF1
GSE40272	X8738	22909	FAN1
GSE40272	X34174	9530	BAG4
GSE40272	X19474	9525	VPS4B
GSE40272	X28293	9962	SLC23A2
GSE40272	X29219	55056	FLJ10038
GSE40272	X24606	4718	NDUFC2
GSE40272	X12514	47	ACLY
GSE40272	X13557	51188	SS18L2
GSE40272	X36866	84259	DCUN1D5
GSE40272	X14121	546	ATRX
GSE40272	X34888	26521	TIMM8B
GSE40272	X10726	7388	UQCRH
GSE40272	X5593	6731	SRP72
GSE40272	X34570	54861	SNRK
GSE40272	X23526	10558	SPTLC1
GSE40272	X29485	2631	GBAS
GSE40272	X29418	5604	MAP2K1
GSE40272	X13355	51650	MRPS33
GSE40272	X39467	23048	FNBP1
GSE40272	X22680	388962	BOLA3
GSE40272	X30964	5911	RAP2A
GSE40272	X33036	9842	PLEKHM1

GSE40272	X42473	9913	SUPT7L
GSE40272	X27589	372	ARCN1
GSE40272	X10612	64853	AIDA
GSE40272	X19596	4628	MYH10
GSE40272	X35782	81892	SLIRP
GSE40272	X19535	7048	TGFBR2
GSE40272	X39514	60	ACTB
GSE40272	X33225	84365	MKI67IP
GSE40272	X26178	51759	C9orf78
GSE40272	X33030	54543	TOMM7
GSE40272	X28031	56650	CLDND1
GSE40272	X39764	219654	ZCCHC24
GSE40272	X3002	81561	C2orf31
GSE40272	X40252	514	ATP5E
GSE40272	X13395	5223	PGAM1
GSE40272	X37553	131544	CRYBG3
GSE40272	X44344	79876	UBA5
GSE40272	X16081	7078	TIMP3
GSE40272	X8034	6643	SNX2
GSE40272	X3338	54948	MRPL16
GSE40272	X10342	7205	TRIP6
GSE40272	X43176	6059	ABCE1
GSE40272	X24623	5824	PEX19
GSE40272	X24731	149371	EXOC8
GSE40272	X11486	51637	C14orf166
GSE40272	X26928	3182	HNRNPAB
GSE40272	X24531	65220	NADK
GSE40272	X36224	27258	LSM3
GSE40272	X13916	60	ACTB
GSE40272	X35346	10286	BCAS2
GSE40272	X27068	9968	MED12
GSE40272	X5010	8883	NAE1
GSE40272	X17676	1477	CSTF1
GSE40272	X24082	285362	SUMF1
GSE40272	X16494	36	ACADSB
GSE40272	X24646	5870	RAB6A
GSE40272	X26324	79612	NAA16
GSE40272	X341	10651	MTX2
GSE40272	X39342	4502	MT2A
GSE40272	X27383	84991	RBM17
GSE40272	X35525	26263	FBXO22
GSE40272	X38146	8883	NAE1
GSE40272	X7965	7431	VIM
GSE40272	X24176	64397	ZFP106
GSE40272	X39470	51029	PPPDE1
GSE40272	X11269	961	CD47
GSE40272	X36511	9765	ZFYVE16
GSE40272	X40421	5756	TWF1
GSE40272	X8221	9252	RPS6KA5
GSE40272	X18397	253832	ZDHHC20
GSE40272	X30596	27247	NFU1
GSE40272	X34582	3263	HPX
GSE40272	X16912	441531	PGAM4

GSE40272	X41742	9126	SMC3
GSE40272	X43141	10044	SH2D3C
GSE40272	X7241	81552	VOPP1
GSE40272	X26480	83452	RAB33B
GSE40272	X41266	145815	MGC18216
GSE40272	X32929	286170	LOC286170
GSE40272	X3096	23076	RRP1B
GSE40272	X14317	567	B2M
GSE40272	X39893	27236	ARFIP1
GSE40272	X7336	60	ACTB
GSE40272	X24442	24145	PANX1
GSE40272	X13001	11334	TUSC2
GSE40272	X2203	54534	MRPL50
GSE40272	X21780	175	AGA
GSE40272	X34143	54662	TBC1D13
GSE40272	X16400	85365	ALG2
GSE40272	X39590	23350	U2SURP
GSE40272	X25770	85021	REPS1
GSE40272	X18675	23548	TTC33
GSE40272	X19965	5412	UBL3
GSE40272	X35675	57496	MKL2
GSE40272	X33625	10725	NFAT5
GSE40272	X42626	55334	SLC39A9
GSE40272	X38286	26092	TOR1AIP1
GSE40272	X7835	81892	SLIRP
GSE40272	X24943	23787	MTCH1
GSE40272	X29424	389599	STRADBP1
GSE40272	X18532	1385	CREB1
GSE40272	X20341	64963	MRPS11
GSE40272	X39842	5052	PRDX1
GSE40272	X34938	96764	TGS1
GSE40272	X36738	10477	UBE2E3
GSE40272	X16143	51510	CHMP5
GSE40272	X43078	56943	ENY2
GSE40272	X16592	6500	SKP1
GSE40272	X40514	51390	AIG1
GSE40272	X4600	22934	RPIA
GSE40272	X38185	6742	SSBP1
GSE40272	X30284	201965	RWDD4
GSE40272	X11726	360132	FKBP9L
GSE40272	X31057	10767	HBS1L
GSE40272	X22302	11157	LSM6
GSE40272	X42710	23588	KLHDC2
GSE40272	X28426	4707	NDUFB1
GSE40272	X3359	55229	PANK4
GSE40272	X35397	81614	NIPA2
GSE40272	X15422	51517	NCKIPSD
GSE40272	X31370	10507	SEMA4D
GSE40272	X27319	63933	CCDC90A
GSE40272	X35062	8545	CGGBP1
GSE40272	X4017	10558	SPTLC1
GSE40272	X36425	2730	GCLM
GSE40272	X35012	23141	ANKLE2

GSE40272	X19214	8562	DENR
GSE40272	X29342	3293	HSD17B3
GSE40272	X41007	23479	ISCU
GSE40272	X43238	10376	TUBA1B
GSE40272	X26231	79680	C22orf29
GSE40272	X13587	171546	SPTSSA
GSE40272	X20782	22938	SNW1
GSE40272	X21150	119504	ANAPC16
GSE40272	X29309	6185	RPN2
GSE40272	X9262	3312	HSPA8
GSE40272	X29575	5716	PSMD10
GSE40272	X20096	6342	SCP2
GSE40272	X34679	23140	ZZEF1
GSE40272	X13394	5937	RBMS1
GSE40272	X39490	84992	PIGY
GSE40272	X32488	567	B2M
GSE40272	X18072	56658	TRIM39
GSE40272	X42879	5683	PSMA2
GSE40272	X16390	26130	GAPVD1
GSE40272	X20910	4724	NDUFS4
GSE40272	X22882	6934	TCF7L2
GSE40272	X36915	9669	EIF5B
GSE40272	X42271	9877	ZC3H11A
GSE40272	X7384	11325	DDX42
GSE40272	X37519	567	B2M
GSE40272	X28295	2475	MTOR
GSE40272	X21511	6880	TAF9
GSE40272	X14899	6427	SRSF2
GSE40272	X31890	2729	GCLC
GSE40272	X24175	57583	TMEM181
GSE40272	X38235	23095	KIF1B
GSE40272	X24728	55841	WWC3
GSE40272	X37343	81565	NDEL1
GSE40272	X19727	5501	PPP1CC
GSE40272	X29173	6230	RPS25
GSE40272	X27406	54906	C10orf18
GSE40272	X29558	339290	LOC339290
GSE40272	X18707	29964	PRICKLE4
GSE40272	X25674	92906	HNRPLL
GSE40272	X31872	127018	LYPLAL1
GSE40272	X43918	5436	POLR2G
GSE40272	X3393	4141	MARS
GSE40272	X32127	64981	MRPL34
GSE40272	X38390	10457	GPNMB
GSE40272	X19682	403	ARL3
GSE40272	X22530	8874	ARHGEF7
GSE40272	X16338	9775	EIF4A3
GSE40272	X22753	91298	C12orf29
GSE40272	X31046	57002	C7orf36
GSE40272	X28520	55749	CCAR1
GSE40272	X16608	84191	FAM96A
GSE40272	X7383	10006	ABI1
GSE40272	X33332	8574	AKR7A2

GSE40272	X21330	79745	CLIP4
GSE40272	X43302	23358	USP24
GSE40272	X31314	5631	PRPS1
GSE40272	X1689	5781	PTPN11
GSE40272	X23858	150472	CBWD2
GSE40272	X5290	5908	RAP1B
GSE40272	X10995	128866	CHMP4B
GSE40272	X761	8655	DYNLL1
GSE40272	X4499	26275	HIBCH
GSE40272	X36467	27342	RABGEF1
GSE40272	X26639	5906	RAP1A
GSE40272	X14191	51626	DYNC2LI1
GSE40272	X40104	2959	GTF2B
GSE40272	X29680	57108	SETD8P1
GSE40272	X28035	60313	GPBP1L1
GSE40272	X27117	5687	PSMA6
GSE40272	X30250	8520	HAT1
GSE40272	X11249	55184	C20orf12
GSE40272	X11533	567	B2M
GSE40272	X6483	11215	AKAP11
GSE40272	X44153	567	B2M
GSE40272	X7858	134553	C5orf24
GSE40272	X25731	123811	FOPNL
GSE40272	X28041	54537	FAM35A
GSE40272	X11024	11345	GABARAPL2
GSE40272	X4193	8805	TRIM24
GSE40272	X32996	91272	BOD1
GSE40272	X26659	23478	SEC11A
GSE40272	X10028	2098	ESD
GSE40272	X36674	64776	C11orf1
GSE40272	X32986	79685	SAP30L
GSE40272	X33292	23326	USP22
GSE40272	X5548	441155	LOC441155
GSE40272	X37926	3688	ITGB1
GSE40272	X35669	908	CCT6A
GSE40272	X11476	7321	UBE2D1
GSE40272	X5203	26101	DKFZP564M1462
GSE40272	X32790	29994	BAZ2B
GSE40272	X36162	90462	ZNF605
GSE40272	X42848	8802	SUCLG1
GSE40272	X11999	93621	MRFAP1
GSE40272	X43305	10289	EIF1B
GSE40272	X18283	80227	PAAF1
GSE40272	X21795	54940	OCIAD1
GSE40272	X5247	57515	SERINC1
GSE40272	X38499	688	KLF5
GSE40272	X9001	8562	DENR
GSE40272	X34377	10483	SEC23B
GSE40272	X24469	8669	EIF3J
GSE40272	X36497	1793	DOCK1
GSE40272	X36352	56681	SAR1A
GSE40272	X15806	5464	PPA1
GSE40272	X17753	7508	XPC

GSE40272	X19152	51030	FAM18B1
GSE40272	X35068	79921	TCEAL4
GSE40272	X8065	64983	MRPL32
GSE40272	X12093	51714	SELT
GSE40272	X17313	5257	PHKB
GSE40272	X18115	10712	FAM189B
GSE40272	X22496	84265	POLR3GL
GSE40272	X11886	55017	C14orf119
GSE40272	X18187	8872	CDC123
GSE40272	X39171	6432	SRSF7
GSE40272	X15897	10210	TOPORS
GSE40272	X36197	567	B2M
GSE40272	X31284	51372	CCDC72
GSE40272	X34873	567	B2M
GSE40272	X6302	114884	OSBPL10
GSE40272	X5597	84817	TXNDC17
GSE40272	X19956	283680	LOC283680
GSE40272	X10178	8772	FADD
GSE40272	X37573	152503	SH3D19
GSE40272	X40873	117143	TADA1
GSE40272	X40138	29105	C16orf80
GSE40272	X7697	221154	EFHA1
GSE40272	X25196	5641	LGMN
GSE40272	X30910	93622	LOC93622
GSE40272	X39911	567	B2M
GSE40272	X44430	51012	SLMO2
GSE40272	X35309	3189	HNRNPH3
GSE40272	X38737	51501	C11orf73
GSE40272	X40444	567	B2M
GSE40272	X16642	54915	YTHDF1
GSE40272	X23208	567	B2M
GSE40272	X27450	567	B2M
GSE40272	X6088	10951	CBX1
GSE40272	X6560	1459	CSNK2A2
GSE40272	X17121	9978	RBX1
GSE40272	X41763	567	B2M
GSE40272	X18609	114882	OSBPL8
GSE40272	X36225	6827	SUPT4H1
GSE40272	X35410	908	CCT6A
GSE40272	X36472	84187	TMEM164
GSE40272	X33807	567	B2M
GSE40272	X40127	54910	SEMA4C
GSE40272	X8844	26269	FBXO8
GSE40272	X278	7336	UBE2V2
GSE40272	X25998	132	ADK
GSE40272	X8144	64398	MPP5
GSE40272	X18203	9326	ZNHIT3
GSE40272	X21070	51218	GLRX5
GSE40272	X25593	567	B2M
GSE40272	X26500	387805	LOC387805
GSE40272	X29683	9946	CRYZL1
GSE40272	X11522	25875	LETMD1
GSE40272	X40778	128240	APOA1BP

GSE40272	X27962	94101	ORMDL1
GSE40272	X35663	567	B2M
GSE40272	X21825	57511	COG6
GSE40272	X24769	84324	SARNP
GSE40272	X2769	6873	TAF2
GSE40272	X14672	5688	PSMA7
GSE40272	X21328	10952	SEC61B
GSE40272	X16118	10016	PDCD6
GSE40272	X7147	3032	HADHB
GSE40272	X42102	10463	SLC30A9
GSE40272	X36737	10539	GLRX3
GSE40272	X6893	567	B2M
GSE40272	X26489	64951	MRPS24
GSE40272	X31956	64979	MRPL36
GSE40272	X20206	3621	ING1
GSE40272	X29086	6429	SRSF4
GSE40272	X12789	55435	AP1AR
GSE40272	X21956	8289	ARID1A
GSE40272	X18948	5082	PDCL
GSE40272	X13681	28987	NOB1
GSE40272	X38487	65018	PINK1
GSE40272	X23496	3930	LBR
GSE40272	X27784	51193	ZNF639
GSE40272	X43734	51143	DYNC1LI1
GSE40272	X41784	55146	ZDHHC4
GSE40272	X23622	5684	PSMA3
GSE40272	X30183	79447	C16orf53
GSE40272	X30282	84061	MAGT1
GSE40272	X24403	3303	HSPA1A
GSE40272	X35481	85027	C5orf62
GSE40272	X20561	54545	MTMR12
GSE40272	X2980	55699	IARS2
GSE40272	X43167	121441	NEDD1
GSE40272	X5691	22978	NT5C2
GSE40272	X6370	4673	NAP1L1
GSE40272	X36427	64430	C14orf135
GSE40272	X16269	51646	YPEL5
GSE40272	X33883	23353	SUN1
GSE40272	X2762	6726	SRP9
GSE40272	X204	1349	COX7B
GSE40272	X3685	2799	GNS
GSE40272	X21109	10988	METAP2
GSE40272	X42742	23644	EDC4
GSE40272	X29047	8050	PDHX
GSE40272	X9772	23189	KANK1
GSE40272	X38670	55326	AGPAT5
GSE40272	X35715	5747	PTK2
GSE40272	X23137	56262	LRRC8A
GSE40272	X34074	51566	ARMCX3
GSE40272	X5765	11235	PDCD10
GSE40272	X7120	648	BMI1
GSE40272	X41901	5516	PPP2CB
GSE40272	X44272	3094	HINT1

GSE40272	X37867	285148	IAH1
GSE40272	X15498	1348	COX7A2P2
GSE40272	X13928	567	B2M
GSE40272	X20536	25814	ATXN10
GSE40272	X24463	84942	WDR73
GSE40272	X34296	23252	OTUD3
GSE40272	X21535	54902	TTC19
GSE40272	X15451	23499	MACF1
GSE40272	X32962	64708	COPS7B
GSE40272	X15403	153527	ZMAT2
GSE40272	X9236	51439	FAM8A1
GSE40272	X38038	4301	MLLT4
GSE40272	X42364	23047	PDS5B
GSE40272	X12654	55627	SMPD4
GSE40272	X13293	54994	C20orf11
GSE40272	X17194	10130	PDIA6
GSE40272	X37227	54386	TERF2IP
GSE40272	X32145	9337	CNOT8
GSE40272	X31836	51626	DYNC2LI1
GSE40272	X28358	7416	VDAC1
GSE40272	X38650	9815	GIT2
GSE40272	X40621	27230	SERP1
GSE40272	X39142	2958	GTF2A2
GSE40272	X2444	9789	SPCS2
GSE40272	X28494	79644	SRD5A3
GSE40272	X28592	6950	TCP1
GSE40272	X34523	23054	NCOA6
GSE40272	X28788	79837	PIP4K2C
GSE40272	X16313	567	B2M
GSE40272	X29131	11260	XPOT
GSE40272	X39122	9987	HNRPDL
GSE40272	X28274	9871	SEC24D
GSE40272	X21039	79811	SLTM
GSE40272	X4942	6513	SLC2A1
GSE40272	X12802	2060	EPS15
GSE40272	X5887	11179	ZNF277
GSE40272	X31273	84191	FAM96A
GSE40272	X30457	10487	CAP1
GSE40272	X14858	2752	GLUL
GSE40272	X7399	54467	ANKIB1
GSE40272	X25292	7705	ZNF146
GSE40272	X28774	567	B2M
GSE40272	X44133	6741	SSB
GSE40272	X37885	54469	ZFAND6
GSE40272	X8339	159	ADSS
GSE40272	X15440	483	ATP1B3
GSE40272	X18927	6631	SNRPC
GSE40272	X6549	84641	HIATL1
GSE40272	X25976	5160	PDHA1
GSE40272	X33802	567	B2M
GSE40272	X28882	23219	FBXO28
GSE40272	X20097	57001	ACN9
GSE40272	X22290	63893	UBE2O

GSE40272	X18416	58515	SELK
GSE40272	X27608	205327	C2orf69
GSE40272	X31287	5721	PSME2
GSE40272	X6806	26036	ZNF451
GSE40272	X12092	8270	LAGE3
GSE40272	X21995	998	CDC42
GSE40272	X19940	7259	TSPYL1
GSE40272	X35248	5687	PSMA6
GSE40272	X32458	51136	RNFT1
GSE40272	X41351	128	ADH5
GSE40272	X25391	5917	RARS
GSE40272	X35298	54888	NSUN2
GSE40272	X25724	55051	C14orf102
GSE40272	X42973	83636	C19orf12
GSE40272	X19209	58505	OSTC
GSE40272	X35784	7381	UQCRB
GSE40272	X2289	85406	DNAJC14
GSE40272	X29885	4801	NFYB
GSE40272	X9667	30968	STOML2
GSE40272	X32715	6432	SRSF7
GSE40272	X13105	25874	BRP44
GSE40272	X21987	3094	HINT1
GSE40272	X32415	51125	GOLGA7
GSE40272	X12042	10921	RNPS1
GSE40272	X13734	94005	PIGS
GSE40272	X30844	23463	ICMT
GSE40272	X16522	10237	SLC35B1
GSE40272	X41768	567	B2M
GSE40272	X12049	8697	CDC23
GSE40272	X27974	51808	PHAX
GSE40272	X4300	150472	CBWD2
GSE40272	X6143	6301	SARS
GSE40272	X28433	9282	MED14
GSE40272	X21617	10713	USP39
GSE40272	X29318	51389	RWDD1
GSE40272	X40450	2501	FTH1P8
GSE40272	X38587	567	B2M
GSE40272	X31373	473	RERE
GSE40272	X22390	10241	CALCOCO2
GSE40272	X38267	23001	WDFY3
GSE40272	X29191	79036	C19orf50
GSE40272	X33392	84084	RAB6C
GSE40272	X22262	23505	TMEM131
GSE40272	X28120	9107	MTMR6
GSE40272	X805	7170	TPM3
GSE40272	X32279	129285	KLRAQ1
GSE40272	X42282	3417	IDH1
GSE40272	X15431	488	ATP2A2
GSE40272	X25026	8887	TAX1BP1
GSE40272	X21427	3151	HMGN2
GSE40272	X23919	56930	LOC56930
GSE40272	X5314	9556	C14orf2
GSE40272	X35829	521	ATP5I

GSE40272	X15250	23585	TMEM50A
GSE40272	X42882	3895	KTN1
GSE40272	X10488	824	CAPN2
GSE40272	X13389	567	B2M
GSE40272	X20251	22936	ELL2
GSE40272	X27867	51187	RSL24D1
GSE40272	X21564	144871	LOC144871
GSE40272	X39193	8503	PIK3R3
GSE40272	X15435	11143	KAT7
GSE40272	X43802	84255	SLC37A3
GSE40272	X8658	8533	COPS3
GSE40272	X19806	8284	KDM5D
GSE40272	X8803	64801	ARV1
GSE40272	X39209	375743	PTAR1
GSE40272	X6723	2958	GTF2A2
GSE40272	X4805	10987	COPS5
GSE40272	X6969	51014	TMED7
GSE40272	X1271	22803	XRN2
GSE40272	X18535	54949	SDHAF2
GSE40272	X6248	55327	LIN7C
GSE40272	X13743	58478	ENOPH1
GSE40272	X19798	84295	PHF6
GSE40272	X30577	3181	HNRNPA2B1
GSE40272	X3473	1345	COX6C
GSE40272	X26286	8446	DUSP11
GSE40272	X36667	9867	PJA2
GSE40272	X43298	55852	TEX2
GSE40272	X43503	26953	RANBP6
GSE40272	X37150	439982	LOC439982
GSE40272	X42797	79086	C19orf42
GSE40272	X10073	55829	SELS
GSE40272	X25965	11328	FKBP9
GSE40272	X13360	51119	SBDS
GSE40272	X1239	54464	XRN1
GSE40272	X44026	3858	KRT10
GSE40272	X32167	4090	SMAD5
GSE40272	X28709	1982	EIF4G2
GSE40272	X22167	7982	ST7
GSE40272	X32065	3187	HNRNPH1
GSE40272	X14385	694	BTG1
GSE40272	X32516	23204	ARL6IP1
GSE40272	X7972	25879	DCAF13
GSE40272	X13923	567	B2M
GSE40272	X28239	567	B2M
GSE40272	X8671	1389	CREBL2
GSE40272	X25452	6389	SDHA
GSE40272	X19797	6729	SRP54
GSE40272	X26556	26355	FAM162A
GSE40272	X11243	51264	MRPL27
GSE40272	X11861	9836	LCMT2
GSE40272	X40686	26133	TRPC4AP
GSE40272	X42505	9989	PPP4R1
GSE40272	X37709	8655	DYNLL1

GSE40272	X39351	6838	SURF6
GSE40272	X22170	284207	METRNL
GSE40272	X22304	805	CALM2
GSE40272	X9118	1345	COX6C
GSE40272	X36668	10787	NCKAP1
GSE40272	X11557	57505	AARS2
GSE40272	X39099	23390	ZDHHC17
GSE40272	X43203	388345	LOC388345
GSE40272	X23585	2110	ETFDH
GSE40272	X31575	3988	LIPA
GSE40272	X23810	58487	CREBZF
GSE40272	X16270	54882	ANKHD1
GSE40272	X31427	84817	TXNDC17
GSE40272	X13307	390916	NUDT19
GSE40272	X9026	9581	PREPL
GSE40272	X32416	55967	NDUFA12
GSE40272	X27591	57062	DDX24
GSE40272	X8394	10600	USP16
GSE40272	X37691	23741	EID1
GSE40272	X27525	253943	YTHDF3
GSE40272	X7037	6185	RPN2
GSE40272	X32206	805	CALM2
GSE40272	X28560	55163	PNPO
GSE40272	X3266	5908	RAP1B
GSE40272	X26315	9895	TECPR2
GSE40272	X32532	29803	REPIN1
GSE40272	X8785	10531	PITRM1
GSE40272	X27449	6389	SDHA
GSE40272	X42657	9371	KIF3B
GSE40272	X18145	284672	PTGES3P
GSE40272	X41305	80829	ZFP91
GSE40272	X8836	23637	RABGAP1
GSE40272	X10921	81688	C6orf62
GSE40272	X36610	9325	TRIP4
GSE40272	X36760	9184	BUB3
GSE40272	X43977	10776	ARPP19
GSE40272	X3267	10473	HMGN4
GSE40272	X38721	9443	MED7
GSE40272	X32154	8559	PRPF18
GSE40272	X32508	84973	SNHG7
GSE40272	X43620	567	B2M
GSE40272	X41804	23412	COMMD3
GSE40272	X6904	7458	EIF4H
GSE40272	X35500	8776	MTMR1
GSE40272	X21463	130535	KCTD18
GSE40272	X23747	27249	MMADHC
GSE40272	X37286	29081	METTL5
GSE40272	X12918	740	MRPL49
GSE40272	X1649	7511	XPNPEP1
GSE40272	X35321	1434	CSE1L
GSE40272	X41231	567	B2M
GSE40272	X34541	6235	RPS29
GSE40272	X19296	55837	EAPP

GSE40272	X42485	663	BNIP2
GSE40272	X40058	56942	C16orf61
GSE40272	X13425	8725	C19orf2
GSE40272	X16504	51105	PHF20L1
GSE40272	X30599	7803	PTP4A1
GSE40272	X38326	64784	CRTC3
GSE40272	X34735	567	B2M
GSE40272	X5897	23176	SEPT8
GSE40272	X40570	3275	PRMT2
GSE40272	X41244	85379	KIAA1671
GSE40272	X30777	10550	ARL6IP5
GSE40272	X18930	10073	SNUPN
GSE40272	X7024	8550	MAPKAPK5
GSE40272	X41282	54664	TMEM106B
GSE40272	X875	6780	STAU1
GSE40272	X29830	10575	CCT4
GSE40272	X36433	27089	UQCRQ
GSE40272	X3944	4724	NDUFS4
GSE40272	X24845	2887	GRB10
GSE40272	X8679	26272	FBXO4
GSE40272	X21377	6386	SDCBP
GSE40272	X36379	1452	CSNK1A1
GSE40272	X4186	84272	YIPF4
GSE40272	X11332	9086	EIF1AY
GSE40272	X36274	10094	ARPC3
GSE40272	X7170	8295	TRRAP
GSE40272	X305	10513	APPBP2
GSE40272	X42997	54832	VPS13C
GSE40272	X39521	388642	LOC388642
GSE40272	X39781	51642	MRPL48
GSE40272	X40958	7324	UBE2E1
GSE40272	X25060	567	B2M
GSE40272	X38952	7334	UBE2N
GSE40272	X20059	1603	DAD1
GSE40272	X10197	26128	KIAA1279
GSE40272	X41070	307	ANXA4
GSE40272	X30743	285636	C5orf51
GSE40272	X20372	5218	CDK14
GSE40272	X15757	25926	NOL11
GSE40272	X40384	10140	TOB1
GSE40272	X43572	4594	MUT
GSE40272	X3830	9646	CTR9
GSE40272	X27323	6730	SRP68
GSE40272	X20838	51451	LCMT1
GSE40272	X22184	60	ACTB
GSE40272	X41409	9533	POLR1C
GSE40272	X37307	2590	GALNT2
GSE40272	X40140	79672	FN3KRP
GSE40272	X24522	567	B2M
GSE40272	X18119	51466	EVL
GSE40272	X39969	79081	C11orf48
GSE40272	X31018	6389	SDHA
GSE40272	X16977	134510	UBLCP1

GSE40272	X32072	6950	TCP1
GSE40272	X28604	10096	ACTR3
GSE40272	X26143	203068	TUBB
GSE40272	X41383	10944	C11orf58
GSE40272	X32170	10899	JTB
GSE40272	X31352	523	ATP6V1A
GSE40272	X2022	5134	PDCD2
GSE40272	X41092	28998	MRPL13
GSE40272	X32533	8933	FAM127A
GSE40272	X2406	1349	COX7B
GSE40272	X31386	4725	NDUFS5
GSE40272	X11654	10971	YWHAQ
GSE40272	X13116	23560	GTPBP4
GSE40272	X15083	10142	AKAP9
GSE40272	X36426	2885	GRB2
GSE40272	X20075	5721	PSME2
GSE40272	X4579	9857	CEP350
GSE40272	X25569	390956	LOC390956
GSE40272	X35706	27248	ERLEC1
GSE40272	X14997	7076	TIMP1
GSE40272	X19894	81853	TMEM14B
GSE40272	X35755	23041	MON2
GSE40272	X24083	51010	EXOSC3
GSE40272	X29522	84186	ZCCHC7
GSE40272	X37526	7458	EIF4H
GSE40272	X37798	4702	NDUFA8
GSE40272	X42077	80267	EDEM3
GSE40272	X44522	9883	POM121
GSE40272	X36278	2224	FDPS
GSE40272	X30668	2332	FMR1
GSE40272	X35006	124808	CCDC43
GSE40272	X33647	5925	RB1
GSE40272	X2274	10618	TGOLN2
GSE40272	X14436	59286	UBL5
GSE40272	X27526	26959	HBP1
GSE40272	X30580	56252	YLPM1
GSE40272	X22612	79157	MFSD11
GSE40272	X16752	64110	MAGEF1
GSE40272	X40270	9296	ATP6V1F
GSE40272	X28556	153830	RNF145
GSE40272	X36778	3183	HNRNPC
GSE40272	X17705	51009	DERL2
GSE40272	X29048	8408	ULK1
GSE40272	X15753	51807	TUBA8
GSE40272	X39562	10447	FAM3C
GSE40272	X16226	4853	NOTCH2
GSE40272	X17489	84892	C3orf39
GSE40272	X25048	55862	ECHDC1
GSE40272	X31800	54989	ZNF770
GSE40272	X26573	54925	ZNF434
GSE40272	X27968	92305	TMEM129
GSE40272	X20076	7534	YWHAZ
GSE40272	X37882	9584	RBM39

GSE40272	X42193	11112	HIBADH
GSE40272	X42410	10413	YAP1
GSE40272	X36206	79176	FBXL15
GSE40272	X12829	6227	RPS21
GSE40272	X25101	2739	GLO1
GSE40272	X2983	26268	FBXO9
GSE40272	X8365	8726	EED
GSE40272	X36838	170622	COMMD6
GSE40272	X8215	754	PTTG1IP
GSE40272	X20049	90843	TCEAL8
GSE40272	X13160	51019	CCDC53
GSE40272	X900	25912	C1orf43
GSE40272	X41115	57599	WDR48
GSE40272	X23947	23451	SF3B1
GSE40272	X9650	81545	FBXO38
GSE40272	X18141	5786	PTPRA
GSE40272	X16142	3190	HNRNPK
GSE40272	X36834	51634	RBMX2
GSE40272	X23244	23271	CAMSAP2
GSE40272	X27625	9728	SECISBP2L
GSE40272	X6847	8661	EIF3A
GSE40272	X22721	51455	REV1
GSE40272	X5633	83707	TRPT1
GSE40272	X11983	51399	TRAPPC4
GSE40272	X5584	25814	ATXN10
GSE40272	X28607	342125	TMC3
GSE40272	X33682	254225	RNF169
GSE40272	X20352	84191	FAM96A
GSE40272	X5434	9117	SEC22C
GSE40272	X10717	56910	STARD7
GSE40272	X29395	51699	VPS29
GSE40272	X28991	54439	RBM27
GSE40272	X38090	10966	RAB40B
GSE40272	X13278	8454	CUL1
GSE40272	X26916	6389	SDHA
GSE40272	X37431	84181	CHD6
GSE40272	X25354	10980	COPS6
GSE40272	X5395	23344	ESYT1
GSE40272	X1480	150580	LOC150580
GSE40272	X4262	6418	SET
GSE40272	X12603	6389	SDHA
GSE40272	X42251	26146	TRAF3IP1
GSE40272	X6007	57129	MRPL47
GSE40272	X37096	56339	METTL3
GSE40272	X6872	5987	TRIM27
GSE40272	X5188	6767	ST13
GSE40272	X14387	1315	COPB1
GSE40272	X28245	4267	CD99
GSE40272	X39972	285172	FAM126B
GSE40272	X42995	55330	CNO
GSE40272	X8216	7982	ST7
GSE40272	X9888	55958	KLHL9
GSE40272	X19356	54880	BCOR

GSE40272	X38399	26175	C14orf109
GSE40272	X20395	292	SLC25A5
GSE40272	X28853	51123	ZNF706
GSE40272	X27256	23341	DNAJC16
GSE40272	X29285	25798	BRI3
GSE40272	X31292	7533	YWHAH
GSE40272	X33420	5861	RAB1A
GSE40272	X32882	146053	RPS3AP47
GSE40272	X31893	8826	IQGAP1
GSE40272	X20001	3550	IK
GSE40272	X6701	375113	PPIAL3
GSE40272	X29674	6902	TBCA
GSE40272	X31975	8724	SNX3
GSE40272	X43426	10975	UQCR11
GSE40272	X30064	441561	LOC441561
GSE40272	X24065	51780	KDM3B
GSE40272	X37001	509	ATP5C1
GSE40272	X40399	56254	RNF20
GSE40272	X38597	51441	YTHDF2
GSE40272	X37143	56270	WDR45L
GSE40272	X2195	51474	LIMA1
GSE40272	X34936	64841	GNPNAT1
GSE40272	X39910	6389	SDHA
GSE40272	X19327	442589	LOC442589
GSE40272	X31304	60682	SMAP1
GSE40272	X39194	9061	PAPSS1
GSE40272	X44323	5440	POLR2K
GSE40272	X21024	56674	TMEM9B
GSE40272	X21622	51290	ERGIC2
GSE40272	X18796	113402	SFT2D1
GSE40272	X37668	84888	SPPL2A
GSE40272	X22087	4259	MGST3
GSE40272	X38946	9950	GOLGA5
GSE40272	X33415	60	ACTB
GSE40272	X20006	10632	ATP5L
GSE40272	X43935	9791	PTDSS1
GSE40272	X14891	23658	LSM5
GSE40272	X10815	51691	NAA38
GSE40272	X27238	64924	SLC30A5
GSE40272	X14750	8334	HIST1H2AC
GSE40272	X40128	55364	IMPACT
GSE40272	X6805	1964	EIF1AX
GSE40272	X6720	5511	PPP1R8
GSE40272	X24722	9991	ROD1
GSE40272	X13458	8565	YARS
GSE40272	X7052	9167	COX7A2L
GSE40272	X11297	5,00E+03	ORC4
GSE40272	X31532	2970	GTF2IP1
GSE40272	X26749	441542	LOC441542
GSE40272	X36168	60485	SAV1
GSE40272	X10038	27238	GPKOW
GSE40272	X30214	989	SEPT7
GSE40272	X34479	8481	OFD1

GSE40272	X27921	5478	PPIA
GSE40272	X32620	6464	SHC1
GSE40272	X2909	4048	LTA4H
GSE40272	X15794	539	ATP5O
GSE40272	X2413	25980	C20orf4
GSE40272	X31097	64343	AZI2
GSE40272	X37404	63931	MRPS14
GSE40272	X27586	9741	LAPTM4A
GSE40272	X12207	79018	C17orf39
GSE40272	X20490	6993	DYNLT1
GSE40272	X24648	389156	RPL21P39
GSE40272	X24346	8763	CD164
GSE40272	X17710	6675	UAP1
GSE40272	X17608	59343	SENP2
GSE40272	X10799	9782	MATR3
GSE40272	X11119	54458	PRR13
GSE40272	X32886	7114	TMSB4X
GSE40272	X1839	5756	TWF1
GSE40272	X25059	6389	SDHA
GSE40272	X19290	4830	NME1
GSE40272	X11681	83955	NACAP1
GSE40272	X20033	390498	LOC390498
GSE40272	X6028	64072	CDH23
GSE40272	X13133	25977	NECAP1
GSE40272	X14269	3927	LASP1
GSE40272	X42040	10531	PITRM1
GSE40272	X42278	10951	CBX1
GSE40272	X39097	8939	FUBP3
GSE40272	X21883	6389	SDHA
GSE40272	X27817	6917	TCEA1
GSE40272	X2760	5832	ALDH18A1
GSE40272	X44408	55707	NECAP2
GSE40272	X35244	3329	HSPD1
GSE40272	X43213	3476	IGBP1
GSE40272	X2416	51566	ARMCX3
GSE40272	X43280	6391	SDHC
GSE40272	X21001	55437	STRADB
GSE40272	X44004	2971	GTF3A
GSE40272	X28459	254225	RNF169
GSE40272	X41135	23389	MED13L
GSE40272	X30745	10617	STAMBP
GSE40272	X36475	3920	LAMP2
GSE40272	X14466	440577	LOC440577
GSE40272	X31543	57561	ARRDC3
GSE40272	X38543	7155	TOP2B
GSE40272	X33090	5128	CDK17
GSE40272	X39585	54918	CMTM6
GSE40272	X12070	6389	SDHA
GSE40272	X12096	3476	IGBP1
GSE40272	X8694	9794	MAML1
GSE40272	X19509	26235	FBXL4
GSE40272	X1292	26065	LSM14A
GSE40272	X39443	23077	MYCBP2

GSE40272	X17589	7251	TSG101
GSE40272	X15122	23673	STX12
GSE40272	X8457	2342	FNTB
GSE40272	X37980	56947	MFF
GSE40272	X39100	26060	APPL1
GSE40272	X18173	6185	RPN2
GSE40272	X8417	10797	MTHFD2
GSE40272	X30939	5007	OSBP
GSE40272	X40548	55100	WDR70
GSE40272	X41117	10217	CTDSPL
GSE40272	X20828	6118	RPA2
GSE40272	X31031	7386	UQCRFS1
GSE40272	X8237	3281	HSBP1
GSE40272	X27432	83787	ARMC10
GSE40272	X42347	4077	NBR1
GSE40272	X11864	8455	ATRN
GSE40272	X33289	442133	LOC442133
GSE40272	X18900	7405	UVRAG
GSE40272	X31754	3020	H3F3A
GSE40272	X36505	1665	DHX15
GSE40272	X20549	80777	CYB5B
GSE40272	X26944	54874	FNBP1L
GSE40272	X44431	10284	SAP18
GSE40272	X28460	2802	GOLGA3
GSE40272	X22832	6434	TRA2B
GSE40272	X28554	9236	CCPG1
GSE40272	X43886	1738	DLD
GSE40272	X30975	23568	ARL2BP
GSE40272	X23940	134505	LOC134505
GSE40272	X12665	900	CCNG1
GSE40272	X27819	4299	AFF1
GSE40272	X5191	539	ATP5O
GSE40272	X13896	5930	RBBP6
GSE40272	X42047	23379	KIAA0947
GSE40272	X12284	23270	TSPYL4
GSE40272	X42297	7534	YWHAZ
GSE40272	X29685	23013	SPEN
GSE40272	X42714	22826	DNAJC8
GSE40272	X31138	6635	SNRPE
GSE40272	X5330	533	ATP6V0B
GSE40272	X28428	11143	KAT7
GSE40272	X17512	94239	H2AFV
GSE40272	X40492	6164	RPL34
GSE40272	X37097	11152	WDR45
GSE40272	X29943	79718	TBL1XR1
GSE40272	X27427	1431	CS
GSE40272	X3492	5441	POLR2L
GSE40272	X41193	829	CAPZA1
GSE40272	X7082	2504	FTH1P12
GSE40272	X44163	1974	EIF4A2
GSE40272	X30767	6651	SON
GSE40272	X7083	440525	LOC440525
GSE40272	X27810	9748	SLK

GSE40272	X5151	57504	MTA3
GSE40272	X25046	285086	LOC285086
GSE40272	X23512	6309	SC5DL
GSE40272	X39517	6185	RPN2
GSE40272	X21711	3324	HSP90AA2
GSE40272	X36593	23399	CTDNEP1
GSE40272	X16121	51246	SHISA5
GSE40272	X30002	1859	DYRK1A
GSE40272	X33020	6389	SDHA
GSE40272	X17381	2926	GRSF1
GSE40272	X37786	51651	PTRH2
GSE40272	X30630	6389	SDHA
GSE40272	X8889	6389	SDHA
GSE40272	X15944	8899	PRPF4B
GSE40272	X38083	10890	RAB10
GSE40272	X43310	51699	VPS29
GSE40272	X37634	26292	MYCBP
GSE40272	X42433	9491	PSMF1
GSE40272	X19958	51542	VPS54
GSE40272	X19493	6389	SDHA
GSE40272	X8865	1595	CYP51A1
GSE40272	X7942	51119	SBDS
GSE40272	X40616	55486	PARL
GSE40272	X20543	8720	MBTPS1
GSE40272	X32866	152137	CCDC50
GSE40272	X5307	5814	PURB
GSE40272	X40105	55651	NHP2
GSE40272	X4860	23118	TAB2
GSE40272	X18222	7417	VDAC2
GSE40272	X39733	23326	USP22
GSE40272	X17694	80145	THOC7
GSE40272	X21135	54497	HEATR5B
GSE40272	X6157	400452	LOC400452
GSE40272	X17801	51693	TRAPPC2L
GSE40272	X37840	6897	TARS
GSE40272	X39387	57804	POLD4
GSE40272	X42274	440866	LOC440866
GSE40272	X6667	9552	SPAG7
GSE40272	X7007	51081	MRPS7
GSE40272	X28042	29121	CLEC2D
GSE40272	X35814	10054	UBA2
GSE40272	X14610	23386	NUDCD3
GSE40272	X27815	64756	ATPAF1
GSE40272	X38000	65117	RSRC2
GSE40272	X24411	4700	NDUFA6
GSE40272	X26450	119504	ANAPC16
GSE40272	X27136	7514	XPO1
GSE40272	X40431	84893	FBXO18
GSE40272	X10048	498	ATP5A1
GSE40272	X31874	84661	DPY30
GSE40272	X14471	9446	GSTO1
GSE40272	X22938	51493	C22orf28
GSE40272	X25364	29979	UBQLN1

GSE40272	X35625	2107	ETF1
GSE40272	X16321	26520	TIMM9
GSE40272	X23673	64083	GOLPH3
GSE40272	X23715	10694	CCT8
GSE40272	X2272	25793	FBXO7
GSE40272	X20371	4848	CNOT2
GSE40272	X36810	9512	PMPCB
GSE40272	X4530	221154	EFHA1
GSE40272	X19037	6117	RPA1
GSE40272	X44168	388532	RPL21P131
GSE40272	X1444	7323	UBE2D3
GSE40272	X16380	223	ALDH9A1
GSE40272	X31028	5757	PTMA
GSE40272	X6479	54543	TOMM7
GSE40272	X28922	55197	RPRD1A
GSE40272	X37311	79602	ADIPOR2
GSE40272	X34522	10277	UBE4B
GSE40272	X19909	55039	TRMT12
GSE40272	X38633	4831	NME2
GSE40272	X26962	10641	NPRL2
GSE40272	X18678	10120	ACTR1B
GSE40272	X36196	6389	SDHA
GSE40272	X18579	10959	TMED2
GSE40272	X26810	122704	MRPL52
GSE40272	X38599	10728	PTGES3
GSE40272	X12545	79893	GGNBP2
GSE40272	X21930	4904	YBX1
GSE40272	X42384	8992	ATP6V0E1
GSE40272	X20823	29117	BRD7
GSE40272	X29890	5202	PFDN2
GSE40272	X31684	50854	C6orf48
GSE40272	X25333	4717	NDUFC1
GSE40272	X5044	1781	DYNC1I2
GSE40272	X22586	3098	HK1
GSE40272	X43367	55272	IMP3
GSE40272	X44284	5538	PPT1
GSE40272	X20168	60488	MRPS35
GSE40272	X8897	5706	PSMC6
GSE40272	X43091	2339	FNTA
GSE40272	X22743	9874	TLK1
GSE40272	X41993	51247	PAIP2
GSE40272	X26262	7764	ZNF217
GSE40272	X24967	60	ACTB
GSE40272	X35658	6389	SDHA
GSE40272	X36729	6389	SDHA
GSE40272	X516	376	ARF2
GSE40272	X34173	442441	LOC442441
GSE40272	X6335	439953	LOC439953
GSE40272	X10074	51199	NIN
GSE40272	X28544	8813	DPM1
GSE40272	X34029	57169	ZNFX1
GSE40272	X5260	29074	MRPL18
GSE40272	X44207	6613	SUMO2

GSE40272	X15434	4738	NEDD8
GSE40272	X19076	10552	ARPC1A
GSE40272	X27620	84280	BTBD10
GSE40272	X17636	6389	SDHA
GSE40272	X41951	64689	GORASP1
GSE40272	X18169	6389	SDHA
GSE40272	X21183	11017	SNRNP27
GSE40272	X17741	5686	PSMA5
GSE40272	X20586	6647	SOD1
GSE40272	X22970	2130	EWSR1
GSE40272	X9232	51082	POLR1D
GSE40272	X37099	9296	ATP6V1F
GSE40272	X6943	4077	NBR1
GSE40272	X27719	51255	RNF181
GSE40272	X3652	6709	SPTAN1
GSE40272	X44413	29979	UBQLN1
GSE40272	X23365	51255	RNF181
GSE40272	X27098	10197	PSME3
GSE40272	X1439	57095	PITHD1
GSE40272	X37676	2071	ERCC3
GSE40272	X40876	26985	AP3M1
GSE40272	X8832	1997	ELF1
GSE40272	X26606	582	BBS1
GSE40272	X38069	27068	PPA2
GSE40272	X40975	114883	OSBPL9
GSE40272	X17665	6138	RPL15
GSE40272	X32464	2497	FTH1P2
GSE40272	X31163	6389	SDHA
GSE40272	X8139	23191	CYFIP1
GSE40272	X30607	9939	RBM8A
GSE40272	X17071	8683	SRSF9
GSE40272	X30458	4673	NAP1L1
GSE40272	X39379	1998	ELF2
GSE40272	X39489	4299	AFF1
GSE40272	X35747	1540	CYLD
GSE40272	X42361	9276	COPB2
GSE40272	X17296	55577	NAGK
GSE40272	X20026	6389	SDHA
GSE40272	X15450	10724	MGEA5
GSE40272	X35055	5701	PSMC2
GSE40272	X40047	119358	SNRPD2P1
GSE40272	X6155	150928	PTMAP5
GSE40272	X15720	55505	NOP10
GSE40272	X22647	84945	ABHD13
GSE40272	X15805	539	ATP5O
GSE40272	X34684	442060	LOC442060
GSE40272	X37342	51185	CRBN
GSE40272	X36595	2547	XRCC6
GSE40272	X5873	3185	HNRNPF
GSE40272	X26549	7528	YY1
GSE40272	X8610	54940	OCIAD1
GSE40272	X40057	51667	NUB1
GSE40272	X25626	4701	NDUFA7

GSE40272	X33814	6222	RPS18
GSE40272	X15863	6144	RPL21
GSE40272	X38329	29110	TBK1
GSE40272	X35009	9419	CRIPT
GSE40272	X24160	5584	PRKCI
GSE40272	X29287	391532	LOC391532
GSE40272	X40443	6389	SDHA
GSE40272	X31594	9698	PUM1
GSE40272	X9830	2503	FTH1P11
GSE40272	X37282	150472	CBWD2
GSE40272	X38798	51100	SH3GLB1
GSE40272	X37728	79665	DHX40
GSE70768	ILMN_1739161	8611	PPAP2A
GSE70768	ILMN_1720114	51053	GMNN
GSE70768	ILMN_1663575	256227	MGC87042
GSE70768	ILMN_1661593	51030	FAM18B
GSE70768	ILMN_1733094	26872	STEAP1
GSE70768	ILMN_1677607	6309	SC5DL
GSE70768	ILMN_1659415	8649	MAP2K1IP1
GSE70768	ILMN_1708416	23204	ARL6IP1
GSE70768	ILMN_1757074	2790	GNG10
GSE70768	ILMN_1724139	114908	TMEM123
GSE70768	ILMN_3239610	100133565	LOC100133565
GSE70768	ILMN_1768050	60592	SCOC
GSE70768	ILMN_2340565	27032	ATP2C1
GSE70768	ILMN_1702177	2739	GLO1
GSE70768	ILMN_1736888	51128	SAR1B
GSE70768	ILMN_1661895	51050	PI15
GSE70768	ILMN_1708627	653226	LOC653226
GSE70768	ILMN_2228873	83930	STARD3NL
GSE70768	ILMN_1810759	27249	C2orf25
GSE70768	ILMN_2094905	51397	COMMD10
GSE70768	ILMN_1703337	441763	LOC441763
GSE70768	ILMN_1745076	9685	CLINT1
GSE70768	ILMN_2333594	6613	SUMO2
GSE70768	ILMN_2343278	8611	PPAP2A
GSE70768	ILMN_2333367	2280	FKBP1A
GSE70768	ILMN_2334760	51566	ARMCX3
GSE70768	ILMN_1772743	10026	PIGK
GSE70768	ILMN_2225318	6611	SMS
GSE70768	ILMN_2094166	51510	CHMP5
GSE70768	ILMN_1741176	25978	CHMP2B
GSE70768	ILMN_2041293	6713	SQLE
GSE70768	ILMN_1792689	8334	HIST1H2AC
GSE70768	ILMN_1723884	378	ARF4
GSE70768	ILMN_2307740	4179	CD46
GSE70768	ILMN_1790315	79846	C7orf63
GSE70768	ILMN_1764168	6814	STXBP3
GSE70768	ILMN_1682316	51592	TRIM33
GSE70768	ILMN_1798657	79718	TBL1XR1
GSE70768	ILMN_1691097	3320	HSP90AA1
GSE70768	ILMN_1784753	51247	PAIP2
GSE70768	ILMN_1793474	3638	INSIG1

GSE70768	ILMN_3200921	642590	LOC642590
GSE70768	ILMN_2279635	1982	EIF4G2
GSE70768	ILMN_1731640	286527	MGC39900
GSE70768	ILMN_1694305	6611	SMS
GSE70768	ILMN_1680781	64430	C14orf135
GSE70768	ILMN_1685636	3781	KCNN2
GSE70768	ILMN_1770653	114569	MAL2
GSE70768	ILMN_1724376	27248	C2orf30
GSE70768	ILMN_1696432	3417	IDH1
GSE70768	ILMN_1857915	401397	LOC401397
GSE70768	ILMN_1674522	25994	HIGD1A
GSE70768	ILMN_1734895	113402	SFT2D1
GSE70768	ILMN_1699476	6120	RPE
GSE70768	ILMN_3198367	646347	LOC646347
GSE70768	ILMN_1720422	9908	G3BP2
GSE70768	ILMN_1785191	28978	TMEM14A
GSE70768	ILMN_1782609	10735	STAG2
GSE70768	ILMN_2357272	9774	BCLAF1
GSE70768	ILMN_2395974	10935	PRDX3
GSE70768	ILMN_2352077	9365	KL
GSE70768	ILMN_2381753	9908	G3BP2
GSE70768	ILMN_1692177	8848	TSC22D1
GSE70768	ILMN_1653871	10135	NAMPT
GSE70768	ILMN_2180885	80157	FLJ21511
GSE70768	ILMN_1672405	51014	TMED7
GSE70768	ILMN_2383435	5716	PSMD10
GSE70768	ILMN_2394296	51128	SAR1B
GSE70768	ILMN_1677396	54602	NDFIP2
GSE70768	ILMN_1786532	10175	CNIH
GSE70768	ILMN_1787567	8848	TSC22D1
GSE70768	ILMN_2360705	2181	ACSL3
GSE70768	ILMN_1772946	29966	STRN3
GSE70768	ILMN_2353033	8939	FUBP3
GSE70768	ILMN_1701681	90701	SEC11C
GSE70768	ILMN_2395969	10935	PRDX3
GSE70768	ILMN_3177285	10236	HNRNPR
GSE70768	ILMN_1751234	29071	C1GALT1C1
GSE70768	ILMN_2368971	5718	PSMD12
GSE70768	ILMN_1781761	22875	ENPP4
GSE70768	ILMN_2224444	83891	SNX25
GSE70768	ILMN_2090558	27249	C2orf25
GSE70768	ILMN_2410742	221037	JMJD1C
GSE70768	ILMN_1807994	57092	PCNP
GSE70768	ILMN_1763265	57132	CHMP1B
GSE70768	ILMN_2105308	8813	DPM1
GSE70768	ILMN_1706817	27230	SERP1
GSE70768	ILMN_2111918	64430	C14orf135
GSE70768	ILMN_1776102	5716	PSMD10
GSE70768	ILMN_1787657	9069	CLDN12
GSE70768	ILMN_1732534	51510	CHMP5
GSE70768	ILMN_2119945	4709	NDUFB3
GSE70768	ILMN_1711766	6500	SKP1A
GSE70768	ILMN_1744647	55832	CAND1

GSE70768	ILMN_3251440	5286	PIK3C2A
GSE70768	ILMN_1800220	51133	KCTD3
GSE70768	ILMN_1708130	26298	EHF
GSE70768	ILMN_1773117	54880	BCOR
GSE70768	ILMN_1699112	1315	COPB1
GSE70768	ILMN_2325574	113201	CASC4
GSE70768	ILMN_1722522	905	CCNT2
GSE70768	ILMN_3249578	100132394	LOC100132394
GSE70768	ILMN_2099594	6726	SRP9
GSE70768	ILMN_1761721	55737	VPS35
GSE70768	ILMN_2131880	84661	DPY30
GSE70768	ILMN_1741477	4089	SMAD4
GSE70768	ILMN_1757287	5597	MAPK6
GSE70768	ILMN_1735347	84693	MCEE
GSE70768	ILMN_3243011	641844	LOC641844
GSE70768	ILMN_1670821	246126	CYorf15A
GSE70768	ILMN_2225608	57579	FAM135A
GSE70768	ILMN_2413259	122809	SOCS4
GSE70768	ILMN_2174369	60481	ELOVL5
GSE70768	ILMN_1662129	5955	RCN2
GSE70768	ILMN_1763404	653226	LOC653226
GSE70768	ILMN_1659786	54482	CCDC76
GSE70768	ILMN_1681249	55605	KIF21A
GSE70768	ILMN_1690690	11196	SEC23IP
GSE70768	ILMN_1751956	4259	MGST3
GSE70768	ILMN_1776005	58505	OSTC
GSE70768	ILMN_1748926	84928	TMEM209
GSE70768	ILMN_1717337	64844	7-Mar
GSE70768	ILMN_2344298	261729	STEAP2
GSE70768	ILMN_1734833	4683	NBN
GSE70768	ILMN_1676296	8611	PPAP2A
GSE70768	ILMN_1751120	8365	HIST1H4H
GSE70768	ILMN_1784333	9728	SECISBP2L
GSE70768	ILMN_1713143	11222	MRPL3
GSE70768	ILMN_1656682	51582	AZIN1
GSE70768	ILMN_1672366	84992	PIGY
GSE70768	ILMN_1720996	6558	SLC12A2
GSE70768	ILMN_1661799	25994	HIGD1A
GSE70768	ILMN_1669881	27075	TSPAN13
GSE70768	ILMN_2234758	6727	SRP14
GSE70768	ILMN_1814770	131034	CPNE4
GSE70768	ILMN_1738736	8723	SNX4
GSE70768	ILMN_1705629	26872	STEAP1
GSE70768	ILMN_1743299	5828	PXMP3
GSE70768	ILMN_1655316	5706	PSMC6
GSE70768	ILMN_2388466	7072	TIA1
GSE70768	ILMN_1711516	523	ATP6V1A
GSE70768	ILMN_2162328	5805	PTS
GSE70768	ILMN_1759983	1810	DR1
GSE70768	ILMN_1709043	55848	C9orf46
GSE70768	ILMN_1751708	9445	ITM2B
GSE70768	ILMN_2330267	6059	ABCE1
GSE70768	ILMN_1763147	4712	NDUFB6

GSE70768	ILMN_2408645	653566	LOC653566
GSE70768	ILMN_3256004	100130003	LOC100130003
GSE70768	ILMN_1785071	6414	SEPP1
GSE70768	ILMN_1781952	4257	MGST1
GSE70768	ILMN_2176955	8065	CUL5
GSE70768	ILMN_2306565	10651	MTX2
GSE70768	ILMN_1683562	6637	SNRPG
GSE70768	ILMN_1710326	56650	CLDND1
GSE70768	ILMN_1738632	5573	PRKAR1A
GSE70768	ILMN_1672006	6741	SSB
GSE70768	ILMN_1766024	11235	PDCD10
GSE70768	ILMN_3244248	645203	TMEM14D
GSE70768	ILMN_1801928	7534	YWHAZ
GSE70768	ILMN_1651496	3017	HIST1H2BD
GSE70768	ILMN_1660890	33	ACADL
GSE70768	ILMN_1691570	29081	METTL5
GSE70768	ILMN_1772929	522	ATP5J
GSE70768	ILMN_1735461	9188	DDX21
GSE70768	ILMN_2367020	23480	SEC61G
GSE70768	ILMN_1673820	6596	HLTF
GSE70768	ILMN_2083469	8660	IRS2
GSE70768	ILMN_2352563	56650	CLDND1
GSE70768	ILMN_1727813	25874	BRP44
GSE70768	ILMN_1748291	163859	C1orf55
GSE70768	ILMN_2134110	58515	SELK
GSE70768	ILMN_1731612	51377	UCHL5
GSE70768	ILMN_1761981	84191	FAM96A
GSE70768	ILMN_1701696	644863	LOC644863
GSE70768	ILMN_1739497	404672	GTF2H5
GSE70768	ILMN_2194448	201595	STT3B
GSE70768	ILMN_1806634	23530	NNT
GSE70768	ILMN_3237850	285636	C5orf51
GSE70768	ILMN_1770378	10393	ANAPC10
GSE70768	ILMN_1659753	3920	LAMP2
GSE70768	ILMN_1814573	55783	FTSJD1
GSE70768	ILMN_1700915	648	BMI1
GSE70768	ILMN_2340259	5142	PDE4B
GSE70768	ILMN_3243945	440145	C13orf37
GSE70768	ILMN_1666156	9643	MORF4L2
GSE70768	ILMN_1776297	2803	GOLGA4
GSE70768	ILMN_1729175	26273	FBXO3
GSE70768	ILMN_2151056	119032	C10orf32
GSE70768	ILMN_1732071	8349	HIST2H2BE
GSE70768	ILMN_1766851	55863	TMEM126B
GSE70768	ILMN_1698001	4690	NCK1
GSE70768	ILMN_1732398	4246	SCGB2A1
GSE70768	ILMN_2220184	2673	GFPT1
GSE70768	ILMN_2330112	10175	CNIH
GSE70768	ILMN_2217809	84233	TMEM126A
GSE70768	ILMN_1737462	55074	OXR1
GSE70768	ILMN_1761519	1982	EIF4G2
GSE70768	ILMN_1749297	86	ACTL6A
GSE70768	ILMN_1767837	51026	GOLT1B

GSE70768	ILMN_1679727	1195	CLK1
GSE70768	ILMN_3246409	3187	HNRNPH1
GSE70768	ILMN_2169439	3685	ITGAV
GSE70768	ILMN_3251545	51510	CHMP5
GSE70768	ILMN_1690524	6845	VAMP7
GSE70768	ILMN_1671158	28998	MRPL13
GSE70768	ILMN_2378048	3190	HNRPK
GSE70768	ILMN_2324157	9039	UBA3
GSE70768	ILMN_1788462	262	AMD1
GSE70768	ILMN_1777663	7155	TOP2B
GSE70768	ILMN_2348093	522	ATP5J
GSE70768	ILMN_1652024	1456	CSNK1G3
GSE70768	ILMN_1756928	6252	RTN1
GSE70768	ILMN_2381758	9908	G3BP2
GSE70768	ILMN_2369924	4712	NDUFB6
GSE70768	ILMN_1684042	10282	BET1
GSE70768	ILMN_1698487	6392	SDHD
GSE70768	ILMN_3236481	100129086	LOC100129086
GSE70768	ILMN_1733356	25843	PREI3
GSE70768	ILMN_1780382	653566	LOC653566
GSE70768	ILMN_1722811	1027	CDKN1B
GSE70768	ILMN_1750394	25800	SLC39A6
GSE70768	ILMN_1798705	892	CCNC
GSE70768	ILMN_1764087	6856	SYPL1
GSE70768	ILMN_1807455	51635	DHRS7
GSE70768	ILMN_2090123	54505	DHX29
GSE70768	ILMN_2313851	114883	OSBPL9
GSE70768	ILMN_1758543	10175	CNIH
GSE70768	ILMN_1760338	643357	LOC643357
GSE70768	ILMN_1673282	3920	LAMP2
GSE70768	ILMN_3229859	25843	MOBKL3
GSE70768	ILMN_2351611	29979	UBQLN1
GSE70768	ILMN_1672124	51313	C4orf18
GSE70768	ILMN_1719032	27258	LSM3
GSE70768	ILMN_1805344	1655	DDX5
GSE70768	ILMN_1773576	8895	CPNE3
GSE70768	ILMN_1760087	1811	SLC26A3
GSE70768	ILMN_2390338	10477	UBE2E3
GSE70768	ILMN_1765409	8027	STAM
GSE70768	ILMN_1772278	85461	TANC1
GSE70768	ILMN_1807397	4711	NDUFB5
GSE70768	ILMN_2290618	9117	SEC22C
GSE70768	ILMN_2377174	6856	SYPL1
GSE70768	ILMN_1792508	9528	TMEM59
GSE70768	ILMN_1800602	25801	GCA
GSE70768	ILMN_1654939	10959	TMED2
GSE70768	ILMN_2076940	64769	C1orf149
GSE70768	ILMN_1756417	353322	ANKRD37
GSE70768	ILMN_1809101	261729	STEAP2
GSE70768	ILMN_1737283	286527	MGC39900
GSE70768	ILMN_1756999	5934	RBL2
GSE70768	ILMN_1742887	645058	LOC645058
GSE70768	ILMN_1764970	221037	JMJD1C

GSE70768	ILMN_1712748	51527	C14orf129
GSE70768	ILMN_3193306	26175	C14orf109
GSE70768	ILMN_1783684	648695	LOC648695
GSE70768	ILMN_1667430	8560	DEGS1
GSE70768	ILMN_1732489	84068	SLC10A7
GSE70768	ILMN_2402416	10049	DNAJB6
GSE70768	ILMN_1807710	3094	HINT1
GSE70768	ILMN_1777261	10447	FAM3C
GSE70768	ILMN_1731194	11171	STRAP
GSE70768	ILMN_2175131	51522	TMEM14C
GSE70768	ILMN_1755303	7764	ZNF217
GSE70768	ILMN_1753719	2958	GTF2A2
GSE70768	ILMN_1751492	51030	FAM18B
GSE70768	ILMN_1717765	55190	NUDT11
GSE70768	ILMN_1672191	515	ATP5F1
GSE70768	ILMN_1813491	10558	SPTLC1
GSE70768	ILMN_2142117	127018	LYPLAL1
GSE70768	ILMN_1798588	6596	HLTF
GSE70768	ILMN_1695423	928	CD9
GSE70768	ILMN_1667994	262	AMD1
GSE70768	ILMN_2354140	51126	NAT5
GSE70768	ILMN_2351309	7073	TIAL1
GSE70768	ILMN_1769319	7555	CNBP
GSE70768	ILMN_3187771	55449	C14orf167
GSE70768	ILMN_1708502	27125	AFF4
GSE70768	ILMN_1666096	2181	ACSL3
GSE70768	ILMN_1657857	51522	TMEM14C
GSE70768	ILMN_1787026	23480	SEC61G
GSE70768	ILMN_2062112	55854	ZC3H15
GSE70768	ILMN_1754600	54874	FNBP1L
GSE70768	ILMN_1696568	27032	ATP2C1
GSE70768	ILMN_2220518	10542	HBXIP
GSE70768	ILMN_2224486	57415	C3orf14
GSE70768	ILMN_1791483	5144	PDE4D
GSE70768	ILMN_1711878	58478	ENOPH1
GSE70768	ILMN_1730433	23607	CD2AP
GSE70768	ILMN_1677824	5867	RAB4A
GSE70768	ILMN_1652128	55788	LMBRD1
GSE70768	ILMN_2368773	10447	FAM3C
GSE70768	ILMN_1664466	55958	KLHL9
GSE70768	ILMN_1656129	57181	SLC39A10
GSE70768	ILMN_1766981	25972	UNC50
GSE70768	ILMN_2375879	7422	VEGFA
GSE70768	ILMN_1656913	4190	MDH1
GSE70768	ILMN_3290211	644761	LOC644761
GSE70768	ILMN_1803775	3336	HSPE1
GSE70768	ILMN_1669633	52	ACP1
GSE70768	ILMN_2072140	91408	BTF3L4
GSE70768	ILMN_1706094	25831	HECTD1
GSE70768	ILMN_2184184	301	ANXA1
GSE70768	ILMN_1655422	6139	RPL17
GSE70768	ILMN_1703697	10314	LANCL1
GSE70768	ILMN_1815051	8539	API5

GSE70768	ILMN_2409395	892	CCNC
GSE70768	ILMN_3260070	100128266	LOC100128266
GSE70768	ILMN_3272603	58516	FAM60A
GSE70768	ILMN_1657949	6009	RHEB
GSE70768	ILMN_2052598	83787	ARMC10
GSE70768	ILMN_1716071	152559	PAQR3
GSE70768	ILMN_2091347	3417	IDH1
GSE70768	ILMN_1812478	9326	ZNHIT3
GSE70768	ILMN_1803279	50999	TMED5
GSE70768	ILMN_1703791	310	ANXA7
GSE70768	ILMN_1750373	3730	KAL1
GSE70768	ILMN_1792587	9525	VPS4B
GSE70768	ILMN_1756525	27252	KLHL20
GSE70768	ILMN_1673543	55276	PGM2
GSE70768	ILMN_1704369	51474	LIMA1
GSE70768	ILMN_2362245	3188	HNRNPH2
GSE70768	ILMN_3244607	150864	FAM117B
GSE70768	ILMN_1773935	55858	TMEM165
GSE70768	ILMN_1801767	171586	ABHD3
GSE70768	ILMN_1798886	11051	NUDT21
GSE70768	ILMN_1665049	80208	SPG11
GSE70768	ILMN_1655329	55781	RIOK2
GSE70768	ILMN_1693259	10015	PDCD6IP
GSE70768	ILMN_1695386	5889	RAD51C
GSE70768	ILMN_1749009	25996	REXO2
GSE70768	ILMN_2045729	55759	WDR12
GSE70768	ILMN_2378100	26234	FBXL5
GSE70768	ILMN_1775573	286148	DPY19L4
GSE70768	ILMN_1703720	51639	SF3B14
GSE70768	ILMN_1658992	8813	DPM1
GSE70768	ILMN_1719518	378	ARF4
GSE70768	ILMN_1748591	4953	ODC1
GSE70768	ILMN_1727361	23592	LEMD3
GSE70768	ILMN_1796458	11345	GABARAPL2
GSE70768	ILMN_1725105	9521	EEF1E1
GSE70768	ILMN_1744023	145815	MGC18216
GSE70768	ILMN_1759453	7381	UQCRB
GSE70768	ILMN_2125374	55907	CMAS
GSE70768	ILMN_1678922	26091	HERC4
GSE70768	ILMN_1775448	5217	PFN2
GSE70768	ILMN_1679796	9804	TOMM20
GSE70768	ILMN_1730888	340252	ZNF680
GSE70768	ILMN_1682935	127018	LYPLAL1
GSE70768	ILMN_3247578	2195	FAT1
GSE70768	ILMN_1758623	3017	HIST1H2BD
GSE70768	ILMN_1708841	64083	GOLPH3
GSE70768	ILMN_1783333	56942	C16orf61
GSE70768	ILMN_1665280	28972	SPCS1
GSE70768	ILMN_2330861	10051	SMC4
GSE70768	ILMN_2143566	25800	SLC39A6
GSE70768	ILMN_1742230	11177	BAZ1A
GSE70768	ILMN_1726239	6902	TBCA
GSE70768	ILMN_1678766	6993	DYNLT1

GSE70768	ILMN_2206554	80255	SLC35F5
GSE70768	ILMN_1761456	79868	ALG13
GSE70768	ILMN_1666384	151579	LOC151579
GSE70768	ILMN_2191929	54942	C9orf6
GSE70768	ILMN_2307455	7319	UBE2A
GSE70768	ILMN_1788160	3799	KIF5B
GSE70768	ILMN_1677440	10159	ATP6AP2
GSE70768	ILMN_1746494	2339	FNTA
GSE70768	ILMN_1670970	5530	PPP3CA
GSE70768	ILMN_1807633	10247	HRSP12
GSE70768	ILMN_1730611	57142	RTN4
GSE70768	ILMN_1665887	80349	WDR61
GSE70768	ILMN_1797082	23161	SNX13
GSE70768	ILMN_1753342	6303	SAT1
GSE70768	ILMN_2187487	54497	HEATR5B
GSE70768	ILMN_2108339	55623	THUMPD1
GSE70768	ILMN_1741976	56916	SMARCAD1
GSE70768	ILMN_2168314	5238	PGM3
GSE70768	ILMN_1713901	11015	KDELR3
GSE70768	ILMN_3239621	11017	SNRNP27
GSE70768	ILMN_1754839	1665	DHX15
GSE70768	ILMN_2166865	56943	ENY2
GSE70768	ILMN_1790909	4780	NFE2L2
GSE70768	ILMN_2407082	9403	Selenoprotein 15
GSE70768	ILMN_1765060	55030	FBXO34
GSE70768	ILMN_1696494	54918	CMTM6
GSE70768	ILMN_1681340	51668	C1orf41
GSE70768	ILMN_2328776	51765	MST4
GSE70768	ILMN_1723212	6428	SFRS3
GSE70768	ILMN_2312275	6729	SRP54
GSE70768	ILMN_1693668	23063	KIAA0261
GSE70768	ILMN_1738346	221154	EFHA1
GSE70768	ILMN_1736054	10923	SUB1
GSE70768	ILMN_1662846	26996	GPR160
GSE70768	ILMN_3251560	153339	TMEM167A
GSE70768	ILMN_2343010	388962	BOLA3
GSE70768	ILMN_1798346	57614	KIAA1468
GSE70769	ILMN_1760849	81831	NETO2
GSE70769	ILMN_1763641	80110	ZNF614
GSE70769	ILMN_1808590	2982	GUCY1A3
GSE70769	ILMN_1729142	201161	CENPV
GSE70769	ILMN_1714737	25842	ASF1A
GSE70769	ILMN_1724139	114908	TMEM123
GSE70769	ILMN_1715886	29883	CNOT7
GSE70769	ILMN_2409395	892	CCNC
GSE70769	ILMN_1703408	7976	FZD3
GSE70769	ILMN_3246608	201161	CENPV
GSE70769	ILMN_1662846	26996	GPR160
GSE70769	ILMN_1784946	23595	ORC3L
GSE70769	ILMN_1662049	55326	AGPAT5
GSE70769	ILMN_1670172	55339	WDR33
GSE70769	ILMN_1656134	29883	CNOT7
GSE70769	ILMN_1654441	4942	OAT

GSE70769	ILMN_1811330	54463	FAM134B
GSE70769	ILMN_1774823	6164	RPL34
GSE70769	ILMN_1736054	10923	SUB1
GSE70769	ILMN_1744647	55832	CAND1
GSE70769	ILMN_1675542	729148	LOC729148
GSE70769	ILMN_3272603	58516	FAM60A
GSE70769	ILMN_1810176	6885	MAP3K7
GSE70769	ILMN_1758214	57038	RARS2
GSE70769	ILMN_2325347	8706	B3GALNT1
GSE70769	ILMN_2090558	27249	C2orf25
GSE70769	ILMN_1781097	23190	UBXN4
GSE70769	ILMN_2352563	56650	CLDND1
GSE70769	ILMN_1712748	51527	C14orf129
GSE70769	ILMN_3188984	441951	C20orf199
GSE70769	ILMN_1777564	4085	MAD2L1
GSE70769	ILMN_1757384	5901	RAN
GSE70769	ILMN_1736510	3344	FOXN2
GSE70769	ILMN_2255310	6210	RPS15A
GSE70769	ILMN_1676423	892	CCNC
GSE70769	ILMN_1767747	3066	HDAC2
GSE70769	ILMN_3229859	25843	MOBKL3
GSE70769	ILMN_1722156	112611	RWDD2A
GSE70769	ILMN_1710326	56650	CLDND1
GSE70769	ILMN_1697024	730432	LOC730432
GSE70769	ILMN_1812776	23219	FBXO28
GSE70769	ILMN_1707551	125061	AFMID
GSE70769	ILMN_1723212	6428	SFRS3
GSE70769	ILMN_1703132	57226	LYRM2
GSE70769	ILMN_1669881	27075	TSPAN13
GSE70769	ILMN_1800573	6227	RPS21
GSE70769	ILMN_1774028	9650	MTFR1
GSE70769	ILMN_1810759	27249	C2orf25
GSE70769	ILMN_2045911	23219	FBXO28
GSE70769	ILMN_1676448	57590	WDFY1
GSE70769	ILMN_1652024	1456	CSNK1G3
GSE70769	ILMN_2283597	54463	FAM134B
GSE70769	ILMN_3307930	5901	RAN
GSE70769	ILMN_1678766	6993	DYNLT1
GSE70769	ILMN_1657470	253943	YTHDF3
GSE70769	ILMN_2361570	57231	SNX14
GSE70769	ILMN_1729033	6133	RPL9
GSE70769	ILMN_2206554	80255	SLC35F5
GSE70769	ILMN_2380566	6477	SIAH1
GSE70769	ILMN_1726421	51108	METTL9
GSE70769	ILMN_1810719	54165	DCUN1D1
GSE70769	ILMN_2224031	1070	CETN3
GSE70769	ILMN_2330267	6059	ABCE1
GSE70769	ILMN_1722522	905	CCNT2
GSE70769	ILMN_1759359	91408	BTF3L4
GSE70769	ILMN_2197519	199692	ZNF627
GSE70769	ILMN_3306168	25843	MOBKL3
GSE70769	ILMN_1670420	10988	METAP2
GSE70769	ILMN_1676846	6059	ABCE1

GSE70769	ILMN_1727618	137682	C8orf38
GSE70769	ILMN_1703330	56929	FEM1C
GSE70769	ILMN_1680673	221294	NT5DC1
GSE70769	ILMN_3280847	651697	LOC651697
GSE70769	ILMN_1768050	60592	SCOC
GSE70769	ILMN_1677404	5911	RAP2A
GSE70769	ILMN_1796119	221302	ZUFSP
GSE70769	ILMN_1752631	8545	CGGBP1
GSE70769	ILMN_3237679	375743	PTAR1
GSE70769	ILMN_3251451	51003	MED31
GSE70769	ILMN_2148847	55122	AKIRIN2
GSE70769	ILMN_3260070	100128266	LOC100128266
GSE70769	ILMN_1759983	1810	DR1

log2 Fold Regulation	raw p value	pFDR
0,655081364	1,39E-06	7,10E-04
0,584318901	3,99E-07	3,12E-04
0,579356564	3,03E-04	0,017754718
0,57544362	1,03E-44	6,33E-41
0,547970214	3,22E-04	0,01850288
0,546921971	3,78E-07	3,12E-04
0,545838652	7,92E-05	0,008446266
0,53083549	2,74E-05	0,004310263
0,502608246	6,70E-04	0,025419647
0,472393767	2,50E-04	0,016404092
0,466812144	4,02E-04	0,020074913
0,464885861	1,68E-05	0,003556422
0,462411017	4,76E-04	0,021996983
0,459707895	3,96E-04	0,019953431
0,456861595	5,69E-04	0,023754937
0,454960037	3,86E-04	0,019743686
0,454309125	5,62E-04	0,023754937
0,4442706	1,19E-04	0,010414504
0,429672819	2,41E-04	0,016141477
0,426477488	5,01E-05	0,006837753
0,426219309	4,15E-04	0,020260029
0,42369559	5,24E-05	0,006996009
0,42057271	6,13E-04	0,02457455
0,413129205	3,68E-04	0,019484281
0,397893163	4,97E-04	0,02252774
0,389550342	3,01E-04	0,017754718
0,385410845	1,75E-04	0,013625307
0,379634294	2,11E-04	0,015539329
0,374610431	2,78E-04	0,017124331
0,374198723	5,75E-07	3,93E-04
0,371650854	0,00177202	0,045872284
0,371384624	6,24E-05	0,007669316
0,369491691	7,94E-06	0,0021285
0,367950664	1,70E-04	0,013572549
0,365651774	0,001547933	0,043229542
0,362919901	5,50E-06	0,001989516
0,361470301	0,001209035	0,036773826
0,353278132	8,06E-04	0,028120878
0,34971767	6,43E-06	0,002080867
0,347193376	2,23E-05	0,003906778
0,346251509	5,84E-05	0,007469598
0,344346501	3,61E-06	0,00147672
0,34371744	1,32E-05	0,003119137
0,34343741	7,42E-04	0,026489678
0,340622736	0,001935976	0,048748505
0,336191708	2,65E-05	0,004287068
0,333961819	3,25E-04	0,01850288
0,333132655	7,28E-05	0,008136516
0,33259502	1,09E-06	6,10E-04
0,330985567	0,001966213	0,04910737
0,326610891	2,60E-04	0,016840112

0,326349261	7,97E-05	0,008446266
0,326037606	0,001336677	0,038922004
2,418834038	3,24E-05	0,001970335
1,999079281	6,04E-04	0,012577491
1,94256871	0,001748884	0,024708788
1,922714588	3,94E-04	0,009664799
1,876413319	0,003383417	0,037943333
1,85812685	6,35E-04	0,013011861
1,790998943	0,001528166	0,022752499
1,593718288	0,001398883	0,021539802
1,577251057	0,001106995	0,018771646
1,558190275	4,15E-04	0,009972409
1,537597252	0,002316394	0,029701048
1,524669133	7,68E-04	0,014679326
1,471566596	0,003761196	0,040343791
1,431093023	8,84E-05	0,003654516
1,424524313	0,004597575	0,045621381
1,386573467	1,11E-04	0,004246399
1,381240486	0,002820966	0,033717389
1,320206131	4,22E-04	0,010048386
1,303082981	0,001477142	0,022274557
1,296180233	9,37E-04	0,016823018
1,262317125	0,004584898	0,045536162
1,244621564	1,76E-04	0,005735638
1,237441332	4,75E-04	0,01080474
1,210907505	0,002934518	0,03465408
1,19370296	0,002901126	0,034405688
1,189481501	0,001737696	0,024611739
1,189145877	0,001288367	0,020548027
1,182904334	0,002159213	0,028346591
1,182203488	0,00142177	0,021774239
1,176485729	6,09E-06	7,36E-04
1,176455603	7,07E-04	0,013892214
1,164625264	9,98E-04	0,017438748
1,16015222	4,92E-06	6,45E-04
1,157241543	3,34E-06	5,13E-04
1,156983615	0,004335127	0,044125511
1,152156977	3,20E-06	5,04E-04
1,126926533	0,003765934	0,04036327
1,125509514	0,001465351	0,022178925
1,120261628	0,001405738	0,02158469
1,119303383	0,003051426	0,035516784
1,115824524	7,41E-07	2,31E-04
1,108016913	0,004302625	0,044022306
1,10527537	0,002977879	0,034907008
1,102866808	5,66E-05	0,002769913
1,099254228	1,07E-06	2,71E-04
1,08872833	1,04E-04	0,004094156
1,082545455	0,003912477	0,041484741
1,081031712	1,37E-05	0,001164149
1,068864165	5,59E-06	7,01E-04
1,063328224	6,77E-05	0,003106515
1,056602537	0,002750472	0,033139581

1,051269556	5,75E-04	0,012208884
1,043084567	1,49E-04	0,005197203
1,037211945	3,96E-07	1,59E-04
1,0347426	3,88E-08	5,53E-05
1,034658034	1,70E-08	3,99E-05
1,033089323	0,001649699	0,02377568
1,030550211	3,09E-05	0,001926224
1,029939746	0,002359629	0,030056421
1,024249471	0,003046853	0,03550066
1,019031184	1,69E-06	3,54E-04
1,016922833	2,31E-04	0,006834019
1,015362051	6,26E-06	7,50E-04
1,010721459	3,96E-04	0,00969337
1,010551797	0,004155193	0,04322552
1,0097574	1,35E-04	0,004874403
1,009510571	1,60E-05	0,001271126
1,007606765	6,12E-05	0,002934596
1,007147992	0,001265121	0,020273251
1,001641121	2,58E-05	0,00173298
1,001598309	4,34E-04	0,010215739
0,99967389	0,003011692	0,035183003
0,99857611	2,70E-04	0,007602377
0,997704545	3,75E-12	1,67E-07
0,995406977	0,00461544	0,045669692
0,99429334	0,001168274	0,019338557
0,993884249	0,00374398	0,040227758
0,99367759	1,82E-04	0,005856129
0,992984144	5,52E-04	0,01197575
0,986332452	5,42E-06	6,87E-04
0,982867865	4,85E-07	1,80E-04
0,98066649	4,88E-04	0,011028096
0,976872093	3,84E-06	5,58E-04
0,976770613	4,00E-04	0,009757706
0,976629493	2,08E-06	3,84E-04
0,975559197	8,61E-05	0,003611058
0,974804968	1,65E-06	3,53E-04
0,974424947	1,96E-05	0,001474572
0,971721987	0,004942268	0,047806381
0,97114482	1,88E-06	3,66E-04
0,969188161	0,001509387	0,022592118
0,968891121	7,41E-04	0,014343636
0,968801797	0,003477565	0,03851823
0,96680444	8,26E-04	0,01538217
0,965330867	0,00156966	0,023120972
0,964357294	5,20E-07	1,87E-04
0,962972516	1,31E-05	0,001132287
0,96070666	6,94E-05	0,003163102
0,960684461	0,005245561	0,049682812
0,960534355	4,69E-08	5,81E-05
0,960385307	5,13E-04	0,011433521
0,9525074	6,77E-06	7,79E-04
0,950587738	0,001218412	0,019839004
0,950429175	5,98E-06	7,30E-04

0,948874736	6,40E-05	0,003012578
0,946059725	4,73E-09	1,91E-05
0,944592495	1,96E-04	0,006131281
0,944330338	2,39E-08	5,07E-05
0,942703488	0,001525018	0,022734408
0,938603594	3,31E-05	0,001989127
0,937939218	0,002505192	0,03127558
0,937413848	1,03E-04	0,00409329
0,936861522	0,003867219	0,041102541
0,93435148	0,001255575	0,020212623
0,933589323	4,28E-04	0,01014071
0,930272199	5,20E-06	6,68E-04
0,93002907	2,51E-04	0,007246812
0,927874736	9,03E-05	0,003700939
0,927552326	0,004204435	0,043475742
0,926221459	5,44E-04	0,011865083
0,92554334	0,003548508	0,038941796
0,923957717	1,05E-04	0,004105033
0,923062896	0,004962394	0,04790003
0,922229915	4,39E-06	5,98E-04
0,921929175	0,001528516	0,022752499
0,920614693	6,42E-04	0,013101756
0,919957188	0,002986986	0,034967748
0,919346723	6,57E-04	0,013299131
0,917005814	2,77E-05	0,001818956
0,916388478	8,09E-05	0,00347139
0,916090909	1,10E-04	0,004239262
0,915593023	3,36E-06	5,13E-04
0,913036469	0,001378169	0,021345322
0,912912791	3,40E-04	0,008720464
0,910795455	5,95E-07	2,07E-04
0,908098837	0,001236341	0,020027944
0,906877907	0,001176805	0,01943627
0,906776956	0,001150476	0,019172013
0,906049683	0,002256036	0,029238543
0,905537526	5,90E-05	0,002854743
0,905268499	5,95E-06	7,30E-04
0,904448732	7,39E-04	0,014323063
0,903925476	4,31E-06	5,93E-04
0,903272727	0,003200192	0,036573513
0,902875793	7,15E-05	0,003212301
0,902125264	7,81E-07	2,31E-04
0,900902748	0,001705589	0,024319388
0,899775899	0,002745801	0,033110172
0,899689218	2,22E-04	0,006659823
0,896319767	9,98E-07	2,62E-04
0,896048626	3,41E-04	0,008725273
0,895662791	2,20E-06	3,97E-04
0,895235201	1,70E-05	0,001322796
0,894441332	0,002643315	0,032391696
0,892684461	3,75E-04	0,009305519
0,88857981	8,51E-06	8,69E-04
0,885403805	0,002870183	0,034147824

0,882257928	5,64E-08	5,89E-05
0,881120507	1,40E-04	0,004976299
0,880664905	3,48E-11	7,75E-07
0,880516913	3,04E-05	0,001911084
0,880512156	0,001201251	0,019655014
0,877934461	2,00E-04	0,006203376
0,877911734	4,98E-04	0,011204493
0,877789112	5,44E-04	0,011865083
0,875899049	1,01E-06	2,63E-04
0,875707717	2,92E-05	0,001866567
0,875653805	0,003209581	0,036630181
0,875157505	8,24E-09	2,82E-05
0,872922833	2,13E-04	0,006483092
0,872108351	4,49E-08	5,81E-05
0,871005285	7,29E-04	0,014205812
0,870250529	5,10E-04	0,011390052
0,869882664	5,36E-05	0,002661235
0,866022199	0,003139162	0,036089559
0,865941332	7,35E-04	0,014261239
0,865530127	0,003456177	0,038392006
0,8639963	0,004162337	0,043260875
0,86397463	1,69E-04	0,005589448
0,861258985	4,04E-05	0,002279965
0,861183404	0,001052187	0,01809599
0,855928647	2,36E-04	0,006932556
0,855630021	4,51E-04	0,010460185
0,855469345	0,001481292	0,022306514
0,854582981	1,44E-07	1,01E-04
0,853616808	1,37E-06	3,17E-04
0,851964588	0,005276267	0,049909967
0,851360465	5,68E-06	7,08E-04
0,8510074	4,18E-04	0,009992372
0,849542283	0,003545189	0,038924547
0,849473573	4,79E-06	6,42E-04
0,847889535	2,78E-05	0,001821276
0,846187104	0,002485726	0,031102303
0,845951374	1,41E-04	0,004995481
0,845011099	2,63E-04	0,007462687
0,844998943	1,76E-07	1,02E-04
0,844519027	5,87E-05	0,002842762
0,844056025	1,60E-08	3,96E-05
0,843798626	4,14E-06	5,83E-04
0,842414905	1,26E-06	3,04E-04
0,841770085	0,003317302	0,037481244
0,841114693	4,84E-04	0,010955962
0,83977167	2,49E-07	1,19E-04
0,839752643	1,14E-07	9,12E-05
0,839290698	0,003269043	0,037156478
0,838565011	8,70E-06	8,78E-04
0,838489429	4,33E-06	5,93E-04
0,837914376	0,00336073	0,037755451
0,837469873	0,004874145	0,0472912
0,836860994	5,03E-04	0,011290289

0,836476744	2,43E-07	1,19E-04
0,834964059	7,52E-07	2,31E-04
0,834238901	0,002734676	0,033060917
0,834117865	9,72E-04	0,017142881
0,833511099	1,21E-04	0,004547059
0,832947146	3,46E-04	0,008818933
0,832563953	8,04E-05	0,003468463
0,831858879	1,79E-07	1,02E-04
0,83169186	4,51E-04	0,010463245
0,831213002	0,003843139	0,040934667
0,828645349	1,91E-04	0,006022449
0,828131607	1,62E-05	0,001281749
0,827269027	7,53E-06	8,23E-04
0,827050211	0,001649843	0,02377568
0,826692389	9,22E-06	9,13E-04
0,825541755	7,12E-04	0,013957257
0,825400106	4,76E-04	0,010812604
0,825100423	1,28E-08	3,80E-05
0,824437104	1,05E-04	0,004110075
0,824366279	1,90E-04	0,006012071
0,823976744	9,21E-05	0,003745886
0,822740486	0,001324851	0,020816289
0,822150634	0,001619368	0,023572924
0,821751586	1,85E-04	0,005918122
0,820189218	6,09E-04	0,012641815
0,820012156	5,31E-05	0,002650117
0,81982241	0,001200745	0,019655014
0,819662791	1,58E-06	3,42E-04
0,819586681	1,02E-05	9,68E-04
0,819381078	1,22E-04	0,004565261
0,819129493	1,11E-04	0,004239262
0,818010571	1,35E-04	0,004874403
0,817687632	5,00E-04	0,011237271
0,814481501	5,47E-09	2,03E-05
0,814199789	5,22E-05	0,002616381
0,813239958	1,82E-04	0,005856743
0,813019027	2,19E-04	0,006606841
0,812124736	5,18E-08	5,89E-05
0,812062896	2,58E-04	0,007391856
0,812000529	0,00440141	0,044497591
0,811813425	3,31E-04	0,008539003
0,811694503	0,0011085	0,018788828
0,811588266	4,31E-06	5,93E-04
0,810865222	1,41E-05	0,001185823
0,809951374	9,79E-06	9,52E-04
0,809268499	0,001403385	0,021570866
0,809146934	9,39E-08	8,64E-05
0,806513214	8,80E-06	8,83E-04
0,805987315	1,70E-04	0,00559584
0,80519556	3,84E-04	0,009461867
0,80480444	0,00178648	0,025021877
0,804617336	4,17E-04	0,009989073
0,803500529	2,46E-10	3,65E-06

0,803433932	4,22E-06	5,88E-04
0,802628436	0,001924037	0,026169255
0,802628436	7,27E-04	0,014179653
0,802607294	1,44E-07	1,01E-04
0,802417548	2,30E-04	0,006830579
0,802199789	7,56E-07	2,31E-04
0,802056554	0,00450608	0,045064623
0,801738901	9,87E-04	0,017336748
0,800715645	5,43E-04	0,011865083
0,800692389	1,93E-04	0,006065689
0,799715645	5,87E-07	2,06E-04
0,799561311	2,63E-04	0,007469042
0,799511099	5,77E-06	7,14E-04
0,798272727	0,004411467	0,044528522
0,797474101	7,68E-04	0,014681711
0,796315539	5,36E-04	0,011767563
0,796204545	0,004161231	0,043260875
0,794157505	6,09E-06	7,36E-04
0,79380074	1,97E-07	1,07E-04
0,793614165	0,001422482	0,021774239
0,792425476	3,51E-08	5,53E-05
0,792202431	2,00E-05	0,001493338
0,792088266	3,13E-04	0,008302485
0,791472516	8,90E-04	0,01618331
0,791365751	2,00E-09	1,49E-05
0,790682347	3,08E-08	5,49E-05
0,790567653	0,004336869	0,044125511
0,790262685	0,001226876	0,019931635
0,790091966	2,50E-06	4,31E-04
0,789930761	9,61E-05	0,003882573
0,78972093	3,60E-05	0,002087579
0,789658562	0,002179158	0,028499244
0,789424947	3,08E-05	0,001925126
0,78897833	0,003343013	0,037622834
0,788882135	3,65E-07	1,52E-04
0,78864852	0,001982993	0,02665372
0,788643235	1,71E-06	3,57E-04
0,7884963	0,001993908	0,026768121
0,788208774	1,59E-04	0,005386936
0,787395877	3,02E-06	4,84E-04
0,78641649	1,80E-04	0,005807147
0,785202431	1,87E-05	0,001417843
0,784747357	4,40E-04	0,010291188
0,784306025	8,78E-07	2,43E-04
0,784171247	4,82E-04	0,010938256
0,784078753	5,15E-06	6,63E-04
0,783790169	4,71E-04	0,010750193
0,783569767	1,05E-06	2,69E-04
0,783443446	8,42E-06	8,65E-04
0,782991543	0,003791669	0,040509447
0,782807082	0,002839471	0,033863823
0,782493129	2,20E-06	3,97E-04
0,782042812	1,20E-05	0,001066623

0,779263742	0,001532983	0,022776921
0,778671776	1,01E-04	0,004044624
0,77696723	3,23E-04	0,008446399
0,776937632	3,25E-05	0,00197257
0,776661734	2,75E-07	1,25E-04
0,776318182	3,67E-06	5,43E-04
0,775335624	3,64E-04	0,009105395
0,77512315	4,95E-07	1,80E-04
0,774588795	2,16E-05	0,001562278
0,774459302	1,24E-06	3,00E-04
0,774386892	6,42E-05	0,00301735
0,774330338	1,89E-06	0,000366552
0,774112051	0,002965258	0,034865552
0,773137421	2,17E-06	3,97E-04
0,773135835	7,51E-05	0,003316898
0,772218816	6,12E-04	0,012669117
0,771950846	6,68E-05	0,003086127
0,771873679	1,78E-07	1,02E-04
0,771559197	6,74E-05	0,003099929
0,770589852	0,00476529	0,046549363
0,770466702	0,001365287	0,021234403
0,770461945	2,49E-04	0,007205528
0,770145877	0,004714474	0,046262045
0,770004228	4,46E-05	0,002396026
0,769812368	7,28E-05	0,003256747
0,769693975	0,003089527	0,035717589
0,769387949	0,001873199	0,025713337
0,769076638	0,002198956	0,028690769
0,768792812	1,20E-04	0,004519639
0,768761099	0,001832264	0,025362448
0,768290698	5,77E-04	0,012217603
0,768234144	0,002192814	0,0286526
0,768169133	1,39E-04	0,004976299
0,768030127	1,59E-04	0,005386936
0,767766385	0,003950352	0,041767024
0,767591966	9,99E-04	0,017441169
0,76757241	0,001545613	0,022873018
0,767216702	1,04E-04	0,004094156
0,766268499	0,003771857	0,040390707
0,766107822	0,00217667	0,028478286
0,765954545	0,004236687	0,043644536
0,765646406	9,63E-07	2,59E-04
0,765262685	0,001016107	0,017645855
0,76511945	1,55E-04	0,00531987
0,765081924	0,002555004	0,031637634
0,764643763	2,98E-05	0,001883356
0,764500529	0,004632378	0,045762839
0,764490486	8,21E-05	0,003498171
0,764406977	0,002835677	0,033832436
0,764241015	0,001819458	0,02527157
0,764030655	4,67E-04	0,01068376
0,764006871	7,55E-04	0,014518574
0,763867336	0,00121945	0,019839004

0,763779598	3,63E-04	0,009093356
0,761910677	8,10E-05	0,00347139
0,761799154	1,17E-04	0,00442011
0,761132664	0,001222064	0,019867008
0,761117865	0,005228667	0,04957882
0,760089852	2,07E-05	0,001531483
0,759612579	2,53E-06	4,31E-04
0,759205074	8,36E-04	0,015491598
0,758977801	0,001377913	0,021345322
0,758540698	2,30E-07	1,19E-04
0,757349894	1,51E-05	0,001233839
0,7565037	2,09E-05	0,001543341
0,756353594	5,61E-05	0,002759165
0,756341438	2,89E-06	4,70E-04
0,756302854	0,001883039	0,025808638
0,756172833	8,20E-06	8,55E-04
0,756138478	4,13E-05	0,002295678
0,755798097	1,08E-06	2,71E-04
0,755786469	6,43E-04	0,013101756
0,755599366	3,29E-05	0,001984078
0,755515328	2,49E-07	1,19E-04
0,755414376	0,003707402	0,040024851
0,754817653	0,002717057	0,032968933
0,754485201	1,12E-06	2,75E-04
0,753694503	3,52E-04	0,00890974
0,75368129	0,001467953	0,022199084
0,753321353	8,36E-04	0,015491598
0,753301797	4,70E-05	0,002458825
0,753143235	7,83E-04	0,014836369
0,752965116	6,95E-04	0,013735308
0,75269186	9,63E-06	9,42E-04
0,752659619	7,91E-05	0,003429092
0,752486786	1,52E-06	3,40E-04
0,752442389	6,54E-06	7,62E-04
0,752409619	4,78E-05	0,002479468
0,75239482	1,79E-06	3,63E-04
0,752041755	5,89E-04	0,012377363
0,75202537	4,23E-04	0,010071272
0,751075053	0,005208935	0,049512338
0,750780655	9,77E-05	0,003939475
0,750471459	6,63E-05	0,003074296
0,750366279	6,93E-04	0,013724255
0,750110465	7,97E-04	0,015008697
0,749829281	0,002004749	0,026865085
0,749816068	1,68E-06	3,54E-04
0,749500529	0,002753867	0,03315805
0,749419133	3,47E-05	0,002050321
0,749204017	2,55E-07	1,21E-04
0,748941332	0,00209898	0,027760385
0,748936575	0,002312226	0,029673229
0,748896406	3,05E-04	0,008148423
0,747798626	9,89E-08	8,64E-05
0,747690803	0,001372872	0,021314731

0,747388478	6,50E-05	0,003042768
0,747367336	7,76E-05	0,003387102
0,747151691	2,31E-04	0,00683738
0,746524313	8,91E-07	2,43E-04
0,746289112	6,58E-04	0,013301696
0,745928647	0,001396758	0,02152844
0,745140592	9,09E-05	0,003714145
0,744899049	3,74E-04	0,009281936
0,743350423	1,33E-05	0,001151746
0,743222516	2,03E-06	3,79E-04
0,74282981	7,89E-07	2,31E-04
0,741433932	0,001249764	0,020141666
0,741337738	0,001669431	0,023941766
0,740296512	0,002342664	0,029921995
0,73884778	4,59E-08	5,81E-05
0,738819767	6,85E-06	7,85E-04
0,737385835	4,30E-07	1,68E-04
0,737061839	0,001318699	0,020768523
0,737056025	1,76E-04	0,005744748
0,736763742	6,73E-05	0,003097837
0,736448732	0,001894184	0,02590398
0,735879493	2,26E-05	0,001599003
0,735425476	0,004780518	0,046667411
0,734895877	0,003541036	0,038898126
0,734801268	4,08E-05	0,002285797
0,734439218	0,004694273	0,046159316
0,733988901	3,11E-09	1,54E-05
0,733622622	0,004365571	0,044326416
0,733179704	0,003519121	0,038714676
0,733147463	2,26E-05	0,001599003
0,732770613	9,11E-06	9,06E-04
0,732378964	5,36E-04	0,011768168
0,732145877	3,20E-05	0,001958212
0,732116279	2,41E-04	0,007041381
0,73192019	1,60E-04	0,005403379
0,731825053	7,82E-07	2,31E-04
0,730149577	0,001647132	0,023775293
0,729430761	1,90E-04	0,006020961
0,729259514	9,83E-06	9,54E-04
0,729037526	9,42E-04	0,016845832
0,728686047	0,00367233	0,039785485
0,728348309	4,41E-04	0,010299269
0,727894292	3,67E-04	0,009141185
0,727659091	6,49E-06	7,62E-04
0,727175476	0,002480183	0,031050391
0,72642759	1,79E-04	0,005801157
0,725352537	8,89E-07	2,43E-04
0,724975159	4,88E-07	1,80E-04
0,72387685	1,20E-07	9,25E-05
0,723802854	8,06E-04	0,01514285
0,723121564	0,001681201	0,024056353
0,72280444	0,001448633	0,022032446
0,722743658	5,62E-04	0,012058374

0,722651691	5,44E-04	0,011865083
0,721826638	0,003253988	0,037013703
0,721749471	5,60E-04	0,012055378
0,721396406	6,92E-06	7,89E-04
0,721309725	5,26E-05	0,002630461
0,721113636	1,67E-04	0,00554301
0,720694503	7,41E-06	8,19E-04
0,720693446	1,81E-07	1,02E-04
0,72057241	0,002659995	0,032495221
0,720367865	3,45E-05	0,002045664
0,72032981	3,17E-04	0,008324846
0,720087738	8,50E-05	0,003578629
0,719983087	1,24E-05	0,001094687
0,719633721	1,56E-06	3,40E-04
0,719270085	0,002519969	0,031374489
0,719261628	0,001745421	0,024685986
0,719074524	1,54E-05	0,001248099
0,718859408	0,002556106	0,031637634
0,71780814	1,04E-04	0,004094156
0,717797569	0,002525377	0,031418958
0,717305497	8,79E-04	0,016047938
0,716809197	2,64E-06	4,45E-04
0,716218288	1,95E-06	3,70E-04
0,716132135	6,59E-04	0,013308319
0,716071882	3,59E-04	0,009018869
0,714987844	4,29E-05	0,002349422
0,714966173	0,004070475	0,042707216
0,714436047	4,50E-05	0,00240429
0,713789112	2,64E-04	0,007477706
0,713763742	7,13E-04	0,013971714
0,713357822	7,59E-06	8,23E-04
0,713169133	1,00E-05	9,62E-04
0,712437632	2,37E-07	1,19E-04
0,712264271	1,56E-07	1,01E-04
0,712263214	0,002903164	0,034406175
0,712203488	9,37E-04	0,016823018
0,712189218	9,89E-04	0,017352697
0,712077696	1,53E-06	3,40E-04
0,711956131	5,37E-04	0,011777764
0,711673362	3,41E-04	0,008721042
0,711295455	0,003655966	0,039645877
0,710325053	8,08E-05	0,00347139
0,710164905	6,96E-05	0,003165786
0,710059197	6,12E-07	2,10E-04
0,70933351	0,003852598	0,041015801
0,708912262	0,002340095	0,029910243
0,708772727	5,69E-08	5,89E-05
0,708660677	2,80E-04	0,007732976
0,708599366	0,001238748	0,020036162
0,70835518	4,76E-05	0,002473781
0,70822833	0,00183752	0,025395741
0,708132135	0,001486033	0,022347692
0,707602537	2,47E-07	1,19E-04

0,707364693	6,60E-04	0,01331298
0,707162262	1,74E-05	0,001342792
0,706978858	4,02E-04	0,009780498
0,706821353	2,27E-05	0,001603585
0,706553911	1,88E-04	0,005994917
0,706278013	0,002129879	0,028069027
0,705766913	2,63E-04	0,007469042
0,705661734	2,69E-06	4,51E-04
0,705022727	4,79E-04	0,010873195
0,704738372	2,07E-04	0,006360711
0,704563425	0,003547771	0,038941796
0,703616279	0,001280173	0,020446045
0,702910677	3,24E-04	0,008463664
0,702860994	1,34E-04	0,004846643
0,702824524	2,50E-05	0,001699294
0,702056554	1,09E-06	2,71E-04
0,70172833	3,98E-08	5,53E-05
0,701679704	0,001847521	0,025492144
0,701196617	0,002065411	0,027454985
0,701173362	0,001453394	0,02205041
0,700852537	3,87E-06	5,58E-04
0,700727801	4,18E-04	0,009992372
0,700683932	4,46E-05	0,002396026
0,70055074	4,35E-05	0,002369416
0,700342495	1,48E-04	0,005184191
0,700338266	7,55E-05	0,003322643
0,700102537	0,002296116	0,029566639
0,700080338	0,003539082	0,038886253
0,700011628	1,17E-05	0,001051959
0,699343023	6,36E-04	0,013016024
0,699280655	1,02E-06	2,63E-04
0,699147463	9,73E-07	2,59E-04
0,698734672	9,71E-06	9,46E-04
0,698696617	0,0018497	0,025500784
0,698581395	5,41E-05	0,002681146
0,698460359	1,04E-04	0,004094156
0,698258985	3,42E-04	0,008746318
0,697990486	7,88E-06	8,32E-04
0,69729334	2,86E-05	0,001862274
0,69669186	5,50E-08	5,89E-05
0,696544926	1,93E-06	3,70E-04
0,696201374	1,01E-04	0,004050611
0,695791226	0,002070449	0,027513744
0,695761099	0,00268727	0,032732232
0,695651691	8,48E-04	0,015654901
0,695436047	5,07E-04	0,011358438
0,695043869	0,001647891	0,023775293
0,694464059	3,69E-04	0,009176939
0,694322939	3,59E-05	0,002087579
0,69415592	4,13E-05	0,002295678
0,694031712	6,73E-04	0,013500026
0,693068182	0,002966992	0,034865552
0,692434989	2,71E-04	0,007609606

0,692433932	8,26E-05	0,003512233
0,69212685	0,004025974	0,042365454
0,69197463	0,0023705	0,030134573
0,691811839	0,001473527	0,022234684
0,691807611	0,003083104	0,035671111
0,691574524	0,00336562	0,037800854
0,691011099	4,63E-06	6,25E-04
0,690984144	1,90E-07	1,05E-04
0,690766385	0,003469276	0,038466303
0,690115751	0,002302079	0,02962837
0,688977273	7,39E-05	0,003280167
0,688410677	2,78E-04	0,007702264
0,688195032	7,78E-06	8,29E-04
0,688164905	1,48E-08	3,87E-05
0,688143235	1,05E-04	0,004112788
0,687258985	2,51E-05	0,001700039
0,686878964	0,001322055	0,020787016
0,686693446	2,47E-05	0,001687449
0,686396934	8,82E-06	8,83E-04
0,685959831	0,002860231	0,034065813
0,685635835	7,43E-06	8,19E-04
0,685557082	2,77E-04	0,0076952
0,685062368	4,15E-05	0,002295678
0,684765328	9,14E-04	0,016525274
0,684738901	0,00170452	0,024311926
0,684621036	4,57E-04	0,010543218
0,684281184	4,35E-05	0,002369675
0,683126321	3,58E-04	0,009013238
0,683118393	9,41E-04	0,016833945
0,683021142	0,003806389	0,040630669
0,682760042	6,87E-05	0,003138992
0,682326638	2,11E-05	0,001543562
0,681882135	1,09E-05	0,001016299
0,681850423	0,001068042	0,018302682
0,681764271	0,00471924	0,046272024
0,681750529	7,25E-06	8,07E-04
0,6815074	1,73E-06	3,58E-04
0,681352008	9,57E-04	0,016986886
0,680820296	0,001311483	0,020730549
0,680730973	1,25E-04	0,004634753
0,680472516	0,000103196	0,00409329
0,680455603	0,003895765	0,041337059
0,680280127	1,19E-05	0,001063493
0,680276956	1,51E-07	1,01E-04
0,679273784	0,001189026	0,019565568
0,679225687	6,26E-06	7,50E-04
0,679045983	3,07E-04	0,008171005
0,679031184	3,17E-04	0,008324846
0,678784884	0,005122074	0,048966369
0,678721459	1,40E-06	3,22E-04
0,67862685	2,18E-05	0,001567122
0,677983615	1,08E-04	0,004173632
0,677730973	4,05E-05	0,002280817

0,677403805	2,43E-05	0,001671314
0,677102008	3,80E-08	5,53E-05
0,676680233	2,34E-05	0,001631936
0,676563425	8,53E-06	8,69E-04
0,676220402	7,05E-04	0,013880078
0,676158562	3,54E-05	0,00208053
0,676060254	1,62E-04	0,005441346
0,675638478	0,003306128	0,037456486
0,675553911	1,59E-04	0,005386936
0,675391121	3,06E-04	0,008148423
0,675162791	9,51E-04	0,016928334
0,674464059	0,00101528	0,017645195
0,674196089	7,02E-06	7,93E-04
0,673948203	2,34E-04	0,006879986
0,673337209	5,13E-04	0,011434536
0,673017442	1,32E-06	3,11E-04
0,672806554	0,002512486	0,031313984
0,672729915	0,001246333	0,020114724
0,672142178	0,003602635	0,039322658
0,671856237	0,003198814	0,036572893
0,671703488	4,17E-04	0,009992372
0,671566596	1,05E-05	9,98E-04
0,671332981	1,99E-04	0,006187029
0,670960888	2,50E-05	0,001699294
0,670736786	6,35E-04	0,013003699
0,670402748	0,001648215	0,023775293
0,670094609	0,002704482	0,032852047
0,669901163	4,71E-04	0,010750193
0,66962685	2,98E-05	0,001883356
0,669086681	2,84E-06	4,64E-04
0,669064482	0,001312359	0,020737036
0,668879493	0,00457792	0,045521965
0,667641649	2,65E-05	0,001761829
0,667489958	0,004252462	0,043766556
0,667167548	0,003335963	0,037590977
0,665910148	1,55E-05	0,001256155
0,665849366	1,13E-05	0,0010264
0,665727801	2,62E-05	0,001751934
0,665594609	0,003434121	0,038291557
0,66532981	2,36E-07	1,19E-04
0,665249471	3,69E-04	0,009176939
0,665159619	0,001912122	0,026062904
0,664725159	1,13E-05	0,0010264
0,663940275	1,36E-05	0,001161591
0,663639006	6,86E-04	0,01367164
0,663559197	7,31E-10	8,14E-06
0,66304704	8,30E-06	8,56E-04
0,662840381	3,52E-04	0,00890974
0,662438689	1,89E-04	0,006012071
0,661977801	0,001126506	0,0189374
0,661895877	1,13E-05	0,0010264
0,661883192	4,71E-05	0,002458825
0,661763742	0,001154971	0,019203819

0,661667548	0,001264391	0,020273251
0,661459302	2,36E-06	0,000411684
0,661278013	1,09E-06	2,71E-04
0,660692389	1,10E-07	9,12E-05
0,660381078	1,33E-04	0,004830239
0,660352008	8,54E-07	2,43E-04
0,65973203	1,50E-04	0,005235277
0,659524841	4,42E-04	0,010303274
0,659491543	6,49E-08	6,57E-05
0,659172833	6,84E-04	0,013647932
0,658353066	1,30E-05	0,001124924
0,658022199	1,03E-04	0,004094156
0,657881078	1,67E-05	0,001302373
0,657692389	6,93E-04	0,013724255
0,65709778	7,58E-04	0,014543309
0,656667019	1,96E-05	0,001477462
0,656442918	1,29E-04	0,004733924
0,656211416	5,10E-04	0,011387241
0,656073467	1,40E-05	0,001181122
0,655896934	0,004469468	0,04483964
0,65519186	2,35E-06	0,000411684
0,655052326	0,001464881	0,022178925
0,654717759	1,33E-04	0,00482787
0,654258457	5,42E-04	0,011844716
0,65421723	1,83E-05	0,001392836
0,654210359	0,001119268	0,018877957
0,653976216	5,35E-07	1,91E-04
0,65377167	0,003439397	0,038291557
0,653621564	2,07E-04	0,006360711
0,652792283	2,05E-04	0,006336057
0,652701903	0,001081949	0,018472949
0,65230074	1,71E-05	0,001323825
0,650576638	0,004204736	0,043475742
0,650488901	9,87E-04	0,017336748
0,650386892	0,001275191	0,020381088
0,64947833	2,43E-05	0,001673747
0,649030655	0,004261137	0,043795131
0,648924947	2,92E-05	0,001866567
0,648694503	1,47E-05	0,001223516
0,648376321	9,22E-05	0,003746071
0,648230444	9,87E-04	0,017336748
0,647853594	1,49E-05	0,001230225
0,647524313	8,67E-04	0,015897873
0,647436575	0,00211389	0,027907864
0,64719926	4,29E-04	0,010145058
0,647060782	3,14E-05	0,001938712
0,646928118	3,68E-06	5,43E-04
0,646765856	1,95E-06	3,70E-04
0,64640592	6,78E-06	7,79E-04
0,64615592	9,39E-04	0,016823018
0,646035941	0,001818189	0,025263805
0,645705603	3,38E-06	5,13E-04
0,645428118	0,00399266	0,04208449

0,645158034	4,85E-06	6,43E-04
0,645082981	3,42E-05	0,002032448
0,644959302	9,67E-04	0,017082322
0,644542812	6,87E-05	0,003138992
0,644513742	0,001286907	0,020538878
0,644301797	1,40E-04	0,004976299
0,644167548	0,001346643	0,021084307
0,644084038	6,57E-04	0,013299131
0,644048626	9,44E-06	9,29E-04
0,64404334	9,41E-04	0,016833945
0,642208245	9,45E-04	0,016855012
0,641963531	3,86E-06	5,58E-04
0,641658034	5,01E-05	0,002559682
0,641587738	4,92E-06	6,45E-04
0,641539112	0,004847663	0,047075279
0,641447674	0,001730694	0,024543788
0,641195032	1,70E-07	1,01E-04
0,640997357	2,92E-04	0,007884729
0,640436047	3,18E-07	1,35E-04
0,639878964	1,67E-07	1,01E-04
0,639873679	5,22E-04	0,011582547
0,639571882	7,68E-05	0,003364229
0,639570296	3,28E-08	5,53E-05
0,639457188	6,56E-06	7,62E-04
0,638966702	4,59E-04	0,010572932
0,638816596	1,40E-04	0,004993368
0,638774841	0,002999418	0,035088221
0,638721987	1,19E-04	0,004485192
0,638491015	6,27E-04	0,012889904
0,638010042	0,001152543	0,019177764
0,637701903	3,72E-06	5,47E-04
0,637654334	2,66E-06	4,47E-04
0,636913319	1,87E-06	3,66E-04
0,636874736	0,001772041	0,024905017
0,63669186	0,002408816	0,030482475
0,6365037	0,00419785	0,043455503
0,636213531	5,19E-04	0,011526964
0,636039641	0,002195604	0,028672035
0,635721459	8,66E-06	8,77E-04
0,635433404	2,40E-05	0,001663187
0,635177061	2,22E-04	0,006655488
0,635035412	0,001029985	0,017792285
0,633899577	8,14E-05	0,003483468
0,633788584	0,004728767	0,046324648
0,633576638	2,85E-04	0,007759204
0,633499471	9,45E-04	0,01685263
0,633141649	3,22E-05	0,001964194
0,633127907	0,001493735	0,022425666
0,632589323	0,001139752	0,01904781
0,631932875	2,54E-06	4,31E-04
0,631616808	2,80E-07	1,25E-04
0,631407505	4,30E-04	0,010160829
0,630711416	7,88E-04	0,014909159

0,630708774	5,77E-04	0,012214826
0,630119979	9,56E-04	0,016978558
0,630084038	4,62E-07	1,77E-04
0,629876321	1,48E-05	0,0012271
0,629618922	2,35E-05	0,001631936
0,629319767	7,92E-04	0,014958655
0,629162262	4,62E-05	0,002436301
0,628976216	8,53E-04	0,015714799
0,628955603	0,003144492	0,036137321
0,628406977	1,16E-07	9,12E-05
0,628243129	0,001949971	0,026401073
0,628180761	4,21E-06	5,88E-04
0,628132664	8,48E-04	0,015654901
0,628094609	1,06E-06	2,70E-04
0,628005814	4,55E-09	1,91E-05
0,62768129	1,90E-04	0,006012071
0,627614693	9,75E-08	8,64E-05
0,627547569	0,005060317	0,048568578
0,626630021	0,004718226	0,046272024
0,626591438	7,75E-06	8,29E-04
0,626330867	9,02E-05	0,003699381
0,626237315	5,93E-04	0,012403501
0,624792283	3,88E-07	1,57E-04
0,624758457	5,23E-04	0,011588541
0,62468129	8,93E-04	0,016211432
0,624590381	1,54E-04	0,005316662
0,624496829	8,27E-04	0,01538217
0,62427167	7,64E-06	8,26E-04
0,624195032	0,00489439	0,047456621
0,624020085	5,79E-05	0,002820726
0,623744715	3,41E-04	0,008721042
0,623734672	0,004231581	0,043619232
0,623616808	6,93E-04	0,013724255
0,623389006	3,64E-05	0,002104509
0,623087738	4,34E-04	0,010224501
0,623087738	0,001317927	0,020768119
0,622616279	4,30E-04	0,010160829
0,62232981	3,54E-06	5,29E-04
0,622058668	0,00443443	0,044650533
0,621981501	1,59E-05	0,001271059
0,621727801	7,04E-05	0,003188109
0,621506871	0,001317905	0,020768119
0,621341966	0,002330893	0,029818296
0,621318182	1,87E-05	0,001416753
0,621158562	1,39E-04	0,004976299
0,621149049	3,80E-04	0,009402704
0,620923362	0,00135678	0,021190891
0,620863636	6,92E-04	0,013724255
0,620658562	4,50E-06	6,10E-04
0,620132135	6,23E-05	0,002965203
0,620125793	0,004493628	0,044980714
0,619952431	0,003071149	0,035597515
0,619591966	1,79E-05	0,001375149

0,619367865	1,64E-05	0,001288965
0,619134249	2,02E-05	0,00150334
0,618976744	0,003060731	0,035587889
0,6187426	0,001787999	0,025021877
0,61872833	1,80E-04	0,005807147
0,618581924	0,001855993	0,025571453
0,61812315	6,15E-04	0,012697223
0,617983615	0,00176277	0,024809113
0,617823996	3,15E-04	0,008303463
0,617774841	1,15E-06	2,81E-04
0,617754228	2,78E-04	0,007702264
0,617752114	2,92E-04	0,007884729
0,617742072	7,46E-06	8,19E-04
0,617397463	2,53E-06	4,31E-04
0,617254757	1,65E-04	0,00550079
0,617112051	1,00E-04	0,004019932
0,616970402	7,99E-04	0,015038423
0,616950846	2,87E-04	0,007795518
0,616730973	0,001821503	0,025290141
0,616703488	1,42E-04	0,005027842
0,616653805	3,01E-04	0,008053731
0,616650634	0,001801933	0,025151746
0,615929175	0,00452112	0,045144306
0,615899577	0,004358243	0,044272196
0,615741015	8,59E-04	0,015785961
0,615676533	7,71E-04	0,014712821
0,615561839	1,12E-05	0,0010264
0,615310782	6,20E-05	0,00296404
0,615115222	2,91E-05	0,001866567
0,615114693	2,61E-04	0,007443233
0,615002643	0,002460663	0,030918979
0,614792812	0,003888955	0,041284469
0,614705603	1,09E-06	2,71E-04
0,614024313	2,39E-06	4,15E-04
0,613938161	4,44E-05	0,002392073
0,613918076	8,03E-04	0,015100538
0,613596723	4,35E-04	0,010245115
0,61352167	0,002406882	0,030475314
0,61259778	3,92E-05	0,002229831
0,612576638	2,82E-04	0,007759204
0,612164905	0,00280152	0,033582054
0,612125793	6,02E-07	2,08E-04
0,61190222	0,002542115	0,031549285
0,611810254	3,35E-05	0,001998978
0,611664905	6,29E-04	0,012908933
0,611060782	1,59E-05	0,001271059
0,610868393	0,002064548	0,027451704
0,610556025	0,001847373	0,025492144
0,610342495	6,86E-04	0,013664343
0,61029334	4,00E-06	5,73E-04
0,610182347	8,40E-05	0,003548585
0,609585624	1,51E-05	0,001233839
0,6095	0,001006316	0,01754809

0,609494186	0,001080415	0,018469462
0,609224101	3,10E-05	0,001926224
0,609063953	0,00240252	0,030437391
0,608735201	0,001629806	0,023647584
0,608317125	1,67E-05	0,001301871
0,608245243	6,48E-04	0,013175081
0,607845666	0,00119578	0,019619371
0,607845137	2,71E-08	5,25E-05
0,607454017	1,54E-04	0,005306474
0,607118393	0,002754238	0,03315805
0,60671723	8,75E-04	0,016007755
0,606192918	8,31E-04	0,015417975
0,605695032	2,37E-04	0,006954732
0,605651691	1,54E-04	0,005316662
0,605641121	3,85E-06	5,58E-04
0,605615222	0,002203235	0,028736201
0,60506871	9,90E-04	0,017368606
0,604873679	6,88E-04	0,013684083
0,604610465	3,93E-06	5,64E-04
0,604438689	7,47E-05	0,003306721
0,604125264	4,14E-05	0,002295678
0,603389535	9,17E-04	0,016572533
0,603330867	1,64E-04	0,005488906
0,602877907	5,44E-05	0,002687277
0,602824524	5,72E-06	7,12E-04
0,602726216	0,003337809	0,037592756
0,60244186	2,21E-06	3,97E-04
0,602299154	0,001194109	0,019605742
0,601936047	3,15E-04	0,008303463
0,601859937	1,80E-05	0,001380111
0,6017537	7,24E-04	0,014137988
0,601519027	5,27E-04	0,011631738
0,601491015	0,002154873	0,028323008
0,601391121	1,10E-05	0,001016299
0,601218816	1,70E-04	0,00559584
0,600743129	0,001604043	0,023426388
0,600730444	3,32E-04	0,008546688
0,600674419	0,001262756	0,020269621
0,600484144	5,29E-06	6,77E-04
0,600468288	0,00215892	0,028346591
0,600446617	1,50E-04	0,005234605
0,600075581	9,42E-04	0,016843106
0,59987315	0,002645193	0,032396337
0,599750529	2,10E-05	0,001543341
0,599737844	0,004462727	0,044822484
0,599356765	1,75E-05	0,001346581
0,599324524	0,001028926	0,017785208
0,599161205	5,86E-05	0,00283931
0,599121564	1,29E-05	0,001122176
0,599117336	0,003572274	0,039096655
0,598741543	1,91E-06	3,69E-04
0,598595666	5,56E-04	0,012019926
0,598434461	0,003299061	0,037392715

0,597973044	3,94E-04	0,009668762
0,597887949	0,001578563	0,023206436
0,597812896	8,49E-05	0,003578629
0,597715645	2,50E-04	0,007214493
0,597041755	0,0016357	0,023702212
0,596306025	2,23E-04	0,006680424
0,596289641	0,001247933	0,020125971
0,595631078	1,05E-04	0,004105519
0,595468288	0,00466396	0,045932222
0,595466702	6,56E-05	0,003057668
0,595358879	4,21E-04	0,010043152
0,59526797	5,63E-04	0,012069922
0,59519926	4,54E-05	0,002415327
0,595112051	0,0013683	0,021273836
0,595084567	7,71E-06	8,29E-04
0,594922833	2,59E-04	0,007409751
0,594921247	0,004174326	0,043320001
0,594807082	1,83E-04	0,005859569
0,594646934	7,50E-04	0,014454311
0,594359408	0,003636797	0,039511585
0,594321353	0,004197245	0,043455503
0,594038055	2,91E-04	0,007873587
0,59396723	2,39E-05	0,001656458
0,593964059	2,10E-05	0,001543341
0,593909619	3,16E-05	0,001949906
0,593789112	0,004770303	0,046588111
0,593680761	0,003633386	0,039511585
0,593665433	1,47E-04	0,005133223
0,593552854	7,07E-04	0,013892214
0,593426533	1,24E-06	3,00E-04
0,593196089	3,28E-05	0,001980465
0,593192918	7,15E-06	8,02E-04
0,592146406	0,001714667	0,024409086
0,592131078	6,26E-07	2,13E-04
0,591734144	1,25E-04	0,004630441
0,590863108	0,001192679	0,019596709
0,590710359	0,003076535	0,035627242
0,590502114	6,93E-04	0,013724255
0,590217759	0,002507999	0,031300605
0,5900037	7,48E-05	0,003306721
0,589717759	3,56E-05	0,002082716
0,58963055	5,12E-05	0,002587139
0,589472516	3,45E-04	0,008787377
0,589405391	7,36E-04	0,01428767
0,589364693	0,004325204	0,044057139
0,589036469	1,75E-06	3,58E-04
0,588770613	8,40E-05	0,003548585
0,588627378	2,90E-08	5,38E-05
0,588575581	0,001620727	0,023584994
0,588136364	1,10E-04	0,004213886
0,588087738	1,07E-05	0,001003929
0,588049154	2,73E-04	0,007630608
0,587957188	0,003467539	0,038466303

0,587938161	3,14E-04	0,008302485
0,587876321	4,24E-04	0,010084929
0,587852537	0,004489321	0,044957809
0,587486258	1,62E-05	0,001282689
0,587167019	1,43E-04	0,005059531
0,586713531	5,38E-04	0,011786355
0,586279598	2,86E-07	1,26E-04
0,585513742	0,004853002	0,047116849
0,585254757	3,01E-04	0,008040175
0,585086681	9,91E-07	2,61E-04
0,58502167	7,10E-05	0,003200508
0,58493129	0,001138865	0,019042637
0,584591438	4,26E-04	0,010096928
0,58452537	1,15E-04	0,004360192
0,584524313	4,28E-04	0,010139572
0,584226744	0,002747562	0,033122441
0,584214059	1,58E-04	0,00538196
0,584159091	6,15E-04	0,012697223
0,584126321	4,52E-04	0,010471704
0,584001586	7,31E-06	8,12E-04
0,583877378	0,002709418	0,03290303
0,583530655	3,43E-05	0,002037783
0,58342019	2,43E-05	0,001671314
0,583289641	4,31E-05	0,002354203
0,583211416	0,005205779	0,049502454
0,583118393	0,004131848	0,043102821
0,582000529	6,85E-05	0,003138365
0,581510571	0,004016133	0,042281876
0,580909091	3,59E-04	0,009017286
0,580772727	8,98E-05	0,003688279
0,580226216	0,003431603	0,038281319
0,580060254	0,001276894	0,020401
0,57990592	6,51E-05	0,003044664
0,579841438	1,28E-05	0,001116061
0,579533298	3,09E-05	0,001926224
0,579464059	6,72E-04	0,01349285
0,579103594	5,43E-06	6,87E-04
0,579094609	4,58E-05	0,002426352
0,579033298	3,57E-05	0,002082716
0,578972516	0,003763211	0,040343791
0,578810782	8,24E-04	0,015357341
0,578439746	0,001723362	0,024463167
0,578276427	9,52E-04	0,016928334
0,578147992	0,001243021	0,020075829
0,577890592	2,28E-05	0,001605602
0,577864693	8,28E-06	8,56E-04
0,577846723	1,29E-04	0,004734028
0,57751797	5,36E-05	0,002661235
0,577441332	2,14E-05	0,001558917
0,576797569	5,82E-04	0,012279129
0,576758985	0,003080586	0,035660507
0,576692918	0,003512531	0,038661921
0,57655444	0,001593995	0,023333191

0,576424419	4,34E-06	5,94E-04
0,576286998	7,71E-06	8,29E-04
0,576144292	0,004083322	0,042776926
0,575814482	3,59E-05	0,002087579
0,575763742	3,36E-04	0,008630289
0,575529598	7,25E-04	0,014150598
0,575518499	3,57E-04	0,00899617
0,575226216	0,002971592	0,034879204
0,574928647	0,001560956	0,02303227
0,574230444	6,00E-06	7,30E-04
0,574216702	1,83E-04	0,005859569
0,573700846	1,41E-04	0,005004974
0,573311311	0,002805356	0,033591879
0,573267442	4,99E-05	0,00255176
0,572865751	7,21E-05	0,003228251
0,572723573	6,13E-04	0,012669606
0,572563425	0,001103811	0,018761291
0,572561839	3,27E-04	0,008497118
0,572497886	0,001722555	0,024459517
0,572219873	4,43E-04	0,01031814
0,571942389	0,001261265	0,020260293
0,571643763	1,07E-05	0,001003929
0,571639535	3,40E-06	5,14E-04
0,571187632	8,07E-04	0,015146987
0,571015856	1,07E-05	0,001003929
0,570346195	1,33E-06	3,11E-04
0,570090381	8,04E-05	0,003468463
0,56940222	7,85E-04	0,01487155
0,569269556	0,004701687	0,046201618
0,56923203	2,84E-04	0,007759204
0,569128436	0,003437776	0,038291557
0,569110994	7,80E-05	0,00339366
0,568385835	4,39E-05	0,00237714
0,568361522	0,001091148	0,01857955
0,568085624	0,00373699	0,040191506
0,567887949	7,92E-05	0,003431754
0,567579281	0,00160677	0,023448548
0,567370507	6,13E-04	0,01267083
0,567107822	0,002717256	0,032968933
0,566935518	3,58E-04	0,009013238
0,566932347	9,52E-05	0,003860366
0,56665592	9,87E-05	0,003963071
0,566015328	0,004957557	0,047891869
0,565816596	3,48E-05	0,002050321
0,565642706	0,004785433	0,046684697
0,565436047	1,80E-06	3,63E-04
0,565412791	8,26E-05	0,003512233
0,56532611	7,78E-04	0,01478302
0,565325053	5,60E-04	0,012055378
0,565282241	1,59E-04	0,005386936
0,565151163	2,60E-04	0,007431766
0,564943975	0,00144749	0,022032446
0,564497886	5,80E-05	0,002820937

0,564403277	3,14E-04	0,008303463
0,563968816	8,27E-04	0,01538217
0,563928118	0,003565425	0,03905048
0,563817125	0,001085151	0,018512815
0,563644292	7,01E-05	0,003179541
0,563636892	0,002343182	0,029921995
0,563609408	0,001920455	0,026136488
0,563147992	0,003003262	0,035112418
0,563048626	2,51E-05	0,001699294
0,56265222	0,003502201	0,038614365
0,562613108	0,001242637	0,020075829
0,561966173	2,46E-04	0,007151821
0,561794926	0,001471899	0,022217642
0,56160148	0,003656278	0,039645877
0,561485201	0,001193216	0,019598301
0,561455074	2,83E-04	0,007759204
0,561381078	8,29E-04	0,015390494
0,561304968	3,58E-04	0,009013238
0,560968816	0,003036991	0,035404272
0,560915433	4,71E-05	0,002458825
0,560896406	1,87E-06	3,66E-04
0,560292283	4,88E-06	6,43E-04
0,560200317	3,05E-05	0,001915156
0,560172304	5,45E-04	0,011865083
0,559957188	0,001559616	0,023026699
0,559692918	5,58E-08	5,89E-05
0,559535941	0,004311769	0,044050124
0,559001586	2,63E-04	0,007462687
0,558500529	3,96E-04	0,009682712
0,558104123	3,38E-06	5,13E-04
0,55798203	0,002703107	0,032844303
0,557968816	0,002475793	0,031012437
0,557258457	1,07E-04	0,004151615
0,557113108	0,001219402	0,019839004
0,556635835	7,29E-07	2,30E-04
0,55642389	3,26E-06	5,07E-04
0,556397992	0,001186515	0,019538685
0,556350951	3,39E-05	0,002018935
0,556112579	0,001509289	0,022592118
0,555774841	3,98E-05	0,002254591
0,555348309	7,58E-04	0,014543309
0,55532981	0,003201721	0,036577959
0,555008985	3,56E-05	0,002082716
0,554906977	6,04E-04	0,012577491
0,554839852	0,001208388	0,019738337
0,554684461	5,66E-04	0,012110917
0,554531184	0,003976411	0,041962865
0,554297569	3,28E-04	0,008498288
0,554184461	3,31E-04	0,008539003
0,554063953	4,12E-04	0,009923209
0,553887421	5,35E-05	0,002661235
0,553692389	0,003863848	0,041091865
0,5532426	2,15E-05	0,00156106

0,552490486	1,34E-05	0,001153897
0,552234672	0,002510007	0,031300605
0,55194556	3,26E-05	0,00197257
0,551894292	2,15E-04	0,006519425
0,551400106	0,001972016	0,026586404
0,55117759	0,003103275	0,035792929
0,55110518	1,90E-04	0,006020961
0,550993129	7,83E-07	2,31E-04
0,550818182	9,33E-04	0,016780237
0,550608351	7,42E-05	0,003290333
0,550327696	0,001268837	0,020319038
0,549735201	2,77E-04	0,007698371
0,54965592	0,001629359	0,023647584
0,549628436	9,61E-04	0,017026974
0,549524841	1,59E-04	0,005386936
0,549302326	7,78E-04	0,01478302
0,54926797	0,00344713	0,038329744
0,549213002	1,26E-04	0,004665842
0,549066068	3,27E-05	0,00197571
0,548880021	0,003879073	0,041204846
0,54884778	9,54E-04	0,016952303
0,548828224	7,05E-07	2,29E-04
0,548828224	4,61E-05	0,002436301
0,548762156	6,59E-04	0,013308319
0,548745772	6,04E-04	0,012577491
0,54858351	0,001520221	0,02270849
0,548364693	9,80E-04	0,017256765
0,548157505	1,88E-06	3,66E-04
0,548154863	5,25E-05	0,002629351
0,547922833	6,99E-04	0,013800714
0,547114693	2,16E-05	0,001562278
0,547053383	5,61E-04	0,012058374
0,546857822	5,11E-05	0,002587139
0,546509514	4,90E-08	5,81E-05
0,546035941	5,55E-04	0,012019926
0,545910148	6,22E-05	0,002965203
0,545784355	0,00476019	0,046530153
0,545389535	2,32E-05	0,001622481
0,545265856	3,43E-04	0,008763443
0,545241015	2,73E-05	0,001793417
0,545126321	6,52E-06	7,62E-04
0,545049154	0,001337435	0,020969631
0,544379493	8,11E-08	7,86E-05
0,54432981	0,004428649	0,044644282
0,544207717	6,39E-05	0,003012578
0,544079281	0,001927912	0,026189966
0,543977273	5,33E-05	0,002654886
0,54379704	3,25E-04	0,008477337
0,543339852	0,001164407	0,019303069
0,543178647	7,97E-05	0,003448841
0,543137421	6,09E-04	0,012644217
0,542979915	0,001871684	0,025704006
0,542863636	8,33E-05	0,003528935

0,542628964	0,00235008	0,029984793
0,54247463	0,002662118	0,032496956
0,542345137	2,47E-04	0,007178612
0,542137949	0,001140974	0,019056447
0,542063425	7,77E-05	0,003387239
0,541893763	2,53E-05	0,001707921
0,541752643	1,13E-04	0,00431598
0,541298097	6,44E-05	0,003022293
0,541036998	0,00429921	0,044022306
0,540971987	1,85E-04	0,005918122
0,540832981	1,28E-04	0,004723652
0,54030814	0,001439335	0,021946324
0,540225687	6,43E-04	0,013101756
0,540134249	2,44E-04	0,007113591
0,540122093	1,74E-06	3,58E-04
0,540053383	7,75E-07	2,31E-04
0,539291226	6,06E-04	0,012606343
0,538741543	0,005237487	0,04962745
0,538619979	0,002628405	0,032271127
0,538356237	6,21E-05	0,00296404
0,538156977	5,66E-04	0,012117436
0,538080338	0,00251982	0,031374489
0,538077167	4,77E-04	0,010845514
0,538019556	1,04E-04	0,004103487
0,537956131	8,71E-04	0,015946919
0,53782611	0,001790359	0,025023466
0,537401163	1,56E-04	0,005322108
0,537327167	1,20E-04	0,004520192
0,537096195	6,58E-05	0,003063878
0,537063953	0,001823391	0,025302535
0,536905391	0,001893607	0,02590398
0,536796512	2,85E-04	0,007759204
0,536724101	0,002211241	0,028794261
0,536642178	9,64E-06	9,42E-04
0,536546512	1,98E-04	0,006169312
0,536372622	1,62E-04	0,005444242
0,536353066	0,001404583	0,021574398
0,536161205	0,001849639	0,025500784
0,535956131	0,003768105	0,040370532
0,535924947	1,60E-07	1,01E-04
0,535724101	8,58E-07	2,43E-04
0,535624207	1,36E-05	0,001163819
0,535348309	0,003744259	0,040227758
0,535316068	1,48E-05	0,0012271
0,535227273	3,12E-05	0,001929977
0,535039112	0,004574549	0,045521965
0,534994715	2,55E-04	0,007331463
0,534692389	8,66E-05	0,003620006
0,534623679	4,99E-04	0,011229795
0,533718816	2,47E-05	0,001685141
0,533590909	1,37E-05	0,001165126
0,533436575	0,001191798	0,019596692
0,533399577	0,001804843	0,025178493

0,53322093	0,001535347	0,02279192
0,533104651	4,43E-04	0,01031814
0,532484672	4,48E-04	0,010398045
0,532093023	1,32E-04	0,004810941
0,53197833	7,76E-04	0,014772651
0,531723573	0,004581424	0,045521965
0,531605708	5,45E-04	0,011865083
0,531284884	0,004953239	0,047881311
0,531053911	0,003029291	0,035342259
0,530943975	1,64E-05	0,001289977
0,530913848	1,72E-04	0,00564288
0,530798626	9,74E-04	0,017174477
0,53039852	2,35E-05	0,001631936
0,530393235	6,35E-04	0,013003699
0,530374207	0,001593873	0,023333191
0,530368922	0,001787956	0,025021877
0,530242072	0,001364287	0,021226268
0,530233087	0,001675916	0,023991307
0,530093552	6,72E-04	0,013489501
0,529958774	6,93E-04	0,013724255
0,529903277	0,001127044	0,0189374
0,529635835	1,02E-04	0,004052636
0,529625264	8,93E-04	0,016211432
0,52957241	1,02E-04	0,004052636
0,529275899	0,001807166	0,02518723
0,529225687	0,001749566	0,02470916
0,529153805	1,99E-04	0,006191578
0,528915433	0,004324344	0,044057139
0,528791755	0,002515912	0,031347911
0,528651691	2,02E-04	0,006264766
0,528586681	0,002360473	0,030058576
0,528450317	0,004349769	0,044216367
0,528338795	7,77E-04	0,014781399
0,52831871	7,33E-04	0,014242035
0,528002643	2,68E-04	0,007567586
0,527857822	0,001535528	0,02279192
0,527828753	1,80E-04	0,005807147
0,527675476	1,01E-05	9,66E-04
0,527619979	3,73E-07	1,54E-04
0,527375264	6,36E-04	0,013014301
0,527291755	0,002926261	0,034593257
0,526811311	0,002967298	0,034865552
0,526653277	0,003321176	0,037481244
0,526647463	9,70E-04	0,017116793
0,526638478	0,00226173	0,029273677
0,526408034	0,002818171	0,033709073
0,526008985	2,25E-05	0,001599003
0,525839323	1,26E-05	0,001108953
0,525762685	6,77E-04	0,013526406
0,525316596	7,61E-04	0,014591223
0,525156448	0,001807847	0,025188851
0,523483615	2,62E-04	0,007462687
0,523030655	2,24E-05	0,001599003

0,522915433	0,001197966	0,019647354
0,522788055	0,001152039	0,019176536
0,52264482	0,00263352	0,032316118
0,522567653	0,004199159	0,043458956
0,522484672	4,36E-04	0,010246217
0,522277484	4,68E-05	0,002454888
0,522223573	1,38E-04	0,004951133
0,52219926	3,19E-05	0,001953832
0,522154863	5,60E-05	0,002758858
0,521748943	4,06E-05	0,002284185
0,521328224	3,76E-04	0,009305519
0,521302326	0,002820309	0,033717389
0,521282241	1,93E-04	0,006057821
0,521263214	9,89E-06	9,56E-04
0,52120296	8,79E-04	0,016047938
0,521054968	1,46E-04	0,005110857
0,520858351	0,001657624	0,023842872
0,520680761	0,004604157	0,045625713
0,520592495	6,76E-04	0,013516663
0,520368393	0,001106572	0,018771646
0,520320825	0,001188997	0,019565568
0,520239958	0,001037654	0,017894407
0,520180761	2,59E-04	0,007418231
0,519424419	8,66E-05	0,003620006
0,518785412	1,66E-05	0,001297315
0,518610465	9,25E-06	9,14E-04
0,517980444	4,48E-05	0,002399541
0,517961416	4,61E-05	0,002436301
0,517832452	8,45E-05	0,003562854
0,517602537	0,003287793	0,037321978
0,517571882	0,001106956	0,018771646
0,517431818	0,002750275	0,033139581
0,517172833	0,004830791	0,047013934
0,51688055	1,66E-04	0,005525897
0,51672093	7,33E-04	0,014242035
0,516628436	0,002053411	0,027344442
0,516599894	0,002502223	0,031247278
0,516413848	9,47E-04	0,0168771
0,516176533	2,72E-05	0,001791338
0,515765328	0,004454979	0,044763926
0,515765328	3,65E-05	0,002106138
0,515708245	0,003783617	0,04044575
0,515609408	1,05E-04	0,004112788
0,51502167	6,30E-04	0,012925968
0,514834567	0,001249844	0,020141666
0,514592495	0,002459983	0,030918979
0,51420296	3,84E-05	0,002192026
0,51407981	0,002614119	0,032140027
0,513650106	8,02E-04	0,015080427
0,513006871	0,00208108	0,027630285
0,512830338	0,00228602	0,029472785
0,512735729	0,004836187	0,047028192
0,512715116	6,10E-04	0,012648831

0,512076638	4,17E-05	0,002301591
0,511944503	0,003830663	0,040830019
0,51170296	0,003693478	0,039942288
0,511466702	8,55E-06	8,69E-04
0,511343552	0,005004031	0,048204918
0,511332981	0,003997042	0,042112182
0,511275899	8,83E-04	0,016091292
0,511183932	2,47E-04	0,007178612
0,51118129	8,85E-04	0,016119037
0,511122622	0,001863808	0,025623914
0,510963002	6,59E-05	0,003063878
0,510819239	6,44E-04	0,013101756
0,510690803	0,00230648	0,029667872
0,510647992	4,17E-04	0,009989073
0,510412791	0,001360522	0,021217735
0,510328224	2,89E-05	0,001866567
0,510262156	6,39E-05	0,003012578
0,509692389	3,76E-04	0,009305519
0,509536998	6,11E-04	0,012648831
0,50944186	7,83E-06	8,29E-04
0,509402748	0,002905346	0,034409927
0,50908351	7,99E-04	0,015038423
0,508809725	1,82E-06	3,63E-04
0,508777484	5,61E-06	7,01E-04
0,508675476	0,004653487	0,045879795
0,50867019	0,004928599	0,047725977
0,508650106	2,82E-04	0,007759204
0,508465116	5,56E-04	0,012019926
0,50827907	0,00226384	0,029284787
0,508243129	3,44E-04	0,008779164
0,508219345	1,64E-04	0,005488906
0,508206131	0,000283411	0,007759204
0,508192918	1,09E-04	0,004200837
0,508035412	0,004871301	0,047273906
0,507819767	0,002092461	0,027698834
0,507679175	0,004121109	0,043051284
0,507251586	6,67E-05	0,003086127
0,507144292	0,002293959	0,02955694
0,506849894	1,33E-04	0,00482787
0,506516385	0,003423099	0,038253518
0,506391121	1,34E-04	0,004860744
0,506365222	0,003483186	0,038527525
0,506044397	2,75E-04	0,007651372
0,505972516	6,02E-05	0,002904001
0,50589482	2,16E-05	0,001562654
0,505803911	2,69E-04	0,007582082
0,505626321	0,00171941	0,02445007
0,50527907	0,004811376	0,046896705
0,504885835	1,68E-04	0,005570375
0,504529598	0,002976896	0,034904679
0,503633721	0,001877571	0,025754265
0,503509514	0,004378291	0,044351479
0,503156448	0,001026673	0,017760055

0,503023256	4,72E-04	0,010752547
0,502746829	0,00182193	0,025290141
0,502506871	3,14E-04	0,008303463
0,502439746	6,36E-04	0,013016024
0,502348309	2,61E-04	0,007443233
0,501676004	1,03E-06	2,66E-04
0,501671247	2,09E-04	0,006402118
0,501550211	2,72E-06	4,53E-04
0,501452431	1,89E-04	0,006012071
0,50127537	7,10E-04	0,013932253
0,501223573	0,002050943	0,027319742
0,501144292	5,11E-05	0,002587139
0,500995243	0,002289183	0,029505031
0,50082981	0,0019782	0,026629479
0,500785412	0,001342965	0,02103412
0,500317653	0,001109787	0,018793982
0,500285941	0,001269028	0,020319038
0,499980973	0,002017269	0,026951785
0,499944503	3,50E-04	0,008889481
0,499935518	2,16E-04	0,006532318
0,499932875	0,004399353	0,044486892
0,499928118	3,60E-05	0,002087579
0,499899577	0,002163802	0,028365037
0,499831924	5,71E-04	0,012176757
0,499571353	4,15E-05	0,002295678
0,499512685	1,40E-04	0,004993368
0,499451374	0,001068314	0,018302682
0,499353594	0,002634848	0,032323515
0,499274313	0,00177238	0,024905017
0,499190275	0,002132619	0,028096836
0,499176533	0,004881279	0,047350108
0,499128964	2,92E-05	0,001866567
0,499036469	0,003448031	0,038330193
0,498910677	1,27E-04	0,004691206
0,498286469	0,001199285	0,019652056
0,498131607	1,52E-04	0,005270661
0,498099894	0,004526446	0,04517724
0,498011099	0,003443353	0,038326015
0,497787526	0,003225387	0,036754061
0,497723573	3,64E-04	0,009109573
0,497624207	3,15E-04	0,008303463
0,496913848	1,56E-04	0,005322108
0,496912791	5,82E-04	0,012283556
0,49621723	0,001650977	0,023776637
0,496133721	9,92E-04	0,017380519
0,495113636	0,001531372	0,022768164
0,494732558	0,003761408	0,040343791
0,494529598	0,001034831	0,017852642
0,494165962	0,001789856	0,025023466
0,49398203	1,39E-05	0,001176188
0,493803911	4,61E-04	0,010594032
0,493694503	3,15E-04	0,008303463
0,493635307	5,97E-04	0,012482124

0,493375264	0,003408757	0,038160254
0,493050211	7,98E-05	0,003448841
0,492907505	0,00290268	0,034406175
0,492455074	0,003736659	0,040191506
0,492433404	0,001593497	0,023333191
0,492310254	8,49E-04	0,015658245
0,49213055	0,001900075	0,025945864
0,491424947	0,004058218	0,042624209
0,49127907	0,004074293	0,042722531
0,4912537	0,002520158	0,031374489
0,490751586	9,44E-04	0,01685263
0,490612051	0,003857813	0,041061512
0,490550211	0,001236355	0,020027944
0,490327696	6,94E-07	2,29E-04
0,49023203	7,54E-04	0,014518574
0,490147992	0,001967691	0,026560245
0,490095666	2,07E-04	0,006363819
0,489985201	7,17E-04	0,014043587
0,489650634	7,71E-04	0,014712821
0,489587209	1,56E-04	0,005322108
0,48938055	9,84E-05	0,003958507
0,48927167	0,001545226	0,022873018
0,489058668	0,003310093	0,037458868
0,48905444	0,00363666	0,039511585
0,488847252	0,001132896	0,018991869
0,488751057	3,58E-05	0,002087579
0,488466702	5,07E-04	0,011347845
0,488364165	0,001574907	0,023179586
0,488235729	2,90E-05	0,001866567
0,4879963	6,46E-06	7,62E-04
0,487986786	0,001045997	0,018010392
0,487827696	5,50E-04	0,01196545
0,487610994	1,72E-04	0,005654961
0,487425476	0,003408503	0,038160254
0,487405391	6,37E-05	0,00301116
0,487393235	0,004374182	0,044340892
0,487325581	9,01E-06	9,00E-04
0,486902748	5,31E-04	0,011700411
0,486896406	3,78E-05	0,002168435
0,486716173	2,83E-04	0,007759204
0,486546512	4,01E-04	0,009759681
0,486515856	0,004617971	0,045669692
0,486276427	0,00350452	0,038620723
0,486201374	0,002526922	0,031418958
0,486195032	0,00421455	0,04353839
0,486073467	0,001256139	0,0202144
0,485790698	0,002508834	0,031300605
0,485567125	0,002541544	0,031549285
0,485374207	0,005159429	0,049204246
0,485179704	4,57E-04	0,010543218
0,484720402	2,17E-05	0,001563442
0,484619979	6,92E-04	0,013724255
0,484613636	5,17E-04	0,011502768

0,484256342	2,83E-04	0,007759204
0,483635835	0,004959337	0,047898676
0,483547569	0,001318056	0,020768119
0,483057082	8,13E-04	0,015220961
0,4827463	1,11E-04	0,004255047
0,482424419	0,003813218	0,040693817
0,482358879	0,002189601	0,02862741
0,481628436	1,64E-05	0,001289977
0,481362051	0,003379688	0,037911066
0,480853594	3,26E-04	0,008477337
0,48079334	0,003064358	0,035591387
0,480715645	0,004451932	0,044754424
0,480165433	0,001228839	0,019940761
0,479957188	0,004516227	0,045135924
0,47981871	3,66E-04	0,009126551
0,479748943	1,68E-04	0,005583663
0,479369979	6,15E-04	0,012692396
0,479331924	0,001957757	0,026490373
0,47880444	7,03E-06	7,93E-04
0,478743658	7,56E-04	0,014524687
0,478663319	2,73E-04	0,00762624
0,478628436	9,24E-04	0,016669981
0,478539112	0,003918376	0,041497892
0,478460888	0,004670551	0,045976804
0,478280655	0,001327349	0,020848172
0,478040698	0,001844933	0,025474483
0,477912791	0,002283988	0,029463638
0,477825053	2,27E-04	0,00674015
0,477767442	0,001272219	0,020355511
0,477762685	1,71E-04	0,005640263
0,477278013	8,57E-04	0,015754112
0,477150106	5,51E-04	0,01196545
0,476567125	1,59E-05	0,001271059
0,476377907	6,69E-04	0,01346176
0,47609408	3,69E-04	0,009176939
0,475979915	1,50E-05	0,0012336
0,475419133	2,38E-04	0,00697858
0,475205074	3,48E-04	0,008859432
0,47480444	1,16E-04	0,004404682
0,474587209	0,004122527	0,043056004
0,474176533	0,003388501	0,037990783
0,473776956	0,002932105	0,034634759
0,473508457	5,78E-04	0,012227872
0,473504757	0,002719286	0,032968933
0,473417019	0,003320446	0,037481244
0,472223573	6,68E-05	0,003086127
0,472202431	6,86E-05	0,003138992
0,472115751	0,001174384	0,019403475
0,472068182	0,001400397	0,021539802
0,472007928	4,10E-04	0,009884298
0,471987315	0,003561958	0,039036452
0,471904863	0,001451307	0,022038492
0,471725687	9,78E-05	0,003939475

0,471716173	4,40E-04	0,010291188
0,471705603	0,003864355	0,041091865
0,471433932	4,78E-05	0,002479468
0,471384249	9,45E-04	0,01685263
0,471136364	2,30E-04	0,006830579
0,471002643	0,001664002	0,023894678
0,470961945	0,0046345	0,045763928
0,470812368	0,003478781	0,03851823
0,470189746	7,20E-05	0,003227737
0,469670719	0,003505379	0,038620723
0,469193446	2,59E-04	0,007407658
0,469050211	2,09E-04	0,006402118
0,468768499	2,25E-04	0,006704079
0,468323996	5,14E-04	0,011451009
0,46829334	6,20E-05	0,00296404
0,468233615	2,25E-04	0,006711698
0,467932347	0,002223204	0,028905552
0,467927061	9,66E-04	0,017066695
0,467598309	0,003075631	0,035627242
0,467573996	4,06E-04	0,009842211
0,467520613	3,35E-04	0,008605302
0,467428118	4,05E-04	0,009832897
0,467409091	0,002016958	0,026951785
0,467360994	0,001022747	0,01772656
0,467210888	1,78E-04	0,005792816
0,467	0,004612593	0,045668668
0,46668129	0,004312209	0,044050124
0,466672304	0,002690681	0,032755865
0,466637949	4,11E-04	0,009914046
0,466489958	7,49E-04	0,014450483
0,465994715	4,58E-04	0,010561152
0,465755285	0,003426648	0,038263473
0,465539641	2,66E-05	0,001764845
0,465510042	7,21E-06	8,05E-04
0,465318182	0,002677185	0,032636157
0,464659091	0,002422132	0,030600633
0,464417019	0,004155255	0,04322552
0,464013742	0,00115865	0,019250152
0,463965116	0,003480057	0,038522781
0,463786998	0,001712974	0,024401247
0,463738372	9,13E-05	0,003726602
0,463531184	0,00476013	0,046530153
0,463033827	3,17E-05	0,001951337
0,462455074	8,63E-05	0,003616575
0,462039641	0,004022262	0,042336396
0,461797569	0,001469378	0,022199283
0,461631078	0,001639332	0,023727325
0,46125	5,88E-04	0,012359415
0,461046512	0,003705321	0,040012979
0,460575053	0,003076913	0,035627242
0,460523256	3,11E-04	0,00823789
0,460230444	0,001011807	0,01759856
0,459943975	0,003970325	0,041908571

0,459900634	1,26E-04	0,004674132
0,459895349	1,20E-05	0,001068665
0,459883192	2,69E-04	0,007582082
0,459878964	6,65E-06	7,68E-04
0,459731501	8,35E-05	0,003533845
0,459610465	3,22E-05	0,001964194
0,459570296	0,003717892	0,040089514
0,459326638	0,001669108	0,023941766
0,45929704	1,40E-04	0,004993368
0,459280655	0,001748985	0,024708788
0,459277484	2,07E-04	0,006360711
0,459275899	6,43E-04	0,013101756
0,459222516	0,00330721	0,037456486
0,458690803	0,004608188	0,045637477
0,458352537	7,39E-04	0,014323063
0,45773203	5,55E-04	0,012019926
0,457591438	0,00472068	0,046275959
0,457573467	8,24E-04	0,015357341
0,456845137	6,10E-04	0,012648831
0,456815539	0,00442612	0,044644282
0,456108351	0,001517153	0,022677879
0,456063953	1,70E-04	0,00559584
0,455821882	0,001852543	0,025532084
0,455787526	0,00237395	0,030169828
0,45568129	4,58E-04	0,010561152
0,455408034	0,004575905	0,045521965
0,455378436	0,001061898	0,018241883
0,455169133	9,27E-04	0,016711341
0,454581395	0,001958385	0,026490833
0,454365751	0,004313637	0,044050124
0,454106765	0,001570154	0,023120972
0,45382611	0,002247359	0,029134564
0,453587209	0,003481856	0,038527525
0,453078224	5,81E-04	0,012276824
0,453008985	1,69E-04	0,00559584
0,452591966	1,64E-04	0,005496793
0,45257611	0,001483136	0,022319186
0,452541226	7,79E-04	0,01479303
0,452528541	4,96E-05	0,002550636
0,452345137	0,002600356	0,032014999
0,451890592	7,50E-07	2,31E-04
0,451606765	0,002096574	0,027736798
0,451075581	8,23E-04	0,015353242
0,450697146	0,002425854	0,030619749
0,450244186	8,74E-05	0,0036389
0,450088266	1,89E-04	0,005994917
0,44897093	0,00516079	0,049204246
0,448911734	0,003683459	0,039882355
0,448848309	0,002924977	0,034587252
0,448742072	0,003669822	0,039773367
0,448429175	0,00285315	0,034008759
0,447502114	5,08E-05	0,002582144
0,447026427	0,001439243	0,021946324

0,446963002	8,07E-04	0,015150346
0,446959831	0,00107679	0,018422534
0,446776956	0,003916749	0,041490525
0,446505285	0,004465298	0,044829065
0,446435518	0,005084632	0,048749053
0,446421247	0,004814862	0,04690304
0,446298097	0,001448818	0,022032446
0,446104651	0,002876776	0,034207985
0,44610148	8,52E-06	8,69E-04
0,445903805	0,001856972	0,025571453
0,445544926	4,15E-04	0,009972409
0,445393763	1,51E-04	0,005259016
0,445202431	8,69E-04	0,015926312
0,445195032	1,21E-04	0,004533065
0,444663848	0,003151036	0,036175196
0,444570296	0,001126415	0,0189374
0,444533298	6,60E-04	0,01331298
0,444340909	0,003019959	0,035251852
0,443946617	6,55E-04	0,013281876
0,443650634	0,001487084	0,022355946
0,442457188	0,003447049	0,038329744
0,44226797	0,003087901	0,035717338
0,441975159	0,002613581	0,032140027
0,441884249	0,002910555	0,034462456
0,441483087	1,33E-04	0,004833434
0,441294926	0,002974604	0,034896173
0,441286998	0,002428929	0,0306396
0,441211416	3,25E-04	0,008477337
0,441195032	0,00261971	0,032190997
0,440614693	7,75E-05	0,003382553
0,44004334	2,50E-06	4,31E-04
0,43987685	0,004805063	0,046855675
0,4394926	0,005166812	0,04924058
0,43914852	0,004181978	0,043345768
0,438724101	1,78E-04	0,005792816
0,438323467	7,40E-04	0,014323063
0,437795455	2,34E-04	0,006890111
0,4374963	1,08E-04	0,00418342
0,437403277	2,24E-04	0,006689908
0,436778013	2,91E-05	0,001866567
0,43667389	8,84E-04	0,016110334
0,436614165	0,003051306	0,035516784
0,436346195	0,002985373	0,034967748
0,436249471	1,44E-04	0,005062162
0,435822939	4,25E-04	0,010090913
0,435763214	5,62E-04	0,012058374
0,435437632	1,40E-04	0,004976299
0,435372093	4,07E-04	0,009842211
0,435231501	0,002971366	0,034879204
0,433535941	0,005231242	0,04957882
0,433429704	0,002075412	0,027571474
0,433272727	2,01E-04	0,00621794
0,433188689	0,003419747	0,038225654

0,433002114	4,44E-05	0,002391693
0,432868393	5,25E-04	0,011622432
0,432426533	3,30E-04	0,008527772
0,432198203	0,001522478	0,022727947
0,431801797	0,003772121	0,040390707
0,431158562	0,001114335	0,018816132
0,431049683	1,91E-04	0,006022449
0,430288055	5,10E-04	0,011387241
0,43009408	0,002959744	0,03483192
0,429792283	1,83E-04	0,005869493
0,429491543	0,002958819	0,034830245
0,429293869	0,001988418	0,026702468
0,429236258	1,35E-04	0,004874403
0,428881607	0,004379001	0,044351479
0,428850423	0,0013942	0,021516466
0,428513742	1,78E-05	0,001369277
0,428048097	0,001506311	0,022568821
0,427771142	0,002884859	0,034261687
0,427525899	0,001110914	0,018793982
0,42723203	4,07E-04	0,009842211
0,426877907	0,001944079	0,026353335
0,426866808	0,002229323	0,028949002
0,426754757	3,08E-04	0,008180522
0,426720402	0,004280776	0,04395641
0,425716702	0,002104042	0,027810811
0,425465116	0,001561028	0,02303227
0,4252426	5,02E-05	0,002559682
0,425227801	7,56E-04	0,014524687
0,425088266	0,003050702	0,035516784
0,424835095	4,04E-04	0,009811395
0,424683932	0,003070344	0,035597445
0,424676533	0,00374904	0,040269407
0,424029598	0,004608418	0,045637477
0,423462474	0,004815414	0,04690304
0,423089323	0,001088035	0,018549713
0,422837738	3,76E-04	0,009305519
0,422291226	0,004622889	0,045689369
0,42210518	7,74E-04	0,014754587
0,422029598	0,001293857	0,02061018
0,421827167	0,004440818	0,044693128
0,421534355	0,004833668	0,047021382
0,421292283	0,00121349	0,019803777
0,421182347	0,001049094	0,018056734
0,421078224	9,59E-04	0,017004371
0,421068182	0,002858758	0,034057378
0,420775899	3,93E-04	0,009656577
0,420215116	0,001650768	0,023776637
0,419772199	6,61E-05	0,003069979
0,419718288	3,94E-04	0,009664799
0,419673362	0,003014368	0,035205031
0,419238372	0,003717022	0,040089514
0,419223573	3,28E-04	0,008506843
0,419082452	0,004117921	0,043028072

0,419009514	1,26E-04	0,00466353
0,418725159	0,001178627	0,019459137
0,418704017	0,003946785	0,041749129
0,418419133	1,53E-05	0,00124424
0,41822463	0,003206487	0,036604245
0,418186047	6,84E-04	0,013647932
0,418143235	0,002158479	0,028346591
0,418012156	4,48E-05	0,00239942
0,417975159	0,004896831	0,047464925
0,417745243	5,12E-05	0,002587139
0,417516913	0,001358168	0,0211977
0,417374207	3,28E-04	0,008505116
0,417012685	0,005283271	0,049943673
0,416710888	0,001985988	0,026677878
0,416593023	1,16E-05	0,001044039
0,416124736	0,001490406	0,02239078
0,416113636	0,001381372	0,021365219
0,416096723	1,99E-04	0,006185713
0,415816068	0,003906099	0,04143684
0,414963531	0,001913038	0,026067408
0,414469345	0,003577503	0,039144258
0,414425476	2,25E-06	4,03E-04
0,414421776	0,003789727	0,040501347
0,413791755	1,43E-04	0,005059531
0,413498943	0,002055458	0,027355335
0,412955603	0,001296152	0,020634662
0,412537526	0,001446704	0,022032446
0,412003171	0,00289855	0,03438429
0,41176797	0,001201906	0,019655014
0,411763214	0,001895006	0,02590398
0,411563953	0,003512478	0,038661921
0,410761099	0,001427639	0,021823185
0,410323996	0,00115298	0,01917788
0,410266913	0,001416213	0,021715596
0,410212474	7,52E-04	0,014499707
0,410192918	2,21E-05	0,001585299
0,410185518	8,44E-04	0,015594173
0,410110465	0,002076579	0,02757875
0,41010148	0,001031423	0,017807642
0,410095666	0,001969817	0,026572839
0,409773784	0,001646195	0,023775293
0,409636892	0,002315337	0,029696046
0,409635835	1,86E-04	0,005938807
0,408887421	0,003068223	0,035591387
0,408629493	1,91E-04	0,006024593
0,408476744	0,002125895	0,028041413
0,408293869	0,004257529	0,043795131
0,408037526	0,004682272	0,046051476
0,407641649	0,003156481	0,036200382
0,40761945	5,03E-04	0,011290289
0,406989958	0,004446204	0,044727123
0,406915962	7,76E-04	0,014772651
0,406765856	0,002313858	0,029685627

0,406428647	0,001245698	0,020111774
0,406221459	0,001847929	0,025492144
0,40621723	0,002861302	0,034067536
0,406197146	0,001408206	0,021615132
0,406168605	0,002366947	0,030106589
0,405979915	2,09E-04	0,006402118
0,405338266	0,002353041	0,029998242
0,405226744	0,001213728	0,019803777
0,405088795	0,002038807	0,027166206
0,405034355	0,003332103	0,037585519
0,404103066	0,004934852	0,047755387
0,404058668	0,003168725	0,036303416
0,402888478	0,001449737	0,022032446
0,402386892	2,56E-04	0,007359599
0,401980444	0,003938456	0,041687333
0,401646406	0,001654793	0,023816186
0,401506871	6,63E-04	0,013370653
0,401204017	0,003334693	0,037586179
0,401178118	1,79E-04	0,005797805
0,40060518	0,003132319	0,036040925
0,400518499	0,00296286	0,034850176
0,400273256	0,005094901	0,048784879
0,3997426	6,95E-04	0,013735308
0,399442389	0,004544365	0,045295186
0,39934408	7,40E-06	8,19E-04
0,399334038	0,001916414	0,026097444
0,398921776	7,54E-04	0,014518574
0,398647992	3,45E-04	0,008794861
0,39840592	0,001970545	0,026574613
0,398201903	5,83E-04	0,012285835
0,397539112	8,21E-04	0,015322631
0,396778013	0,001166961	0,019331022
0,396675476	0,003127045	0,036011136
0,396604123	0,004512884	0,045112637
0,396565539	0,005113406	0,048920013
0,396491543	5,56E-04	0,012019926
0,39527907	2,66E-04	0,007532706
0,395153277	0,001300689	0,020651518
0,394410148	0,002972591	0,034881747
0,394368393	0,003088921	0,035717589
0,394121036	0,004435167	0,044650533
0,39373203	1,53E-04	0,005289296
0,393463002	0,001285766	0,02052802
0,393451374	0,001138028	0,019042637
0,39335148	0,002646063	0,032396337
0,3927537	1,91E-04	0,006024593
0,392392706	0,003455922	0,038392006
0,391912791	1,30E-04	0,004770434
0,39162315	0,005151632	0,04915382
0,391405391	7,42E-05	0,003290333
0,391152748	0,001579653	0,023214803
0,390815011	2,85E-04	0,007759204
0,390575581	0,004134584	0,043121266

0,390401691	0,001023671	0,017728772
0,390302326	0,001969742	0,026572839
0,38997463	0,005274147	0,049909489
0,389726216	9,56E-04	0,016978047
0,389664905	0,001363718	0,021224826
0,389390592	2,42E-04	0,007064463
0,389034884	0,002245284	0,029116138
0,388939218	2,33E-04	0,006874218
0,3887574	7,29E-05	0,003256747
0,388741015	5,34E-04	0,011749934
0,388540698	4,42E-04	0,010303274
0,388497886	0,001714376	0,024409086
0,387976216	0,001319113	0,020768523
0,387455074	0,003082799	0,035671111
0,386714059	0,004573533	0,045521965
0,386488901	0,001424754	0,021796405
0,385783827	0,002796642	0,033550662
0,38560148	0,004506233	0,045064623
0,385576638	0,001501503	0,022527092
0,38512685	6,97E-04	0,013756067
0,385091438	0,001787572	0,025021877
0,384933932	5,71E-04	0,012176757
0,384345137	0,001618071	0,023564177
0,38432611	0,004979374	0,047988148
0,383988901	0,00369596	0,039959428
0,383686047	5,56E-04	0,012019926
0,383263214	0,001380997	0,021365219
0,383033827	0,003298374	0,037392715
0,382961416	6,81E-04	0,013599132
0,382447674	3,16E-04	0,008314475
0,38185518	0,001870634	0,02570189
0,381621036	0,00185827	0,025571453
0,380840381	1,76E-04	0,005735638
0,380675476	0,002723121	0,033006444
0,380633192	0,001544977	0,022873018
0,380430761	0,002767854	0,033285986
0,380377378	4,71E-04	0,010746062
0,379904334	0,001977713	0,026629479
0,379669133	0,002945443	0,034740424
0,379457717	1,62E-04	0,005444242
0,379332452	0,002227898	0,028941237
0,378953488	0,001387295	0,021441933
0,37895296	1,42E-04	0,005027842
0,378928647	9,65E-04	0,017065388
0,37880444	8,46E-04	0,015628057
0,378603066	0,004303995	0,044022306
0,377998943	4,43E-05	0,002389556
0,377827167	0,005225246	0,049574727
0,377616279	4,15E-04	0,009972409
0,377244715	0,001745709	0,024685986
0,377139006	0,003686796	0,039893015
0,376467759	1,36E-04	0,004905178
0,376438161	9,81E-04	0,017273018

0,376341966	8,71E-04	0,01594887
0,37590592	0,00372421	0,040114248
0,375341966	0,002224348	0,028911983
0,374946089	0,001138102	0,019042637
0,374786469	0,001382547	0,021375969
0,374165433	0,001742968	0,024662889
0,373649577	2,45E-04	0,007135035
0,373447674	0,00150273	0,022537916
0,373357294	0,004320005	0,044057139
0,371133192	0,001324072	0,020811377
0,371094609	5,12E-04	0,011426534
0,37084408	0,002262033	0,029273677
0,370785941	5,38E-04	0,011786355
0,370580867	0,003730548	0,040148235
0,370097252	0,005042044	0,048445384
0,369784884	0,004466115	0,044829065
0,369709302	3,84E-04	0,009461867
0,369001586	0,004206637	0,043475742
0,368815011	0,001828449	0,025343382
0,368488901	0,00259103	0,031926649
0,368352537	0,003725966	0,040118302
0,368283298	9,29E-04	0,016717931
0,367940275	2,22E-04	0,006655488
0,367906977	0,001128227	0,018950138
0,367569767	5,15E-05	0,002595405
0,366669133	6,75E-04	0,0135127
0,36654334	2,90E-04	0,007855215
0,366016385	7,93E-04	0,014958655
0,365289112	5,87E-04	0,012347473
0,365163319	0,00296833	0,034868488
0,364861522	0,001894271	0,02590398
0,364054968	0,004545817	0,045299521
0,363909091	0,001926054	0,0261807
0,363474101	0,003989084	0,04205675
0,36317759	0,003139526	0,036089559
0,362510571	0,00120428	0,019678443
0,362150634	0,00430305	0,044022306
0,362058668	0,004526209	0,04517724
0,361541226	0,005219521	0,049543858
0,360947674	0,004316691	0,044057139
0,360569767	0,001260215	0,020250729
0,360218288	0,004663591	0,045932222
0,360123679	0,001941297	0,026336862
0,360011628	0,001362963	0,021224826
0,359748414	0,002668337	0,032555032
0,358882664	0,00517607	0,049307715
0,358721987	9,96E-04	0,017433268
0,358656977	0,001812819	0,025234445
0,358252114	0,001018823	0,017686066
0,357960888	0,002169712	0,028434136
0,357701903	0,004520777	0,045144306
0,356309725	0,005278482	0,049920316
0,356008457	2,21E-05	0,001585299

0,355484672	4,93E-04	0,011129617
0,355421776	8,66E-04	0,015885798
0,35512685	9,91E-04	0,01736895
0,353252114	0,001667709	0,023932489
0,353211416	0,002501719	0,031247278
0,352820296	5,13E-04	0,011433308
0,352370507	4,07E-06	5,79E-04
0,352059197	0,001141542	0,019058788
0,351692389	4,07E-04	0,009842211
0,350809197	0,003667625	0,039759237
0,350616279	0,002458458	0,030908714
0,350224101	3,15E-04	0,008303463
0,349732558	0,002631193	0,032296465
0,348669662	0,001755667	0,024756076
0,348507928	0,003004272	0,03511474
0,347746829	0,001479007	0,022287174
0,346887421	0,001202256	0,019655014
0,346445032	0,00243764	0,030716331
0,345786469	0,003340464	0,037613157
0,345765328	0,001066409	0,018294399
0,345701903	0,002692489	0,032759963
0,34564852	0,004139879	0,043156274
0,344578753	0,005197038	0,049439911
0,344525899	8,80E-04	0,016050303
0,343877378	7,38E-04	0,014316344
0,343709302	0,002576715	0,031794236
0,34289852	0,004596596	0,045621381
0,342284355	0,003487282	0,038527525
0,341176533	0,002108461	0,027855599
0,340553383	8,90E-04	0,016177196
0,340168605	2,79E-04	0,007731199
0,340022727	0,001783534	0,025021877
0,339021142	0,004275373	0,043925254
0,338950317	0,002822589	0,033725702
0,338856765	0,003264469	0,037113959
0,338391121	0,002229796	0,028949002
0,337188161	1,21E-04	0,004544245
0,337161205	0,002861906	0,034067536
0,336745243	0,004090729	0,0428072
0,336620507	0,001110236	0,018793982
0,336328224	0,003110345	0,035855897
0,334764271	0,002211946	0,028794261
0,33445296	0,00456946	0,045494415
0,33343129	0,002259204	0,029262565
0,333232558	0,001637194	0,023708445
0,331087209	4,02E-04	0,009780498
0,331015328	8,60E-04	0,015802141
0,330450317	1,47E-04	0,005136852
0,329684989	0,004430767	0,044644282
0,329598837	0,00200388	0,026861515
0,329457188	7,20E-04	0,014075384
0,328966702	0,001742625	0,024662889
0,328763742	0,002424361	0,030609623

0,32785148	0,004091017	0,0428072
0,327015328	0,004810181	0,046895312
0,326964059	7,66E-04	0,014661389
0,326939218	0,001301383	0,020651518
0,326896934	0,003705405	0,040012979
0,326233615	0,003145967	0,036144948
0,325841966	0,002272858	0,029379628
0,325664905	0,002862857	0,034069755
0,324752643	0,004784155	0,046684697
0,32437315	0,001259123	0,020240478
0,324036998	5,12E-05	0,002587139
0,322897992	8,45E-04	0,01561541
0,60663755	6,45E-06	1,64E-04
0,578250103	8,87E-08	4,61E-06
0,554209079	1,97E-05	4,09E-04
0,530944175	6,47E-14	4,64E-11
0,530549357	7,73E-05	0,001253932
0,528187914	2,44E-07	1,07E-05
0,521108482	4,51E-16	1,12E-12
0,519763623	2,10E-14	1,95E-11
0,518113034	8,11E-06	1,98E-04
0,513849789	1,06E-10	1,97E-08
0,51296253	1,06E-05	2,46E-04
0,511665683	2,44E-16	7,22E-13
0,511075744	7,02E-06	1,75E-04
0,507327346	1,24E-07	6,16E-06
0,506213871	1,16E-08	8,80E-07
0,501860086	5,64E-04	0,006192715
0,496369825	2,35E-06	7,11E-05
0,491704883	1,02E-08	7,91E-07
0,491326938	1,67E-09	1,77E-07
0,489714316	1,53E-09	1,66E-07
0,485393777	1,31E-06	4,34E-05
0,485199695	8,36E-16	1,72E-12
0,482502008	2,38E-05	4,76E-04
0,480479207	1,24E-04	0,001834693
0,479093689	1,33E-07	6,54E-06
0,478968329	1,95E-10	3,21E-08
0,478748129	1,34E-09	1,49E-07
0,477853087	3,74E-04	0,004468654
0,477566103	2,23E-05	4,54E-04
0,474998511	1,14E-12	4,83E-10
0,472669108	6,19E-09	5,26E-07
0,472532608	2,70E-04	0,003446856
0,471238732	4,48E-05	8,01E-04
0,471198047	9,75E-08	4,98E-06
0,470767134	1,56E-04	0,002215572
0,470191147	1,09E-14	1,14E-11
0,470150054	1,29E-13	7,42E-11
0,469900573	4,04E-12	1,34E-09
0,468991976	1,32E-10	2,38E-08
0,466917675	1,14E-13	6,93E-11
0,464717058	1,87E-05	3,92E-04

0,464053635	3,08E-04	0,003841094
0,463445741	2,21E-09	2,25E-07
0,460256689	0,002130587	0,017510556
0,460122417	4,32E-04	0,005021346
0,458049465	3,45E-15	4,50E-12
0,457604393	5,36E-04	0,005963129
0,45685268	1,20E-05	2,73E-04
0,45372934	2,33E-08	1,55E-06
0,45127649	1,18E-04	0,001759162
0,450491373	1,36E-10	2,43E-08
0,449910721	5,63E-09	4,82E-07
0,44815675	3,00E-07	1,26E-05
0,445891197	9,52E-16	1,81E-12
0,445681209	8,15E-04	0,008269392
0,44394114	2,37E-09	2,35E-07
0,44313077	2,30E-08	1,53E-06
0,442706741	8,30E-17	3,93E-13
0,442625198	7,20E-16	1,55E-12
0,441229122	5,94E-05	0,001005459
0,439030267	0,001085619	0,010298643
0,438951349	8,49E-11	1,68E-08
0,437608727	5,00E-04	0,00563742
0,43748624	2,84E-05	5,52E-04
0,437014976	4,87E-04	0,005514826
0,436390447	3,36E-10	5,06E-08
0,435126907	1,85E-08	1,28E-06
0,434440411	2,94E-07	1,24E-05
0,434103241	5,65E-10	7,62E-08
0,431933236	2,25E-06	6,86E-05
0,431125705	6,06E-07	2,26E-05
0,430833633	2,89E-04	0,003652367
0,430089494	4,42E-15	5,50E-12
0,429517708	1,50E-13	8,34E-11
0,428183346	7,54E-04	0,00778508
0,426359131	6,91E-06	1,73E-04
0,426205609	1,62E-12	6,24E-10
0,425054896	4,70E-12	1,52E-09
0,424606144	3,88E-14	3,16E-11
0,423864829	3,25E-08	2,04E-06
0,422967218	3,78E-09	3,51E-07
0,422913226	4,64E-08	2,73E-06
0,421695111	1,14E-12	4,83E-10
0,420634912	6,87E-12	2,11E-09
0,420027392	1,42E-09	1,58E-07
0,4188601	5,06E-10	7,07E-08
0,418734645	1,50E-08	1,09E-06
0,418665693	7,94E-12	2,30E-09
0,417254292	8,78E-12	2,49E-09
0,41680238	7,64E-10	9,74E-08
0,416517192	1,30E-08	9,64E-07
0,416120221	2,13E-09	2,19E-07
0,412976176	3,11E-15	4,21E-12
0,412558379	6,52E-39	3,09E-34

0,412406849	3,36E-09	3,20E-07
0,41065931	6,41E-11	1,32E-08
0,410398655	1,91E-05	3,99E-04
0,4091423	9,59E-09	7,53E-07
0,408701723	1,37E-11	3,62E-09
0,408434696	5,81E-09	4,96E-07
0,408186766	5,11E-15	6,20E-12
0,407620839	0,002323845	0,018744044
0,404419764	2,78E-19	6,57E-15
0,403968486	1,38E-15	2,18E-12
0,403492069	9,51E-11	1,82E-08
0,403259464	1,06E-12	4,69E-10
0,402909532	1,14E-08	8,65E-07
0,402119331	2,39E-06	7,20E-05
0,401761227	5,11E-07	1,95E-05
0,400550955	4,71E-07	1,82E-05
0,400476849	3,51E-06	9,86E-05
0,39921761	5,86E-16	1,39E-12
0,398364457	1,95E-04	0,002661579
0,397990905	1,21E-08	9,12E-07
0,397680411	3,82E-18	3,94E-14
0,39757424	3,02E-15	4,20E-12
0,397313842	1,78E-09	1,87E-07
0,396005092	1,17E-15	2,05E-12
0,395999798	4,91E-06	1,31E-04
0,395943415	2,69E-04	0,003444925
0,395645885	3,95E-16	1,04E-12
0,394758165	3,70E-16	1,03E-12
0,394509496	0,001757677	0,01506759
0,394407842	9,94E-16	1,81E-12
0,393290951	1,17E-05	2,69E-04
0,392937002	0,001309541	0,011938244
0,392537141	6,52E-11	1,34E-08
0,392452735	2,35E-09	2,34E-07
0,392447103	4,72E-13	2,33E-10
0,391378118	3,28E-10	4,97E-08
0,391123872	3,40E-05	6,40E-04
0,390525021	5,51E-08	3,16E-06
0,390079316	0,003644664	0,026284126
0,390048693	1,10E-05	2,55E-04
0,389934612	5,18E-04	0,005801626
0,389859644	8,10E-10	1,00E-07
0,388713507	5,90E-04	0,006431047
0,388245371	1,70E-08	1,19E-06
0,388170451	1,36E-11	3,62E-09
0,38753391	8,08E-09	6,49E-07
0,386840971	3,39E-13	1,71E-10
0,386488226	2,36E-09	2,35E-07
0,386275974	1,63E-16	5,95E-13
0,385779341	2,22E-08	1,48E-06
0,385573847	1,91E-06	6,00E-05
0,384606797	1,98E-13	1,06E-10
0,384532538	4,34E-09	3,93E-07

0,383829784	6,42E-05	0,001072876
0,382726424	1,24E-12	5,05E-10
0,382399649	6,08E-04	0,006578487
0,381978698	3,92E-04	0,004646963
0,381492817	6,80E-13	3,19E-10
0,381387376	3,23E-08	2,03E-06
0,381038551	6,68E-14	4,72E-11
0,380487431	1,36E-10	2,43E-08
0,380241802	2,11E-08	1,42E-06
0,379847837	7,95E-11	1,59E-08
0,379647216	7,70E-10	9,78E-08
0,379171599	4,93E-05	8,66E-04
0,378215444	1,78E-10	3,01E-08
0,378107111	2,65E-04	0,00339641
0,378066284	3,45E-04	0,004202067
0,377882026	3,52E-06	9,89E-05
0,377573464	5,38E-08	3,09E-06
0,377410177	7,92E-06	1,94E-04
0,377230631	6,09E-08	3,42E-06
0,377156841	1,46E-10	2,56E-08
0,377093975	1,35E-05	3,02E-04
0,376524543	9,36E-11	1,81E-08
0,376327802	3,33E-04	0,004079088
0,375710122	1,27E-17	1,00E-13
0,375285452	6,85E-10	8,91E-08
0,37519425	1,08E-12	4,69E-10
0,375159322	2,27E-05	4,61E-04
0,375153831	1,86E-05	3,91E-04
0,374961151	2,57E-10	4,00E-08
0,374903238	3,63E-08	2,24E-06
0,374781356	1,01E-09	1,19E-07
0,37456609	3,08E-11	7,32E-09
0,374128624	1,36E-11	3,62E-09
0,373832685	9,70E-04	0,009461625
0,372806142	8,04E-14	5,41E-11
0,372722825	6,26E-15	7,40E-12
0,372689934	3,19E-04	0,003961823
0,371936996	2,04E-14	1,93E-11
0,37184544	1,57E-05	3,40E-04
0,371229587	2,99E-14	2,57E-11
0,371201316	2,96E-12	1,03E-09
0,370815745	1,10E-07	5,53E-06
0,370171386	1,33E-04	0,001943025
0,370108881	3,13E-09	3,01E-07
0,368350399	6,06E-14	4,44E-11
0,368265382	0,003189081	0,023817903
0,367887075	1,48E-05	3,25E-04
0,367806579	7,33E-11	1,48E-08
0,367093561	1,16E-06	3,92E-05
0,367076189	1,00E-10	1,88E-08
0,365802493	2,05E-12	7,51E-10
0,364715168	3,50E-10	5,22E-08
0,36446894	6,98E-12	2,12E-09

0,364386082	4,56E-05	8,13E-04
0,364347044	1,85E-07	8,55E-06
0,364264646	1,32E-08	9,77E-07
0,364248226	6,25E-11	1,30E-08
0,364010465	2,59E-14	2,27E-11
0,364007419	1,73E-08	1,21E-06
0,363866448	4,72E-10	6,66E-08
0,36369945	2,22E-09	2,25E-07
0,363230962	0,001280928	0,01173391
0,362702901	1,90E-12	7,12E-10
0,362211388	8,97E-08	4,64E-06
0,362123915	1,61E-16	5,95E-13
0,362083554	0,001876638	0,015824688
0,362019978	8,77E-06	2,11E-04
0,361718343	8,26E-08	4,39E-06
0,36148212	1,28E-10	2,33E-08
0,361427054	5,19E-07	1,98E-05
0,361426861	5,54E-05	9,53E-04
0,361270344	1,30E-15	2,12E-12
0,361094596	9,29E-05	0,001448304
0,361021149	1,84E-12	7,02E-10
0,360966736	6,72E-04	0,007116311
0,360654588	4,28E-07	1,69E-05
0,36029623	8,14E-06	1,98E-04
0,359761578	0,001662167	0,01441428
0,359712787	1,10E-12	4,74E-10
0,359697985	2,42E-07	1,06E-05
0,359481735	9,55E-07	3,33E-05
0,359354644	6,44E-04	0,006893717
0,359260741	9,49E-04	0,009320699
0,358885404	6,78E-09	5,65E-07
0,358193223	5,06E-10	7,07E-08
0,357758507	2,09E-04	0,002806832
0,357419305	9,31E-06	2,21E-04
0,357054288	3,44E-09	3,26E-07
0,356544027	7,66E-14	5,25E-11
0,356300692	0,001487176	0,01318674
0,35567229	9,88E-05	0,001521234
0,355621751	7,37E-14	5,13E-11
0,355565824	6,42E-09	5,42E-07
0,35545704	6,52E-12	2,02E-09
0,355113789	1,77E-07	8,24E-06
0,354971015	2,45E-10	3,88E-08
0,354638749	5,16E-08	2,99E-06
0,353999038	6,69E-07	2,45E-05
0,353818147	1,67E-08	1,17E-06
0,353641607	9,64E-08	4,93E-06
0,352568994	0,008177286	0,048312138
0,35246289	0,003971826	0,028061919
0,352360953	0,008428882	0,049408611
0,352291923	3,67E-09	3,44E-07
0,352054515	1,22E-06	4,09E-05
0,351968344	1,40E-10	2,47E-08

0,35148768	5,96E-06	1,53E-04
0,350762283	1,21E-06	4,07E-05
0,350378502	7,23E-06	1,80E-04
0,350041982	3,78E-05	6,98E-04
0,34982894	0,001961256	0,016392176
0,349646272	2,00E-07	9,09E-06
0,349279113	2,13E-05	4,37E-04
0,349259502	7,87E-10	9,85E-08
0,349079941	1,14E-11	3,15E-09
0,348966245	8,32E-06	2,02E-04
0,348365814	3,14E-05	6,00E-04
0,348050807	5,81E-08	3,30E-06
0,347962563	6,32E-09	5,35E-07
0,347941088	5,79E-05	9,85E-04
0,347811059	6,55E-04	0,006975354
0,347164116	8,13E-15	9,38E-12
0,346965589	2,75E-06	8,09E-05
0,346653144	5,74E-14	4,31E-11
0,346090216	0,003205228	0,023894299
0,346013668	0,006528237	0,040978346
0,345893563	2,52E-06	7,54E-05
0,345844019	1,68E-15	2,49E-12
0,345791066	9,06E-05	0,001420442
0,345742579	1,53E-12	6,00E-10
0,345621489	1,16E-10	2,14E-08
0,345318596	3,41E-05	6,41E-04
0,344982364	8,82E-06	2,12E-04
0,344757026	5,21E-07	1,98E-05
0,344494248	2,93E-11	7,05E-09
0,344286545	3,37E-06	9,53E-05
0,343888479	9,44E-05	0,001466418
0,342834456	1,21E-08	9,12E-07
0,342494527	0,004750139	0,03224657
0,342404121	2,22E-07	9,95E-06
0,342173223	5,62E-09	4,82E-07
0,342083999	1,11E-09	1,29E-07
0,341984929	3,81E-07	1,54E-05
0,341964616	6,06E-08	3,41E-06
0,34154759	5,27E-10	7,30E-08
0,341335159	1,02E-10	1,92E-08
0,341063618	1,45E-04	0,002086458
0,340811912	1,58E-13	8,71E-11
0,340710944	0,004830392	0,032622899
0,340583368	9,61E-09	7,53E-07
0,340491747	3,25E-05	6,18E-04
0,340416031	2,55E-04	0,003293984
0,340339484	1,46E-05	3,20E-04
0,340273273	5,39E-10	7,37E-08
0,339512855	3,84E-14	3,16E-11
0,339463222	0,003928833	0,027812144
0,339398101	0,003431267	0,025124222
0,338478972	1,02E-11	2,85E-09
0,338434433	2,20E-12	8,00E-10

0,338362107	1,66E-05	3,56E-04
0,338227835	0,001982557	0,016538081
0,338188886	1,04E-07	5,29E-06
0,337944589	8,89E-09	7,06E-07
0,337881585	8,57E-07	3,03E-05
0,337301971	3,36E-11	7,88E-09
0,337163077	3,34E-04	0,004094603
0,336939262	2,01E-08	1,37E-06
0,336825564	2,98E-07	1,25E-05
0,336698121	8,03E-13	3,73E-10
0,336204858	1,22E-08	9,17E-07
0,336025626	1,53E-12	6,00E-10
0,335957444	9,64E-09	7,53E-07
0,335935083	1,13E-09	1,30E-07
0,335818769	8,57E-09	6,83E-07
0,335690274	2,59E-06	7,70E-05
0,335283598	2,92E-06	8,49E-05
0,335250703	2,18E-13	1,15E-10
0,335088891	3,82E-08	2,35E-06
0,334942947	9,10E-12	2,56E-09
0,33463419	3,81E-09	3,52E-07
0,334630981	4,80E-11	1,06E-08
0,334461309	1,53E-08	1,10E-06
0,334276932	6,36E-06	1,62E-04
0,334263371	4,44E-04	0,005125458
0,334252332	2,15E-09	2,20E-07
0,334082974	2,18E-07	9,83E-06
0,333980136	9,73E-16	1,81E-12
0,333203778	8,01E-05	0,001287923
0,33293478	6,26E-09	5,31E-07
0,332674727	4,55E-09	4,08E-07
0,332465739	7,16E-04	0,007478262
0,332351864	4,16E-18	3,94E-14
0,332294118	8,37E-07	2,98E-05
0,332157629	6,19E-08	3,46E-06
0,331864223	5,69E-05	9,73E-04
0,331802572	1,26E-06	4,22E-05
0,331623436	2,67E-07	1,15E-05
0,331546214	1,04E-10	1,95E-08
0,331217835	7,60E-08	4,10E-06
0,330949598	1,09E-05	2,52E-04
0,330875982	9,87E-05	0,001520237
0,330706465	1,66E-04	0,002333244
0,330599918	5,41E-05	9,35E-04
0,330419158	0,00403566	0,028398447
0,330255007	6,03E-08	3,40E-06
0,330155673	3,48E-08	2,16E-06
0,329953841	1,57E-06	5,05E-05
0,32987231	8,94E-10	1,08E-07
0,329609534	0,002703399	0,021031307
0,329450047	3,92E-08	2,39E-06
0,329418027	9,93E-11	1,87E-08
0,32918432	1,50E-09	1,64E-07

0,328901102	7,24E-08	3,93E-06
0,328824149	1,35E-04	0,001965281
0,32832459	1,42E-08	1,04E-06
0,327986668	4,73E-11	1,05E-08
0,327875762	5,00E-12	1,59E-09
0,327744232	1,33E-09	1,49E-07
0,327288676	6,15E-11	1,29E-08
0,327085762	2,92E-06	8,49E-05
0,327019297	1,69E-04	0,002374079
0,32680455	9,05E-08	4,67E-06
0,326759689	1,08E-09	1,28E-07
0,326725201	1,96E-06	6,12E-05
0,326687352	6,59E-13	3,12E-10
0,325928007	2,19E-04	0,002906282
0,325717041	5,10E-09	4,47E-07
0,325704674	4,66E-08	2,74E-06
0,325154032	3,52E-15	4,50E-12
0,324955328	9,04E-05	0,001418961
0,324751145	0,001036238	0,009948848
0,324741872	4,83E-08	2,82E-06
0,324604964	1,16E-09	1,33E-07
0,324556852	4,55E-08	2,69E-06
0,324523822	7,31E-07	2,66E-05
0,32433116	3,58E-05	6,68E-04
0,324312032	7,72E-09	6,27E-07
0,324061982	4,03E-08	2,43E-06
0,324060797	2,83E-08	1,81E-06
0,32397941	6,58E-06	1,66E-04
0,323937666	3,10E-12	1,05E-09
0,323891944	4,39E-08	2,61E-06
0,323882576	3,70E-12	1,23E-09
0,323856833	1,15E-09	1,32E-07
0,323618547	2,69E-08	1,75E-06
0,323146447	0,002817963	0,021711896
0,322480217	3,72E-10	5,50E-08
0,322186157	1,42E-08	1,04E-06
0,322039491	1,61E-09	1,73E-07
0,786771064	3,76E-05	0,007904345
0,555674328	4,47E-04	0,032977597
0,538541484	8,10E-05	0,012443541
0,527119762	6,15E-04	0,03854472
0,481715122	7,19E-09	4,68E-05
0,476966368	1,68E-06	0,001295115
0,469226488	8,04E-07	8,51E-04
0,46232366	5,09E-05	0,009373727
0,45597949	1,40E-04	0,01714243
0,454196874	3,69E-04	0,029172609
0,45156614	3,42E-04	0,028061639
0,433496782	1,82E-07	4,54E-04
0,432513422	8,42E-05	0,012691785
0,428384634	8,33E-08	2,93E-04
0,4281815	3,63E-05	0,007762381
0,423837436	5,03E-04	0,034476343

0,421276002	2,27E-04	0,022213247
0,420514566	5,93E-04	0,037986282
0,419881197	1,29E-05	0,004226654
0,417947823	3,65E-22	1,73E-17
0,414821932	2,43E-07	4,99E-04
0,410000121	1,27E-04	0,01617063
0,408335274	6,79E-06	0,002869788
0,403883355	3,09E-05	0,007174976
0,403296023	5,67E-04	0,037379805
0,400267573	2,62E-07	5,15E-04
0,398314695	6,36E-07	7,92E-04
0,392301414	7,25E-07	8,17E-04
0,390802539	4,00E-05	0,008200378
0,387562495	1,70E-05	0,005129671
0,387117789	8,68E-04	0,047180042
0,385335214	1,06E-06	0,001047269
0,384767362	2,69E-06	0,001702166
0,382353503	5,84E-04	0,037844749
0,381915221	8,82E-04	0,047580514
0,379921011	2,77E-04	0,025001422
0,379678885	7,46E-05	0,011806928
0,379283892	9,06E-04	0,048323595
0,379090278	7,48E-06	0,00309314
0,374163621	1,97E-04	0,020474862
0,369987555	8,09E-07	8,51E-04
0,365474167	3,42E-04	0,028061639
0,364550724	5,78E-09	4,56E-05
0,364293966	2,99E-05	0,00708452
0,357537792	1,51E-05	0,004711049
0,356139689	6,65E-04	0,04057153
0,355706087	4,00E-04	0,030515241
0,355536721	8,93E-07	9,18E-04
0,355282798	3,14E-06	0,001751528
0,353772772	1,77E-04	0,019439782
0,353174104	1,18E-08	6,19E-05
0,351671456	4,66E-04	0,033513331
0,351055288	8,14E-06	0,003185469
0,349425437	6,98E-05	0,011525206
0,348658594	2,02E-06	0,001432517
0,348070629	1,13E-04	0,015152552
0,347970295	2,06E-04	0,021101618
0,347362627	1,73E-04	0,019255853
0,347138722	1,02E-05	0,003691091
0,346873217	2,58E-05	0,00652458
0,346482667	2,02E-06	0,001432517
0,34586087	2,36E-04	0,022688356
0,34536944	2,35E-06	0,00153752
0,344567303	4,82E-09	4,56E-05
0,344197898	6,76E-08	2,67E-04
0,343993725	3,11E-06	0,001751528
0,343294645	5,31E-04	0,035808645
0,342603526	1,60E-06	0,001263198
0,341076455	2,72E-07	5,15E-04

0,340111621	2,24E-04	0,022119735
0,339033064	6,52E-04	0,040084405
0,338904952	2,87E-05	0,007056298
0,336480406	8,70E-04	0,047180042
0,336290196	3,15E-06	0,001751528
0,333176113	1,76E-04	0,019439782
0,331344303	3,78E-09	4,56E-05
0,330533954	3,22E-09	4,56E-05
0,329861216	3,99E-06	0,002073673
0,32968707	3,17E-05	0,00721118
0,328620362	5,10E-05	0,009373727
0,326854199	4,77E-04	0,03378353
0,324769306	6,41E-05	0,011065702
