# Supplementary material for: The Impact of Cand1 in Prostate Cancer
Source: Cancers (Basel). 2020 Feb 12;12(2):428. doi: 10.3390/cancers12020428 (PMC7072594; doi:10.3390/cancers12020428)
Supplement: Supplementary file 1 [file cancers-12-00428-s001.zip › Supplementary material revision/Supplementary_Table_S1.pdf]

		GSE16560
**n**	total	281
	with Cand1 expression data	280
**Age, years**	Mean	74,0
	SD	7,2
	Median	74,0
	Q25	69,0
	Q75	79,0
	Min	51,0
	Max	91,0
**Gleason Score**	Mean	7,2
	SD	1,1
	Median	7,0
	Q25	6,0
	Q75	8,0
	Min	6,0
	Max	10,0
**Follow-up, months**	Mean	102,1
	SD	56,4
	Median	100,0
	Q25	52,0
	Q75	149,0
	Min	6,0
	Max	274,0
**Survival event**	Type	Overall survival
	Events, number	206
	Events, % of total n	73,3
**log2 Cand1 expression**	Mean	12,3
	SD	0,5
	Median	12,4
	Q25	12,2
	Q75	12,6
	Min	9,9
	Max	13,1
	Stratification threshold	12,56
**n, Cand1 strata**	$Cand1^{lo}$	187
	$Cand1^{hi}$	93
	NA	1

GSE70768	GSE70769	GSE40272
112	93	87
111	92	86
60,5	NA	61,0
6,6		7,1
62,0		62,0
56,0		57,0
65,0		65,0
41,0		43,0
73,0		73,0
6,9	7,0	NA
0,5	0,9	
7,0	7,0	
7,0	7,0	
7,0	7,0	
6,0	5,0	
9,0	10,0	
35,6	78,9	35,6
17,1	21,0	25,6
34,0	79,6	32,4
21,0	67,9	16,0
50,8	92,2	59,0
2,0	1,8	0,0
66,8	122,3	91,8
Biochemical relapse-free survival	Biochemical relapse-free survival	Disease-free survival
19	45	86
17,0	48,4	100,0
8,2	8,6	-0,8
0,3	0,2	0,8
8,2	8,6	-0,8
8,0	8,5	-1,1
8,4	8,8	-0,4
7,6	8,0	-4,2
8,8	9,2	3,6
8,26	8,70	-0,79
67	64	44
44	28	42
1	1	1
